# Choice-selective sequences dominate in cortical relative to thalamic inputs to NAc to support reinforcement learning

**DOI:** 10.1016/j.celrep.2022.110756

**Published:** 2022-05-17

**Authors:** Nathan F. Parker, Avinash Baidya, Julia Cox, Laura M. Haetzel, Anna Zhukovskaya, Malavika Murugan, Ben Engelhard, Mark S. Goldman, Ilana B. Witten

**Affiliations:** 1 Princeton Neuroscience Institute, Princeton University, Princeton, NJ 08544, USA; 2 Department of Psychology, Princeton University, Princeton, NJ 08544, USA; 3 Center for Neuroscience, University of California, Davis, Davis, CA 95616, USA; 4 Department of Neurobiology, Physiology and Behavior, University of California, Davis, Davis, CA 95616, USA; 5 Department of Ophthalmology and Vision Science, University of California, Davis, Davis, CA 95616, USA; 6 Department of Physics and Astronomy, University of California, Davis, Davis, CA 95616, USA; 7 Department of Neuroscience, Feinberg School of Medicine, Northwestern University, Chicago, IL 60611, USA; 8 These authors contributed equally; 9 Lead contact

## Abstract

How are actions linked with subsequent outcomes to guide choices? The nucleus accumbens, which is implicated in this process, receives glutamatergic inputs from the prelimbic cortex and midline regions of the thalamus. However, little is known about whether and how representations differ across these input pathways. By comparing these inputs during a reinforcement learning task in mice, we discovered that prelimbic cortical inputs preferentially represent actions and choices, whereas midline thalamic inputs preferentially represent cues. Choice-selective activity in the prelimbic cortical inputs is organized in sequences that persist beyond the outcome. Through computational modeling, we demonstrate that these sequences can support the neural implementation of reinforcement-learning algorithms, in both a circuit model based on synaptic plasticity and one based on neural dynamics. Finally, we test and confirm a prediction of our circuit models by direct manipulation of nucleus accumbens input neurons.

## In brief

Learning to make proper choices requires associating outcomes with the actions that preceded them. Parker et al. record and optogenetically manipulate two input pathways to a key brain area associated with action-outcome learning, the nucleus accumbens. The results motivate two alternative models for the circuitry and mechanisms underlying reinforcement learning.

## Graphical Abstract

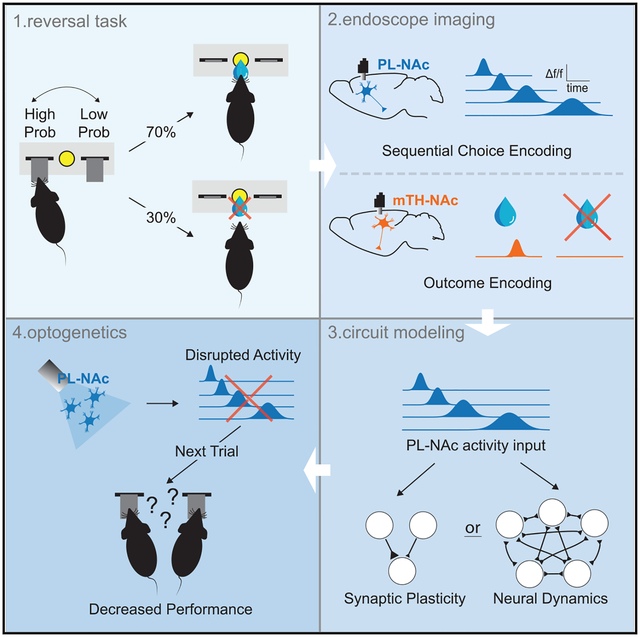

## INTRODUCTION

Multiple lines of experimental evidence implicate the nucleus accumbens (NAc, part of the ventral striatum) in reward-based learning and decision-making (Apicella et al., 1991; Cador et al., 1989; Carelli et al., 1993; Cox and Witten, 2019; Di Ciano et al., 2001; Everitt et al., 1991; Parkinson et al., 1999; Phillips et al., 1993, 1994; Robbins et al., 1989; Roitman et al., 2005; Setlow et al., 2003; Stuber et al., 2011; Taylor and Robbins, 1986). The NAc is a site of convergence of glutamatergic inputs from a variety of regions, including the prefrontal cortex and the midline thalamus, along with dense dopaminergic inputs from the midbrain (Brog et al., 1993; Do-Monte et al., 2017; Groenewegen et al., 1980; Hunnicutt et al., 2016; Otis et al., 2017; Phillipson and Griffiths, 1985; Poulin et al., 2018; Reed et al., 2018; Swanson, 1982; Wright and Groenewegen, 1995; Zhu et al., 2016).

A central question in reinforcement learning is how actions and outcomes become associated with each other, even when they are separated in time (Asaad et al., 2017; Gersch et al., 2014; Sutton, 1988; Wörgötter and Porr, 2005). A possible mechanism that could contribute to solving this problem of temporal credit assignment in the brain is that neural activity in the glutamatergic inputs to the NAc provides a neural memory trace of previous actions. However, whether glutamatergic inputs to the NAc indeed represent memories of previous actions is unclear. More broadly, what information is carried by glutamatergic inputs to the NAc during reinforcement learning, and whether different inputs provide overlapping or distinct streams of information, has not been examined systematically. To date, there have been relatively few recordings of cellular-resolution activity of glutamatergic inputs to the NAc during reinforcement learning or comparison of multiple inputs within the same task, nor examination of the timescale with which information is represented within and across trials. Furthermore, if glutamatergic inputs do indeed provide memories of previous actions, construction of a neurally plausible instantiation of an algorithm for credit assignment based on the measured signals remains to be demonstrated (for a review of biological instantiation of reinforcement learning algorithms, see Joel et al., 2002).

To address these gaps, we recorded from glutamatergic inputs to the NAc during a probabilistic reversal learning task and built circuit-based computational models to connect our data to prominent theories of reinforcement learning. In this task, dopamine neurons that project to the NAc encode reward prediction error (RPE), and inhibition of dopamine neurons substitutes for a negative RPE (Parker et al., 2016). To compare activity in major cortical and thalamic inputs to the NAc core, we combined a retrograde viral targeting strategy with cellular-resolution imaging to examine the inputs from prelimbic cortex (“PL-NAc,” part of the medial prefrontal cortex) and midline regions of the thalamus (“mTH-NAc”). We found that PL-NAc neurons preferentially encode actions and choices relative to mTH-NAc neurons, with choice-selective sequential activity that bridges the delay between choice and reward and that persists until the start of the subsequent trial. We demonstrate with computational modeling that these choice-selective sequences can support neural instantiations of reinforcement learning algorithms, either through dopamine-dependent changes in synaptic weights onto NAc neurons (Fisher et al., 2017; Gerfen and Sur-meier, 2011; Reynolds and Wickens, 2002; Russo et al., 2010) or dopamine-dependent changes in neural dynamics (Wang et al., 2018). Finally, we test and confirm a prediction of our models through direct optogenetic manipulation of PL-NAc neurons. Thus, by recording and manipulating glutamatergic inputs to the NAc and integrating these data with computational modeling, we provide specific proposals for how reinforcement learning could be implemented by neural circuitry.

## RESULTS

### Cellular-resolution imaging of glutamatergic inputs to the NAc during a probabilistic reversal learning task

**Figure 1.**
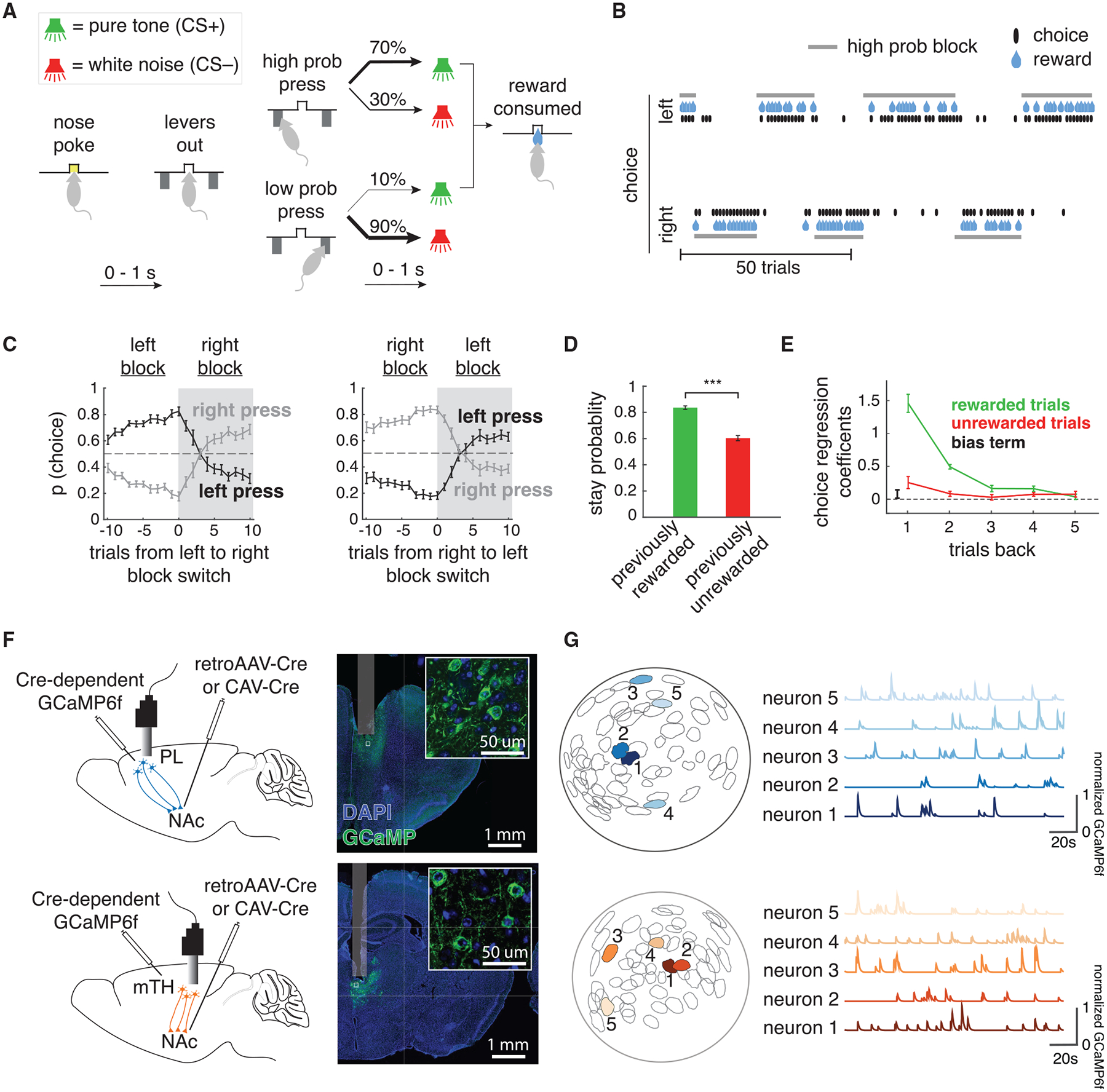
Cellular-resolution imaging of PL and mTH neurons that project to the NAc in mice performing a reinforcement learning task (A) Schematic of probabilistic reversal learning task. (B) Example behavior during a recording session. The choice of the mouse (black marks) follows the identity of the high-probability lever as it alternates between left and right (gray lines). (C) Left: probability the mice choose the left or right lever ten trials before and after a reversal from a left-to-right high-probability block. Right: same as left for right-to-left high-probability block reversals. (D) Mice had a significantly higher stay probability following a rewarded versus unrewarded trial (***p = 5 × 10 ^9^, two-tailed t test, n = 16 mice). (E) Coefficients from a logistic regression that uses choice and outcome from the previous five trials to predict choice on the current trial. Positive regression coefficients indicate a greater likelihood of repeating the previous choice. (F) Left: surgical schematic for PL-NAc (top) and mTH-NAc (bottom) recordings showing the injection site and optical lens implant with miniature head-mounted microscope attached. Right: coronal section from a PL-NAc (top) and mTH-NAc (bottom) mouse showing GCaMP6f expression in the recording sites. Inset: confocal image showing GCaMP6f expression in individual neurons. (G) Left: example field of view from a recording in PL-NAc (top, blue) and mTH-NAc (bottom, orange) with five representative regions of interest (ROIs). Right, normalized GCaMP6f fluorescence traces from the five ROIs on the left. For visualization, each trace was normalized by the peak fluorescence across the hour-long session. Data in (C), (D), and (E) are presented as mean ± SEM across mice (n = 16).

Mice performed a probabilistic reversal learning task while inputs from thalamus or cortex were imaged (Figure 1A). A trial was initiated when the mouse entered a central nose poke, which prompted the presentation of a lever on either side after a variable delay of 0–1 s. Each lever had either a high (70%) or low (10%) reward probability, with the identity of the high- and low-probability levers reversing in an unsignaled manner after a variable number of trials (see STAR Methods for block-reversal probabilities). After a variable delay (0–1 s), either a sound (positive conditioned stimulus [CS+]) was presented at the same time as a reward was delivered to a central reward port, or another sound (CS−) was presented that signaled the absence of reward.

As expected, mice switched the lever they were more likely to press following block reversals (Figures 1B and 1C). Similarly, mice were significantly more likely to return to the previously chosen lever (i.e., stay) following rewarded, as opposed to unrewarded, trials (Figure 1D), meaning that, as expected, mice were using previous choices and outcomes to guide behavior. A logistic regression to predict choice based on previous choices and outcomes indicated that mice relied on ~3 previous trials to guide their choices (Figure 1E; see STAR Methods for choice regression details).

To image activity of glutamatergic input neurons to the NAc during this behavior, we injected a retroAAV or CAV2 to express Cre-recombinase in the NAc as well as an AAV2/5 to Cre-dependently express GCaMP6f in either the PL or mTH (Figure 1F). A gradient refractive index (GRIN) lens was implanted above either the PL or mTH (see Figure S1 for implant locations), and a head-mounted miniature microscope was used to image activity in these populations during behavior (Figures 1F and 1G, n = 278 neurons in PL-NAc from n = 7 mice, n = 256 neurons in mTH-NAc from n = 9 mice). Behavior between mice in the PL-NAc versus mTH-NAc cohorts was similar (Figure S2).

### Actions are preferentially represented by PL-NAc neurons, while reward-predicting stimuli are preferentially represented by mTH-NAc neurons

**Figure 2.**
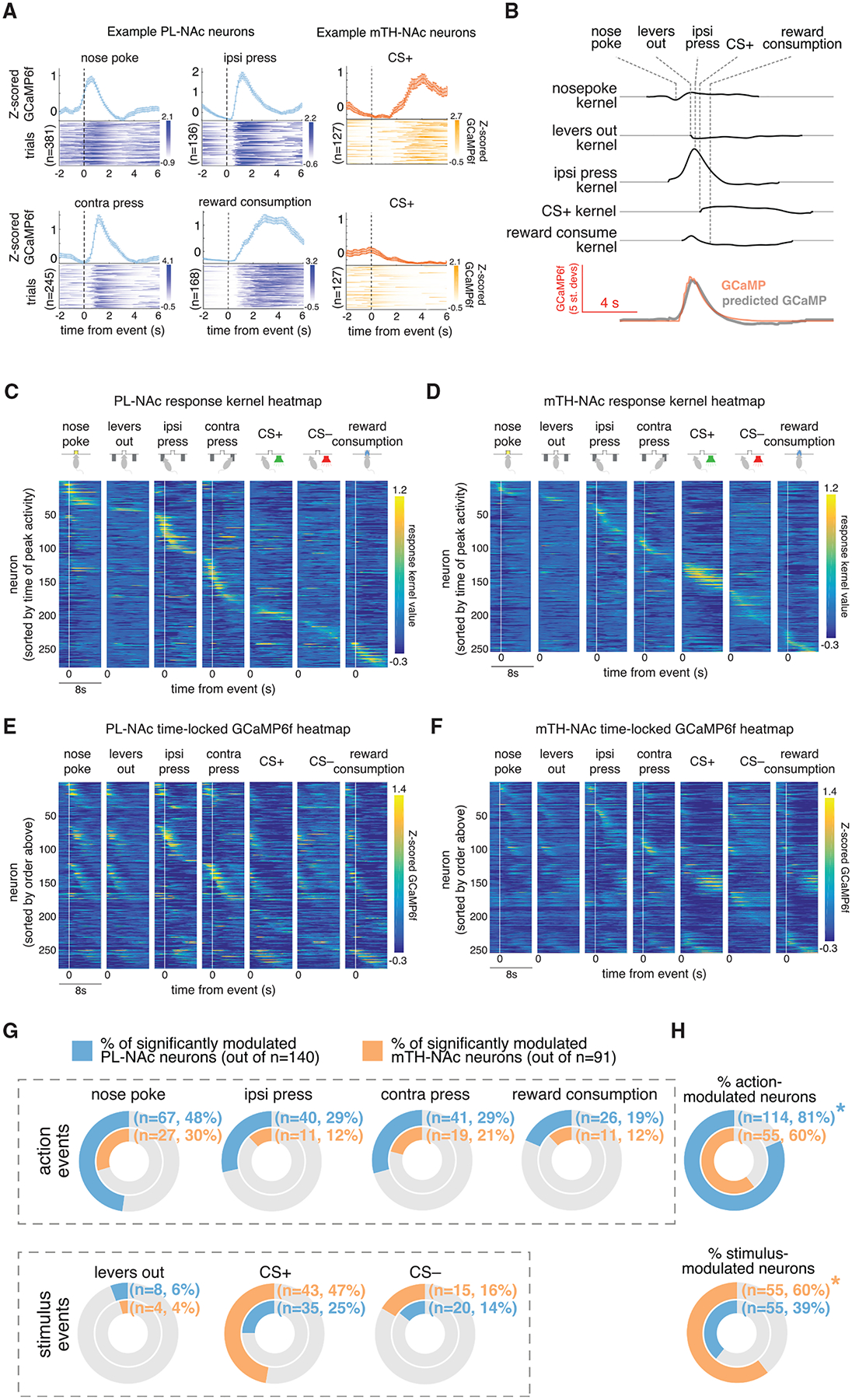
PL-NAc preferentially represents action events while mTH-NAc preferentially represents the CS+ (A) Time-locked responses of individual PL-NAc (blue) and mTH-NAc (orange) neurons to task events. Data are presented as mean ± SEM across trials. (B) Kernels representing the response to each of the task events for an example neuron, generated from the encoding model. The predicted GCaMP trace is the sum of the individual response kernels (see STAR Methods). (C) Heatmap of response kernels generated from the encoding model for all PL-NAc neurons. Heatmap is ordered by the time of the peak response across all behavioral events (n = 278 neurons, n = 7 mice). (D) Same as (C) except the heatmap of response kernels is from mTH-NAc neurons (n = 256 neurons, n = 9 mice). (E) Heatmap of mean *Z*-scored GCaMP6f fluorescence from PL-NAc neurons aligned to the time of each event in the task. Neurons are ordered as in (C). (F) Same as (E) for mTH-NAc neurons. (G) Top row: fraction of neurons significantly modulated by action events in the PL-NAc (blue) and mTH-NAc (orange). For all action events, PL-NAc had a larger fraction of significantly modulated neurons than mTH-NAc. Bottom row: fraction of neurons in PL-NAc (blue) and mTH-NAc (orange) significantly modulated by stimulus events. Two out of three stimulus events had a larger fraction of significantly modulated neurons in mTH-NAc than in PL-NAc. Significance was determined using the linear model used to generate response kernels in (B) (STAR Methods). (H) Top: a significantly larger fraction of event-modulated PL-NAc neurons encode at least one action event (p = 0.0004: two-proportion *Z* test comparing fraction of action-modulated PL-NAc and mTH-NAc neurons). Bottom: a significantly larger fraction of mTH-NAc neurons encode a stimulus event (p = 0.002: two-proportion *Z* test comparing fraction of stimulus-modulated neurons between PL-NAc and mTH-NAc). Asterisk denotes p < 0.05. For (G) and (H), fractions are determined using the total number of neurons significantly modulated by at least one task event (n = 140 for PL-NAc, n = 90 for mTH-NAc).

Individual PL-NAc and mTH-NAc neurons displayed elevated activity when time-locked to specific behavioral events in the task (Figure 2A). Given the correlation between the timing of task events, as well as the temporal proximity of events relative to the time course of GCaMP6f, we built a linear encoding model to properly relate neural activity to each event (Engelhard et al., 2019; Krumin et al., 2018; Lovett-Barron et al., 2019; Musall et al., 2019; Park et al., 2014; Parker et al., 2016; Pinto and Dan, 2015; Sabatini, 2019; Steinmetz et al., 2019). In brief, time-lagged versions of each behavioral event (e.g., nose poke, lever press) were used to predict the GCaMP6f fluorescence in each neuron using a linear regression. This allowed us to obtain “response kernels,” which related each event to the GCaMP6f fluorescence in each neuron, while removing the potentially confounding (linear) contributions of correlated task events (Figure 2B; see STAR Methods for details). To visualize the response kernels we plotted them as a heatmap, where each row was the response kernel for a particular neuron associated with each behavioral event. This heatmap was then ordered by the time of peak kernel value across all behavioral events. Visual observation revealed a clear difference between the PL-NAc and mTH-NAc populations: PL-NAc neurons were robustly modulated by the action events in our task (Figure 2C; kernel values associated with “nose poke,” “ipsilateral lever press,” “contralateral lever press,” and “reward consumption”) while mTH-NAc neurons appeared to be most strongly modulated by the stimulus events, specifically the positive reward auditory cue (Figure 2D, kernel values associated with “CS+”).

Examination of the GCaMP6f fluorescence time-locked to each behavioral event (rather than the encoding model-derived response kernels) revealed similar observations of action encoding in PL-NAc and CS+ encoding in mTH-NAc (Figures 2E and 2F). While this time-locked GCaMP6f heatmap displays neurons which appear to respond to multiple events (Figure 2E, see neurons approximately 70–170 that show elevated activity to “lever press,” “levers out,” and “nose poke”), this impression is likely a result of the temporal correlation between neighboring behavioral events, which our encoding model accounts for. To illustrate this, we applied our encoding model to a population of simulated neurons that responded only to the lever press events. We observed a similar multi-peak heatmap when simply time-locking the simulated GCaMP6f fluorescence, but this multi-peak effect is eliminated by the use of our encoding model, which recovers the true relationship between GCaMP6f fluorescence and behavior in the simulated data (Figure S3).

This encoding model was used to identify neurons in the PL-NAc and mTH-NAc populations that were significantly modulated by each event in our task (by comparing the encoding model with and without each task event, see STAR Methods). We found that a substantial fraction of both PL-NAc and mTH-NAc neurons were modulated by at least one task event (Figure 2G). Of these neurons that were selective to at least one task event, the selectivity for actions versus sensory stimuli differed between the two populations (Figure 2H). In particular, more PL-NAc neurons were modulated by at least one action event (nose poke, ipsilateral lever press, contralateral lever press, and reward consumption). By contrast, a significantly larger fraction of mTH-NAc neurons were modulated by at least one stimulus cue (levers out, CS+, and CS−).

### PL-NAc neurons preferentially encode choice relative to mTH-NAc neurons

**Figure 3.**
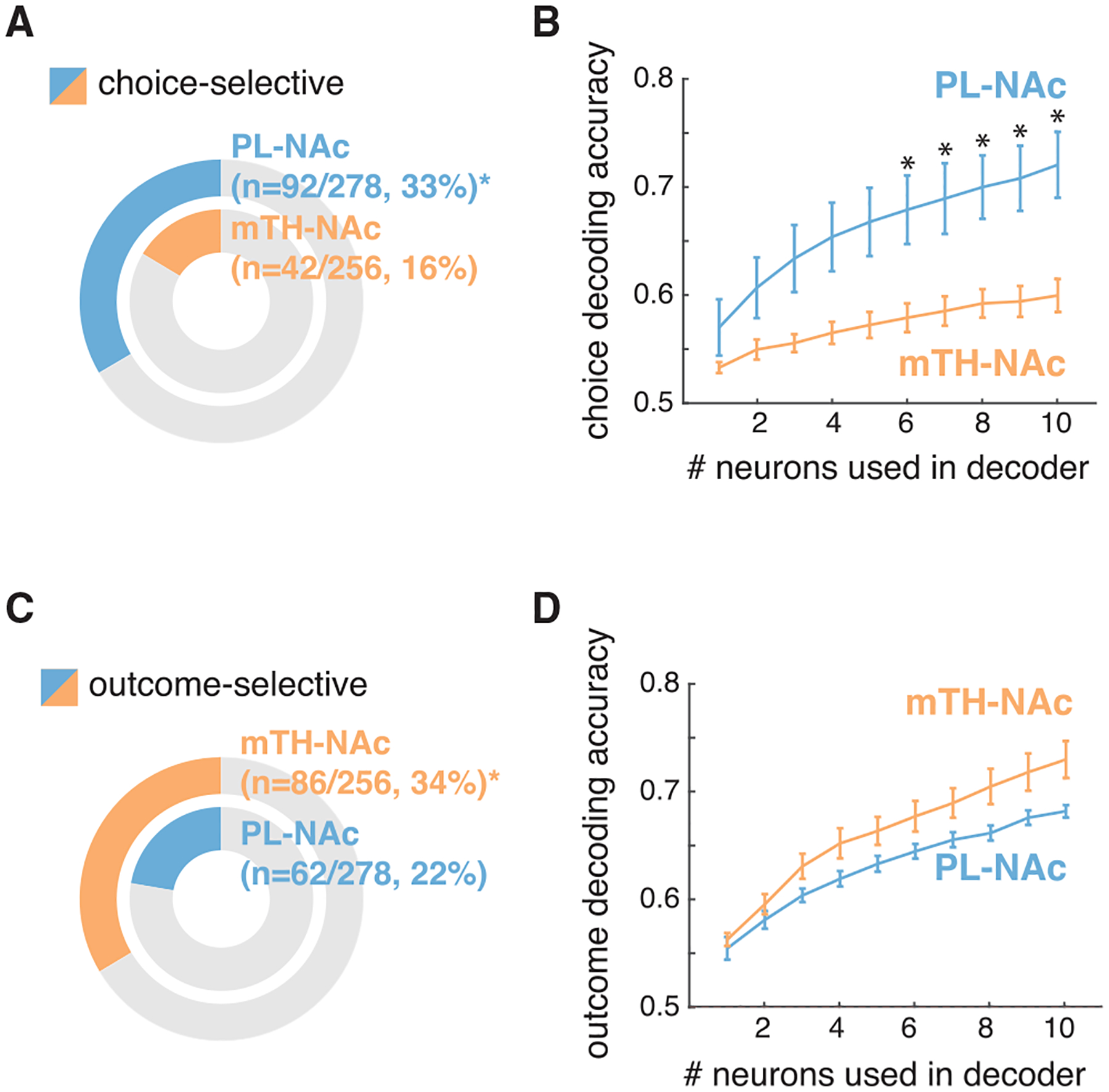
PL-NAc preferentially represents choice but not outcome relative to mTH-NAc (A) Fraction of choice-selective neurons in PL-NAc (n = 92 out of 278 neurons, 7 mice) and mTH-NAc (n = 42 out of 256 neurons, 9 mice). A significantly larger fraction of PL-NAc neurons was choice-selective compared with mTH-NAc neurons (p = 9.9 × 10 ^−6^: two-proportion *Z* test). (B) Choice decoding accuracy using randomly selected subsets of simultaneously imaged neurons around the lever press. The PL-NAc population more accurately decoded the choice of the trial compared with mTH-NAc (*p < 0.05, unpaired two-tailed t test, n = 9 PL-NAc and 6 mTH-NAc mice, peak decoding accuracy of 72% ± 3% for PL-NAc and 60% ± 2% for mTH-NAc). (C) Fraction of outcome-selective neurons in mTH-NAc (n = 86 out of 256 neurons, 9 mice) and PL-NAc (n = 62 out of 278 neurons, 7 mice). A significantly larger fraction of mTH-NAc neurons were outcome-selective compared with PL-NAc neurons (p = 0.004: two-proportion *Z* test). (D) Outcome decoding accuracy using neural activity after the time of the CS from randomly selected, simultaneously imaged neurons in mTH-NAc (orange, peak decoding accuracy: 73% ± 2%) and PL-NAc (blue, peak decoding accuracy: 68% ± 1%). p > 0.05, unpaired two-tailed t test. Data in (B) and (D) are presented as mean ± SEM across mice; n = 6 PL-NAc mice and 9 mTH-NAc mice. In (A) and (C) the asterisk denotes p < 0.05, two-proportion *Z* test.

This preferential representation of actions in PL-NAc relative to mTH-NAc suggests that lever choice (contralateral versus ipsilateral to the recording site) could also be preferentially encoded in PL-NAc. Indeed, a significantly larger fraction of neurons were choice-selective in PL-NAc compared with mTH-NAc (Figure 3A; significant choice selectivity was determined with a nested comparison of the encoding model with and without choice information, see STAR Methods). A logistic regression population decoder supported this observation of preferential choice selectivity in PL-NAc relative to mTH-NAc (Figure 3B).

In contrast to the preferential representation of choice in PL-NAc compared with mTH-NAc, a larger fraction of neurons in mTH-NAc encoded outcome (CS identity or reward consumption) compared with PL-NAc (Figure 3C). However, while outcome decoding accuracy in mTH-NAc was slightly higher relative to PL-NAc, this difference was not statistically significant (Figure 3D). These results suggest that, unlike the preferential choice representation observed in PL-NAc over mTH-NAc, outcome was more similarly represented between these two populations. This is presumably due to the fact that both CS+ and reward consumption responses contribute to outcome representation, and although more neurons encoded CS+ in mTH-NAc, the opposite was true for reward consumption (Figure 2G). We found no obvious relationship between the strength of either choice or outcome decoding and recording location in either PL-NAc or mTH-NAc (Figure S4).

### PL-NAc neurons display choice-selective sequences that persist into the next trial

**Figure 4.**
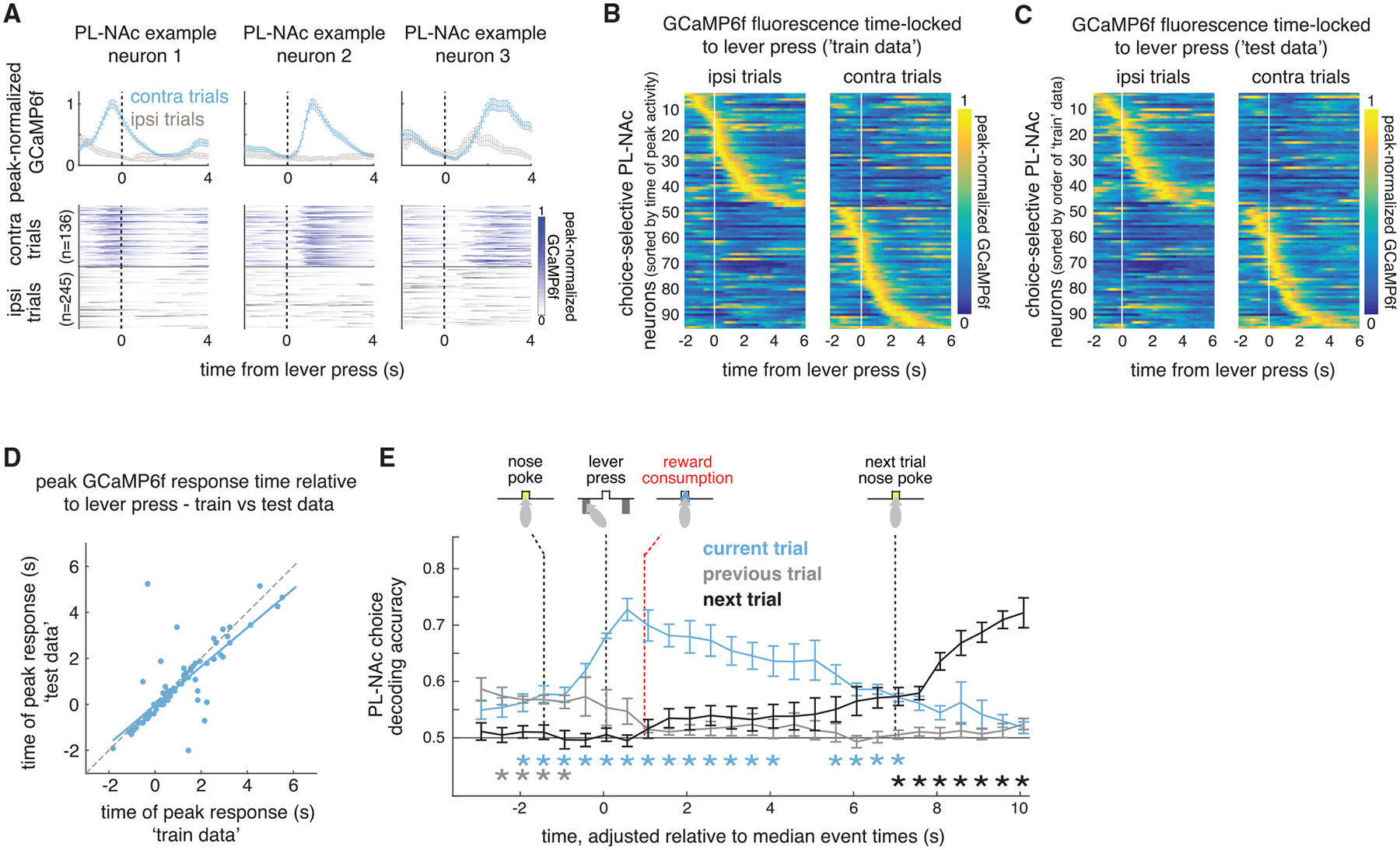
Choice-selective sequences in PL-NAc persist into the subsequent trial (A) Top: average peak-normalized GCaMP6f fluorescence of three simultaneously imaged PL-NAc choice-selective neurons. Data are presented as mean ± SEM across trials. Bottom: heatmaps of GCaMP6f fluorescence across trials aligned to ipsilateral (blue) and contralateral (gray) press. (B and C) Heatmaps showing sequential activation of choice-selective PL-NAc neurons (n = 92/278 neurons from 7 mice). Each row is a neuron’s average GCaMP6f fluorescence time-locked to the ipsilateral (left column) and contralateral (right column) lever press, normalized by its peak average fluorescence. In (B) (“train data”), heatmap is average fluorescence from half of trials and ordered by the time of peak activity. In (C) (“test data”), the peak-normalized, time-locked GCaMP6f fluorescence from the other half of trials was plotted in the order from “train data” in (B). (D) Correlation between time of peak activity using the “train” and “test” trials for choice-selective PL-NAc neurons in response to a contralateral or ipsilateral lever press (R^2^ = 0.80, p = 5.3 × 10^−22^, n = 92 neurons). (E) Average decoding accuracy of choice on the current (blue), previous (gray), and next (black) trial as a function of time-adjusted GCaMP6f fluorescence throughout the current trial from ten simultaneously imaged PL-NAc neurons. Data are presented as mean ± SEM across mice. Red dashed line indicates median onset of reward consumption. *p < 0.01, two-tailed, one-sample t test across mice comparing decoding accuracy to chance, n = 6 mice.

We next examined the temporal organization of choice-selective activity in PL-NAc neurons. Across the population, choice-selective PL-NAc neurons displayed sequential activity with respect to the lever press that persisted for >4 s after the press (Figures 4A–4C; see Figure S5 for sequences without peak normalization). These sequences were visualized by time-locking the GCaMP6f fluorescence of choice-selective neurons with respect to the lever press, rather than with the encoding model from the earlier figures. The robustness of these sequences was confirmed using a cross-validation procedure in which the order of peak activity across the PL-NAc choice-selective population was first established using half of the trials (Figure 4B, “train”), after which the population heatmap was plotted using the same established ordering and activity from the other half of the trials (Figure 4C, “test”). To quantify the consistency of these sequences, we correlated the neurons’ time of peak activity in the “training” and “test” data and observed a strong correlation (Figure 4D). Additionally, the ridge-to-background ratio, a metric used to confirm the presence of sequences (Akhlaghpour et al., 2016; Harvey et al., 2012; Kondo et al., 2017), was significantly higher when calculated using the PL-NAc choice-selective sequences compared with sequences generated using shuffled data (Figures S6A–S6C).

In contrast, choice-selective sequential activity in the mTH-NAc population was significantly less consistent than in PL-NAc (Figures S7A–S7D). Additionally, while the ridge-to-background ratio of the sequences generated using mTH-NAc activity was significantly higher than that using shuffled data, this ratio was also significantly lower than that obtained from PL-NAc sequences (Figures S6D–S6F). The ridge-to-background ratio of both the PL-NAc and mTH-NAc sequences did not significantly change across either a block or recording session (Figures S8A–S8D).

A striking feature of these choice-selective sequences in PL-NAc was that they persisted for seconds after the choice, potentially providing a neural “bridge” between choice and outcome. To further quantify the timescale of choice encoding both within and across trials, we used activity from simultaneously imaged neurons at each time point in the trial to predict the mouse’s choice (with a decoder based on a logistic regression using random combinations of ten simultaneously imaged neurons to predict choice). Choice on the current trial could be decoded above chance for ~7 s after the lever press, spanning the entire trial (including the time of reward delivery and consumption) as well as the beginning of the next trial (Figure 4E). Choice on the previous or subsequent trial was not represented as strongly as current-trial choice (Figure 4E; in all cases we corrected for cross-trial choice correlations with a weighted decoder, see STAR Methods) and choice from two trials back could not be decoded above chance at any time point (Figure S8E). We also examined the temporal extent of choice decoding in the mTH-NAc population (Figure S7E). Similar to PL-NAc, we observed that decoding persisted up to the start of the next trial. However, the peak decoding accuracy across all time points in the trial was lower in mTH-NAc (60% ± 0.1%) than in PL-NAc (73% ± 0.2%).

### Synaptic plasticity or neural dynamics models incorporating choice-selective sequences in PL-NAc neurons can reproduce behavioral and neural recordings

We next used computational modeling to explain how a biologically realistic circuit incorporating the observed choice-selective sequences in PL-NAc neurons could solve the probabilistic reversal task. We constructed two models of the observed trial-by-trial changes in choice probabilities, one based on synaptic plasticity and one based on slow neural dynamics. Each model sought to explain two features of our data: first, how choices made at an earlier time (around the time of the nose poke, when choice-selective activity appears, Figures 4B and 4C) could be reinforced by rewards that occur at a later time, and, second, how this reinforcement could persist across multiple trials as suggested by our choice regressions (Figure 1E).

#### Synaptic plasticity model

**Figure 5.**
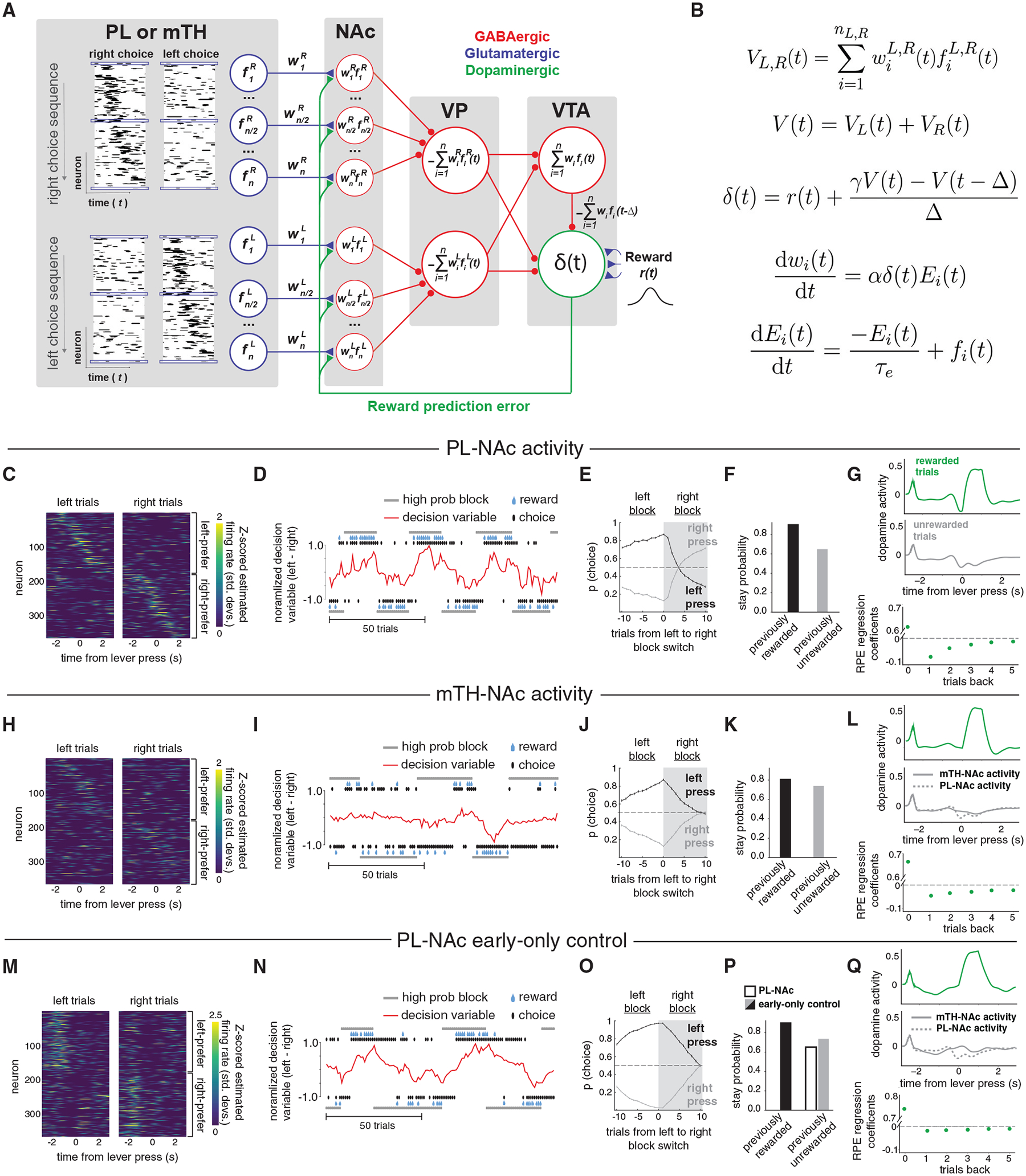
Choice-selective sequences recorded in PL-NAc, combined with known downstream connectivity, can implement a temporal difference (TD) learning model based on synaptic plasticity (A) Schematic of circuit architecture used in the model. Model implementation used single-trial recorded PL-NAc or mTH-NAc responses as input. See results and STAR Methods for model details and Figure S9 for alternative, mathematically equivalent circuit architectures. (B) Model equations. *V*: value; *V*_L_, *V*_R_: weighted sum of the *n*_L_ left-choice- or *n*_R_ right-choice-preferring NAc neuron activities $f_{i}^{L}$ and $f_{i}^{R}$, respectively, with weights $w_{i}^{L}$ or $W_{i}^{R}$; *α*: learning rate; *τ*_e_: decay time constant for the PL-NAc synaptic eligibility trace *E*(*t*); Δ: delay of the pathway through the VTA GABA interneuron; *γ*: discounting of value during time Δ. (C) Heatmap of single-trial PL-NAc estimated firing rates input to the model. (D) Behavior of the synaptic plasticity model for 120 example trials. The decision variable (red trace) and the choice of the model (black dots) follow the identity of the higher probability lever. (E) Probability the model chooses left (black) and right (gray) following a left-to-right block reversal. (F) Stay probability of the synaptic plasticity model following rewarded and unrewarded trials. (G) Top: simulated VTA dopamine neuron activity averaged across rewarded (green) and unrewarded (gray) trials. Bottom: coefficients from a linear regression that uses outcome of the current and previous five trials to predict dopamine neuron activity following outcome feedback (STAR Methods). (H–L) Same as (C) to (G), instead showing results from using estimated firing rates from mTH-NAc single-trial activity. The mTH-NAc model input generates worse performance than using PL-NAc input, with less and slower modulation of the decision variables, and weaker modulation of dopamine activity by previous trial outcomes. Dashed line in (L) shows results from PL-NAc model (same data as in G). (M) Control model including only early-firing neurons active at the onset of the sequence, when the model makes the choice. (N–Q) Same as (D) to (G), instead showing results from using the early-only control model. Open bar in (P) and dashed line in (Q) show results from PL-NAc model (same data as in F and G).

The synaptic plasticity model mathematically implemented a temporal difference (TD) reinforcement learning algorithm by combining the recorded choice-selective sequential activity of PL-NAc neurons with the known connectivity of downstream structures (Figures 5A and 5B). The goal of TD learning is to learn to predict the sum of future rewards, or “value” (Dayan and Niv, 2008; O’Doherty et al., 2003; Sutton and Barto, 1998; Tsitsiklis and Van Roy, 1997). When this sum of expected future rewards changes, such as when an unexpected reward is received or an unexpected predictor of reward is experienced, a TD RPE occurs and adjusts the weights of reward-predicting inputs to reduce this error. The error signal in the TD algorithm closely resembles the RPE signal observed in ventral tegmental area (VTA) dopamine neurons (Parker et al., 2016; Schultz, 1998; Schultz et al., 1997), but how this signal is computed remains an open question.

In our model, the PL-NAc sequences (Figure 5C) enabled the calculation of the RPE in dopamine neurons which, in turn, reinforced those PL-NAc inputs that lead to better-than-predicted rewards. In more detail, the model took as inputs experimental, single-trial recordings of choice-selective, sequentially active PL neurons (Figure 5A, left; see STAR Methods). These inputs represented temporal basis functions *f*_i_(*t*) for computing the estimated value of making a left or right choice. These basis functions are weighted in the NAc by the strength *w*_i_ of the PL-NAc synaptic connection and summed together to create a (sign-inverted) representation of the estimated value, at time *t*, of making a left choice, *V*_L_(*t*), or right choice, *V*_R_(*t*). To create the RPE observed in dopamine neurons requires that the dopamine neuron population receive a fast, positive value signal *V*(*t*) and a delayed negative value signal *V*(*t*–Δ), as well as a direct reward signal *r*(*t*) (Figure 5B). In Figure 5A, the summation of NAc inputs and sign inversion occurs in the ventral pallidum (VP) (Kimura et al., 1996; Oorschot, 1996), so that the fast value signal is due to direct VP to VTA dopamine input. The delayed negative value signal to the dopamine population is due to a slower, disynaptic pathway that converges first upon the VTA *γ*-aminobutyric acid (GABA) neurons, so that these neurons encode a value signal as observed experimentally (Cohen et al., 2012). The temporal discounting factor *γ* is implemented through different strengths of the two pathways to the VTA dopamine neurons (Figure 5B). Other mathematically equivalent circuit architectures, including those involving other structures such as the lateral habenula (Li et al., 2019), are given in Figure S9. Learning is achieved through dopamine-dependent modification of the PL-NAc synaptic strengths. We assume that PL-NAc neuronal activity leads to an exponentially decaying synaptic “eligibility trace” (Gerstner et al., 2018; Sutton and Barto, 1998). The correlation of this presynaptically driven eligibility trace with dopamine input then drives learning (Figure 5B). Altogether, this circuit architecture (as well as those shown in Figure S9) realizes a TD learning algorithm for generating value representations in the NAc, providing a substrate for the selection of proper choice based on previous trial outcomes.

The synaptic plasticity model was able to correctly perform the task and recapitulate the mice’s behavior. It achieved a comparable rate of reward (47.2% for the model, 47.6% for the mice) and exhibited similar alternation of choice following block reversals (Figures 5D and 5E; compare with Figures 1B and 1C; choice was based upon a probabilistic readout, at the start of the sequence, of the difference between right and left values plus a stay-with-previous choice bias [STAR Methods]) and similarly higher stay probability following rewarded relative to unrewarded trials (Figure 5F; compare with Figure 1D).

Model neuron responses resembled those previously observed experimentally. The RPE signal within a trial showed characteristic positive response to rewarded outcomes and negative response to unrewarded outcomes (Figure 5G; compare with Figures S10A and S10B) and had similar dependence upon previous trial outcomes (Figure 5G, multiple linear regression similar to Bayer and Glimcher, 2005; Parker et al., 2016; Figures S10C and S10D). The VTA GABA interneuron had a sustained value signal, due to the converging input of the transient, sequential value signals from NAc/VP (Figure S11), replicating the sustained value signal in VTA GABA interneurons observed in monosynaptic inputs to VTA dopamine neurons (Cohen et al., 2012). Alternatively, the VP neurons shown in Figure 5A could project to a second set of VP neurons that functionally take the place of the VTA GABA interneurons (Figures S9A, S9C, and S9F), leading to sustained positive value encoding VP neurons as observed in VTA-projecting VP neurons (Tian et al., 2016).

We next ran the same model using single-trial activity from choice-selective mTH-NAc neurons instead of PL-NAc (Figure 5H). In line with the less consistent sequential choice-selective activity in mTH-NAc relative to PL-NAc (Figures 4 and S7), the correct value after a block switch was learned much more slowly within the NAc and VTA GABA neurons (Figures S11C and S11D), leading to correspondingly slow changes in choice probability (Figures 5I and 5J). As a result, choice probabilities were often out of sync with the current block, leading to overall reward rate near chance levels (38.7% reward rate, chance rate of 40%). Stay probabilities were inappropriately high following unrewarded trials (Figure 5K), reflecting reduced formation of an RPE and thus less negative modulation of dopamine signal at the time of expected reward (Figure 5L).

The choice-selective sequences in PL-NAc neurons were critical to model performance, as they allowed proper formation of an RPE at the time of reward receipt. This was verified by generating a control model that only included early-firing PL-NAc neurons (neurons active at the onset of the sequence when the model makes its choice) (Figure 5M). This “early-only control” model failed to quickly modulate lever value following block reversals (~10 trials to reverse following a block switch rather than ~3 trials for the full PL-NAc data; Figures 5N–5P). The inferior performance of this control model (model reward rate: 43.9%) reflected two factors. First, the early-only control model was unable to generate a well-timed RPE signal due to the absence of significant PL-NAc input activity at the time of reward. As a result, on unrewarded trials there was almost no negative reward-predictive dip in dopamine activity at the time of reward omission, unlike for the model with the full PL-NAc input activity (Figure 5Q). This lack of learning from unrewarded trials is evident in the stay probability plot (Figure 5P), which shows less modulation by unrewarded trials when controlling (by adjusting the model’s action-selection parameters) for the stay probability following rewarded trials. Second, unlike the sequential model, the RPE in the early-only control model could not propagate backward across successive trials, so single-trial learning (enabled by the eligibility trace) was the only mechanism available to bridge the gap in time between the firing of the early-firing decision neurons and an RPE occurring at the time of reward.

#### Neural dynamics model

**Figure 6.**
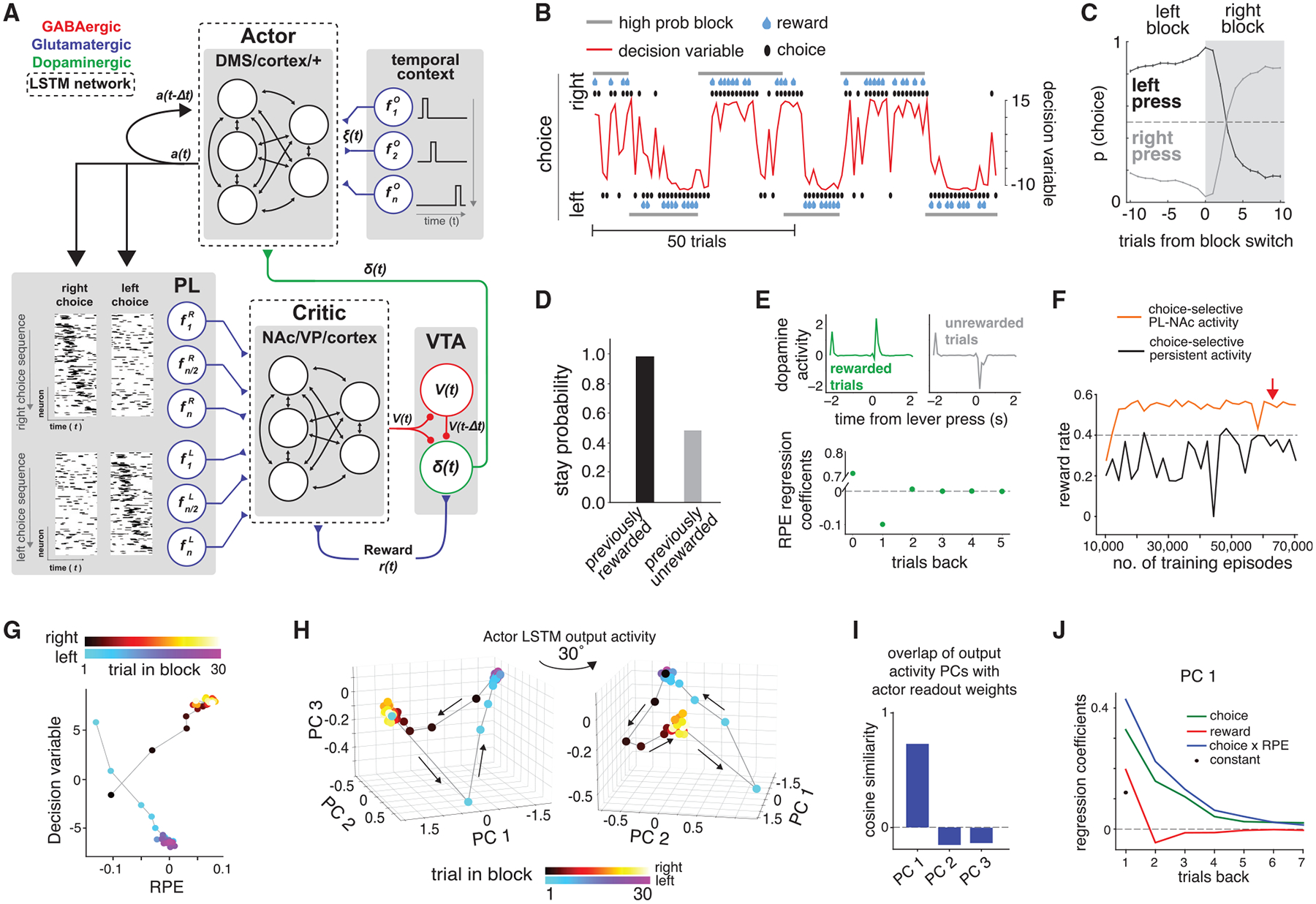
Neural dynamics model, with recorded choice-selective PL-NAc activity input to the critic, performs the task similarly to synaptic plasticity model (A) Model schematic. See results and STAR Methods for details. (B–E) Example behavior and dopamine activity from the neural dynamics model. Panel descriptions are the same as those for the synaptic plasticity model (Figures 5D–5G). (F) Reward rate as a function of the number of training episodes for the model with recorded PL-NAc input to the critic (orange) and for a model with persistent choice-selective input to the critic (black). Red arrow indicates the training duration used to generate all other figure panels. Gray dashed line indicates chance reward rate of 0.4. (G) Relationship between the decision variable used to select the choice on the next trial and the calculated RPE across right and left blocks. The RPE shown is an average of 0–2 s after lever press, averaged across blocks. The decision variable is also averaged across blocks. (H) Evolution of the principal components of the output of the actor LSTM units across trials within a right and left block. The displayed activity is from the first time point in each trial (when the choice is made), averaged across blocks. The first three components accounted for 70.9%, 16.6%, and 6.4% of the total variance at this time point, respectively. (I) Cosine of the angle between the actor network’s readout weight vector and the vectors corresponding to the first three principal components (PCs). Network activity in the PC1 direction (but not PC2 or PC3) aligns with the network readout weights. (J) Coefficients from a linear regression that uses choice on the previous trial (green), average RPE from 0–2 s after the lever press (red), and “choice × RPE” interaction (blue) from the previous seven trials to predict the amplitude of activity in PC1 on the current trial.

The synaptic plasticity model described above requires fast, dopamine-mediated synaptic plasticity, on the timescale of a trial, to mediate behavior. Whether plasticity operates in the NAc on this timescale is unclear. We thus developed an alternative model (Figure 6A and STAR Methods) in which the across-trial updating of values and corresponding selection of actions is accomplished through the dynamics of a recurrent neural network rather than the dynamics of synaptic plasticity (Botvinick et al., 2019, 2020; Doshi-Velez and Konidaris, 2016; Song et al., 2017; Wang et al., 2018). The initial learning of the neural network’s synaptic weights is based on a reinforcement learning algorithm, which models slow initial task acquisition, but during task performance synaptic weights remain fixed and the dopamine RPE serves only to alter neural dynamics.

Similar to the synaptic plasticity model, single-trial, experimentally recorded PL-NAc activity was input to a (now recurrent) neural network that modeled NAc and other associated brain regions (the “critic network”) to calculate value. RPE was calculated in the dopamine neurons from the value signal using the same circuit architecture as the synaptic plasticity model. However, rather than reweighting PL-NAc synapses on the timescale of trials, the RPE was input to a second recurrent neural network that modeled dorsomedial striatum (DMS) and other associated brain regions (the “actor network;” Atallah et al., 2007; Lau and Glimcher, 2008; O’Doherty et al., 2004; Richard et al., 2016; Tsutsui et al., 2016). This actor network used the RPE input from the previous timestep, the action from the previous timestep, and a “temporal context” sequence that may arise from hippocampus or other cortical or subcortical areas (Akhlaghpour et al., 2016; Howard and Eichenbaum, 2013; Leon and Shadlen, 2003) to generate a decision variable corresponding to the probability of selecting one of three choices (left, right, or nothing) at any time. Selection of the left or right choice then triggered the onset of the corresponding PL-NAc activity sequence.

The neural dynamics model appropriately modulated choice following a reversal in the identity of the high-probability lever (Figure 6B–6D) and generated RPE signals in VTA dopamine neurons that resemble previous experimental recordings (Figures 6E and S10). By contrast, when we replaced the choice-selective sequences to the NAc by choice-selective persistent activity, the model failed to train within the same number of training episodes (Figure 6F). This suggests that temporal structure in this input is beneficial for efficient task learning.

To reveal how the model appropriately modulates its choices, we analyzed the evolution of the actor network’s activity across trials (Figures 6G–6J). We found that the actor network’s activity at the time of decision was low-dimensional, with the first three principal components explaining ~94% of the variance. Given the symmetry in the block structure, the average RPE signal as a function of trial number is similar for the left and right blocks. However, the model should make opposite choices for left and right blocks, meaning that the actor network needs to respond oppositely to similar RPE inputs. Consistent with this, the decision variable for a given RPE was approximately opposite for left versus right blocks (Figure 6G). At a block reversal, for example from a left block to a right block, the network activity rapidly transitioned from the approximately steady-state representation of the left block (cluster of blue-purple points in Figure 6H) to the approximately steady-state representation of the right block (cluster of red-yellow points). Furthermore, the model learned to align the first principal component of activity along the direction of the network readout weights that determine the actor’s choice *a*(*t*) (Figure 6I). Thus, the actor learned to generate an explicit representation of the decision variable in the first principal component of its activity.

To solve the reversal learning task, the network needs to use its past history of choices and rewards to accumulate evidence for whether the current block is a left block or a right block. Rewarded left-side choices, or unrewarded right-side choices, represent evidence that the current block is a left block, while the converse represents evidence for a right block. In the synaptic plasticity model (Figure 5), new evidence (not accounted for by previous expectations) is accumulated in the PL-NAc synaptic weights as the product of the eligibility trace (which, due to the choice selectivity of the PL-NAc activity, represents the current choice) and the RPE. To analyze whether the actor network uses a similar accumulation of evidence to solve the task, we linearly regressed the first principal component of actor activity (PC1, which correlated strongly with the decision variable as described above) against the past history of choices and RPEs, which serve as inputs to the network, as well as the product of these (“choice × RPE”). PC1 most strongly depended upon the “choice × RPE” predictor, with coefficients that decayed on a timescale of approximately three trials, suggesting that the actor used a leaky accumulation of evidence over this timescale to solve the task (Figure 6J, blue trace). In addition, like the mice and the synaptic plasticity model, the neural dynamics model tended to stay with its previous choices, as evident from the positive coefficients for the previous choice regressors in Figure 6J (green trace). Thus, both the synaptic plasticity model and the neural dynamics model follow the same principle of accumulating evidence across trials to perform fast reversal learning in addition to having a tendency to repeat their previous choices.

### Stimulation of PL-NAc (but not mTH-NAc) neurons decreases the effect of previous trial outcomes on subsequent choice in both the models and the mice

**Figure 7.**
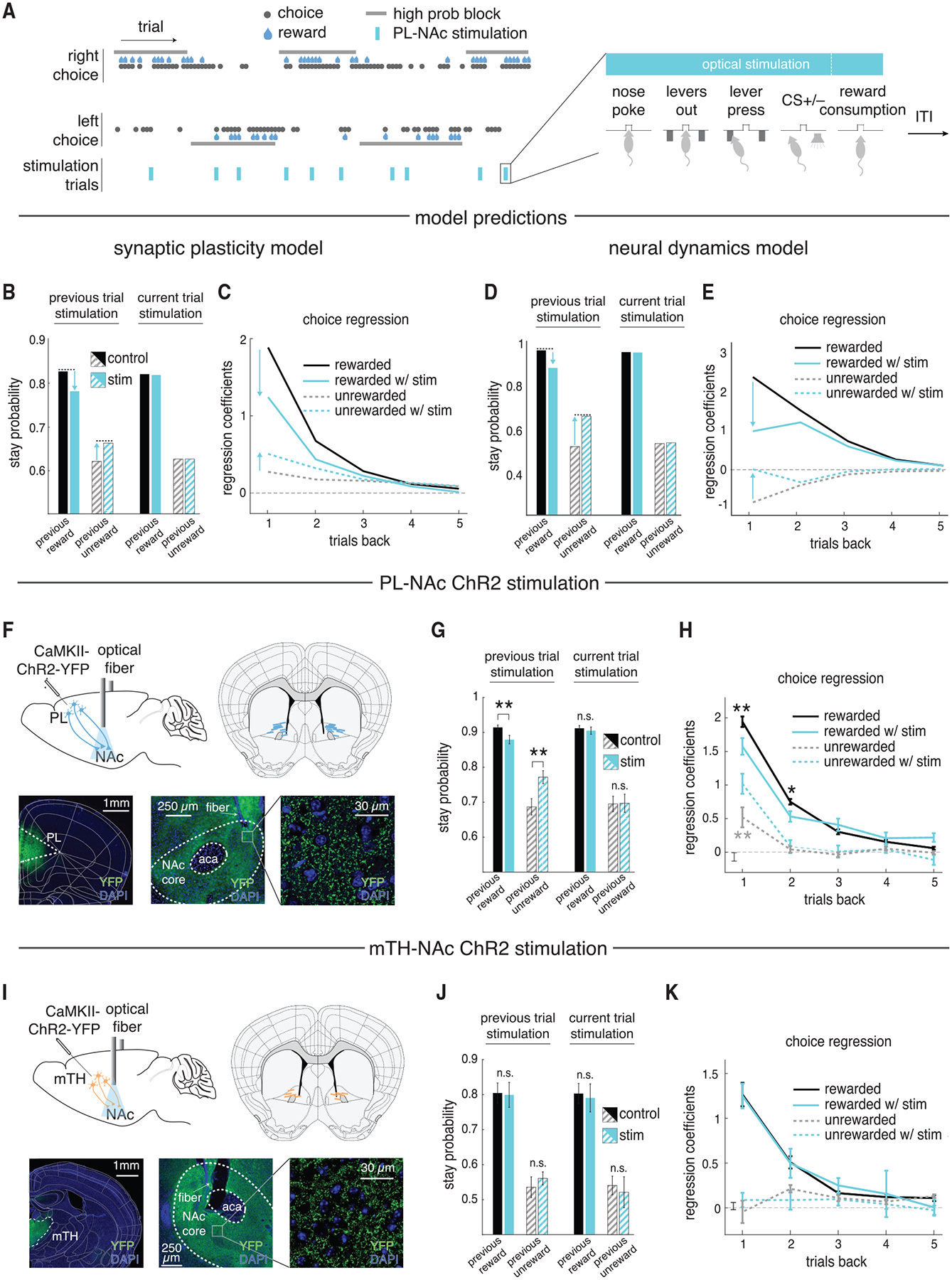
Stimulation of PL-NAc neurons disrupts the influence of previous trial outcomes on subsequent choice in both the models and mice (A) In the mice and models, PL-NAc neurons were stimulated for the whole trial on a random 10% of trials, disrupting the endogenous choice-selective sequential activity (see STAR Methods and Figure S13). (B) Effect of stimulating the PL-NAc input on the previous (left) or current (right) trial in the synaptic plasticity model. (C) Logistic choice regression showing dependence of the current choice on previously rewarded and unrewarded choices, with and without stimulation. Higher coefficients indicate a higher probability of staying with the previously chosen lever. (D and E) Same as (B) and (C) for the neural dynamics model. (F) Top left: schematic illustrating injection site in the PL (black needle) and optical fiber implant in the NAc core. Top right: location of optical fiber tips of PL-NAc ChR2 cohort (n = 14 mice) Bottom left: coronal section showing ChR2-YFP expression in PL. Bottom middle and right: ChR2-YFP expression in PL terminals in the NAc core. (G) Similar to the models, PL-NAc ChR2 stimulation on the previous trial significantly reduced the mice’s stay probability following a rewarded trial (p = 0.002) while increasing stay probability following an unrewarded trial (p = 0.0005). Stimulation on the current trial had no significant effect on stay probability following rewarded (p = 0.62) or unrewarded (p = 0.91) trials. All comparisons were paired two-tailed t tests, n = 14 mice. (H) PL-NAc ChR2 stimulation decreased the weight of rewarded choices one and two trials back (p = 0.002: one trial back; p = 0.023: two trials back) and increased the weight of unrewarded choices one trial back (p = 5.4 × 10^−6^). (I–K) Same as (F) to (H) for mTH-NAc ChR2 stimulation (n = 8 mice). mTH-NAc stimulation had no significant effect on stay probability following either rewarded (p = 0.85) or unrewarded choices (p = 0.40) on the previous trial back (J, paired t test, n = 8 mice) or multiple trials back (K, p > 0.05 for all trials back, one-sample t tests). Current-trial stimulation also had no effect following either rewarded (p = 0.59) or unrewarded (p = 0.50) choices. **p < 0.005 and *p < 0.05 for one-sample two-tailed t tests.

We next generated experimentally testable predictions from our models by examining the effect of disruption of the PL-NAc inputs on behavioral performance. To do so, we simulated optogenetic-like neural stimulation of this projection by replacing the PL-NAc sequential activity in the model with constant, choice-independent activity across 70% of the population on a subset of trials (Figure 7A). For both models, this generated a decrease in the probability of staying with the previously chosen lever following rewarded trials and an increase following unrewarded trials relative to unstimulated trials (Figures 7B and 7D). In other words, the effect of previous outcome on choice was reduced when PL-NAc activity was disrupted. This effect persists for multiple trials, as revealed by a logistic regression of current-trial choice on the history of previous rewarded and unrewarded choices with and without stimulation (Figures 7C and 7E; note that the negative coefficients for unrewarded trials in the neural dynamics model reflect that, unlike the synaptic plasticity model, this model does not include an explicit stay-with-previous choice bias). This reduced effect of outcome on choice arises because the stimulation disrupts the calculation of value. In the synaptic plasticity model, the stimulation of both left- and right-preferring PL-NAc neurons has two effects: first, it disrupts the RPE calculation by the circuit; second, it leads to dopamine indiscriminately adjusting the synaptic weights (i.e., value) of both the right and left PL-NAc synapses following rewarded or unrewarded outcomes. These weight changes then persist for multiple trials, leading to decreased performance in subsequent trials. In the neural dynamics model, stimulation reduces behavioral performance on subsequent trials by disrupting the RPE signal that is transmitted to the actor, and this effect lasts for multiple trials because the actor network temporally accumulates RPE signals across multiple trials (Figure 6J). In both models, the choice behavior on the current trial is unaffected because choice is determined at the beginning of the trial, before the weights are updated (Figures 7B and 7D).

We tested these model predictions experimentally by performing an analogous optogenetic manipulation in mice (Figure 7F). In close agreement with our models, mice significantly decreased their stay probability following a rewarded trial that was paired with stimulation and significantly increased their stay probability following an unrewarded trial paired with stimulation (Figure 7G). Similar to the models, the effect of stimulation on the mouse’s choice persisted for multiple trials. Mice had a significant decrease in their stay probability following PL-NAc stimulation on rewarded choices one and two trials back (Figure 7H). Also similar to the model, stimulation on the current trial had no significant effect on choice following either rewarded or unrewarded trials (Figure 7G).

In contrast to PL-NAc stimulation, but consistent with the relatively weak choice encoding in mTH-NAc compared with PL-NAc (Figures 3A and 3B) and weak trial-by-trial learning in our synaptic plasticity model (Figures 5H–5K), mTH-NAc stimulation (Figure 7I) had no significant effect on the mice’s stay probability on the subsequent trial following either rewarded or unrewarded stimulation trials (Figure 7J). Similarly, inclusion of mTH-NAc stimulation in our choice regression model revealed no significant effect of stimulation on rewarded or unrewarded choices (Figure 7K). Additionally, there was no effect on the mice’s stay probability for current-trial stimulation (Figure 7J).

For both PL-NAc and mTH-NAc stimulation, we observed an increase in the probability of mice abandoning the trials with stimulation compared with those trials without (p = 0.0006 for PL-NAc and p = 0.032 for mTH-NAc, paired two-tailed t test comparing percentage of abandoned trials on stimulated versus non-stimulated trials; 12.2% ± 2.5% and 22.1% ± 7.9% abandoned for PL-NAc and mTH-NAc stimulated trials, respectively; 0.9% ± 0.2% and 6.4% ± 3.1% for PL-NAc and mTH-NAc non-stimulated trials, respectively). Relatedly, we also found an increase in the latency to initiate a trial following either PL-NAc or mTH-NAc stimulation (Figures S12A–S12C). Together, these results suggest that this manipulation had some influence on the mouse’s motivation to perform the task. However, unlike the stronger effect of PL-NAc versus mTH-NAc stimulation on subsequent choice behavior, this trial-abandonment effect was stronger for mTH-NAc than for PL-NAc.

To control for non-specific effects of optogenetic stimulation, we ran a control cohort of mice that received identical stimulation but did not express the opsin (Figures S12E and S12F). Stimulation had no significant effect on the mice’s choice behavior (Figures S12D, S12G, and S12H) or probability of abandoning trials on stimulation versus control trials (p = 0.38, paired two-tailed t test comparing percentage of abandoned trials on stimulated with non-stimulated trials; 0.4% ± 0.08% for stimulated trials, 0.4% ± 0.01% for non-stimulated trials).

## DISCUSSION

This work provides both experimental and computational insights into how the NAc and associated regions could contribute to reinforcement learning. Experimentally, we found that mTH-NAc neurons were preferentially modulated by a reward-predictive cue while PL-NAc neurons more strongly encoded actions (e.g., nose poke, lever press). In addition, PL-NAc neurons display choice-selective sequential activity which persists for several seconds after the lever-press action, beyond the time the mice receive reward feedback. Computationally, we demonstrate that the choice-selective and sequential nature of PL-NAc activity can contribute to performance of a choice task by implementing a circuit-based version of reinforcement learning based on either synaptic plasticity or neural dynamics. Furthermore, PL-NAc perturbations affect future but not current choice in both the models and mice, consistent with perturbation of the critic, not the actor.

### Relationship to previous neural recordings in the NAc and associated regions

To our knowledge, a direct comparison, at cellular resolution, of activity across multiple glutamatergic inputs to the NAc has not previously been conducted. The preferential representations of actions relative to sensory stimuli in PL-NAc is somewhat surprising, given that previous studies have focused on sensory representations in this projection (Otis et al., 2017), and also given that the NAc is heavily implicated in Pavlovian conditioning (Day and Carelli, 2007; Day et al., 2006; Di Ciano et al., 2001; Parkinson et al., 1999; Roitman et al., 2005; Wan and Peoples, 2006).

On the other hand, there is extensive previous evidence of action correlates in prefrontal cortex (Cameron et al., 2019; Genovesio et al., 2006; Luk and Wallis, 2013; Siniscalchi et al., 2019; Sul et al., 2010), and NAc is implicated in operant conditioning in addition to Pavlovian conditioning (Atallah et al., 2007; Cardinal and Cheung, 2005; Collins et al., 2019; Hernandez et al., 2002; Kelley et al., 1997; Kim et al., 2009; Salamone et al., 1991). Our finding of sustained choice encoding in PL-NAc neurons is in agreement with previous work recording from medial prefrontal cortex neurons during a different reinforcement learning task (Maggi and Humphries, 2019; Maggi et al., 2018). Additionally, other papers have reported choice-selective sequences in other regions of cortex as well as in the hippocampus (Harvey et al., 2012; Pastalkova et al., 2008; Terada et al., 2017). In fact, given previous reports of choice-selective (or outcome-selective) sequences in multiple brain regions and species (Kawai et al., 2015; Kim et al., 2017; Long et al., 2010;Ölveczky et al., 2011; Picardo et al., 2016; Sakata et al., 2008), the relative absence of sequences in mTH-NAc neurons may be more surprising than the presence in PL-NAc.

Our observation of prolonged representation of the CS+ in mTH-NAc (Figures 2D and 2F) is in line with previous observations of pronounced and prolonged encoding of task-related stimuli in the primate thalamus during a Pavlovian conditioning task (Matsumoto et al., 2001). Together with our data, this suggests that the thalamus is contributing information about task-relevant stimuli to the striatum, which could potentially serve to bridge the gap between a CS and unconditioned stimulus (US) in a Pavlovian trace conditioning task (Campus et al., 2019; Do-Monte et al., 2017; Otis et al., 2019; Zhu et al., 2018).

### Implementation of reinforcement learning in models based on synaptic plasticity or neural dynamics

We presented two different classes of models that could solve the reversal learning task when provided with the choice-selective sequences observed in PL-NAc neurons as inputs. In our synaptic plasticity model, we show how these sequences may contribute to a neural implementation of TD learning by providing a temporal basis set that bridges the gap in time between actions and outcomes and enables the calculation of RPE in dopamine neurons. Other forms of neural dynamics, such as constant or slowly decaying persistent activity, can also maintain values across a delay period. However, creating a temporally precise RPE from such persistent activity is challenging if the persistent activity does not have sharp temporal features. Likewise, synaptic eligibility traces are another useful mechanism for bridging gaps in time, enabling earlier inputs to be reinforced by an RPE, but they do not provide the active input required to create the RPE itself.

A limitation of the synaptic plasticity model for producing the rapid reversals of behavior at block switches is that it requires a dopamine-dependent synaptic plasticity mechanism that operates on the timescale of trials (Figure 5). Whether dopamine-mediated synaptic plasticity operates on such fast timescales is not clear. Furthermore, model-free TD learning cannot take advantage of additional task-structure information such as the reward probabilities within a block (Collins and Cockburn, 2020; Doll et al., 2012; but see Figure S14 for challenges in identifying this ability within tasks like ours). These observations motivated the neural dynamics model in which, following initial slow-timescale learning of synaptic weights, the plasticity was turned off and trial-by-trial modulation of behavior was mediated by dopamine-dependent neural dynamics instead of synaptic plasticity (Figure 6; see related work by Botvinick et al., 2019, 2020; Doshi-Velez and Konidaris, 2016; Nagabandi et al., 2018; Song et al., 2017; Wang et al., 2018; Sæmundsson et al., 2018; Finn et al., 2017; Duan et al., 2016; Rakelly et al., 2019). Because the recurrent “critic” network dynamics can be trained to construct a temporally rich representation, the neural dynamics model has less need for precise temporal sequences in the PL-NAc inputs. However, we found that strictly eliminating the temporal structure of the PL-NAc input while preserving the choice selectivity made training of the network less efficient (Figure 6F), suggesting that having temporal structure in PL-NAc inputs facilitates the calculation of value.

Previous work in biological TD learning has used sequentially active neurons as the basis for learning in the context of sequential behaviors (Fee and Goldberg, 2011; Jin et al., 2009) and learning the timing of a CS-US relationship (Aggarwal et al., 2012; Carrillo-Reid et al., 2008; Gershman et al., 2014; Ponzi and Wickens, 2010). Likewise, our neural dynamics model was inspired by a previous meta-reinforcement learning model that was used to solve a reversal learning task (Wang et al., 2018). Here we extend these ideas in multiple important ways:

First, we link these theoretical ideas directly to data, by demonstrating that choice-selective sequential activity in the NAc is provided primarily by PL-NAc (as opposed to mTH-NAc) input neurons and that perturbation of the PL-NAc (but not mTH-NAc) projection disrupts action-outcome pairing consistent with model predictions. As such, our models provide a mechanistic explanation of a puzzling experimental finding: that optogenetic manipulation of PL-NAc neurons affects subsequent choices but not the choice on the stimulation trial itself, and that this stimulation creates oppositely directed effects following rewarded versus unrewarded trials.

Second, both of our models replicate numerous experimental findings in the circuitry downstream of PL-NAc. Each calculates an RPE signal in dopamine neurons (Bayer and Glimcher, 2005; Parker et al., 2016), generates conjunctive encoding of actions and outcomes (Kim et al., 2009, 2013), and calculates chosen value signals (Lau and Glimcher, 2008). Additionally, both models generate encoding of value by GABA interneurons (Cohen et al., 2012; Tian et al., 2016), which produces the temporally delayed, sign-inverted signals required for the calculation of a temporally differenced RPE (Figure 5A; see Aggarwal et al., 2012; Carrillo-Reid et al., 2008; Doya, 2002; Hazy et al., 2010; Ito and Doya, 2015; Joel et al., 2002; Pan et al., 2005; Suri and Schultz, 1998, 1999). Consistent with our models, electrical stimulation of VP generates both immediate inhibition of dopamine neurons and delayed excitation (Chen et al., 2019). Conceptually, the proposed temporal differencing by the VTA GABA interneuron is attractive in that it could provide a generalizable mechanism for calculating RPE: it could extend to any pathway that projects to both the dopamine and GABA neurons in the VTA (Beier et al., 2015) and that also receives a dopaminergic input that can modify synaptic weights.

Third, we showed that the fundamental operating principle of both models was similar: each temporally accumulates the correlation of previous choices with reward to determine the current-trial choice probability. In the synaptic plasticity model, this accumulation is done in the PL-NAc synaptic weights (Figure 5B). In the neural dynamics model, the accumulation is done in the low-dimensional neural dynamics of the actor network (Figure 6J). Future experiments that exploit these differences will need to be designed and executed to determine whether the brain more closely resembles the synaptic plasticity or neural dynamics model.

### Limitations of the study

A limitation of this study is that we could not artificially recapitulate sequential firing to directly test its role in constructing value representations. Additionally, any artificial stimulation can have off-target and unintended consequences. Thus, further work directly investigating the causal role of PL-NAc sequences in reinforcement learning is needed. Neither of our models account for the influence of glutamatergic inputs to NAc from regions other than prelimbic cortex and the medial thalamus. In addition, our neural dynamics model used long short-term memory (LSTM) units, which should not be interpreted as single neurons but might model computations performed by larger populations. Finally, single-photon imaging limits the ability to resolve single z planes during imaging and, thus, can make single neuron identification difficult. Future studies confirming our studies with other methods may helpful.

## STAR★METHODS

### RESOURCE AVAILABILITY

#### Lead contact

Further information and requests for resources and reagents should be directed to and will be fulfilled by the lead contact, Ilana Witten (iwitten@princeton.edu).

#### Materials availability

This study did not generate unique reagents.

#### Data and code availability

- Microscopy data reported in this paper will be shared by the lead contact upon request. Behavioral and one-photon imaging data used in this paper will be shared by the lead contact upon request.
- Original code related to the synaptic plasticity and neural dynamics models (Figures 5 and 6) and the event encoding model (Figure 2) has been deposited at GitHub and is publicly available as of the date of publication. The URLs are listed in the key resources table. All other code used in this study is available from the lead contact upon request.
- Any additional information required to reanalyze the data reported in this paper is available from the lead contact upon request.

### EXPERIMENTAL MODEL AND SUBJECT DETAILS

#### Mice

46 male C57BL/6J mice from The Jackson Laboratory (strain 000664) were used for these experiments. Prior to surgery, mice were group-housed with 3–5 mice/cage. All mice were >6 weeks of age prior to surgery and/or behavioral training. To prevent mice from damaging the implant of cagemates, all mice used in imaging experiments were singly housed post-surgery. All mice were kept on a 12-h on/12-h off light schedule. All experiments and surgeries were performed during the light off time. All experimental procedures and animal care was performed in accordance with the guidelines set forth by the National Institutes of Health and were approved by the Princeton University Institutional Animal Care and Use Committee.

### METHOD DETAILS

#### Probabilistic reversal learning task

Beginning three days prior to the first day of training, mice were placed on water restriction and given per diem water to maintain >80% original body weight throughout training. Mice performed the task in a 21 × 18 cm operant behavior box (MED associates, ENV-307W). A shaping protocol of three stages was used to enable training and discourage a bias from forming to the right or left lever. In all stages of training, the start of a trial was indicated by illumination of a central nose poke port. After completing a nose poke, the mouse was presented with both the right and left lever after a temporal delay drawn from a random distribution from 0 to 1s in 100ms intervals. The probability of reward of these two levers varied based on the stage of training (see below for details). After the mouse successfully pressed one of the two levers, both retracted and, after a temporal delay drawn from the same uniform distribution, the mice were presented with one of two auditory cues for 500ms indicating whether the mouse was rewarded (CS+, 5 kHz pure tone) or not rewarded (CS−, white noise). Concurrent with the CS + presentation, the mouse was presented with 6μL of 10% sucrose reward in a dish located equidistantly between the two levers, just interior to the central nose poke. The start time of reward consumption was defined as the moment the mouse first made contact with the central reward port spout following the delivery of the reward. The end of the reward consumption period (i.e., reward exit) was defined as the first moment at which the mouse was disengaged from the reward port for >100ms. In all stages of training, trials were separated by a 2s intertrial interval, which began either at the end of CS on unrewarded trials or at the end of reward consumption on rewarded trials.

In the first stage of training (“100–100 debias”), during a two-hour session, mice could make a central nose poke and be presented with both the right and left levers, each with a 100% probability of reward. However, to ensure that mice did not form a bias during this stage, after five successive presses of either lever the mouse was required to press the opposite lever to receive a reward. In this case, a single successful switch to the opposite lever returned both levers to a rewarded state. Once a mouse received >100 rewards in a single session they were moved to the second stage (“100–0”) where only one of the two levers would result in a reward. The identity of the rewarded lever reversed after 10 rewarded trials plus a random number of trials drawn from the geometric distribution:

(Equation 1) $$P(k)={\left(1-p\right)}^{k-1}p$$

where *P*(*k*) is the probability of a block reversal *k* trials into a block and *p* is the success probability of a reversal for each trial, which in our case was 0.4. After 3 successive days of receiving >100 total rewards, the mice were moved to the final stage of training (“70–10”), during which on any given trial pressing one lever had a 70% probability of leading to reward (high-probability lever) while pressing the opposite lever had only a 10% reward probability (low-probability lever). The identity of the higher probability lever reversed using the same geometric distribution as the 100–0 training stage. On average, there were 23.23 ± 7.93 trials per block and 9.67 ± 3.66 blocks per session (mean +/− std. dev.). In this final stage, the mice were required to press either lever within 10s of their presentation; otherwise, the trial was considered an ‘abandoned trial’ and the levers retracted. All experimental data shown was collected while mice performed this final “70–10” stage.

#### Cellular-resolution calcium imaging

To selectively image from neurons which project to the NAc, we utilized a combinatorial virus strategy to image cortical and thalamic neurons which send projections to the NAc. 16 mice (7 PL-NAc, 9 mTH-NAc) previously trained on the probabilistic reversal learning task were unilaterally injected with 500nL of a retrogradely transporting virus to express Cre-recombinase (CAV2-cre, IGMM vector core, France, injected at ~2.5 × 10^12^ parts/mL or retroAAV-EF1a-Cre-WPRE-hGHpA, PNI vector core, injected at ~6.0 × 10^13^) in either the right or left NAc core (1.2 mm A/P, +/− 1.0 mm M/L, −4.7 D/V) along with 600nL of a virus to express GCaMP6f in a Cre-dependent manner (AAV2/5-CAG-Flex -GCaMP6f-WPRE-SV40, UPenn vector core, injected at ~1.27 × 10^13^ parts/mL) in either the mTH (−0.3 & −0.8 A/P, +/− 0.4 M/L, −3.7 D/V) or PL (1.5 & 2.0 A/P, +/− 0.4 M/L, −2.5 D/V) of the same hemisphere. 154 of 278 (55%, n = 5 mice) PL-NAc neurons and 95 out of 256 (37%, n = 5 mice) mTH-NAc neurons were labeled using the CAV2-Cre virus, the remainder were labeled using the retroAAV-Cre virus. In this same surgery, mice were implanted with a 500 μ*m* diameter gradient refractive index (GRIN) lens (GLP-0561, Inscopix) in the same region as the GCaMP6f injection – either the PL (1.7 A/P, +/− 0.4 M/L, −2.35 D/V) or mTH (−0.5 A/P, +/− 0.3 M/L, −3.6 D/V). 2–3 weeks after this initial surgery, mice were implanted with a base plate attached to a miniature, head-mountable, one-photon microscope (nVISTA HD v2, Inscopix) above the top of the implanted lens at a distance which focused the field of view. All coordinates are relative to bregma using *Paxinos and Franklin’s the Mouse Brain in Stereotaxic Coordinates*, *2nd edition* (Paxinos and Franklin, 2004). GRIN lens location was imaged using the Nanozoomer S60 Digital Slide Scanner (Hamamatsu) (location of implants shown in Figure S1). The subsequent image of the coronal section determined to be the center of the lens implant was then aligned to the Allen Brain Atlas (Allen Institute, brain-map.org) using the *Wholebrain* software package (wholebrainsoftware.org; Fürth et al., 2018).

Post-surgery, mice with visible calcium transients were then retrained on the task while habituating to carrying a dummy microscope attached to the implanted baseplate. After the mice acclimated to the dummy microscope, they performed the task while images of the recording field of view were acquired at 10 Hz using the Mosaic acquisition software (Inscopix). To synchronize imaging data with behavioral events, pulses from the microscope and behavioral acquisition software were recorded using either a data acquisition card (USB-201, Measurement computing) or, when LED tracking (see below for details) was performed, an RZ5D BioAmp processor from Tucker-Davis Technologies. Acquired videos were then pre-processed using the Mosaic software and spatially downsampled by a factor of 4. Subsequent down-sampled videos then went through two rounds of motion-correction. First, rigid motion in the video was corrected using the translational motion correction algorithm based on (Thévenaz et al., 1998) included in the Mosaic software (Inscopix, motion correction parameters: translation only, reference image: the mean image, speed/accuracy balance: 0.1, subtract spatial mean [r = 20 pixels], invert, and apply spatial mean [r = 5 pixels]). The video then went through multiple rounds of non-rigid motion correction using the NormCore motion correction algorithm (Pnevmatikakis and Giovannucci, 2017) NormCore parameters: gSig = 7, gSiz = 17, grid size and grid overlap ranged from 12–36 and 8–16 pixels, respectively, based on the individual motion of each video. Videos underwent multiple (no greater than 3) iterations of NormCore until non-rigid motion was no longer visible). Following motion correction, the CNMFe algorithm (Zhou et al., 2018) was used to extract the fluorescence traces (referred to as ‘GCaMP6f’ throughout the text) as well as an estimated firing rate of each neuron (CNMFe parameters: spatial downsample factor = 1, temporal downsample = 1, Gaussian kernel width = 4, maximum neuron diameter = 20, tau decay = 1, tau rise = 0.1). Only those neurons with an estimated firing rate of four transients/minute or higher were considered ‘task-active’ and included in this paper – 278/330 (84%; each mouse contributed 49,57,67,12,6,27,60 neurons, respectively) of neurons recorded from PL-NAc passed this threshold while 256/328 (78%; each mouse contributed 17,28,20,46,47,40,13,13,32 neurons, respectively) passed in mTH-NAc. Across all figures, to normalize the neural activity across different neurons and between mice, we Z-scored each GCaMP6f recording trace using the mean and standard deviation calculated using the entire recording session.

#### Optogenetic stimulation of PL-NAc neurons

22 male C57BL/6J mice were bilaterally injected in either the PL (n = 14 mice, M–L ± 0.4, A–P 2.0 and D–V −2.5 mm) or mTH (n = 8 mice, M–L ± 0.3, A–P −0.7 and D–V −3.6 mm) with 600nL AAV2/5-CaMKIIa-hChR2-EYFP (UPenn vector core, injected 0.6 μL per hemisphere of titer of 9.6 × 10^13^ pp per ml). Optical fibers (300 μm core diameter, 0.37 NA) delivering 1–3 mW of 447 nm laser light (measured at the fiber tip) were implanted bilaterally above the NAc Core at a 10° angle (M–L ± 1.1, A–P 1.4 and D–V −4.2 mm). An additional cohort of control mice (n = 8) were implanted with optical fibers in the NAc without injection of ChR2 and underwent the same stimulation protocol outlined below (Figures S12E–12H). Mice were anesthetized for implant surgeries with isoflurane (3–4% induction and 1–2% maintenance). Mice were given 5 days of recovery after the surgical procedure before behavioral testing.

During behavioral sessions, 5 ms pulses of 1–3 mW, 447 nm blue light was delivered at 20 Hz on a randomly selected 10% of trials beginning when the mouse entered the central nose poke. Light stimulation on unrewarded trials ended 1s after the end of the CS− presentation. On rewarded trials, light administration ended either 1s after CS + presentation (‘cohort 1’) or at the end of reward consumption, as measured by the mouse not engaging the reward port for 100ms (‘cohort 2’). See Figure S13 for a schematic of stimulation times as well as the behavior of the two cohorts. Mice alternated between sessions with and without stimulation – sessions without stimulation were excluded from analysis. Anatomical targeting was confirmed as successful in all mice through histology after the experiment, and therefore no mice were excluded from this dataset.

To quantify the effect of laser stimulation on latency times shown in Figures S12A–12D, we ran a mixed effects linear model using the *fitglme* package in MATLAB. In this model, the median latency to initiate a trial of a mouse, defined as the time between illumination of the central nose poke (i.e., trial start) and the mouse initiating a trial via nose poke, was predicted using i) opsin identity (PL-NAc CaMKII-ChR2, mTH-NAc CaMKII-ChR2 or no-opsin controls), ii) laser stimulation on the current trial, iii) laser stimulation on the previous trial, iv) the interaction between opsin identity and laser stimulation on the current trial and v) the interaction between opsin and laser stimulation on the previous trial. To account for individual variation between mice, a random effect of mouse ID was included.

### QUANTIFICATION AND STATISTICAL ANALYSIS

#### Logistic choice regression

For the logistic choice regressions shown in Figures 1E and S2A, we modeled the choice of the mouse on trial *i* based on lever choice and reward outcome information from the previous n trials using the following logistic regression model:

(Equation 2) $$\text{log}\left(\frac{C(i)}{1-C(i)}\right)=\beta_{0}+\overset{n}{\underset{j=1}{\sum }}\beta_{j}^{R}R(i-j)+\overset{n}{\underset{j=1}{\sum }}\beta_{j}^{U}U(i-j)+\text{error}$$

where *C*(*i*) is the probability of choosing the right lever on trial *i*, and *R*(*i-j*) and *U*(*i-j*) are the choice of the mouse *j* trials back from the *i*^th^ trial for either rewarded or unrewarded trials, respectively. *R*(*i-j*) was defined as +1 when the *j*^th^ trial back was both rewarded and a right press, −1 when the *j*^th^ trial back was rewarded and a left press and 0 when it was unrewarded. Similarly, *U*(*i-j*) was defined as +1 when the *j*^th^ trial back was both unrewarded and a right press, −1 when the *j*^th^ trial back was unrewarded and a left press and 0 when it was rewarded. The calculated regression coefficients, $\beta_{j}^{R}$ and $\beta_{j}^{U}$, reflect the strength of the relationship between the identity of the chosen lever on a previously rewarded or unrewarded trial, respectively, and the lever chosen on the current trial.

To examine the effect of optogenetic stimulation from multiple trials back on the mouse’s choice (Figure 7C, 7E, 7H, 7K; S12H and S13C and S13D), we expanded our behavioral logistic regression model to include the identity of those trials with optical stimulation, as well as the interaction between rewarded and unrewarded choice predictors and stimulation:

(Equation 3) $$\text{log}\left(\frac{C(i)}{1-C(i)}\right)=\beta_{0}+\overset{n}{\underset{j=1}{\sum }}\beta_{j}^{R}R(i-j)+\overset{n}{\underset{j=1}{\sum }}\beta_{j}^{U}U(i-j)+\ldots \;\overset{n}{\underset{j=1}{\sum }}\beta_{j}^{LR}L(i-j)R(i-j)+\overset{n}{\underset{j=1}{\sum }}\beta_{j}^{LU}L(i-j)U(i-j)+\overset{n}{\underset{j=1}{\sum }}\beta_{j}^{L}L(i-j)+\text{error}$$

where *L*(*i*) represents optical stimulation on the *i*^th^ trial (1 for optical stimulation, 0 for control trials), $\beta_{j}^{L}$ represents the coefficient corresponding to the effect of stimulation on choice *j* trials back, and $\beta_{j}^{LR}$ and $\beta_{j}^{LU}$ represent the coefficients corresponding to the interaction between rewarded choice × optical stimulation and unrewarded choice × stimulation, respectively.

To visualize the relative influence of stimulation on the mice’s choices compared with unstimulated trials, in Figures 7C, 7E, 7H, 7K, S12H, S13C, and S13D, the solid blue trace represents the sum of the rewarded choice coefficients (represented by the black trace) and rewarded choice × stimulation coefficients ($\beta_{j}^{R}+\beta_{j}^{LR}$). Similarly, the dashed blue trace represents the sum of the unrewarded choice coefficients (gray trace) and unrewarded choice × stimulation coefficients ($\beta_{j}^{U}+\beta_{j}^{LU}$). For all choice regressions, the coefficients for each mouse were fit using the *glmfit* function in MATLAB and error bars represent mean ± SEM across mice.

#### Encoding model to generate response kernels for behavioral events

To determine the response of each neuron attributable to each of the events in our task, we used a multiple linear encoding model with lasso regularization to generate a response kernel for each behavioral event (example kernels shown in Figure 2B). In this model, the dependent variable was the GCaMP6f trace of each neuron recorded during a behavioral session and the independent variables were the times of each behavioral event (‘nose poke’, ‘levers out’, ‘ipsilateral lever press’, ‘contralateral lever press’, ‘CS+’, ‘CS−’ and ‘reward consumption) convolved with a 25 degrees of freedom spline basis set that spanned −2 to 6s before and after the time of action events (‘nose poke’, ‘ipsilateral press’, ‘contralateral press’ and ‘reward consumption’) and 0 to 8s from stimulus events (‘levers out’, ‘CS+’ and ‘CS−’). To generate this kernel, we used the following linear regression with lasso regularization using the *lasso* function in MATLAB:

(Equation 4) $$\underset{\beta_{0},\beta_{jk}}{\text{min}}\left(\overset{T}{\underset{t=1}{\sum }}{\left(F(t)-\overset{K}{\underset{k=1}{\sum }}\overset{N_{sp}}{\underset{j=1}{\sum }}\beta_{jk}X_{jk}(t)-\beta_{0}\right)}^{2}+\lambda \overset{K}{\underset{k=1}{\sum }}\overset{N_{sp}}{\underset{j=1}{\sum }}\left|\beta_{jk}\right|\right)$$

where *F*(*t*) is the Z-scored GCaMP6f fluorescence of a given neuron at time *t*, *T* is the total time of recording, *K* is the total number of behavioral events used in the model, *N*_sp_ is the degrees of freedom for the spline basis set (25 in all cases, splines generated using the FDAfuns MATLAB package), *β*_jk_ is the regression coefficient for the *j*^th^ spline basis function and *k*^*th*^ behavioral event, *β*_*0*_ is the intercept term and *λ* is the lasso penalty coefficient. The value of lambda was chosen for each neuron that minimized the mean squared error of the model, as determined by 5-fold cross validation. The predictors in our model, *X*_jk_, were generated by convolving the behavioral events with a spline basis set, to enable temporally delayed versions of the events to predict neural activity:

(Equation 5) $$X_{jk}(t)=\overset{N=81}{\underset{i=1}{\sum }}S_{j}(i)e_{k}(t-i)$$

where *S*_j_(i) is the *j*^th^ spline basis function at time point *i* with a length of 81 time bins (time window of −2 to 6s for action events or 0 to 8s for stimulus events sampled at 10 Hz) and *e*_k_ is a binary vector of length *T* representing the time of each behavioral event *k* (1 at each time point where a behavioral event was recorded using the MED associates and TDT software, 0 at all other timepoints).

Using the regression coefficients, *β*_jk_, generated from the above model, we then calculated a ‘response kernel’ for each behavioral event:

(Equation 6) $${\text{kernel}}_{k}(t)=\overset{N_{sp}}{\underset{j=1}{\sum }}\beta_{jk}S_{j}(t)$$

This kernel represents the (linear) response of a neuron to each behavioral event, while accounting for the linear component of the response of this neuron to the other events in the task.

#### Quantification of neural modulation to behavioral events

To identify neurons that were significantly modulated by each of the behavioral events in our task (fractions shown in Figures 2G and 2H), we used the encoding model described above, but without the lasso regularization:

(Equation 7) $$F(t)=\beta_{0}+\overset{K}{\underset{k=1}{\sum }}\overset{N_{sp}}{\underset{j=1}{\sum }}\beta_{jk}X_{jk}(t)$$

As above, *F*(*t*) is the Z-scored GCaMP6f fluorescence of a given neuron at time *t*, *K* is the total number of behavioral events used in the model, *N*_sp_ is the degrees of freedom for the spline basis set (25 in all cases), *β*_*jk*_ is the regression coefficient for the *j*^th^ spline basis function and *k*^th^ behavioral event and *β*_*0*_ is the intercept term. To determine the relative contribution of each behavioral event when predicting the activity of a neuron, we compared the full version of this model to a reduced model with the *X* and *β* terms associated with the behavioral event in question excluded. For each behavioral event, we first generated an F-statistic by comparing the fit of a full model containing all event predictors with that of a reduced model that lacks the predictors associated with the event in question. We then calculated this same statistic on 500 instances of shuffled data, where shuffling was performed by circularly shifting the GCaMP6f fluorescence by a random integer. We then compared the F-statistic from the real data to the shuffled distribution to determine whether the removal of an event as a predictor compromised the model significantly more than expected by chance. If the resulting p-value was less than the significance threshold of p = 0.01, after accounting for multiple comparison testing of each of the behavioral events by Bonferroni correction, then the event was considered significantly encoded by that neuron.

To determine whether a neuron was significantly selective to the choice or outcome of a trial (‘choice-selective’ and ‘outcome-selective’, fractions of neurons from each population shown in Figures 3A and 3C), we utilized a nested model comparison test similar to that used to determine significant modulation of behavioral events above, where the full model used the following behavioral events as predictors: ‘nose poke’, ‘levers out’, ‘all lever press’, ‘ipsilateral lever press’, ‘all CS’, ‘CS+’ and ‘reward consumption’. For choice-selectivity, an F-statistic was computed for a reduced model lacking the ‘ipsilateral lever press’ predictors and significance was determined by comparing this value with a null distribution generated using shuffled data as described above. For outcome-selectivity, the reduced model used to test for significance lacked the predictors associated with both the ‘CS+’ and ‘reward consumption’ events.

By separating the lever press and outcome-related events into predictors that were either blind to the choice or outcome of the trial (‘all lever press’ and ‘all CS’, respectively) and those which included choice or outcome information (‘ipsilateral lever press’ or ‘CS+’ and ‘reward consumption’, respectively) we were able to determine whether the model was significantly impacted by the removal of either choice or outcome information. Therefore, neurons with significant encoding of the ‘ipsilateral lever press’ event (using the same p-value threshold determined by the shuffled distribution of F-statistics) were considered choice-selective, while those with significant encoding of the ‘CS+/reward consumption’ events were considered outcome-selective.

#### Neural decoders

##### Choice decoder

In Figure 3B, we quantified how well simultaneously imaged populations of 1–10 PL-NAc or mTH-NAc neurons could be used to decode choice using a logistic regression:

(Equation 8) $$\text{log}\left(\frac{C(i)}{1-C(i)}\right)=\beta_{0}+\overset{n}{\underset{j=1}{\sum }}\beta_{j}X_{j}(i)+\varepsilon$$

where *C*(*i*) is the probability the mouse made an ipsilateral choice on trial *i*, *β*_0_ is the offset term, *n* is the number of neurons (between 1 and 10), *β*_j_ is the regression weight for each neuron, *X*_j_(*i*) is the mean z-scored GCaMP6f fluorescence from −2s to 6s around the lever press on trial *i* and ε is the error term.

Given that the mice’s choices were correlated across neighboring trials, we weighted the logistic regression based on the frequency of each trial type combination. This was to ensure that choice decoding of a given trial was a reflection of the identity of the lever press on the current trial as opposed to that of the previous or future trial. Thus, we classified each trial as one of eight ‘press sequence types’ based on the following ‘previous-current-future’ press sequences: ipsi-ipsi-ipsi, ipsi-ipsi-contra, ipsi-contra-contra, ipsi-contra-ipsi, contra-contra-contra, contra-contra-ipsi, contra-ipsi-ipsi, contra-ipsi-contra. We then used this classification to equalize the effects of press-sequence type on our decoder by generating weights corresponding to the inverse of the frequency of the press sequence type of that trial. These weights were then used as an input to the *fitglm* function in MATLAB, which was used to fit a weighted version of the logistic regression model above (Equation 8).

Decoder performance was evaluated with 5-fold cross-validation by calculating the proportion of correctly classified held-out trials. Predicted ipsilateral press probabilities greater than or equal to 0.5 were decoded as an ipsilateral choice and values less than 0.5 were decoded as a contralateral choice. This was repeated with 100 combinations of randomly selected, simultaneously imaged neurons from each mouse. Reported decoding accuracy is the average accuracy across the 100 runs and 5 combinations of train-test data for each mouse. Note that only 6/7 mice in the PL-NAc cohort were used in the decoder analyses as one mouse had fewer than 10 simultaneously imaged neurons.

##### Outcome decoder

For the outcome decoder in Figure 3D, we used the same weighted logistic regression used for choice decoding, except the dependent variable was the outcome of the trial (+1 for a reward, 0 for no reward) and the predictors were the average GCaMP6f fluorescence during the intertrial interval (ITI) of each trial. The ITI was defined as the time between CS presentation and either 1s before the next trial’s nose poke or 8s after the CS, whichever occurred first. This was used in order to avoid including any neural activity attributable to the next trial’s nose poke in our analysis.

To correct for outcome correlations between neighboring trials, we performed a similar weighting of predictors as performed in the choice decoder above using the following eight outcome sequence types: ‘reward-reward- reward’, ‘reward-reward- unreward’, ‘reward-unreward- unreward’, ‘reward-unreward- reward’, ‘unreward-unreward- unreward’, ‘unreward-unreward- reward’, ‘unreward-reward- reward’, ‘unreward-reward- unreward.’

##### Time course choice decoder

To determine how well activity from PL-NAc and mTH-NAc neurons was able to predict the mouse’s choice as a function of time throughout the trial (Figures 4E, S7E, and S8E), we trained separate logistic regressions on 500ms bins throughout the trial, using the GCaMP6f fluorescence of 10 simultaneously imaged neurons.

Because of the variability in task timing imposed by the jitter and variability of the mice’s actions, we linearly interpolated the GCaMP6f fluorescence trace of each trial to a uniform length, *t*_adjusted_, relative to behavioral events in our task. Specifically, for each trial, *T*, we divided time into the following four epochs: (i) 2s before nose poke, (ii) time from the nose poke to the lever press, (iii) time from the lever press to the nose poke of the subsequent trial, *T*+1 and (iv) the 3s following the next trial nosepoke. For epochs *ii* and *iii*, *t*_adjusted_ was determined by interpolating the GCaMP6f fluorescence trace from each trial to a uniform length defined as the median time between the flanking events across all trials. Thus, *t*_adjusted_ within each epoch for each trial, *T*, was defined as:

(Equation 9) $$T_{\text{adjusted}}(t)\equiv \{\begin{array}{ll} t, & \\ 2+\frac{t-t_{np}^{T}}{t_{lp}^{T}-t_{np}^{T}}\tilde{ep_{ii}}, & \begin{array}{l} t_{np}^{T}-2\leq t<t_{np}^{T}\\ t_{np}^{T}\leq t<t_{lp}^{T} \end{array}\\ 2+\tilde{ep_{ii}}+\frac{t-t_{np}^{T}}{t_{np}^{T+1}-t_{lp}^{T}}\tilde{ep_{iii}}, & \begin{array}{l} t_{lp}^{T}\leq t<t_{np}^{T+1}\\ t_{np}^{T+1}\leq t<t_{np}^{T+1}+3 \end{array}\\ t, & \; \end{array}$$

where $t_{np}^{T}$, and $t_{lp}^{T}$ are the times of the nose poke and lever press on the current trial, $t_{np}^{T+1}$ is the time of the nose poke of the subsequent trial $\tilde{ep_{ii}}$, and $\tilde{ep_{iii}}$ are the median times across trials of epoch *ii* and *iii*.

The resulting time-adjusted GCaMP6f traces were divided into 500ms bins. For each bin, we fit the weighted logistic regression described above to predict choice on the current, previous or future trial from the activity of 10 simultaneously imaged neurons. Predictors were weighted based on press sequence type as described above. Decoding accuracy was assessed as described above using 100 combinations of 10 randomly selected neurons and 5-fold cross-validation. To determine if decoding was significantly above chance, which is 0.5, for each time point we performed a two-tailed, one-sample t test.

#### Statistics

All t-tests reported in the results and as specified in each figure legend were performed using either the *ttest* or *ttest2* function in MATLAB. In all cases, t-tests were two-tailed. In cases where multiple comparisons were performed, we applied a Bonferroni correction to determine the significance threshold. Two-proportion Z-tests (used to compare fractions of significantly modulated/selective neurons, Figures 2H, 3A and 3C) and Fisher’s Z (used to compare correlation coefficients, Figures 4D and S7D) were performed using Vassarstats.net. Asterisks indicating significance thresholds are referenced in respective figure legends.

For all t-tests in this paper, data distributions were assumed to be normal, but this was not formally tested. No statistical methods were used to predetermine sample sizes, but our sample sizes were similar to those generally employed in the field.

#### Synaptic plasticity model

To computationally model how the brain could solve the reversal learning task using fast dopamine-mediated synaptic plasticity, we generated a biological instantiation of the TD algorithm for reinforcement learning (Sutton and Barto, 1998) by combining the recorded PL-NAc activity with known circuit connectivity in the NAc and associated regions (Hunnicutt et al., 2016; Kalivas et al., 1993; Otis et al., 2017; Watabe-Uchida et al., 2012). The goal of the model is to solve the “temporal credit assignment problem” by learning the value of each choice at the onset of the choice-selective PL-NAc sequence, when we assume the mouse makes its decision and which is well before the time of reward.

#### Synaptic plasticity model description

##### The value function

Our implementation of the TD algorithm seeks to learn an estimate, at any given time, of the total discounted sum of expected future rewards, known as the value function *V*(*t*). To do this, we assume that the value function over time is decomposed into a weighted sum of temporal basis functions $f_{i}^{R}(t)$ and $f_{i}^{L}(t)$ (Sutton and Barto, 1998) corresponding to the right-choice and left-choice preferring neurons:

(Equation 10) $$V_{R}(t)=\overset{n_{R}}{\underset{i=1}{\sum }}w_{i}^{R}(t)f_{i}^{R}(t)\;V_{L}(t)=\overset{n_{L}}{\underset{i=1}{\sum }}w_{i}^{L}(t)f_{i}^{L}(t)$$

with the total value being given by the sum over both the left and right neurons as

(Equation 11) $$V(t)=V_{R}(t)+V_{L}(t)$$

Here, *V*_R_(t) and *V*_L_(t) are the components of the value functions encoded by the right- and left-preferring neurons respectively, *n*_R_ and *n*_L_ are the number of right- and left-preferring choice-selective neurons respectively, and $w_{i}^{R,L}$ are the weights between the *i*^th^ PL neuron and the NAc, which multiply the corresponding basis functions. Thus, each term in *V*_R_(t) or *V*_L_(t) above corresponds to the activity of one of the striatal neurons in the model (Figure 5A). Note that, in our model, the total value *V*(t) sums the values associated with the left and right actions and is thus not associated with a particular action. At any given time on a given trial, the choice-selective activity inherent to the recorded PL-NAc neurons results predominantly in activation of the sequence corresponding to the chosen lever compared to the unchosen lever (see Figure 5C), so that a single sequence, corresponding to the chosen action, gets reinforced.

##### The reward prediction error (RPE)

TD learning updates the value function iteratively by computing errors in the predicted value function and using these to update the weights *w*_i_. The RPE at each moment of time is calculated from the change in the estimated value function over a time step of size *dt* as follows

(Equation 12) $$RPE=\delta (t)dt=r(t)dt+e^{\frac{-dt}{\tau }}V(t)-V(t-dt)$$

where *δ*(*t*) is the reward prediction error per unit time. Here, the first two terms represent the estimated value at time *t*, which equals the sum of the total reward received at time *t* and the (discounted) expectation of rewards, i.e., value at all times into the future. This is compared to the previous time step’s estimated value *V*(*t-dt*). The coefficient $e^{\frac{-dt}{\tau }}$ represents the temporal discounting of rewards incurred over the time step *dt*. Here *τ* denotes the timescale of temporal discounting and was chosen to be 0.7s.

To translate this continuous time representation of RPE signals to our biological circuit model, we assume that the RPE *δ*(*t*) is carried by dopamine neurons (Montague et al., 1996; Schultz et al., 1997). These dopamine neurons receive three inputs corresponding to the three terms on the right side of the above equation: a reward signal originating from outside the VTA, a discounted estimate of the value function *V*(*t*) that, in Figure 5A, represents input from the striatum via the ventral pallidum (Chen et al., 2019; Tian et al., 2016) and an oppositely signed, delayed copy of the value function *V*(*t*-Δ) that converges upon the VTA interneurons (Cohen et al., 2012).

Because the analytical formulation of TD learning in continuous time is defined in terms of the infinitesimal time step *dt*, but a realistic circuit implementation needs to be characterized by a finite delay time for the disynaptic pathway through the VTA interneurons, we rewrite the above equation approximately for small, but finite delay Δ as:

(Equation 13) $$\delta (t)dt=r(t)dt+\frac{\gamma V(t)-V(t-\Delta )}{\Delta }dt$$

where we have defined $\gamma =e^{-\frac{\Delta }{\tau }}$ as the discount factor corresponding to one interneuron time delay and, in all simulations, we chose a delay time Δ = 0.01s. Note that the discount factor is biologically implemented in different strengths of the weights of the VP inputs to the GABA interneuron and dopaminergic neuron in the VTA.

The proposed circuit architecture of Figure 5A can be rearranged into several other, mathematically equivalent architectures (Figure S9). These architectures are not mutually exclusive, so other more complicated architectures could be generated by superpositions of these architectures.

##### The eligibility trace

The RPE at each time step *δ*(*t*) was used to update the weights of the recently activated synapses, where the “eligibility” *E*_i_(*t*) of a synapse for updating depends upon an exponentially weighted average of its recent past activity (Gerstner et al., 2018; Sutton and Barto, 1998):

(Equation 14) $$E_{i}(t)=\overset{t}{\underset{-\infty }{\int }}e^{\frac{s-t}{\tau_{e}}}f_{i}(s)ds$$

which can be rewritten as

(Equation 15) $$\frac{dE_{i}(t)}{dt}=-\frac{E_{i}(t)}{\tau_{e}}+f_{i}(t)$$

or, in the limit dt<<1,

(Equation 16) $$E_{i}(t)\approx e^{-\frac{dt}{\tau_{e}}}E_{i}(t-dt)+f_{i}(t)dt$$

where *τ*_e_ defines the time constant of the decay of the eligibility trace, which was chosen to be 0.8s consistent with (Gerstner et al., 2018; Yagishita et al., 2014).

##### Weight updates

The weight of each PL-NAc synapse, *w*_i_, is updated according to the product of its eligibility *E*_i_(*t*) and the RPE rate *δ*(*t*) at that time using the following update rule (Gerstner et al., 2018; Sutton and Barto, 1998):

(Equation 17) $$\frac{d{\hat{W}}_{i}(t)}{dt}=\alpha \delta (t)E_{i}(t)\;w_{i}(t)=\text{max}\left[0,{\hat{w}}_{i}(t)\right]$$

where *α* = 0.009(spikes/s)^−1^ was the learning rate. Note that the units of *α* derive from the units of weight being *value*(spikes/s)^−1^. The PL-NAc weights used in the model are thresholded to be non-negative so that the weights obey Dale’s principle.

##### Action selection

In the model, the decision to go left or right is determined by “probing” the relative values of the left versus right actions just prior to the start of the choice-selective sequence. To implement this, we assumed that the choice was readout in a noisy, probabilistic manner from the activity of the cluster of neurons that responded at the time choice-selectivity robustly appeared, when we assume the decision is made. This corresponded to the first 17 neurons in each (left or right) PL population prior to the start of the sequential activity. This was accomplished by providing a 50 ms long, noisy probe input to each of these PL neurons and reading out the summed activity of the left and the summed activity of the right striatal populations. The difference between these summed activities was then put through a softmax function (given below) to produce the probabilistic decision.

To describe this decision process quantitatively, we define the probability of making a leftward or rightward choice in terms of underlying decision variables *d*_left_ and *d*_right_ corresponding to the summed activity of the first 17 striatal neurons in each population:

(Equation 18) $$d_{\text{left}}=E_{t}\left[\overset{17}{\underset{i=1}{\sum }}w_{i}^{\text{left}}n_{i}^{\text{left}}(t)\right]\;d_{\text{right}}=E_{t}\left[\overset{17}{\underset{i=1}{\sum }}w_{i}^{\text{right}}n_{i}^{\text{right}}(t)\right]$$

where $E_{\text{t}}[.]$ denotes time-averaging over the 50 ms probe period and $n_{i}^{\text{left}}(t)$ and $n_{i}^{\text{right}}(t)$ denote the non-negative stochastic probe input, was chosen independently for each neuron and each time step from a normal distribution (truncated at zero to enforce non-negativity) with mean prior to truncation equal to 0.05s^−1^ (5% of peak activity) and a standard deviation of $0.0025/\sqrt{dt}s^{-1}$. Note that the weights $w_{i}^{\text{left}/\text{right}}$ used here correspond to the weights from the end of the previous trial, which we assume are the same as the weights at the beginning of the next trial. The probability of choosing the left or the right lever for a given trial *n* is modeled as a softmax function of these decision variables plus a “stay with the previous choice” term that models the tendency of mice in our study to return to the previously chosen lever irrespective of reward (Figure 1D), given by the softmax distribution

(Equation 19) $$\text{Pr}ob(\text{left})=\frac{\text{exp}\left(\beta_{\text{value}}d_{\text{left}}+\beta_{\text{stay}}I_{\text{left}}\right)}{\text{exp}\left(\beta_{\text{value }}d_{\text{left}}+\beta_{\text{stay}}I_{\text{left}}\right)+\text{exp}\left(\beta_{\text{value}}d_{\text{right}}+\beta_{\text{stay}}I_{\text{right}}\right)}\;\mathrm{Pr}ob(\text{right})=\frac{\text{exp}\left(\beta_{\text{value}}d_{\text{right}}+\beta_{\text{stay}}I_{\text{right}}\right)}{\text{exp}\left(\beta_{\text{value }}d_{\text{left}}+\beta_{\text{stay}}I_{\text{left}}\right)+\text{exp}\left(\beta_{\text{value}}d_{\text{right}}+\beta_{\text{stay}}I_{\text{right}}\right)}$$

where *I*_*left*/*right*_ is 1 if that action (i.e., left or right) was chosen on the previous and 0 otherwise, and *β*_*value*_ = 7000 and *β*_*stay*_ = 0:15 are free parameters that define the width of the softmax distribution and the relative weighting of the value-driven versus stay contributions to the choice.

#### Synaptic plasticity model implementation

##### Block structure for the model

Block reversals were determined using the same criteria as in the probabilistic reversal learning task performed by the mice – the identity of the rewarded lever reversed after 10 rewarded trials plus a random number of trials drawn from the geometric distribution given by Equation 1. The model used p = 0.4 as in the reversal learning experiments. Given the variation in performance across the models that use PL-NAc, mTH-NAc or early-only activity as input (see Figure 5), the average block length for each model varied as well (because block reversals depended upon the number of rewarded trials). The average block length for the single-trial PL-NAc model, single-trial mTH-NAc model and early-only control were 23.0 ± 7.6, 28.1 ± 8.8 and 25.1 ± 6.3 trials (mean +/− std. dev.), respectively. The PL-NAc model produced a similar block length as that of behaving mice (23.2 ± 7.9 trials, mean +/− std. dev.). Because a block reversal in our task is dependent on the mice receiving a set number of rewards, the choices just prior to a block reversal are more likely to align with the identity of the block and result in reward (see Figure 5E, 5J, and 5O). Thus, the increase in choice probability observed on trials close to the block reversal is an artifact of this reversal rule and not reflective of the model learning choice values.

##### PL-NAc inputs to the neural circuit model

To generate the temporal basis functions *f*_i_(*t*) (example activity shown in Figure 5C), we used the choice-selective sequential activity recorded from the PL-NAc neurons shown in Figures 4B and 4C. Spiking activity was inferred from calcium fluorescence using the CNMFe algorithm (Zhou et al., 2018) and choice-selectivity was determined using the nested comparison model used to generate Figure 3A (see “Quantification of neural modulation to behavioral events” above for details). Model firing rates were generated by Z-scoring the inferred spiking activity of each choice-selective PL-NAc neuron. The resulting model firing rates were interpolated using the *interp* function from Python’s numpy package to match the time step, *dt* = 0.01s, and smoothed using a Gaussian kernel with zero mean and a standard deviation of 0.2s using the *Gaussian_filter1d* function from the ndimage module in Python’s SciPy package.

To generate a large population of model input neurons on each trial, we created a population of 368 choice-selective “pseudoneurons” on each trial. This was done as follows: for each simulated trial, we created 4 copies (pseudoneurons) of each of the 92 recorded choice-selective PL-NAc neurons using that neuron’s inferred spiking activity from 4 different randomly selected trials. The pool of experimentally recorded trials from which pseudoneuron activities were chosen was balanced to have equal numbers of stay and switch trials. This was done because the choices of the mice were strongly positively correlated from trial to trial (i.e., had more stay than switch trials), which (if left uncorrected) potentially could lead to biases in model performance if activity late in a trial was reflective of choice on the next, rather than the present trial. To avoid choice bias in the model, we combined the activity of left- and right-choice-preferring recorded neurons when creating the pool of pseudoneurons. We then randomly selected 184 left-choice-preferring and 184 right-choice-preferring model neurons from this pool of pseudoneurons. An identical procedure, using the 92 most choice-selective mTH-NAc neurons, was followed to create the model mTH-NAc neurons. The identity of these 92 neurons was determined by ranking each neuron’s choice-selectivity using the p value calculated to determine choice-selectivity (see “Quantification of neural modulation to behavioral events” above for details).

To generate the early-only control activity (example activity shown in Figure 5M), similar to the PL-NAc activity, we created a population of 368 pseudoneurons on each trial that were divided into 184 left-choice-preferring and 184 right-choice-preferring pseudoneurons. However, in this case, we only used the early-firing neurons (neurons active at the onset of the sequence) of the PL-NAc population to create the pseudoneurons. Thus, for this control simulation, all neurons contribute to the decision as they are all active at the onset of the sequence when the model makes its choice. More specifically, to create a pool of pseudoneurons, we created multiple copies of each of the first 17 neurons of the left-choice-preferring and right-choice-preferring PL-NAc population, where each copy corresponds to the activity of the neuron on a different randomly selected trial. We then randomly selected 184 left-choice-preferring and 184 right-choice-preferring model neurons from this pool of pseudoneurons. We used a smaller learning rate *α* = 0:003 (*spikes*/_*s*_)^−1^ in this case in order to prevent the PL-NAc synaptic weights from exhibiting unstable growth. We also adjust *β*_*value*_ = 1000 in order to match the stay probability following rewarded trials to that of the model with recorded PL-NAc input (Figure 5P).

To mimic the PL-NAc activity during the optogenetic stimulation of PL-NAc neurons (Figures 7B and 7C), we set $f_{i}^{R,L}(t)$ equal to 0.2 for a randomly selected 70% of PL neurons, at all times *t*, from the time of the simulated nosepoke to 2s after the reward presentation. These ‘stimulation trials’ occurred on a random 10% of trials. 70% of PL neurons were activated to mimic the incomplete penetrance of ChR2 viral expression.

###### Reward input to the neural circuit model.

The reward input *r*(*t*) to the dopamine neurons was modeled by a truncated Gaussian temporal profile centered at the time of the peak reward:

(Equation 20) $$r(t)=R(i)\frac{1}{\sqrt{2\pi \sigma_{r}^{2}}}e^{-\frac{{\left(t-\mu r\right)}^{2}}{2\sigma_{r}^{2}}}$$

where *R*(*i*) is 1 if trial *i* was rewarded and 0 otherwise, *μ*_r_ is the time of peak reward and *σ*_r_ defines the width of the Gaussian (0.3s in all cases, width chosen to approximate distribution of dopamine activity in response to reward stimuli observed in previous studies such as Matsumoto and Hikosaka, 2009 and Schultz et al., 1997). For each trial, a value of *μ*_r_ was randomly drawn from a uniform distribution spanning 0.2–1.2s from the time of the lever press. This distribution was chosen to reflect the 1s jitter between lever press and reward used in our behavioral task (see Methods above) as well as the observed delay between reward presentation and peak dopamine release in a variety of studies (Cohen et al., 2012; Matsumoto and Hikosaka, 2009; Parker et al., 2016; Saunders et al., 2018). To ensure that no residual reward response occurred before the time of the lever press, *r*(*t*) was set to 0 for any time *t* that was 0.2s before the time of the peak reward, *μ*_r_.

##### Initial weights

The performance of the model does not depend on the choice of the initial weights as the model learns the correct weights by the end of the first block irrespective of the chosen initial weights. We chose the initial weights to be zero.

##### Weight and eligibility update implementation

We assumed that the weight and eligibility trace updates start at the time of the simulated nose poke. The nose poke time, relative to the time of the lever press, varies due to a variable delay between the nose poke and the lever presentation as well as variation in time between lever presentation and lever press. To account for this, the weight and eligibility trace updates are initiated at time *t* = *t*_start_, where *t*_start_ was drawn from a Gaussian distribution with a mean at −2.5s, and a variance of 0.2s, which was approximately both the time of the nose poke and the time at which choice-selective sequences initiated in the experimental recordings. The eligibility trace is reset to zero at the beginning of each trial. We stopped updating the weights at the end of the trial, defined as 3s after the time of lever press. The eligibility traces were updated according to Equation 16. The weights were updated by integrating Equation 17 with a first-order forward Euler routine. In all simulations, we used a simulation time step *dt* = 0.01*s*.

#### Neural dynamics model

To computationally model how the brain could solve the reversal learning task without fast dopamine-mediated synaptic plasticity, we used an actor-critic network based on the meta-RL framework introduced by Wang et al. (2018). The model actor and critic networks are recurrent neural networks of Long Short-Term Memory (LSTM) units whose weights are learned slowly during the training phase of the task. The weights are then frozen during the testing phase so that fast reversal learning occurs only through the activation dynamics of the recurrent actor-critic network. Like the synaptic plasticity model, we input recorded PL-NAc activity to a value-generating “critic” network (conceived of as NAc, VP, and associated cortical regions) to generate appropriate reward prediction error signals in dopamine neurons. Unlike the synaptic plasticity model, the reward prediction error signals in this model are sent to an explicit actor network (conceived of as DMS and associated cortical regions), where they act as an input to help generate appropriate action signals based on reward history.

#### Neural dynamics model description

##### LSTM

The model comprises two separate fully connected, gated recurrent neural networks of LSTM units, one each for the actor and critic network. An LSTM unit works by keeping track of a “long-term memory” state (“memory state” ***c***(*t*), also known as cell state) and a “short-term memory” state (“output state” ***h***(*t*), also known as hidden state) at all times. To regulate the information to be kept or discarded in the memory and output states, LSTMs use three types of gates: the input gate ***i***(*t*) regulates what information is input to the network, the forget gate ***φ***(*t*) regulates what information to forget from the previous memory state, and the output gate ***o***(*t*) (not to be confused with the output state ***h***(*t*) regulates the output of the network. More precisely, the dynamics of an LSTM is defined by the following equations:

(Equation 21) $$\varphi (t)=\sigma \left(W_{\varphi }x(t)+U_{\varphi }h(t-\Delta t)+b_{\varphi }\right)\;i(t)=\sigma \left(W_{i}x(t)+U_{i}h(t-\Delta t)+b_{i}\right)\;o(t)=\sigma \left(W_{o}x(t)+U_{o}h(t-\Delta t)+b_{o}\right)\;c(t)=\varphi (t)⊙c(t-\Delta t)+i(t)⊙\text{tanh}\left(W_{c}x(t)+U_{c}h(t-\Delta t)+b_{c}\right)\;h(t)=o(t)⊙\text{tanh}(c(t))$$

where ***x***(*t*) is the vector of external inputs to the LSTM network at time step *t*, ***W***_q_ and ***U***_q_ are the weight matrices of the input and recurrent connections, respectively, where the subscript *q* denotes the state or gate being updated, ***b***_q_ are the bias vectors, ⊙ denotes element-wise multiplication and *σ* denotes the softmax function.

##### Critic network

As in the synaptic plasticity model, the goal of the critic is to learn the value (discounted sum of future rewards) of a given choice at any time in a trial. The learned value signal can then be used to generate the RPE signals that are sent to the actor. The critic is modeled as a network of LSTM units that linearly project through trainable weights to a value readout neuron that represents the estimated value *V*(*t*) at time step *t*. The critic takes as input the reward received *r*(*t*) and the experimentally recorded PL-NAc choice-selective sequential input *f*_*i*_(*t*). The PL-NAc input provides the critic with a representation of the chosen side on the current trial as well as the time during the trial. This allows the critic to output an appropriately timed value signal (and consequently an appropriately timed RPE signal) corresponding to the chosen side. The reward input acts as a feedback signal to the critic that provides information about the correctness of the chosen action.

To map the critic to a biological neural circuit, we hypothesize that NAc, together with VP and associated cortical areas, form the critic recurrent neural network (Figure 6A; Atallah et al., 2007; Lau and Glimcher, 2008; O’Doherty et al., 2004; Richard et al., 2016; Tsutsui et al., 2016). The choice-selective sequential input *f*_*i*_(*t*) to the critic is provided by the recorded choice-selective sequential activity in PL-NAc neurons (Figure 6A).

##### The reward prediction error (RPE)

As in the synaptic plasticity model (Figure 5A), the RPE *δ*(*t*) is computed in the VTA DA neurons based on the value signal from the critic network (Figure 6A).


(Equation 22) δ(t)=r(t)+γV(t)−V(t−Δt)

Unlike the synaptic plasticity model, the RPE signal is conveyed by the VTA dopamine neurons to the actor network. Note that the delay of the negative value signal equals one time step Δt = 0.1s in this model, rather than the smaller delay Δ = 0.01s for the synaptic plasticity model. This is because the neural dynamics model used a larger time step for simulations due to limitations in computational power.

##### Actor network

In contrast to the synaptic plasticity model, in which actions were directly readout from the activity of the value neurons early in the trial, we consider an explicit actor network that generates actions. The actor is modeled as a network of LSTM units that compute the policy, i.e., the probability of choosing an action *a*(*t*) at time step *t* given the current state of the network. The policy is represented by three policy readout neurons, corresponding to choosing left, right or ‘do nothing’, whose activities are given by a (trainable) linear readout of the activities of the actor LSTM units. The actor receives three inputs: (i) an efference copy of the action taken at the previous time step *a*(*t*–Δ*t*), (ii) a ‘temporal context’ input x(*t*), encoded as a vector of all 0s except for a value of 1 in the entry corresponding to the current time point *t*, that provides the actor with a representation of the time within the trial, and (iii) the reward prediction error at the current time step *δ*(*t*).

To map the actor to a biological neural circuit, we hypothesize that the DMS and associated cortical areas form the actor recurrent neural network (Figure 6A; Atallah et al., 2007; O’Doherty et al., 2004; Seo et al., 2012; Tai et al., 2012). The temporal sequence input *ξ*(*t*) to the actor is assumed to originate in the hippocampus or other cortical areas (Figure 6A; Hahnloser et al., 2002; Howard and Eichenbaum, 2013; Kozhevnikov and Fee, 2007; Zhou et al., 2020).

##### Training algorithm

To train the recurrent weights of the network, which are then held fixed during task performance, we implement the Advantage Actor-Critic algorithm (Mnih et al., 2016) on a slightly modified version of the reversal learning task (see “Block structure for training” section below). In brief, the weights of the neural network are updated via gradient descent and backpropagation through time. The loss function for the critic network, $L_{\text{critic}}$, defines the error in the estimated value function. The synaptic weight parameters *θ*_v_ of the critic network are updated through gradient descent on the critic loss function $L_{\text{critic}}$:

(Equation 23) $$\Delta \theta_{v}=-\alpha \nabla L_{\text{critic}}\;\nabla L_{\text{critic}}=-\beta_{v}\delta_{t}\left(s_{t};\theta_{v}\right)\frac{\partial V}{\partial \theta_{v}}$$

where *α* is the learning rate, *s*_t_ is the state at time step *t*, *V* denotes the value function and *β*_*v*_ is the scaling factor of the critic loss term. *δ*_t_(*s*_t_*;θ*_*v*_) is the k-step return temporal difference error (not to be confused with the RPE input to the actor defined in Equation 22) defined as follows:

$$\delta_{t}\left(s_{t};\theta_{v}\right)=R_{t}-V\left(s_{t};\theta_{v}\right)$$

where *R*_t_ is the discounted k-step bootstrapped return at time *t*

$$R_{t}=\overset{k-1}{\underset{i=0}{\sum }}\left(r_{t+i}\overset{i}{\underset{j=0}{\prod }}\gamma_{t+j}\right)+V\left(s_{t+k};\theta_{v}\right)\overset{k}{\underset{j=0}{\prod }}\gamma_{t+j}$$

where *r*_t_ is the reward received at time step *t*, *γ*_*t*_ is the discount factor at time step *t* (defined below), and *k* is the number of time steps until the end of an episode.

The loss function for the actor network, $L_{\text{actor}}$, is given by a weighted sum of two terms: a policy gradient loss term, which enables the actor network to learn a policy *π*(*a*_*t*_*|s*_t_) that approximately maximizes the estimated sum of future rewards *V(s*_*t)*_, and an entropy regularization term that maximizes the entropy of the policy *π* to encourage the actor network to explore by avoiding premature convergence to suboptimal policies. The gradient of the actor loss function $L_{\text{actor}}$ with respect to the synaptic weight parameters of the actor network, *θ*, is given by.

(Equation 24) $$\Delta \theta =-\alpha \nabla L_{\text{actor}}\;\nabla L_{\text{actor}}=-\frac{\partial \text{log}\pi \left(a_{t}|s_{t};\theta \right)}{\partial \theta }\delta_{t}\left(s_{t};\theta_{v}\right)-\beta_{e}\frac{\partial H\left(s_{t};\theta \right)}{\partial \theta }$$

where *a*_*t*_ is the action at time step *t*, *π* is the policy, *β*_*e*_ is the scaling factor of the entropy regularization term and *H*(*s*_t_;*θ*) is the entropy of the policy *π*

$$H\left(s_{t};\theta \right)=-\underset{a\in A}{\sum }\pi \left(a|s_{t};\theta \right)\text{log }\pi \left(a|s_{t};\theta \right)$$

where *A* denotes the space of all possible actions.

#### Neural dynamics model implementation

##### LSTM

Both the actor and critic LSTM networks consisted of 128 units each and were implemented using TensorFlow’s Keras API. The weight matrices ***U***_**q**_ were initialized using Keras’s ‘glorot_uniform’ initializer, the weight matrices ***W***_*q*_ were initialized using Keras’s ‘orthogonal’ initializer and the biases ***b***_*q*_ were initialized to 0. The output and memory states for both LSTM networks were initialized to zero at the beginning of each training or testing episode.

##### PL-NAc inputs to the critic

Input to the critic was identical to the smoothed, single-trial input used for the synaptic plasticity model described above, except i) activity was not interpolated because each time step in this model was equivalent to the sampling rate of the collected data (10 Hz), and ii) we chose to input only the activity from 2s before to 2s after the lever press (as compared to 3s after the lever press for the synaptic plasticity model) in order to reduce the computational complexity of the training process. To reduce episode length, and therefore training time, we also excluded those neurons whose peak activity occurred more than 2s after the lever press, reducing the final number of ‘pseudoneurons’ used as input to 306 (compared with 368 for the synaptic plasticity model).

Optogenetic-like stimulation of the PL-NAc population (Figures 7D and 7E) was performed in a similar manner to the synaptic plasticity model, with activity set to 0.15 for a randomly selected 70% of neurons for the duration of the trial.

##### Trial structure

Each trial was 4s long starting at 2s before lever press and ending at 2s after lever press. At any given time, the model has three different choices: choose left, choose right or do nothing. Similar to the synaptic plasticity model, the model makes its decision to choose left or right at the start of a trial, which then leads to the start of the corresponding choice-selective sequential activity. However, unlike the synaptic plasticity model, the model can also choose ‘do nothing’ at the first time step, in which case an activity pattern of all zeros is input to the critic for the rest of the trial. For all other time steps, the correct response for the model is to ‘do nothing’. Choosing ‘do nothing’ on the first time step or choosing something other than ‘do nothing’ on the subsequent time steps results in a reward *r*(*t*) of −1 at that time. If a left or right choice is made on the first time step, then the current trial is rewarded based on the reward probabilities of the current block (Figure 1A) and the reward input *r*(*t*) to the critic is modeled by a truncated Gaussian temporal profile centered at the time of the peak reward (Equation 20) with the same parameters as in the synaptic plasticity model.

##### Block structure for training

We used a slightly modified version of the reversal learning task performed by the mice in which the block reversal probabilities were altered in order to make the block reversals unpredictable. This was done to discourage the model from learning the expected times of block reversals based on the number of rewarded trials in a block and to instead mimic the results of our behavioral regressions (Figure 1E) suggesting that the mice use only the previous ~4 trials to make a choice. To make the block reversals unpredictable, the identity of the high-probability lever reversed after a random number of trials drawn from a geometric distribution (Equation 1) with p = 0.9.

##### Training

Each training episode was chosen to be 15 trials long and the model was trained for 62,000 episodes. For this model, we used a time step Δ*t* = 0.1s. The values of the training hyperparameters were as follows: the scaling factor of the critic loss term *β*_*v*_ = 0.05, the scaling factor of the entropy regularization term *β*_*e*_ = 0.05, the learning rate *α* = 0.01s^−1^(*α* = 0.001 per time step), and the timescale of temporal discounting within a trial *τ* = 2.5s, leading to a discount factor $\gamma =e^{-\frac{\Delta t}{\tau }}=0.96$ for all times except for the last time step of a trial when the discount factor was 0 to denote the end of a trial. The network’s weights and biases were trained using the RMSprop gradient descent optimization algorithm (Hinton et al., 2012) and backpropagation through time, which involved unrolling the LSTM network over an episode (630 time steps).

##### Block structure for testing

Block reversal probabilities for the testing phase were the same as in the probabilistic reversal learning task performed by the mice. The average block length for the PL-NAc neural dynamics model was 19.3 ± 5.0 trials (mean+/−std. dev.).

##### Testing

The model’s performance (Figures 6B–6J) was evaluated in a testing phase during which all network weights were held fixed so that reversal learning was accomplished solely through the neural dynamics of the LSTM networks. The network weights used in the testing phase were the weights learned at the end of the training phase. A testing episode was chosen to be 1500 trials long and the model was run for 120 episodes.

##### Actor network analysis

For Figures 6G–6J, we tested the model’s performance on a slightly modified version of the reversal learning task in which, after training, block lengths were fixed at 30 trials. This facilitated the calculation and interpretation of the block-averaged activity on a given trial of a block. Dimensionality reduction of the actor network activity (Figure 6H) was performed using the *PCA* function from the decomposition module in Python’s scikit-learn package.

##### Replacing sequential input to the critic with persistent input

In Figure 6F, we analyzed how model performance changed when the temporal structure provided by the choice-selective sequential inputs to the critic were replaced during training by persistent choice-selective input. The persistent choice-selective input was generated by setting the activity of all the left-choice selective neurons to 1 and all the right-choice selective neurons to 0 for all time points on left-choice trials and vice versa on right-choice trials.

#### Cross-trial analysis of RPE in dopamine neurons

To generate the regression coefficients in Figures 5G, 5L, 5Q, 6E, S10C, and S10D, we performed a linear regression analysis adapted from (Bayer and Glimcher, 2005), which uses the mouse’s reward outcome history from the current and previous 5 trials to predict the average dopamine response to reward feedback on a given trial, *i*:

(Equation 25) $$D(i)=\beta_{0}+\overset{5}{\underset{j=0}{\sum }}\beta_{j}\hat{R}(i-j)+\text{error}$$

where *D*(*i*) is the average dopamine activity from 0.2 to 1.2s following breward feedback on trial *i*, $\hat{R}(i-j)$ is the reward outcome *j* trials back from trial *i* (1 if *j* trials back is rewarded and 0 if unrewarded) and *β*_*j*_ are the calculated regression coefficients that represent the effect of reward outcome *j* trials back on the strength of the average dopamine activity, *D*(*i*). For the regression coefficients generated from recorded dopamine activity (Figures S10C and S10D) we used the Z-scored GCaMP6f fluorescence from VTA-NAc terminal recordings of 11 mice performing the same probabilistic reversal learning task described in this paper (for details see Parker et al., 2016). The regression coefficients for the experimental data as well as the synaptic plasticity and neural dynamics model simulations were fit using the *LinearRegression* function from the linear_model module in Python’s scikit-learn package.

#### Simulation of model-free versus model-based task performance

##### Overview

In order to identify possible RPE signatures that distinguish ideal observer (“model-based”) versus Q-learning (“model-free”) behavior in this task (Figure S14), we simulated choices using the two models. Based on the dopaminergic signature of block reversal inference reported in (Bromberg-Martin et al., 2010), we first confirmed that our ideal observer and Q-learning models gave rise to distinct dopamine signatures when performing the task used in (Bromberg-Martin et al., 2010). In that task, reward probabilities were 100% and 0% for the “high probability” and “low probability” choices, respectively, and the reward probabilities reversed with a 5% probability on each trial. Next, we applied the same framework to our task, to determine if we could observe similar distinctions between the models. In this case, the reward probabilities were 70% and 10%, as in the task studied in this paper, and blocks reversed with a 5% probability on each trial, which resulted in block lengths comparable to those observed in our experiments.

##### Ideal observer model

The ideal observer model was provided with knowledge of the reward probabilities associated with each block and the probability of block reversal on each trial. The 5% block reversal probability on each trial can be written in terms of the block state transition probabilities as

(Equation 26) $$T_{ij}=P\left(s(t)=s_{j}|s(t-1)=s_{i}\right)=\left[\begin{array}{ll} 0.95 & 0.05\\ 0.05 & 0.95 \end{array}\right]$$

where *T*_*ij*_ is defined as the transition probability between block state *s*_*i*_ on trial *t* and block state *s*_*j*_ on trial *t*+1. Here, ‘block state’ refers to whether the current block has a higher probability of left or right choices being rewarded. The reward probabilities for each block were as follows

(Equation 27) $$R_{ik}=P\left(r(t)=1|s(t)=s_{i},c(t)=c_{k}\right)==\{\begin{array}{ll} \left[\begin{array}{ll} 1.0 & 0.0\\ 0.0 & 1.0 \end{array}\right], & \text{Bromberg }-\text{ Martin Task}\\ \left[\begin{array}{ll} 0.7 & 0.1\\ 0.1 & 0.7 \end{array}\right], & \text{Our Task} \end{array}$$

where *R*_*ik*_ is defined as the probability of reward for block state *s*_*i*_ and choice *c*_*k*_.

On each trial, the ideal observer model selects the choice with the highest expectation of reward based on its belief about the current block state given the choice and reward history. The expectation of reward ρ_*l*_(*t*+1) for choice *l* on trial *t* + 1, given the entire reward history *r*(1:*t*) and choice history *c*(1:*t*) up until trial *t* is given by

(Equation 28) $$\rho_{l}(t+1)=\overset{2}{\underset{i=1}{\sum }}R_{il}P\left(s(t+1)=s_{i}|r(1:t),c(1:t)\right)\;=\overset{2}{\underset{i=1}{\sum }}\overset{2}{\underset{j=1}{\sum }}R_{il}P\left(s(t+1)=s_{i}|s(t)=s_{j}\right)P\left(s(t)=s_{j}|r(1:t),c(1:t)\right)\;=\overset{2}{\underset{i=1}{\sum }}\overset{2}{\underset{j=1}{\sum }}R_{il}T_{ji}P\left(s(t)=s_{j}|r(1:t),c(1:t)\right)$$

where *l* can be either 1 (left choice) or 2 (right choice) and *P s*(*t*) = *s*_*j*_|*r*(1 : *t*); *c* : (1 : *t*)) is the probability of being block state *s*_*j*_ on trial *t* given the entire reward and choice history up to and including trial *t*. Equation 28 tells us that estimating the block state probability *P s*(*t*) = *s*_*j*_|*r*(1 : *t*); *c* : (1 : *t*)) will provide us with an estimate of the expected reward for a given choice on trial *t*+1 as *R*_*il*_ and *T*_*ji*_ are already known. Using Bayes’ theorem, we can estimate the block state probability as

(Equation 29) $$P\left(s(t)=s_{j}|r(1:t),c(1:t)\right)=\frac{P\left(r(t)|r(1:t-1),c(1:t),s(t)=s_{j}\right)P\left(s(t)=s_{j}|r(1:t-1),c(1:t)\right)}{\overset{2}{\underset{k=1}{\sum }}P\left(r(t)|r(1:t-1),c(1:t),s(t)=s_{k}\right)P\left(s(t)=s_{k}|r(1:t-1),c(1:t)\right)}$$

The first term in the numerator of the right-hand side of Equation 29, *P*(*r*(*t*)(1 : *t* − 1),*c*(1 : *t*),*s*(*t*) = *s*_*j*_), is the probability of receiving reward *r*(t)(1 if rewarded and 0 if unrewarded) on trial *t* given the current choice *c*(*t*) = *c*_*k*_, the block state *s*_*j*_, and the reward *r*(1:*t*-1) and the choice history *c*(1:*t*-1) up to trial *t*-1. Because the past history of rewards and choices does not affect the reward probability once the block state is known, this can be rewritten as

(Equation 30) $$P\left(r(t)|r(1:t-1),c(1:t),s(t)=s_{j}\right)=P\left(r(t)|c(t)=c_{k},s(t)=s_{j}\right)=R_{jk}^{r(t)}{\left(1-R_{jk}\right)}^{1-r(t)}$$

The second term in the numerator of the right-hand side of Equation 29, *P*(*s*(*t*) = *s*_*j*_|*r*(1 : *t* − 1),*c*(1 : *t*)) is the probability that the current block state is *s*_*j*_ given the reward choice history. This can be rewritten as

(Equation 31) $$P\left(s(t)=s_{j}|r(1:t-1),c(1:t)\right)\;=P\left(s(t)=s_{j}|r(1:t-1),c(1:t-1)\right)\;=\overset{2}{\underset{m=1}{\sum }}P\left(s(t)=s_{j}|s(t-1)=s_{m}\right)P\left(s(t-1)=s_{m}|r(1:t-1),c(1:t-1)\right)\;=\overset{2}{\underset{m=1}{\sum }}T_{mj}P\left(s(t-1)=s_{m}|r(1:t-1),c(1:t-1)\right)$$

In the second line above, the dependence on *c*(*t*) has been removed because the choice on the current trial, in the absence of reward information on the current trial, does not provide any additional information about the current state beyond that provided by the past reward and choice history. Combining Equations 29–31, the block state probability on the current trial *t* can be written in terms of the known reward probabilities, known state transition probabilities and the previous block state probability as

(Equation 32) $$P\left(s(t)=s_{j}|r(1:t),c(1:t)\right)=\frac{\overset{2}{\underset{m=1}{\sum }}R_{jk}^{r(t)}{\left(1-R_{jk}\right)}^{1-r(t)}T_{mj}P\left(s(t-1)=s_{m}|r(1:t-1),c(1:t-1)\right)}{\overset{2}{\underset{l=1}{\sum }}\overset{2}{\underset{m=1}{\sum }}R_{lk}^{\left(t\right)}{\left(1-R_{lk}\right)}^{1-r(t)}T_{ml}P\left(s(t-1)=s_{m}|r(1:t-1),c(1:t-1)\right)}$$

The above equation allows us to estimate the current trial block state probability *P*(*s*(*t*) = *s*_*j*_|*r*(1 : *t*), *c*(1 : *t*)) recursively, since it can be expressed in terms of the previous trial block state probability *P*(*s*(*t* − 1) = *s*_*m*_*|r*(1 : *t* − 1)), *c*(1 : *t* − 1)) and other known constant terms. This combined with the known reward and block transition probabilities allows the model to select the optimal choice according to Equation 28.

##### Q-learning model

To simulate trial-by-trial, model-free performance of the tasks, we used a Q-learning model in which the value of the chosen action is updated on each trial as follows:

(Equation 33) $$Q_{\text{right}}(t+1)=\{\begin{array}{ll} Q_{\text{right}}(t)+\alpha \left(r(t)-Q_{\text{right}}(t)\right), & if\;c(t)=\text{right}\\ Q_{\text{right}}(t), & if\;c(t)=\text{left} \end{array}\;Q_{\text{left}}(t+1)=\{\begin{array}{ll} Q_{\text{left}}(t), & if\;c(t)=\text{right}\\ Q_{\text{left}}(t)+\alpha \left(r(t)-Q_{\text{left}}(t)\right), & if\;c(t)=\text{left} \end{array}$$

where *Q*_right_ is the value for the right choice and *Q*_left_ is the value for the left choice. *t* is the current trial and *α* is the learning rate, which was set to 0.612 per trial. *r*(*t*) is the outcome of trial *t* (1 for reward, 0 for no reward). Q-values for each choice were initialized to 0. The outcome *r*(*t*) was determined based on the reward probability for choice *c*(*t*) given the block. Choice was simulated using a softmax equation such that the probability of choosing right or left is given by,

(Equation 34) $$p(c(t)=\text{right})=\frac{\text{exp}\left(\beta_{\text{value}}Q_{\text{right}}(t)+\beta_{\text{stay}}I_{\text{right}}(t)\right)}{\text{exp}\left(\beta_{\text{value}}Q_{\text{right}}(t)+\beta_{\text{stay}}I_{\text{right}}(t)\right)+\text{exp}\left(\beta_{\text{value}}Q_{\text{left}}(t)+\beta_{\text{stay}}I_{\text{left}}(t)\right)}\;p(c(t)=\text{left})=\frac{\text{exp}\left(\beta_{\text{value}}Q_{\text{left}}(t)+\beta_{\text{stay}}I_{\text{left}}(t)\right)}{\text{exp}\left(\beta_{\text{value}}Q_{\text{right}}(t)+\beta_{\text{stay}}I_{\text{right}}(t)\right)+\text{exp}\left(\beta_{\text{value}}Q_{\text{left}}(t)+\beta_{\text{stay}}I_{\text{left}}(t)\right)}$$

Where *β*_value_ is the inverse temperature parameter, which was set to 0.99. *β*_stay_ is a parameter accounting for how likely mice were to repeat their previous choice, which was set to 0.95. *I*_left/right_ is 1 if that action (i.e., left or right) was chosen on the previous trial and 0 otherwise. Parameters for the Q-learning model were fit in (Lee et al., 2019) to the behavior of mice in which dopamine neuron activity was recorded in Parker et al. (2016).

##### Comparison of RPE at block reversals

RPE for both the ideal-observer model and the Q-learning model (Figure S14) was defined as the difference between the experienced reward *r*(*t*) and the expected reward for the chosen action (ρ_chosen_(*t*) for the ideal-observer model or *Q*_chosen_(t) for the Q-learning model) as follows:

(Equation 35) $$RPE_{\text{Ideal Observer}}=r(t)-\rho_{\text{chosen}}(t)\;RPE_{Q-\text{learning}}=r(t)-Q_{\text{chosen}}(t)$$

To identify RPE signatures of model free versus model based performance of the two tasks, we compared the RPE from the ideal-observer model and the Q-learning model on trials around block reversals. Specifically, we compared the RPE from the two models on the first trial of a block with the RPE on the second trial of a block when the choice on trial 1 was different from the choice on trial 2. This means that any changes in RPE from trial 1 to trial 2 were inferred because the new action-outcome relationship for the choice made on trial 2 had not been explicitly experienced in the new block.

## Supplementary Material

1

2

## Figures and Tables

**Table:** KEY RESOURCES TABLE

REAGENT or RESOURCE	SOURCE	IDENTIFIER
Antibodies		
Mouse monoclonal anti-GFP	Life Technologies Corporation	Cat# G10362; RRID: AB_2536526
Donkey anti-rabbit coupled to Alexa 488	Jackson ImmunoResearch	Cat# 711-545-152; RRID: AB_2313584
Bacterial and virus strains		
CAV-Cre virus	IGMM Vector core, France	NA
retroAAV-Ef1a-NLS-Cre_WPRE-hGHpA	PNI Viral Core, Princeton	; RRID: Addgene_5536
AAV2/5-CAG-Flex-GCamp6f-WPRE-SV40	UPenn Vector Core	AV-5-PV2816; RRID: Addgene_100835
AAV2/5-CamKIIa-hChR2-EYFP	UNC Vector Core	https://www.addgene.org/26969; RRID: Addgene_26969
Experimental models: Organisms/strains		
Mouse: wild type C57BL/6J	Jackson Laboratory	JAX: 000,664; RRID: ISMR_JAX_000664
Software and algorithms		
“Synaptic plasticity” temporal difference learning algorithm	Generated by this study	https://github.com/baiydaavi/RL_models_with_choice_selective_sequences
“Neural dynamics” deep reinforcement learning algorithm	Generated by this study	https://github.com/baiydaavi/RL_models_with_choice_selective_sequences
Behavioral event encoding model	Generated by this study	https://github.com/nfparker/event_encoding_model
Other		
Fibers for optogenetics	Thor Labs	BFL37-300
Ferrules for optogenetics	Precision Fiber Products	MM-FER-2006SS-330
0.5mm diameter, ~6.1mm length GRIN lens	Inscopix	GLP-0561
Imaging baseplate	Inscopix	BPL-2
Baseplate cover	Inscopix	BPC-2

## Highlights

- Prelimbic cortex inputs to NAc (PL-NAc) encode actions and choice-selective sequences
- Midline thalamus inputs to NAc (mTH-NAc) preferentially encode cues
- PL-NAc supports learning in models based on synaptic plasticity or neural dynamics
- In models and mice, PL-NAc perturbations affect future, but not current, choice
