# Supplementary material for: Choice-selective sequences dominate in cortical relative to thalamic inputs to NAc to support reinforcement learning
Source: Cell Rep. Author manuscript; Available in PMC 2022 Jun 23. (PMC9218875; doi:10.1016/j.celrep.2022.110756)
Supplement: 1 [file NIHMS1812599-supplement-1.pdf]

Cell Reports, Volume *39*

Supplemental information

Choice-selective sequences dominate in cortical relative to thalamic inputs to NAc to support reinforcement learning

Nathan F. Parker, Avinash Baidya, Julia Cox, Laura M. Haetzel, Anna Zhukovskaya, Malavika Murugan, Ben Engelhard, Mark S. Goldman, and Ilana B. Witten

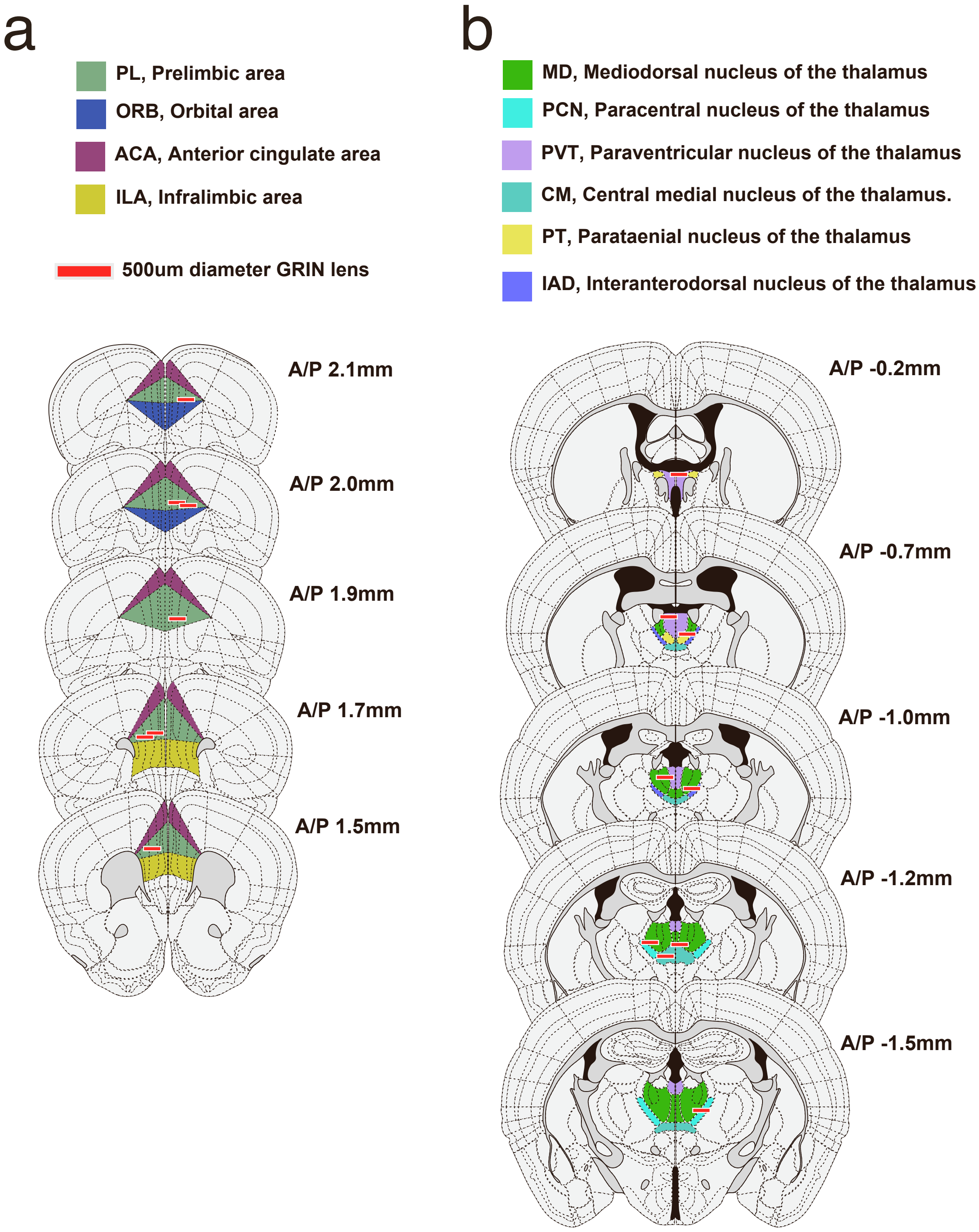

Supplementary Figure 1 - Locations of GRIN lens implants. Related to STAR METHODS. (a) Schematic of coronal sections along the anterior/posterior axis (A/P, numbers relative to bregma) with recording locations of 7 PL-NAc mice. Red lines indicate bottom of lens implant. **(b)** Same as **a** except location of 9 mTH-NAc recordings.

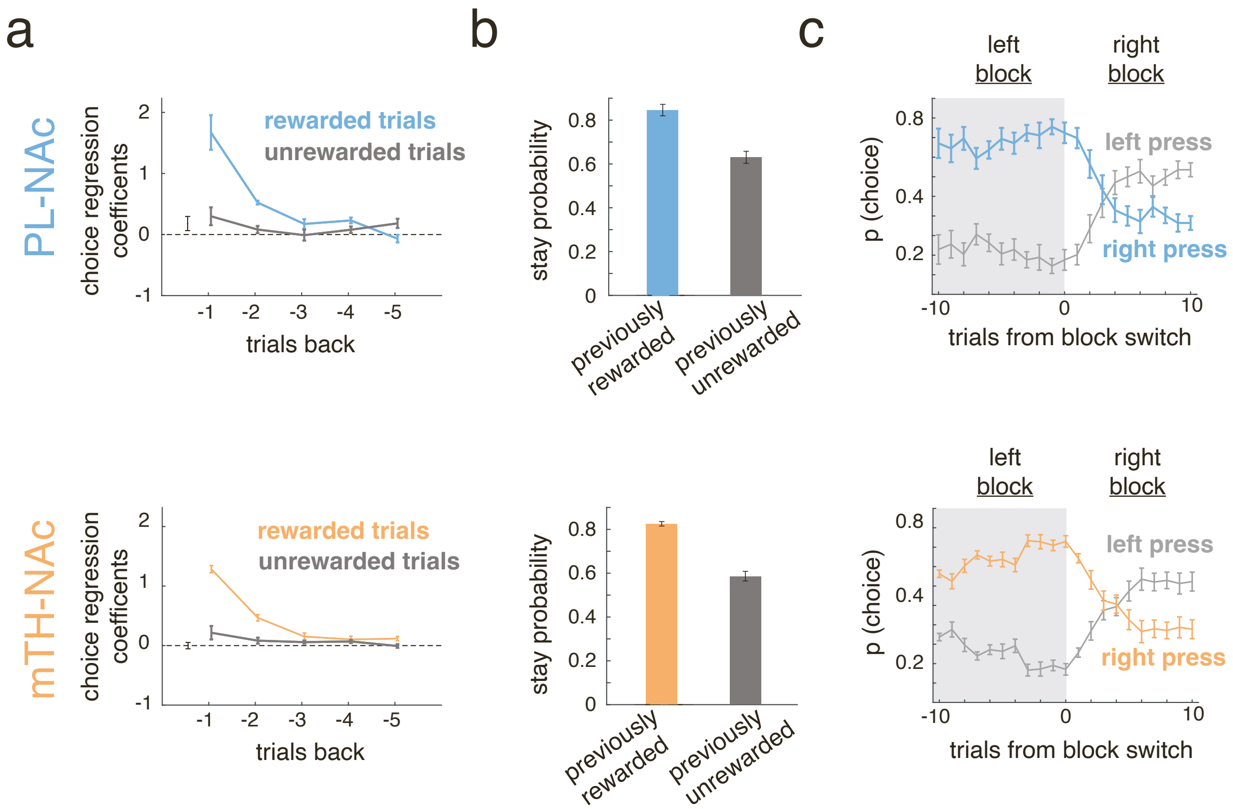

Supplementary Figure 2 - Mice in the PL-NAc and mTH-NAc imaging cohorts have comparable behavior. Related to Figure 1 and STAR METHODS. (a) Top**,** coefficients from logistic regression to predict choice (see **Figure 1**) from PL-NAc imaging cohort (n=7 mice). Bottom, same except coefficients from mTH-NAc imaging cohort (n=9 mice). Both cohorts use choice and outcome information from previous trials to predict the current choice. Regression coefficients between the two cohorts are not significantly different for any trials back for either rewarded or unrewarded trials (P>0.01, unpaired, two-tailed t-test of regression coefficients across mice at each trial back, n=7 and 9 mice for PL-NAc and mTH-NAc, respectively). **(b)** Stay probability following rewarded (blue or orange) and unrewarded (grey) trials for PL-NAc (top) and mTH-NAc (bottom) cohorts. Both cohorts have a significantly higher stay probability following a rewarded trial (PL-NAc: P=0.00008; mTH-NAc: P=0.00003, paired, two-tailed t-test comparing stay probability on rewarded and unrewarded trials across mice, n=7 and 9 mice for PL-NAc and mTH-NAc, respectively). **(c)** Probability of a left or right lever press following a reversal from a left-preferring to right-preferring block of mice from the PL-NAc (top, n=7 mice) and mTH-NAc (bottom, n=9 mice) cohorts. Both cohorts display a qualitatively similar change in choice behavior following a block reversal. In all panels, data are represented as mean +/- SEM across mice.

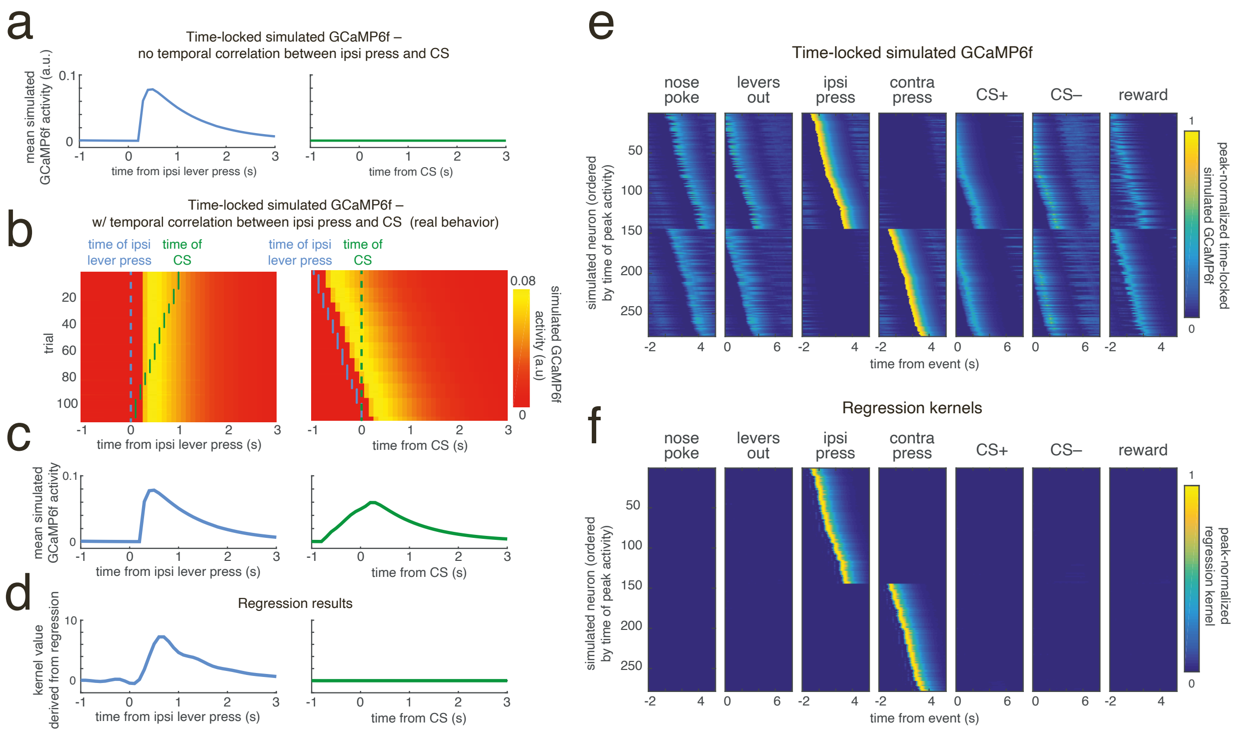

Supplementary Figure 3 - Simulated neural activity to illustrate the ability of the encoding model to successfully relate neural activity to the appropriate behavioral event. Related to Figure 2 and STAR METHODS. (a) Simulated neuron that is responsive only to the ipsilateral lever press. **(b)** Trial-by-trial heatmap of a simulated neuron that has increased activity time-locked to the ipsilateral lever press. In the data, there was a correlation between the time of lever press and the time of the CS, which produced a time-locked response to the CS, even though the neuron did not respond to that event. Left, activity heatmap aligned to the time of an ipsilateral lever press (dashed blue line) sorted by the time of the subsequent CS presentation (green dots). Right, activity heatmap is aligned to the time of the CS (dashed green line), ordered by the time of the preceding lever press (blue dots). **(c)** Average activity across trials of the example simulated neuron in **b** aligned to the lever press, left, and CS presentation, right. Unlike the idealized case in **a**, when the timing of task events is maintained from the real behavior, the temporal correlations result in a bump in activity aligned to the CS (right plot). Note that this bump in activity is generated entirely by the correlation in event times, since this simulated neuron only had activity in relation to the lever press (and not the CS presentation). **(d)** Response kernels for lever press, left, and CS, right, derived from the encoding model used to attribute the neural response of individual task events. The model successfully recovers the fact that neural activity in this simulated neuron is related to the lever press and not the CS. **(e)** Heatmap displaying the average activity from a population of 278 simulated neurons that respond to either the ipsilateral or contralateral lever press, but not the other events. Each neuron responds to the lever press, with a randomly assigned response latency from -1 to 3s. While the strongest average time-locked response is to the ipsilateral or contralateral lever presses, there are visible responses to the other task events as a consequence of the correlation between task events resulting from their temporal proximity. **(f)** Same as **e** except heatmap displays the response kernels derived from the encoding model. The model successfully discovers the underlying structure of the data (i.e., that responses are driven by the lever press).

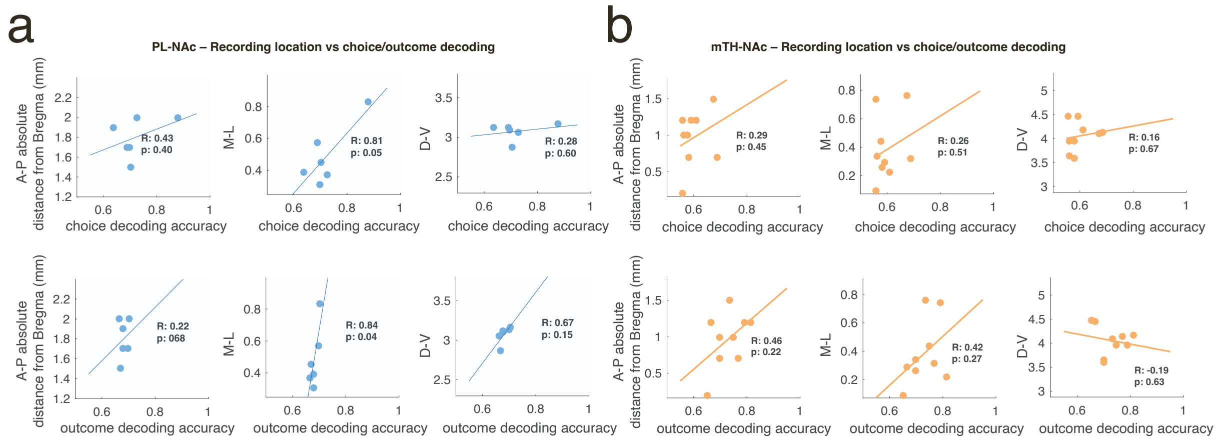

Supplementary Figure 4 - Lack of correlation between recording locations relative to Bregma and choice / outcome decoding. Related to Figure 3. (a) Top, correlations between choice decoding accuracy using recorded PL-NAc activity and, in order, the anterior/posterior (A/P), medial/lateral (M/L) and dorsal/ventral (D/V) recording locations relative to Bregma (see **Supplemental Figure 1** for schematic of recording locations; recording locations were aligned to the Allen atlas using the Wholebrain software suite (http://www.wholebrainsoftware.org/) of Furth et al. (2018); see Methods for details, n=6 mice). Bottom, same as top except correlation between recording location and outcome decoding accuracy using PL-NAc activity. **(b)** Same as **a** except decoding accuracy for choice (top) and outcome (bottom) determined using recorded mTH-NAc activity (n=9 mice). All p-values are calculated from Pearson's correlation coefficient; none are significant at the $p<.05$ level after correction for multiple (6) hypotheses using Bonferroni correction.

a

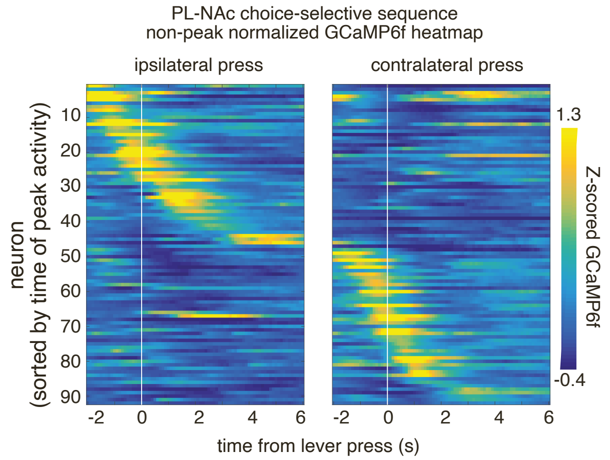

Supplementary Figure 5 - Choice-selective sequences in PL-NAc neurons without peak-normalization. Related to Figure 4. Heatmap demonstrating sequential response of choice-selective PL-NAc neurons to the ipsilateral and contralateral lever press (n=92 neurons from 7 mice). Similar to **Figure 4b-c**, but time-locked, trial-averaged GCaMP6f fluorescence is not normalized by the peak response to the lever press and is taken from all trials.

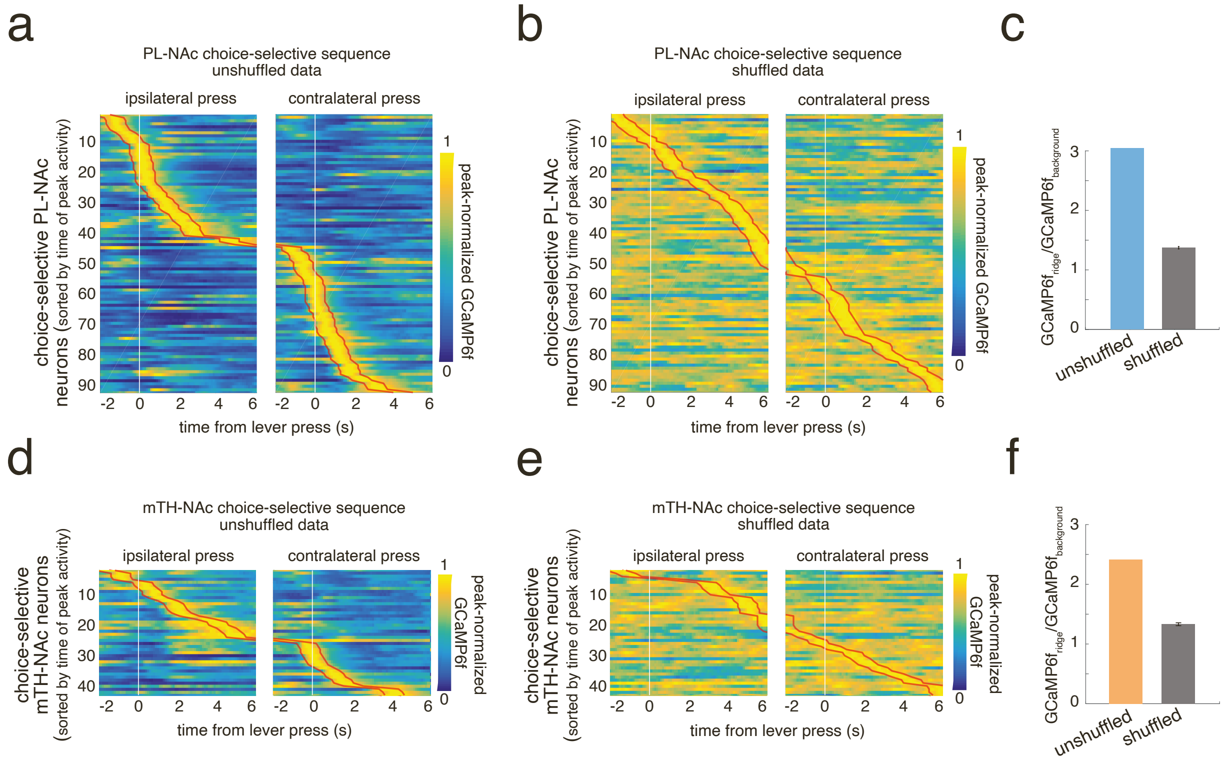

Supplementary Figure 6 – The calculated ridge-to-background ratio of PL-NAc neurons supports the presence of sequences. Related to Figure 4. (a) Sequential activity of PL-NAc choice-selective neurons. Similar to **Figure 4b-c,** the heatmap is ordered by the time of peak activity time-locked to the ipsilateral (left column) and contralateral (right column) lever press of each neuron**,** but instead of cross-validation, activity is averaged across all trials. Red trace represents the borders of the one-second window around the peak defined as the 'ridge'. Activity at all other surrounding timepoints is considered the 'background'. **(b)** Same as **a** for data that is shuffled by temporally shifting the GCaMP6f fluorescence trace across a recording session separately for each neuron by a random number of frames, chosen from a uniform distribution. Ordering by the time of peak activity generates spurious sequential activity across the diagonal in shuffled data. **(c)** Calculated ridge-to-background ratio of PL-NAc neurons using unshuffled (blue) and shuffled (grey) data. A ratio is calculated for each individual neuron and the average of these ratios across all neurons displayed in the heatmap is shown. The ratio calculated from unshuffled data is significantly larger than that from the shuffled data (P<0.0001, comparison between unshuffled data and distribution of 500 shuffled iterations). Error bars for shuffled data indicate one standard deviation. **(d-f)** Same as **a-c** but ridge-to-background is calculated using mTH-NAc neural recordings. Similar to PL-NAc, the ratio calculated from unshuffled data was significantly larger than that from the shuffled data (P<0.0001). However, when comparing across the populations, the ridge-to-background calculated using PL-NAc neurons (3.06+/-0.12, mean+/-sem, n=92 neurons from 7 mice) was significantly larger than that using mTH-NAc (2.42+/-0.12, mean+/-sem, n=42 neurons from 9 mice; P=0.004: unpaired, two-tailed t-test comparing ratio between PL-NAc and mTH-NAc neurons).

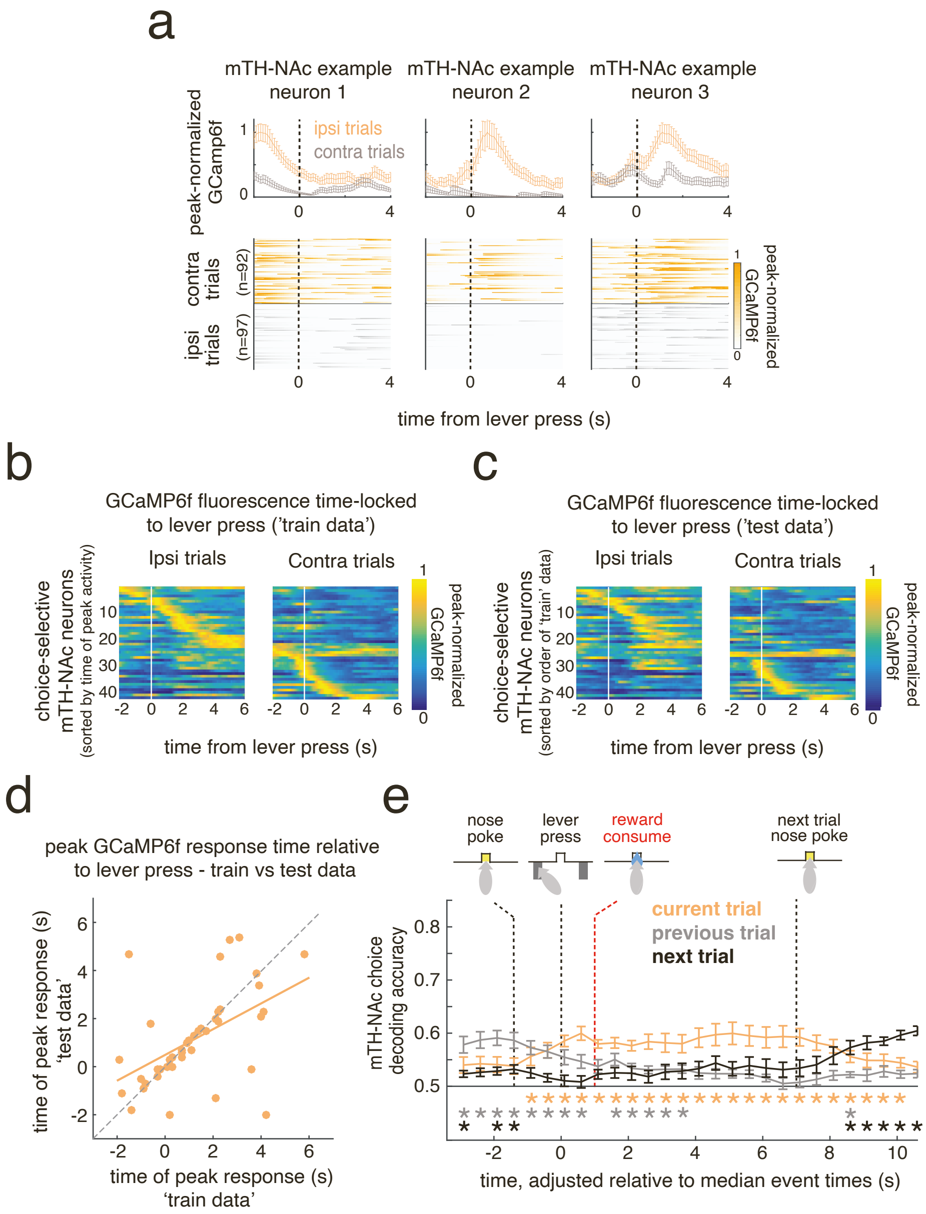

Supplementary Figure 7 - mTH-NAc choice-selective neurons display sequential activity that is less consistent than PL-NAc. Related to Figure 4. (a) Top; average GCaMP6f fluorescence of three simultaneously imaged mTH-NAc choice-selective neurons with different response times relative to the lever press. Error bars are s.e.m across trials. Bottom, heatmaps of GCaMP6f fluorescence response across trials to ipsilateral (orange) and contralateral (grey) lever presses. **(b,c)** Heatmaps of choice-selective mTH-NAc neurons' peak-normalized GCaMP6f responses to lever press (n=42/256 neurons from 9 mice). Each row is the average GCaMP6f fluorescence time-locked to the ipsilateral (left column) and contralateral (right column) lever press for a neuron, normalized by the neuron's peak average fluorescence. In **b** ('train data'), heatmap is generated using a randomly selected half of trials and ordered by the time of each neuron's peak activity. In **c** ('test data'), the peak-normalized, time-locked GCaMP6f fluorescence from the other half of trials was used while maintaining the order from 'train data' in **b**. Compare to PL-NAc data in **Figure 4b-c**. **(d)** Correlation between the time of peak activity using the 'train' (horizontal axis) and 'test' (vertical axis) trials for choice-selective mTH-NAc neurons. While mTH-NAc choice-selective neurons also show significant correlation between 'train' and 'test' trials ($R^2 = 0.51$, $P = 5.5 \times 10^{-4}$, n=42 neurons from 9 mice), this correlation is significantly lower than that of PL-NAc (comparison with data in **Figure 4d**; P=0.005, Z=2.81, Fisher's R-to-Z transformation, comparison of correlation coefficients derived from comparing peak activity

between 'test' and 'training' data from PL-NAc versus mTH-NAc). **(e)** Average choice decoding accuracy of the mice's choice on the current (orange), previous (grey) and next trial (black) as a function of GCaMP6f fluorescence throughout the current trial. GCaMP6f fluorescence is taken from 100 random selections per mouse of 10 simultaneously imaged mTH-NAc neurons (each trial's activity is adjusted in a piecewise linear manner relative to the median time of the nose poke, lever press and next trial nose poke, see Methods for details). Data are represented as mean +/- SEM across mice (n=9 mice). Red dashed line indicates median onset of reward consumption. * indicate significant decoding accuracy above chance, P<0.01, two-tailed, one-sample t-test across mice.

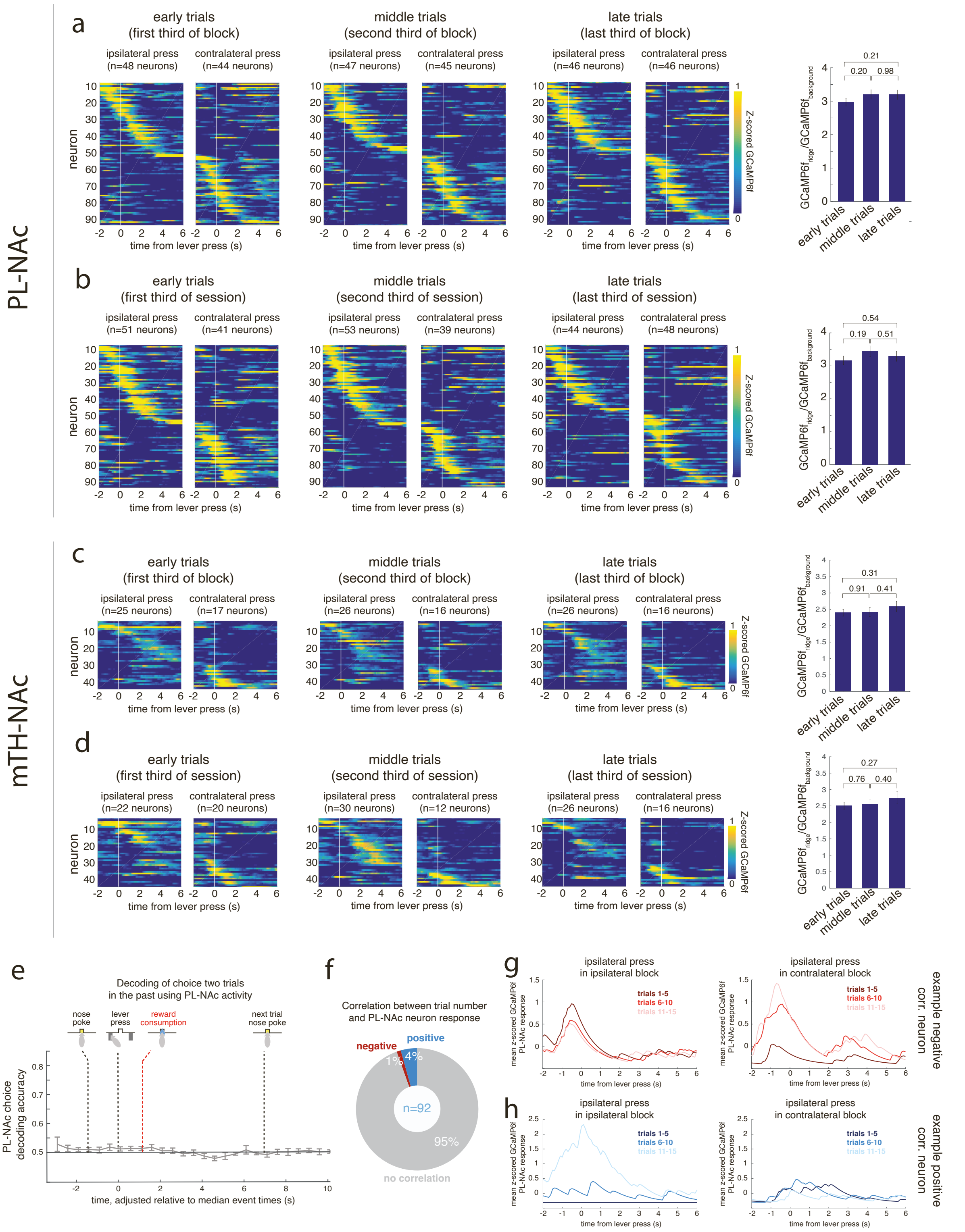

Supplementary Figure 8 – Trial number within a block, and two-trial-back choice, are not strongly encoded by PL-NAc activity. Related to Figure 4. (a) Left, heatmaps of Z-scored GCaMP6f activity from 92 choice-selective PL-NAc neurons averaged across the first, middle and last third of trials of each block. Right, calculated ridge-to-background ratio derived from average activity from each third of trials within a block. Data are represented as mean +/- SEM across neurons (n=92 from 7 mice). No significant changes are observed in the ratios calculated across a block, suggesting that the strength of sequences is not modulated by block trial number (p>0.05, paired,

two-tailed t-test). **(b)** Same as **a** except data is split into the first, middle and final third of the entire recording session. **(c,d)** Same as **a,b** except activity is of mTH-NAc choice-selective neurons. **(e)** Decoding accuracy for the mice's choice two trials back using activity from 10 simultaneously recorded PL-NAc neurons. Unlike choice decoding on the current and previous trial (**Figure 4e;** blue and black traces, respectively), PL-NAc activity is not able to accurately decode choice from two trials back after correcting for cross-trial choice correlations (see Methods for details) at any time point in the trial ($p>0.05$ for all time points: one-sample, two-tailed t-test across mice comparing decoding accuracy with chance rate of 0.5). **(f)** Proportion of PL-NAc choice-selective neurons whose activity is significantly positively (blue, n=4 neurons) or negatively (red, n=1 neuron) correlated with the number of trials into a block ($P<0.01$). Significance was determined by comparing the calculated correlation coefficient of each neuron to a null distribution of 500 correlation coefficients generated using GCaMP6f signal circularly shifted by a random integer, to control for slow drift in the data. R-values were calculated using the maximum GCaMP6f activity from 2s before to 6s after the time of lever press for the first 15 trials in a block. Using the same criteria, no mTH-NAc neurons were significantly correlated, either positively or negatively with trial number in a block ($p>0.05$). **(g)** Left, average activity of the negatively correlated PL-NAc neuron in response to an ipsilateral lever press at various trials in an ipsilateral block, where the ipsilateral lever had a higher probability of reward and, thus, the value of the ipsilateral lever increases as a function of trial number. Right, average activity of the negatively correlated PL-NAc neuron in response to an ipsilateral press in a contralateral block. **(h)** Same as **g** except for an example of a positively correlated PL-NAc neuron.

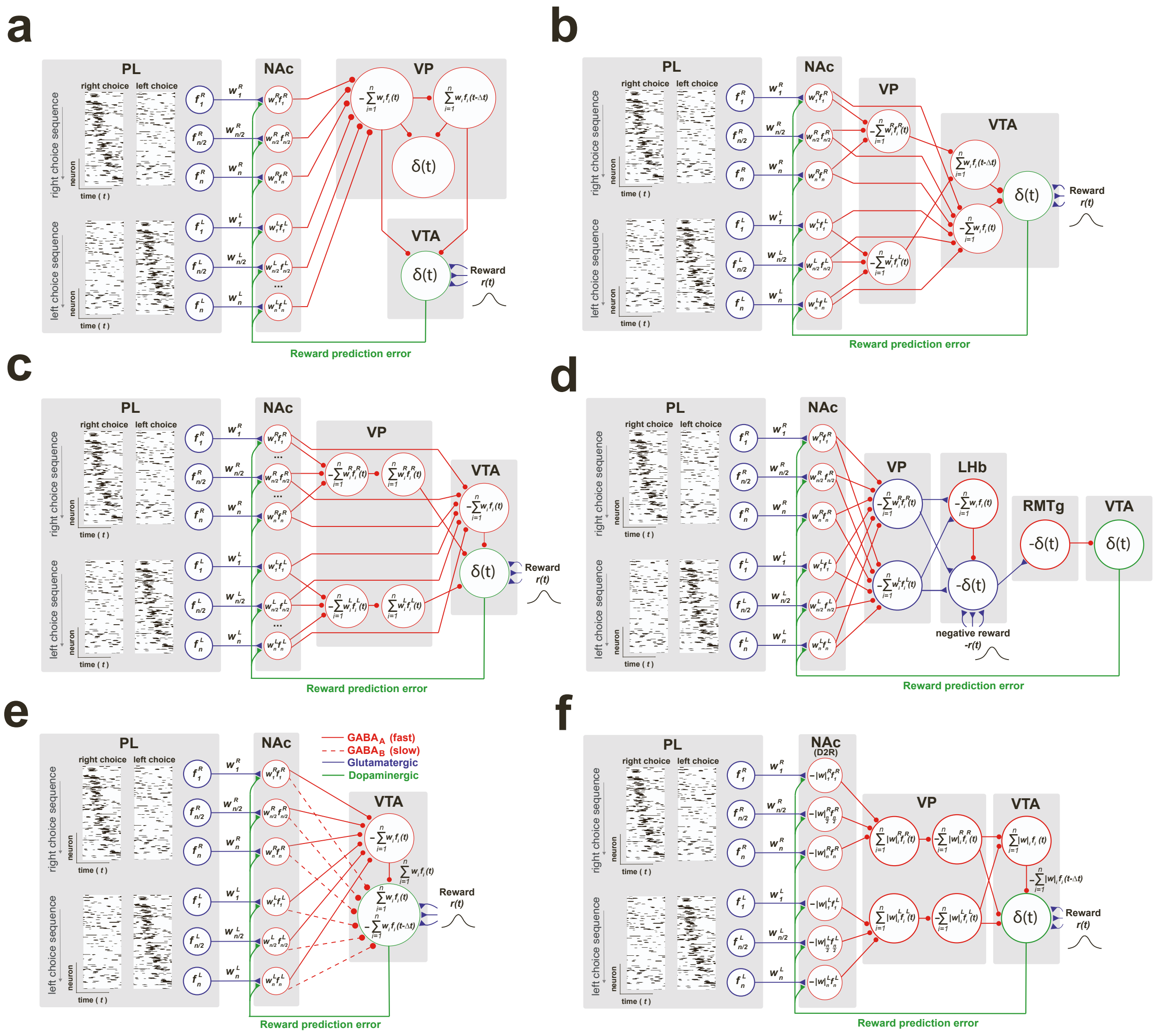

Supplementary Figure 9 - Alternative model architectures used to implement synaptic plasticity model. Related to Figure 5. (a-f) Alternative models constructed using known circuit architecture. All models except **a,d** generate an RPE signal by providing a 'fast excitatory' and 'slow-inhibitory' value pathway to the VTA dopamine neuron population. Note that all model variants rely on choice-selective sequences in PL-NAc to bridge the beginning of the action sequence and outcomes across time. For all model variants, GABAergic, glutamatergic and dopaminergic projections are denoted as red, blue and green, respectively. Brain region abbreviations are: prelimbic cortex, PL; nucleus accumbens, NAc; ventral pallidum, VP; ventral tegmental area, VTA; lateral habenula, LHb; rostromedial tegmental nucleus, RMTg. **(a)** In this model, the delay and inversion of the value signal is accomplished through a second VP neuron. These two VP neurons converge onto a third VP interneuron to generate an RPE signal in VP, as has been observed by (Ottenheimer et al., 2020). **(b)** In this model, the fast excitatory pathway is generated via a direct projection of NAc neurons onto the VTA GABA neuron while the slow inhibitory pathway passes through the VP before synapsing onto a VTA GABA neuron. **(c)** Similar to **b** except that the slow inhibitory pathway contains an additional VP neuron, which accomplishes the sign inversion and delay assigned to a VTA GABA neuron in **b**. Since the models in **b** prescribe a role for the observed NAc-D1R projections to VTA

GABA neurons, they produce negative value signals in VTA GABA neurons, whereas only positive value signals have been observed experimentally in identified GABA interneurons in the VTA (Cohen et al., 2012)**. (d)** In this model, a negative reward prediction error is calculated in the LHb using glutamatergic projections from the VP (Tooley et al., 2018), inversion and delay in the value signal from local inhibitory LHb neurons to produce an inverted RPE, which is then transmitted to the VTA via the RMTg (Webster and Wozny, 2020; Li et al., 2019). **(e)** To account for previous work describing direct projections from NAc D1R neurons to the VTA (Beier et al., 2015; Watabe-Uchida et al., 2012; Yang et al., 2018), this alternative model architecture has NAc neurons projecting directly to the VTA, skipping the VP. In this model, the timing difference needed to compute an RPE signal is generated through the activity of fast ionotropic GABA-A receptors (solid red trace), which have been shown to preferentially express in NAc-VTA GABA interneuron projection postsynaptic densities (Edwards et al., 2017), while activity of metabotropic GABA-B receptors (dashed red trace), which are preferentially expressed in the postsynaptic densities of NAc-VTA DA projections (Edwards et al., 2017), generate the slow-inhibitory pathway. Notably, without this differential expression of GABA receptors in the DAergic and GABAergic populations of the VTA, this model architecture would fail to produce an RPE signal, as it would instead generate a fast-inhibitory and slow-excitatory signal in the VTA DA neuron population. **(f)** Multiple studies have implicated D2-R expressing MSNs as playing a critical role in reversal learning in multiple mammalian species (Boulougouris et al., 2009; DeSteno and Schmauss, 2009; Eisenegger, 2014; Kruzich and Grandy, 2004; Kruzich et al., 2006; Kwak et al., 2014; Piray, 2011). Thus, in this model we account for the possibility that the reversal behavior in our task is mediated specifically by changes to synaptic weights from PL to D2-R-expressing NAc MSNs. This model assumes the opposite dopamine-mediated plasticity rule (LTD rather than LTP) than the previous models.

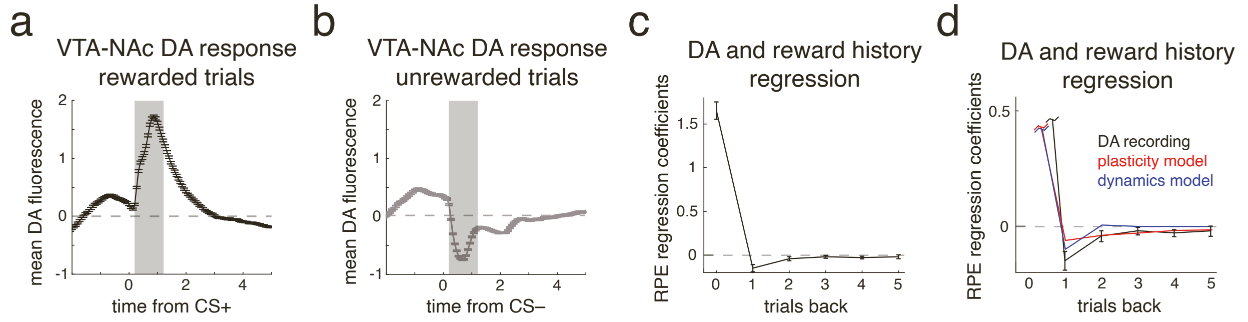

Supplementary Figure 10 - Reward prediction error (RPE) encoding observed in recorded dopamine (DA) activity is similar to that produced by our synaptic plasticity and neural dynamics models. Related to Figures 5 and 6. (a) Mean bulk GCaMP6f fluorescence from VTA-NAc DA terminals in response to a conditioned stimulus signaling reward (CS+, data taken from (Parker et al., 2016, n=11 recording sites). Note that terminal fluorescence recordings are presented here to more accurately reflect the signal that downstream NAc neurons are receiving in our model. **(b)** Same as **a** except DA fluorescence in response to the conditioned stimulus signaling an unrewarded trial (CS–). **(c)** Coefficients from a multiple linear regression in which outcome is predicted using mean DA fluorescence signals from 0.2-1.2s relative to the time of CS presentation across current (“0”) and multiple previous trials (see shaded region in **a,b**), similar to **Figure 5g,l,q** and **Figure 6e**. The positive coefficient for the current trial and negative coefficients for previous trials indicate the encoding of an RPE. **(d)** Same as **c**, but also including coefficients from the synaptic plasticity model (red, same coefficients as **Figure 5g**) and the neural dynamics model (black, same coefficients as **Figure 6e**), to allow direct comparison. Error bars in all panels represent s.e.m across 11 recording sites.

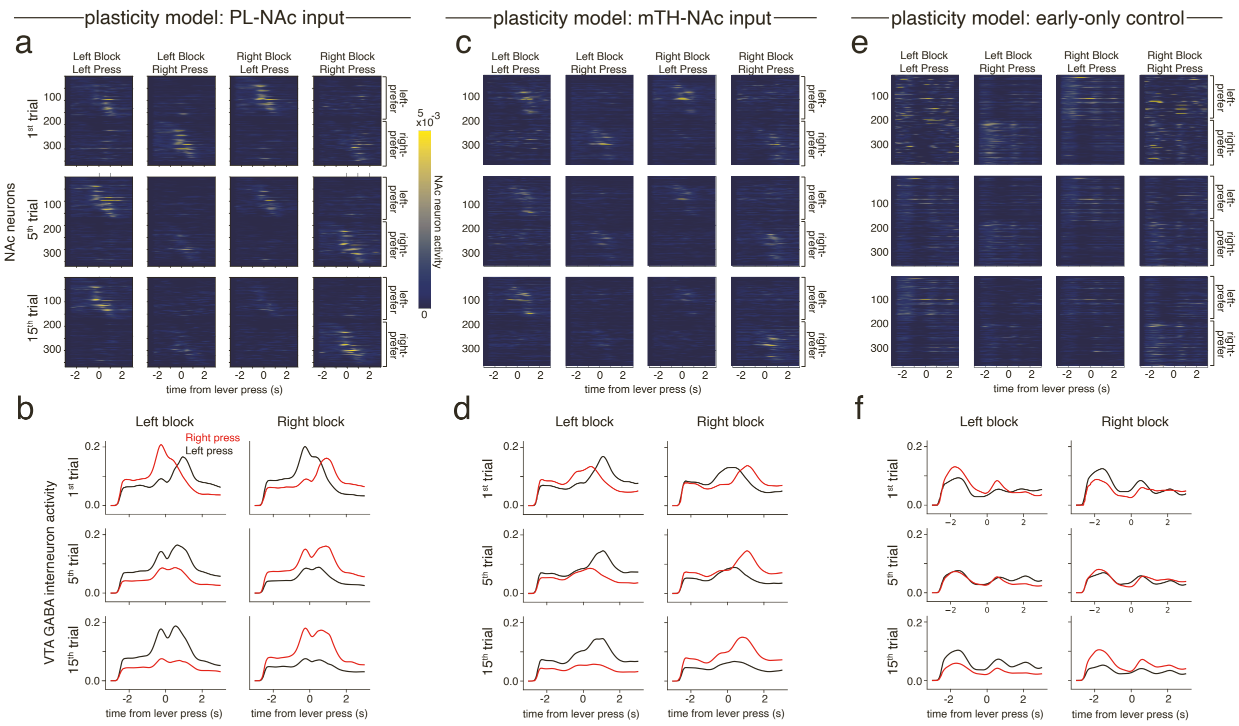

Supplementary Figure 11 - Synaptic plasticity model using sequential PL-NAc but not early-only or mTH-NAc activity correctly modulates activity in NAc projection neurons and VTA GABA interneurons. Related to Figure 5. (a) Heatmaps of average activity relative to the time of the lever press for NAc projection neurons in the PL-NAc model (**Figure 5c**). Top, middle and bottom heatmaps are the average activity across the first, fifth and fifteenth trial of each block, respectively. Each column is the average activity across trials from different block/press combinations. For each subplot, neurons 1-184 are left-preferring and neurons 185-368 are right-preferring. The activity of these left- and right-preferring NAc neurons increases throughout a block of their respective lever preference. In contrast, their activity decreases throughout a block opposite to their lever preference. **(b)** Average activity of VTA GABA interneuron from synaptic plasticity model using PL-NAc activity as input on left (black) or right (red) trials. Activity is relative to the time of the lever press across the first, fifth and fifteenth trials of a left-preferring (left column) or right-preferring (right column) block. Similar to **a**, throughout a left block (left column), the activity on left press trials increases from the first to fifteenth trial while the activity on right press trials decreases. The opposite pattern is seen for left and right press trials throughout a right block (right column). **(c,d)** Same as **a,b** except NAc and VTA GABA interneuron generated using mTH-NAc as input to the synaptic plasticity model. **(e,f)** Same as **a,b** except NAc and VTA GABA interneuron from the early-only control synaptic plasticity model.

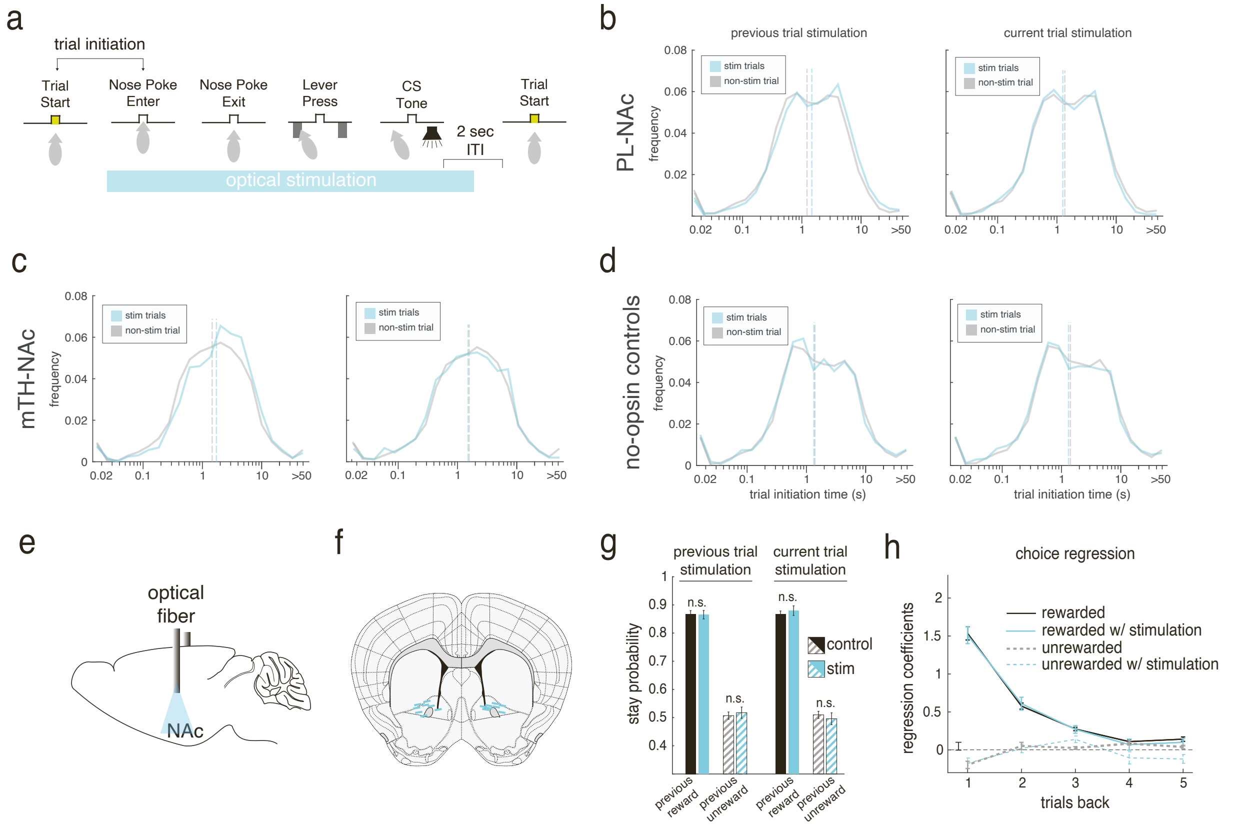

Supplementary Figure 12 – Laser stimulation affects trial initiation times in both PL-NAc and mTH-NAc but does not affect behavior in control mice that do not express opsin. Related to Figure 7. (a) Schematic of trial structure and time of optical stimulation. "trial initiation" time is defined as the latency between the start of the trial and the mouse entering the central nose poke. **(b)** Left, distribution of trial initiation times following stimulation trials (blue) and non-stimulation trials (grey) in PL-NAc ChR2 mice. Blue and grey vertical lines indicate median initiation times for stim and non-stim trials, respectively. Right, same only effect of current trial stimulation on trial initiation times. Previous trial stimulation resulted in significantly longer trial initiations in PL-NAc ChR2 mice than no-opsin control mice (P=3.46x10^-7, p-value from the 'opsin group X previous trial stimulation' interaction term of a mixed effects model used to predict latency times of PL-NAc and control mice, fit using the fitglme function in MATLAB; see Methods for additional model details). In contrast, current trial stimulation had no significant effect on initiation times (P=0.95, same test as above except p-value is that of the interaction term of 'opsin group X current trial stimulation'), an expected result as the start of stimulation was contingent on the mouse performing a nose poke. **(c)** mTH-NAc ChR2 mice had significantly longer trial initiation times following optical stimulation than no-opsin control mice (P=2.34x10^-4, p-value from the 'opsin group X previous trial stimulation' interaction term of a mixed effects model used to predict latency times of mTH-NAc and control mice) but no effect of current trial stimulation was observed (P=0.74, same test as above except the p-value is that from the 'opsin group X current trial stimulation' interaction term). **(d)** Same as **b,c** except latencies from no-opsin control cohort. **(e)** Surgical schematic of no-opsin control cohort. Optical fibers were implanted into the NAc. **(f)** Optical fiber tip locations of no-opsin control cohort (n=8 mice). **(g)** Unlike PL-NAc ChR2 expressing mice (**Figure 7f-h**), neither current nor previous trial stimulation changed the stay probability in control mice following rewarded (P=0.52: previous trial stimulation; P=0.24: current trial stimulation; paired t-test) or unrewarded trials (P=0.52: previous trial stimulation; P=0.47: current trial stimulation; paired t-test). **(h)** Likewise, stimulation from multiple trials back had no effect on choice (P>0.05 for all trials back, t-test across mice's laser x choice interaction term coefficients). Data in **g,h** are represented as mean +/- SEM across mice, n=8.

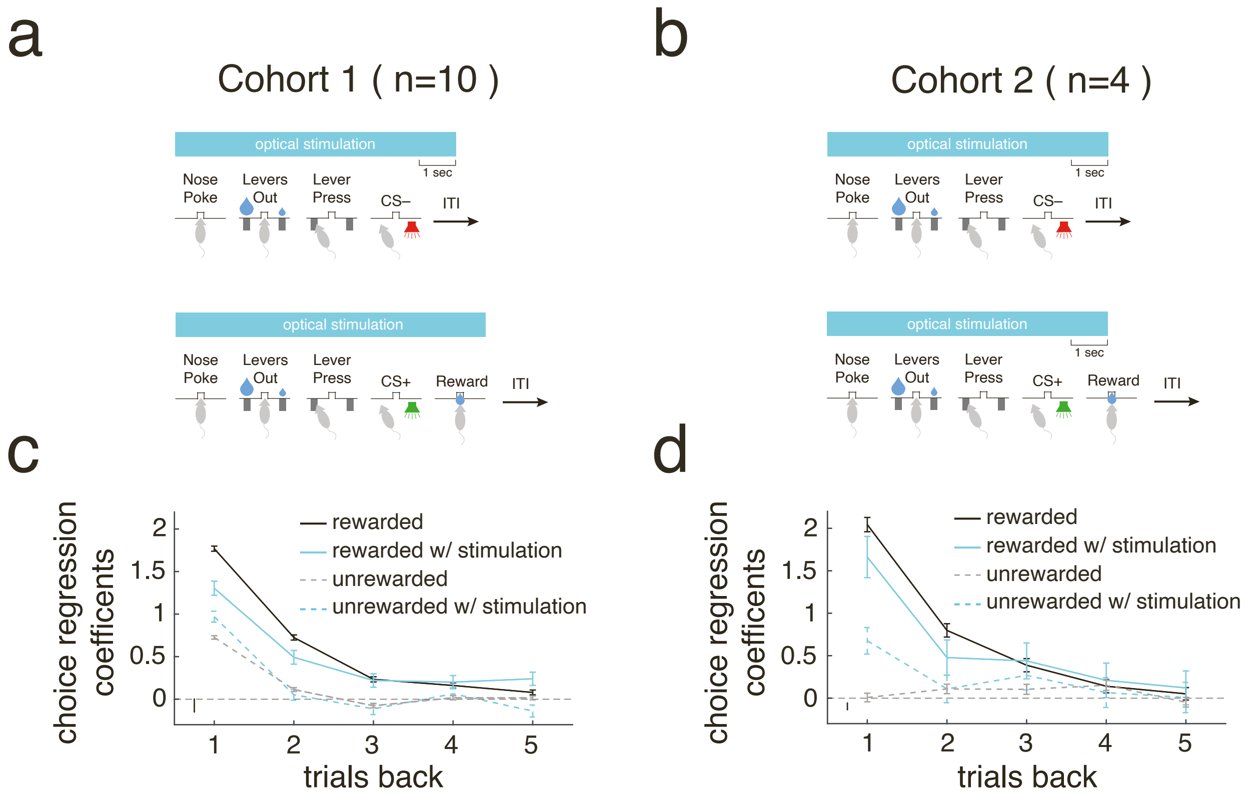

Supplementary Figure 13 - Effect of PL-NAc optogenetic stimulation in two cohorts. Related to Figure 7. (a) Schematic of optical stimulation parameters for cohort 1. On 10% of unrewarded trials, optical stimulation began when the mouse entered the central nosepoke and ended 1s into the intertrial interval (ITI), which began at the end of the 500ms CS– tone. On 10% of rewarded trials, stimulation began with nose poke and ended after the mouse left the reward port. **(b)** Schematic for cohort 2. Unlike cohort 1, optical stimulation ended on the same timescale on both rewarded and unrewarded trials, 1s after the end of CS presentation. **(c)** Logistic regression model similar to that in **Figure 1e** demonstrating the effect of PL-NAc stimulation on lever choice in cohort 1 mice (n=10 mice, see Methods for model details). Rewarded trials with stimulation one and two trials back decreased stay probability compared with rewarded trials without stimulation. Stimulation had an opposite effect on unrewarded trials, for which there was an increase in stay probability following stimulation one trial back compared to trials without stimulation. **(d)** Same as **c** except data from cohort 2 (n=4 mice). Effect of optical stimulation of PL-NAc neurons was qualitatively similar across the two cohorts. Data in **c,d** are represented as mean +/- SEM across mice.

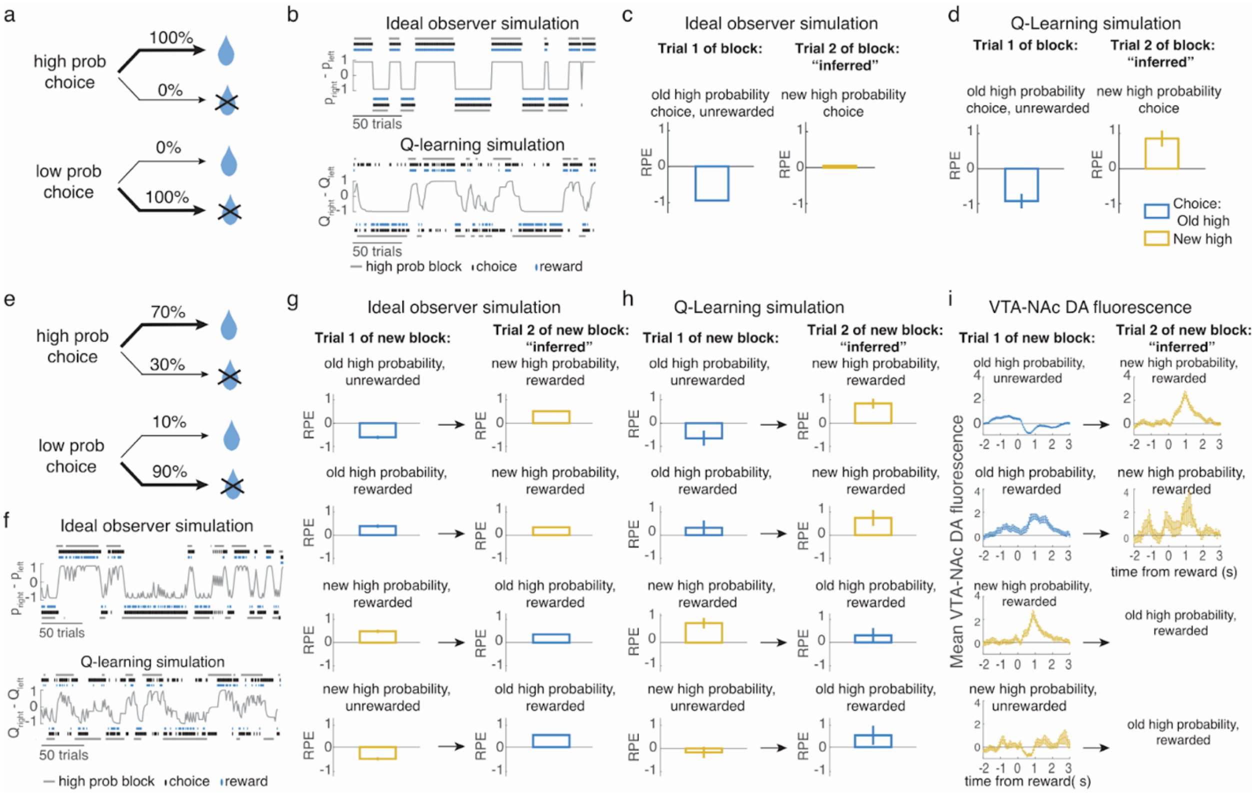

Supplementary Figure 14 - Similar RPE signatures for ideal observer and Q-learning simulation during reversal learning. Related to Figure 5 and STAR METHODS. Given previous evidence that dopamine signals can reflect knowledge of task structure (Bromberg-Martin et al., 2010; Sadacca et al., 2016), we used modeling to gain insight into how clearly RPE in the probabilistic reversal learning task (a bandit task) can indicate the use of model-based inference of block reversals for different reward probability structures. This was done by simulating task performance using an ideal observer model with knowledge of the block structure, and a Q-learning model which did not have information about the block structure (see Methods for more details). **(a-d)** To confirm that our ideal observer simulation captured a previously reported RPE signature of model-based block reversal inference, we first simulated behavior of 100,000 trials of a task similar to that used in (Bromberg-Martin et al., 2010). **(a)** In this task, one option was rewarded 100% of the time while the other was never rewarded, and the identity of the high probability choice randomly reversed with a probability of 0.05 on each trial. **(b)** Example performance of the ideal observer simulation (top) and the Q-learning simulation (bottom). Choice is determined by the difference between expected reward for the available actions, ρ, for the ideal observer and the difference between the action values, Q, for the Q-learning simulation (these values are plotted in grey). **(c)** To evaluate RPE signatures of model-based block reversal inference, we compared the estimated RPE (experienced reward minus expected reward for the chosen action) on trial 1 and trial 2 of the new block. The RPE on trial 2 was low for the high probability choice in the new block even without direct experience of that action-outcome pairing. This means that the ideal observer infers the block reversal, so the new, not yet experienced reward contingency is expected and the RPE is low. **(d)** In contrast, because the Q-learning model only updates the value of the chosen action, on trial 2, when the simulation is rewarded for the previously low-probability choice, the reward remains unexpected and the RPE is high. **e-i)** Simulated performance of 100,000 trials using the reward probabilities from this study. **(e)** The high probability action was rewarded 70% of the time while the low probability action was rewarded 10% of the time and the blocks reversed according to the same rule as in **a**. **(f)** Example performance of the ideal observer (top) and the Q-learning (bottom) simulations in this task. **(g)** To determine whether there is a strong qualitative RPE signature of block reversal inference in this task, we compared RPE on the 4 possible trial-1 types to RPE on the subsequent rewarded switch trials (i.e. choice on trial 2 was different than trial 1, meaning that any changes in RPE must be inferred). We focus on rewarded trials to aid comparison with reward responses recorded in dopamine terminals during this task (Parker et al., 2016). In this case, inference of the block reversal is not obviously reflected in the RPE, since the RPE for a given action on trial 1 and trial 2 are similar (comparing the same color bars for rewarded actions on trials 1

and 2). This is because, even though the ideal observer updates the predicted reward for both the chosen and unchosen actions, when reward delivery is probabilistic, predicted reward remains moderate for both actions and RPE changes only subtly. **(h)** Same as in **g** for the Q-learning simulation. As expected, RPE looks very similar on trial 1 and trial 2 for a given rewarded action because the Q-learning simulation does not update the value of the unchosen action on trial 1. **(i)** Consistent with the results from both simulations, GCaMP6f z-scored dF/F from dopaminergic axons in the NAc recorded in (Parker et al., 2016) is also very similar for a given rewarded action on trial 1 and trial 2. Note that the mice did not make all possible choices in this task, so some trial types are missing. The simulations also rarely made these choices (e.g., switch following a rewarded new high probability choice). Error bars in **c-h** are SEM across block transitions. Data in **i** are represented as mean +/- SEM across 11 recording sites).

Supplemental References

Boulougouris, V., Castañé, A., and Robbins, T.W. (2009). Dopamine D2/D3 receptor agonist quinpirole impairs spatial reversal learning in rats: investigation of D3 receptor involvement in persistent behavior. Psychopharmacology *202*, 611–620.

DeSteno, D.A., and Schmauss, C. (2009). A role for dopamine D2 receptors in reversal learning. Neurosci. *162*, 118–127.

Edwards, N.J., Tejeda, H.A., Pignatelli, M., Zhang, S., McDevitt, R.A., Wu, J., Bass, C.E., Bettler, B., Morales, M., and Bonci, A. (2017). Circuit specificity in the inhibitory architecture of the VTA regulates cocaine-induced behavior. Nat. Neurosci. *20*, 438–448.

Eisenegger, C., Naef, M., Linssen, A., Clark, L., Gandamaneini, P.K., Müller, U., and Robbins, T.W. (2014). Role of dopamine D2 receptors in human reinforcement learning. Neuropsychopharmacology *39,* 2366-2375.

Kruzich, P.J., and Grandy, D.K. (2004). Dopamine D2 receptors mediate two-odor discrimination and reversal learning in C57BL/6 mice. BMC Neurosci. *5*, 12.

Kruzich, P.J., Mitchell, S.H., Younkin, A., and Grandy, D.K. (2006). Dopamine D2 receptors mediate reversal learning in male C57BL/6J mice. Cogn. Affect. Behav. Neurosci. *6*, 86–90.

Kwak, S., Huh, N., Seo, J.-S., Lee, J.-E., Han, P.-L., and Jung, M.W. (2014). Role of dopamine D2 receptors in optimizing choice strategy in a dynamic and uncertain environment. Front. Behav. Neurosci. *8*.

Ottenheimer, D.J., Bari, B.A., Sutlief, E., Fraser, K.M., Kim, T.H., Richard, J.M., Cohen, J.Y., and Janak, P.H. (2020). A quantitative reward prediction error signal in the ventral pallidum. Nat. Neurosci. *23*, 1267–1276.

Piray, P. (2011). The role of dorsal striatal D2-like receptors in reversal learning: a reinforcement learning viewpoint. J. Neurosci. *31*, 14049–14050.

Sadacca, B.F., Jones, J.L., and Schoenbaum, G. (2016). Midbrain dopamine neurons compute inferred and cached value prediction errors in a common framework. Elife *5*.

Tooley, J., Marconi, L., Alipio, J.B., Matikainen-Ankney, B., Georgiou, P., Kravitz, A.V., and Creed, M.C. (2018). Glutamatergic Ventral Pallidal Neurons Modulate Activity of the Habenula–Tegmental Circuitry and Constrain Reward Seeking. Biol. Psychiatry *83*, 1012–1023.

Webster, J.F., and Wozny, C. (2020). Behavior: Local Lateral Habenula Interneurons Mediate Aggression. Curr. Biol. *30*, R954–R956.

Yang, H., de Jong, J.W., Tak, Y., Peck, J., Bateup, H.S., and Lammel, S. (2018). Nucleus accumbens subnuclei regulate motivated behavior via direct inhibition and disinhibition of VTA dopamine subpopulations. Neuron *97*, 434–449.e4.
