# Supplementary material for: Choice-selective sequences dominate in cortical relative to thalamic inputs to NAc to support reinforcement learning
Source: Cell Rep. Author manuscript; Available in PMC 2022 Jun 23. (PMC9218875; doi:10.1016/j.celrep.2022.110756)
Supplement: 2 [file NIHMS1812599-supplement-2.pdf]

Cell Reports

Article

Choice-selective sequences dominate in cortical relative to thalamic inputs to NAc to support reinforcement learning

Graphical abstract

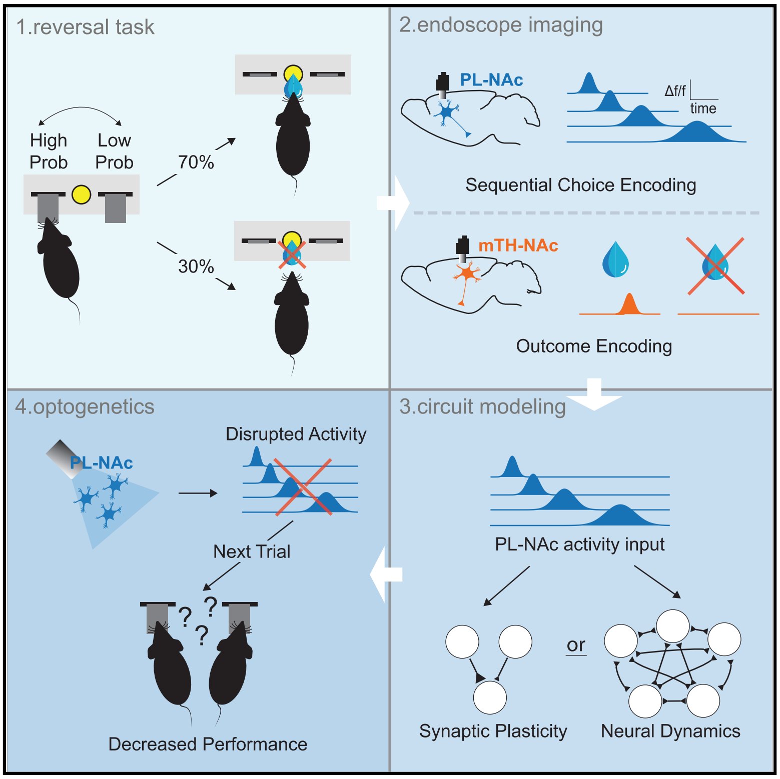

Authors

Nathan F. Parker, Avinash Baidya, Julia Cox, ..., Ben Engelhard, Mark S. Goldman, Ilana B. Witten

Correspondence

msgoldman@ucdavis.edu (M.S.G.), iwitten@princeton.edu (I.B.W.)

In brief

Learning to make proper choices requires associating outcomes with the actions that preceded them. Parker et al. record and optogenetically manipulate two input pathways to a key brain area associated with action-outcome learning, the nucleus accumbens. The results motivate two alternative models for the circuitry and mechanisms underlying reinforcement learning.

Highlights

- Prelimbic cortex inputs to NAc (PL-NAc) encode actions and choice-selective sequences
- Midline thalamus inputs to NAc (mTH-NAc) preferentially encode cues
- PL-NAc supports learning in models based on synaptic plasticity or neural dynamics
- In models and mice, PL-NAc perturbations affect future, but not current, choice

Parker et al., 2022, Cell Reports *39*, 110756
May 17, 2022 © 2022 The Author(s).
https://doi.org/10.1016/j.celrep.2022.110756

CellPress

Cell Reports

CellPress
OPEN ACCESS

Article

Choice-selective sequences dominate in cortical relative to thalamic inputs to NAc to support reinforcement learning

Nathan F. Parker,[1,8] Avinash Baidya,[3,6,8] Julia Cox,[1,7,8] Laura M. Haetzel,[1] Anna Zhukovskaya,[1] Malavika Murugan,[1] Ben Engelhard,[1] Mark S. Goldman,[3,4,5,*] and Ilana B. Witten[1,2,9,*]
[1]Princeton Neuroscience Institute, Princeton University, Princeton, NJ 08544, USA
[2]Department of Psychology, Princeton University, Princeton, NJ 08544, USA
[3]Center for Neuroscience, University of California, Davis, Davis, CA 95616, USA
[4]Department of Neurobiology, Physiology and Behavior, University of California, Davis, Davis, CA 95616, USA
[5]Department of Ophthalmology and Vision Science, University of California, Davis, Davis, CA 95616, USA
[6]Department of Physics and Astronomy, University of California, Davis, Davis, CA 95616, USA
[7]Department of Neuroscience, Feinberg School of Medicine, Northwestern University, Chicago, IL 60611, USA
[8]These authors contributed equally
[9]Lead contact
*Correspondence: msgoldman@ucdavis.edu (M.S.G.), iwitten@princeton.edu (I.B.W.)
https://doi.org/10.1016/j.celrep.2022.110756

SUMMARY

How are actions linked with subsequent outcomes to guide choices? The nucleus accumbens, which is implicated in this process, receives glutamatergic inputs from the prelimbic cortex and midline regions of the thalamus. However, little is known about whether and how representations differ across these input pathways. By comparing these inputs during a reinforcement learning task in mice, we discovered that prelimbic cortical inputs preferentially represent actions and choices, whereas midline thalamic inputs preferentially represent cues. Choice-selective activity in the prelimbic cortical inputs is organized in sequences that persist beyond the outcome. Through computational modeling, we demonstrate that these sequences can support the neural implementation of reinforcement-learning algorithms, in both a circuit model based on synaptic plasticity and one based on neural dynamics. Finally, we test and confirm a prediction of our circuit models by direct manipulation of nucleus accumbens input neurons.

INTRODUCTION

Multiple lines of experimental evidence implicate the nucleus accumbens (NAc, part of the ventral striatum) in reward-based learning and decision-making (Apicella et al., 1991; Cador et al., 1989; Carelli et al., 1993; Cox and Witten, 2019; Di Ciano et al., 2001; Everitt et al., 1991; Parkinson et al., 1999; Phillips et al., 1993, 1994; Robbins et al., 1989; Roitman et al., 2005; Setlow et al., 2003; Stuber et al., 2011; Taylor and Robbins, 1986). The NAc is a site of convergence of glutamatergic inputs from a variety of regions, including the prefrontal cortex and the midline thalamus, along with dense dopaminergic inputs from the midbrain (Brog et al., 1993; Do-Monte et al., 2017; Groenewegen et al., 1980; Hunnicutt et al., 2016; Otis et al., 2017; Phillipson and Griffiths, 1985; Poulin et al., 2018; Reed et al., 2018; Swanson, 1982; Wright and Groenewegen, 1995; Zhu et al., 2016).

A central question in reinforcement learning is how actions and outcomes become associated with each other, even when they are separated in time (Asaad et al., 2017; Gersch et al., 2014; Sutton, 1988; Wörgötter and Porr, 2005). A possible mechanism that could contribute to solving this problem of temporal credit assignment in the brain is that neural activity in the glutamatergic inputs to the NAc provides a neural memory trace of previous actions. However, whether glutamatergic inputs to the NAc indeed represent memories of previous actions is unclear. More broadly, what information is carried by glutamatergic inputs to the NAc during reinforcement learning, and whether different inputs provide overlapping or distinct streams of information, has not been examined systematically. To date, there have been relatively few recordings of cellular-resolution activity of glutamatergic inputs to the NAc during reinforcement learning or comparison of multiple inputs within the same task, nor examination of the timescale with which information is represented within and across trials. Furthermore, if glutamatergic inputs do indeed provide memories of previous actions, construction of a neurally plausible instantiation of an algorithm for credit assignment based on the measured signals remains to be demonstrated (for a review of biological instantiation of reinforcement learning algorithms, see Joel et al., 2002).

To address these gaps, we recorded from glutamatergic inputs to the NAc during a probabilistic reversal learning task

This is an open access article under the CC BY license (http://creativecommons.org/licenses/by/4.0/).

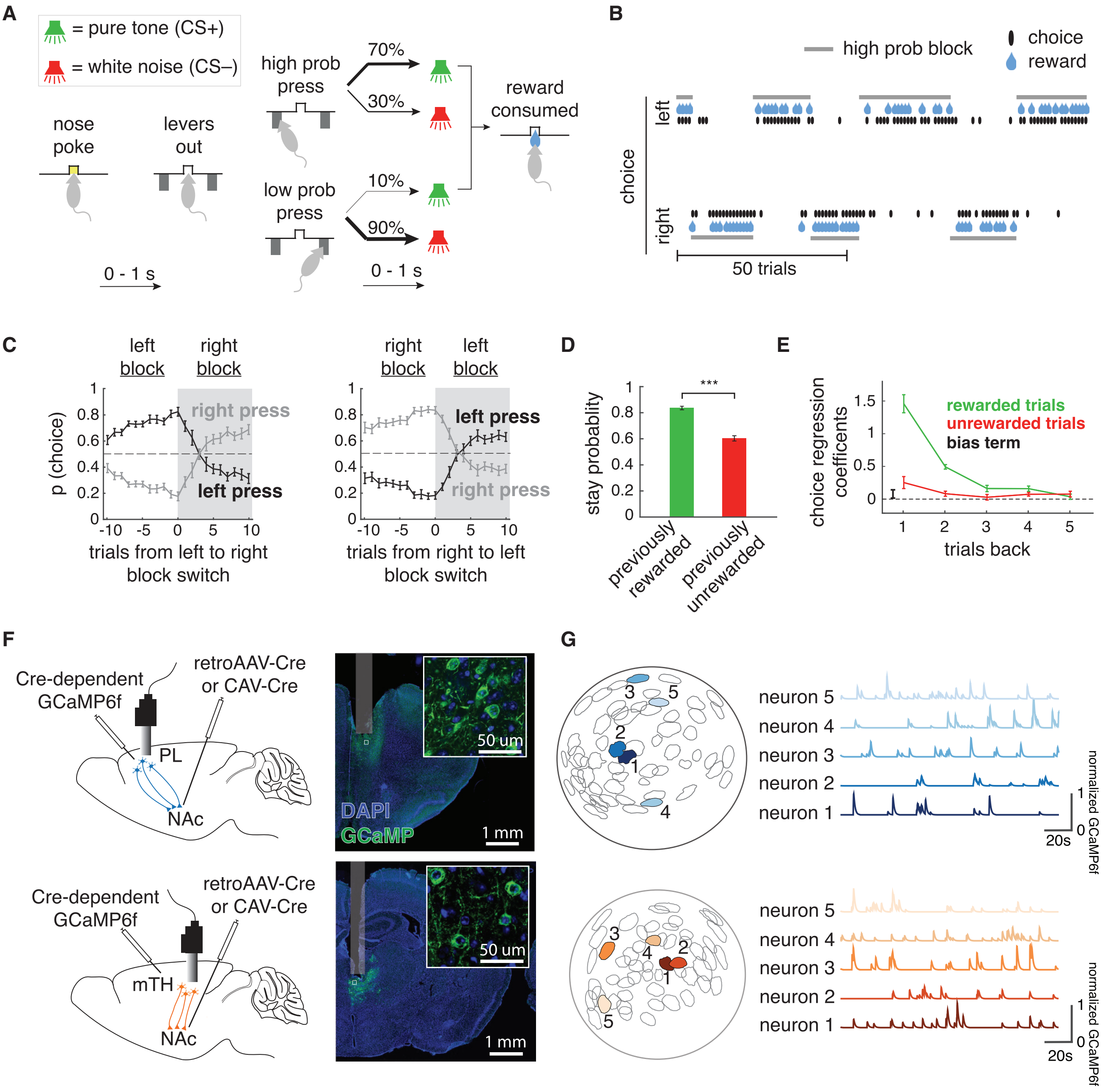

Figure 1. Cellular-resolution imaging of PL and mTH neurons that project to the NAc in mice performing a reinforcement learning task

(A) Schematic of probabilistic reversal learning task.

(B) Example behavior during a recording session. The choice of the mouse (black marks) follows the identity of the high-probability lever as it alternates between left and right (gray lines).

(C) Left: probability the mice choose the left or right lever ten trials before and after a reversal from a left-to-right high-probability block. Right: same as left for right-to-left high-probability block reversals.

(D) Mice had a significantly higher stay probability following a rewarded versus unrewarded trial (***$p = 5 \times 10^{-9}$, two-tailed t test, n = 16 mice).

(E) Coefficients from a logistic regression that uses choice and outcome from the previous five trials to predict choice on the current trial. Positive regression coefficients indicate a greater likelihood of repeating the previous choice.

(F) Left: surgical schematic for PL-NAc (top) and mTH-NAc (bottom) recordings showing the injection site and optical lens implant with miniature head-mounted microscope attached. Right: coronal section from a PL-NAc (top) and mTH-NAc (bottom) mouse showing GCaMP6f expression in the recording sites. Inset: confocal image showing GCaMP6f expression in individual neurons.

(legend continued on next page)

and built circuit-based computational models to connect our data to prominent theories of reinforcement learning. In this task, dopamine neurons that project to the NAc encode reward prediction error (RPE), and inhibition of dopamine neurons substitutes for a negative RPE (Parker et al., 2016). To compare activity in major cortical and thalamic inputs to the NAc core, we combined a retrograde viral targeting strategy with cellular-resolution imaging to examine the inputs from prelimbic cortex ("PL-NAc," part of the medial prefrontal cortex) and midline regions of the thalamus ("mTH-NAc"). We found that PL-NAc neurons preferentially encode actions and choices relative to mTH-NAc neurons, with choice-selective sequential activity that bridges the delay between choice and reward and that persists until the start of the subsequent trial. We demonstrate with computational modeling that these choice-selective sequences can support neural instantiations of reinforcement learning algorithms, either through dopamine-dependent changes in synaptic weights onto NAc neurons (Fisher et al., 2017; Gerfen and Surmeier, 2011; Reynolds and Wickens, 2002; Russo et al., 2010) or dopamine-dependent changes in neural dynamics (Wang et al., 2018). Finally, we test and confirm a prediction of our models through direct optogenetic manipulation of PL-NAc neurons. Thus, by recording and manipulating glutamatergic inputs to the NAc and integrating these data with computational modeling, we provide specific proposals for how reinforcement learning could be implemented by neural circuitry.

RESULTS

Cellular-resolution imaging of glutamatergic inputs to the NAc during a probabilistic reversal learning task

Mice performed a probabilistic reversal learning task while inputs from thalamus or cortex were imaged (Figure 1A). A trial was initiated when the mouse entered a central nose poke, which prompted the presentation of a lever on either side after a variable delay of 0–1 s. Each lever had either a high (70%) or low (10%) reward probability, with the identity of the high- and low-probability levers reversing in an unsignaled manner after a variable number of trials (see STAR Methods for block-reversal probabilities). After a variable delay (0–1 s), either a sound (positive conditioned stimulus [CS+]) was presented at the same time as a reward was delivered to a central reward port, or another sound (CS−) was presented that signaled the absence of reward.

As expected, mice switched the lever they were more likely to press following block reversals (Figures 1B and 1C). Similarly, mice were significantly more likely to return to the previously chosen lever (i.e., stay) following rewarded, as opposed to unrewarded, trials (Figure 1D), meaning that, as expected, mice were using previous choices and outcomes to guide behavior. A logistic regression to predict choice based on previous choices and outcomes indicated that mice relied on ~3 previous trials to guide their choices (Figure 1E; see STAR Methods for choice regression details).

To image activity of glutamatergic input neurons to the NAc during this behavior, we injected a retroAAV or CAV2 to express Cre-recombinase in the NAc as well as an AAV2/5 to Cre-dependently express GCaMP6f in either the PL or mTH (Figure 1F). A gradient refractive index (GRIN) lens was implanted above either the PL or mTH (see Figure S1 for implant locations), and a head-mounted miniature microscope was used to image activity in these populations during behavior (Figures 1F and 1G, n = 278 neurons in PL-NAc from n = 7 mice, n = 256 neurons in mTH-NAc from n = 9 mice). Behavior between mice in the PL-NAc versus mTH-NAc cohorts was similar (Figure S2).

Actions are preferentially represented by PL-NAc neurons, while reward-predicting stimuli are preferentially represented by mTH-NAc neurons

Individual PL-NAc and mTH-NAc neurons displayed elevated activity when time-locked to specific behavioral events in the task (Figure 2A). Given the correlation between the timing of task events, as well as the temporal proximity of events relative to the time course of GCaMP6f, we built a linear encoding model to properly relate neural activity to each event (Engelhard et al., 2019; Krumin et al., 2018; Lovett-Barron et al., 2019; Musall et al., 2019; Park et al., 2014; Parker et al., 2016; Pinto and Dan, 2015; Sabatini, 2019; Steinmetz et al., 2019). In brief, time-lagged versions of each behavioral event (e.g., nose poke, lever press) were used to predict the GCaMP6f fluorescence in each neuron using a linear regression. This allowed us to obtain "response kernels," which related each event to the GCaMP6f fluorescence in each neuron, while removing the potentially confounding (linear) contributions of correlated task events (Figure 2B; see STAR Methods for details). To visualize the response kernels we plotted them as a heatmap, where each row was the response kernel for a particular neuron associated with each behavioral event. This heatmap was then ordered by the time of peak kernel value across all behavioral events. Visual observation revealed a clear difference between the PL-NAc and mTH-NAc populations: PL-NAc neurons were robustly modulated by the action events in our task (Figure 2C; kernel values associated with "nose poke," "ipsilateral lever press," "contralateral lever press," and "reward consumption") while mTH-NAc neurons appeared to be most strongly modulated by the stimulus events, specifically the positive reward auditory cue (Figure 2D, kernel values associated with "CS+").

Examination of the GCaMP6f fluorescence time-locked to each behavioral event (rather than the encoding model-derived response kernels) revealed similar observations of action encoding in PL-NAc and CS+ encoding in mTH-NAc (Figures 2E and 2F). While this time-locked GCaMP6f heatmap displays neurons which appear to respond to multiple events (Figure 2E, see neurons approximately 70–170 that show elevated activity to "lever press," "levers out," and "nose poke"), this impression is likely a result of the temporal correlation between neighboring behavioral events, which our encoding model accounts for. To

(G) Left: example field of view from a recording in PL-NAc (top, blue) and mTH-NAc (bottom, orange) with five representative regions of interest (ROIs). Right, normalized GCaMP6f fluorescence traces from the five ROIs on the left. For visualization, each trace was normalized by the peak fluorescence across the hour-long session.
Data in (C), (D), and (E) are presented as mean ± SEM across mice (n = 16).

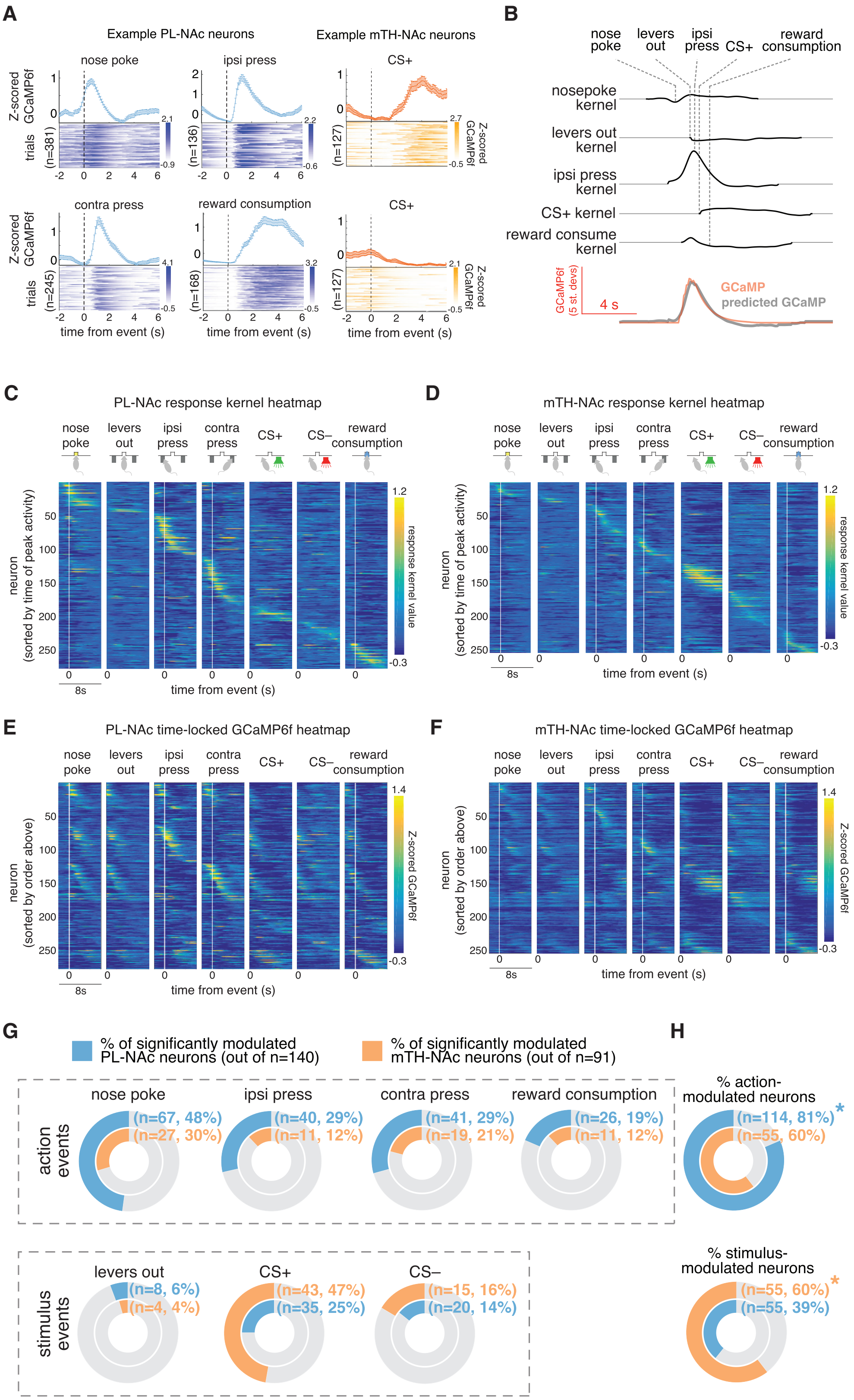

(legend on next page)

illustrate this, we applied our encoding model to a population of simulated neurons that responded only to the lever press events. We observed a similar multi-peak heatmap when simply time-locking the simulated GCaMP6f fluorescence, but this multi-peak effect is eliminated by the use of our encoding model, which recovers the true relationship between GCaMP6f fluorescence and behavior in the simulated data (Figure S3).

This encoding model was used to identify neurons in the PL-NAc and mTH-NAc populations that were significantly modulated by each event in our task (by comparing the encoding model with and without each task event, see STAR Methods). We found that a substantial fraction of both PL-NAc and mTH-NAc neurons were modulated by at least one task event (Figure 2G). Of these neurons that were selective to at least one task event, the selectivity for actions versus sensory stimuli differed between the two populations (Figure 2H). In particular, more PL-NAc neurons were modulated by at least one action event (nose poke, ipsilateral lever press, contralateral lever press, and reward consumption). By contrast, a significantly larger fraction of mTH-NAc neurons were modulated by at least one stimulus cue (levers out, CS+, and CS−).

PL-NAc neurons preferentially encode choice relative to mTH-NAc neurons

This preferential representation of actions in PL-NAc relative to mTH-NAc suggests that lever choice (contralateral versus ipsilateral to the recording site) could also be preferentially encoded in PL-NAc. Indeed, a significantly larger fraction of neurons were choice-selective in PL-NAc compared with mTH-NAc (Figure 3A; significant choice selectivity was determined with a nested comparison of the encoding model with and without choice information, see STAR Methods). A logistic regression population decoder supported this observation of preferential choice selectivity in PL-NAc relative to mTH-NAc (Figure 3B).

In contrast to the preferential representation of choice in PL-NAc compared with mTH-NAc, a larger fraction of neurons in mTH-NAc encoded outcome (CS identity or reward consumption) compared with PL-NAc (Figure 3C). However, while outcome decoding accuracy in mTH-NAc was slightly higher relative to PL-NAc, this difference was not statistically significant (Figure 3D). These results suggest that, unlike the preferential choice representation observed in PL-NAc over mTH-NAc, outcome was more similarly represented between these two populations. This is presumably due to the fact that both CS+ and reward consumption responses contribute to outcome representation, and although more neurons encoded CS+ in mTH-NAc, the opposite was true for reward consumption (Figure 2G). We found no obvious relationship between the strength of either choice or outcome decoding and recording location in either PL-NAc or mTH-NAc (Figure S4).

PL-NAc neurons display choice-selective sequences that persist into the next trial

We next examined the temporal organization of choice-selective activity in PL-NAc neurons. Across the population, choice-selective PL-NAc neurons displayed sequential activity with respect to the lever press that persisted for >4 s after the press (Figures 4A–4C; see Figure S5 for sequences without peak normalization). These sequences were visualized by time-locking the GCaMP6f fluorescence of choice-selective neurons with respect to the lever press, rather than with the encoding model from the earlier figures. The robustness of these sequences was confirmed using a cross-validation procedure in which the order of peak activity across the PL-NAc choice-selective population was first established using half of the trials (Figure 4B, “train”), after which the population heatmap was plotted using the same established ordering and activity from the other half of the trials (Figure 4C, “test”). To quantify the consistency of these sequences, we correlated the neurons’ time of peak activity in the “training” and “test” data and observed a strong correlation (Figure 4D). Additionally, the ridge-to-background ratio, a metric used to confirm the presence of sequences (Akhlaghpour et al., 2016; Harvey et al., 2012; Kondo et al., 2017), was significantly higher when calculated using the PL-NAc choice-selective sequences compared with sequences generated using shuffled data (Figures S6A–S6C).

In contrast, choice-selective sequential activity in the mTH-NAc population was significantly less consistent than in PL-NAc (Figures S7A–S7D). Additionally, while the ridge-to-background ratio of the sequences generated using mTH-NAc activity was significantly higher than that using shuffled data, this ratio was also significantly lower than that obtained from

Figure 2. PL-NAc preferentially represents action events while mTH-NAc preferentially represents the CS+

(A) Time-locked responses of individual PL-NAc (blue) and mTH-NAc (orange) neurons to task events. Data are presented as mean ± SEM across trials.

(B) Kernels representing the response to each of the task events for an example neuron, generated from the encoding model. The predicted GCaMP trace is the sum of the individual response kernels (see STAR Methods).

(C) Heatmap of response kernels generated from the encoding model for all PL-NAc neurons. Heatmap is ordered by the time of the peak response across all behavioral events (n = 278 neurons, n = 7 mice).

(D) Same as (C) except the heatmap of response kernels is from mTH-NAc neurons (n = 256 neurons, n = 9 mice).

(E) Heatmap of mean *Z*-scored GCaMP6f fluorescence from PL-NAc neurons aligned to the time of each event in the task. Neurons are ordered as in (C).

(F) Same as (E) for mTH-NAc neurons.

(G) Top row: fraction of neurons significantly modulated by action events in the PL-NAc (blue) and mTH-NAc (orange). For all action events, PL-NAc had a larger fraction of significantly modulated neurons than mTH-NAc. Bottom row: fraction of neurons in PL-NAc (blue) and mTH-NAc (orange) significantly modulated by stimulus events. Two out of three stimulus events had a larger fraction of significantly modulated neurons in mTH-NAc than in PL-NAc. Significance was determined using the linear model used to generate response kernels in (B) (STAR Methods).

(H) Top: a significantly larger fraction of event-modulated PL-NAc neurons encode at least one action event (p = 0.0004: two-proportion *Z* test comparing fraction of action-modulated PL-NAc and mTH-NAc neurons). Bottom: a significantly larger fraction of mTH-NAc neurons encode a stimulus event (p = 0.002: two-proportion *Z* test comparing fraction of stimulus-modulated neurons between PL-NAc and mTH-NAc). Asterisk denotes $p < 0.05$.

For (G) and (H), fractions are determined using the total number of neurons significantly modulated by at least one task event (n = 140 for PL-NAc, n = 90 for mTH-NAc).

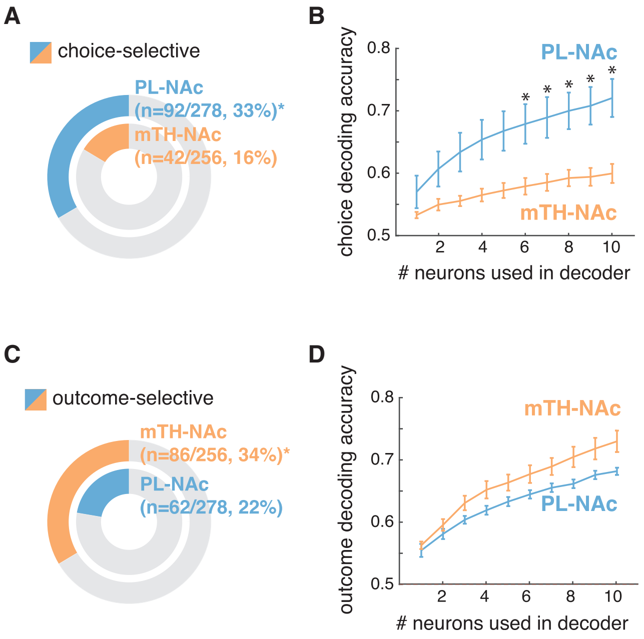

Figure 3. PL-NAc preferentially represents choice but not outcome relative to mTH-NAc

(A) Fraction of choice-selective neurons in PL-NAc (n = 92 out of 278 neurons, 7 mice) and mTH-NAc (n = 42 out of 256 neurons, 9 mice). A significantly larger fraction of PL-NAc neurons was choice-selective compared with mTH-NAc neurons ($p = 9.9 \times 10^{-6}$: two-proportion Z test).

(B) Choice decoding accuracy using randomly selected subsets of simultaneously imaged neurons around the lever press. The PL-NAc population more accurately decoded the choice of the trial compared with mTH-NAc (*$p < 0.05$, unpaired two-tailed t test, n = 9 PL-NAc and 6 mTH-NAc mice, peak decoding accuracy of 72% ± 3% for PL-NAc and 60% ± 2% for mTH-NAc).

(C) Fraction of outcome-selective neurons in mTH-NAc (n = 86 out of 256 neurons, 9 mice) and PL-NAc (n = 62 out of 278 neurons, 7 mice). A significantly larger fraction of mTH-NAc neurons were outcome-selective compared with PL-NAc neurons ($p = 0.004$: two-proportion Z test).

(D) Outcome decoding accuracy using neural activity after the time of the CS from randomly selected, simultaneously imaged neurons in mTH-NAc (orange, peak decoding accuracy: 73% ± 2%) and PL-NAc (blue, peak decoding accuracy: 68% ± 1%). $p > 0.05$, unpaired two-tailed t test.

Data in (B) and (D) are presented as mean ± SEM across mice; n = 6 PL-NAc mice and 9 mTH-NAc mice. In (A) and (C) the asterisk denotes $p < 0.05$, two-proportion Z test.

PL-NAc sequences (Figures S6D–S6F). The ridge-to-background ratio of both the PL-NAc and mTH-NAc sequences did not significantly change across either a block or recording session (Figures S8A–S8D).

A striking feature of these choice-selective sequences in PL-NAc was that they persisted for seconds after the choice, potentially providing a neural "bridge" between choice and outcome. To further quantify the timescale of choice encoding both within and across trials, we used activity from simultaneously imaged neurons at each time point in the trial to predict the mouse's choice (with a decoder based on a logistic regression using random combinations of ten simultaneously imaged neurons to predict choice). Choice on the current trial could be decoded above chance for ~7 s after the lever press, spanning the entire trial (including the time of reward delivery and consumption) as well as the beginning of the next trial (Figure 4E). Choice on the previous or subsequent trial was not represented as strongly as current-trial choice (Figure 4E; in all cases we corrected for cross-trial choice correlations with a weighted decoder, see STAR Methods) and choice from two trials back could not be decoded above chance at any time point (Figure S8E). We also examined the temporal extent of choice decoding in the mTH-NAc population (Figure S7E). Similar to PL-NAc, we observed that decoding persisted up to the start of the next trial. However, the peak decoding accuracy across all time points in the trial was lower in mTH-NAc (60% ± 0.1%) than in PL-NAc (73% ± 0.2%).

Synaptic plasticity or neural dynamics models incorporating choice-selective sequences in PL-NAc neurons can reproduce behavioral and neural recordings

We next used computational modeling to explain how a biologically realistic circuit incorporating the observed choice-selective sequences in PL-NAc neurons could solve the probabilistic reversal task. We constructed two models of the observed trial-by-trial changes in choice probabilities, one based on synaptic plasticity and one based on slow neural dynamics. Each model sought to explain two features of our data: first, how choices made at an earlier time (around the time of the nose poke, when choice-selective activity appears, Figures 4B and 4C) could be reinforced by rewards that occur at a later time, and, second, how this reinforcement could persist across multiple trials as suggested by our choice regressions (Figure 1E).

Synaptic plasticity model

The synaptic plasticity model mathematically implemented a temporal difference (TD) reinforcement learning algorithm by combining the recorded choice-selective sequential activity of PL-NAc neurons with the known connectivity of downstream structures (Figures 5A and 5B). The goal of TD learning is to learn to predict the sum of future rewards, or "value" (Dayan and Niv, 2008; O'Doherty et al., 2003; Sutton and Barto, 1998; Tsitsiklis and Van Roy, 1997). When this sum of expected future rewards changes, such as when an unexpected reward is received or an unexpected predictor of reward is experienced, a TD RPE occurs and adjusts the weights of reward-predicting inputs to reduce this error. The error signal in the TD algorithm closely resembles the RPE signal observed in ventral tegmental area (VTA) dopamine neurons (Parker et al., 2016; Schultz, 1998; Schultz et al., 1997), but how this signal is computed remains an open question.

In our model, the PL-NAc sequences (Figure 5C) enabled the calculation of the RPE in dopamine neurons which, in turn, reinforced those PL-NAc inputs that lead to better-than-predicted rewards. In more detail, the model took as inputs experimental, single-trial recordings of choice-selective, sequentially active PL neurons (Figure 5A, left; see STAR Methods). These inputs represented temporal basis functions $f_i(t)$ for computing the estimated value of making a left or right choice. These basis functions are weighted in the NAc by the strength w_i of the PL-NAc synaptic connection and summed together to create a (sign-inverted) representation of the estimated value, at time t, of making a left choice, $V_L(t)$, or right choice, $V_R(t)$. To create

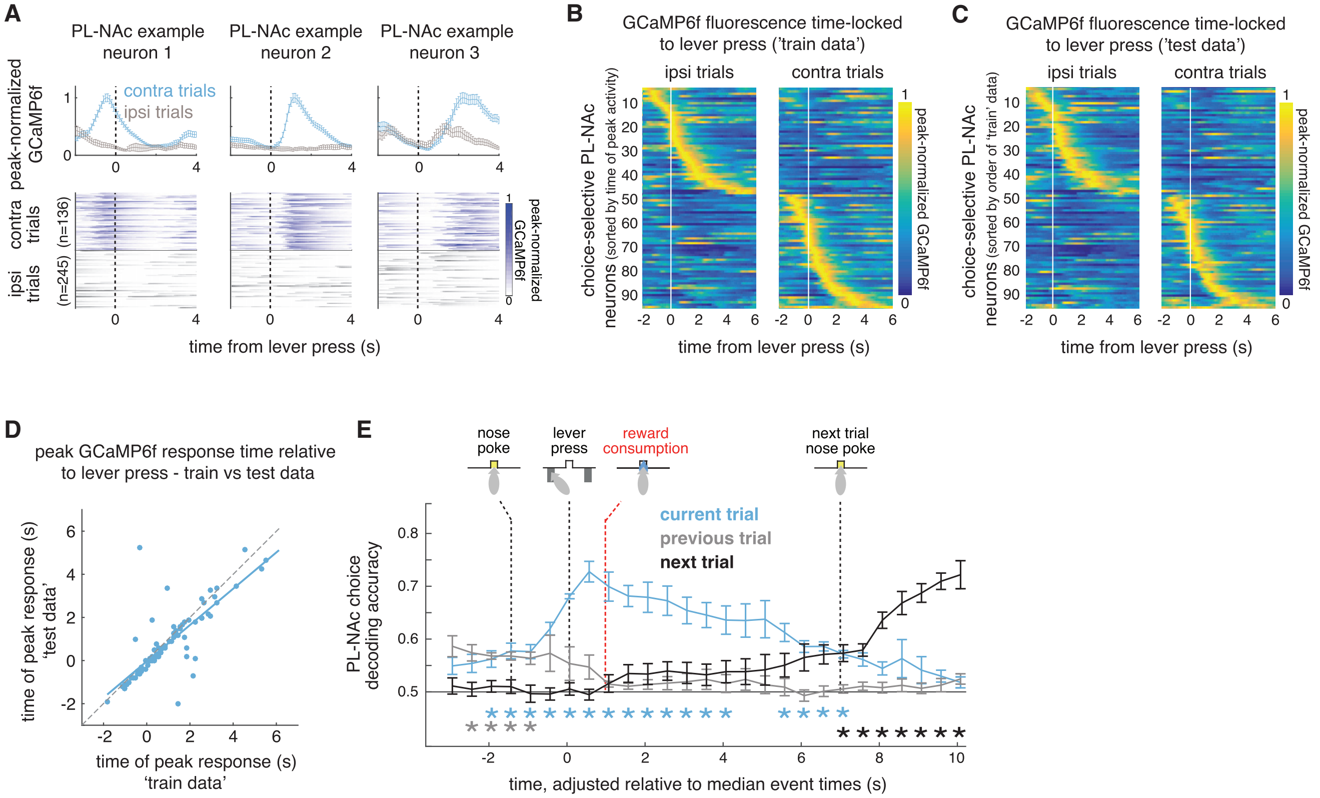

Figure 4. Choice-selective sequences in PL-NAc persist into the subsequent trial

(A) Top: average peak-normalized GCaMP6f fluorescence of three simultaneously imaged PL-NAc choice-selective neurons. Data are presented as mean ± SEM across trials. Bottom: heatmaps of GCaMP6f fluorescence across trials aligned to ipsilateral (blue) and contralateral (gray) press.

(B and C) Heatmaps showing sequential activation of choice-selective PL-NAc neurons (n = 92/278 neurons from 7 mice). Each row is a neuron's average GCaMP6f fluorescence time-locked to the ipsilateral (left column) and contralateral (right column) lever press, normalized by its peak average fluorescence. In (B) ("train data"), heatmap is average fluorescence from half of trials and ordered by the time of peak activity. In (C) ("test data"), the peak-normalized, time-locked GCaMP6f fluorescence from the other half of trials was plotted in the order from "train data" in (B).

(D) Correlation between time of peak activity using the "train" and "test" trials for choice-selective PL-NAc neurons in response to a contralateral or ipsilateral lever press ($R^2 = 0.80$, $p = 5.3 \times 10^{-22}$, n = 92 neurons).

(E) Average decoding accuracy of choice on the current (blue), previous (gray), and next (black) trial as a function of time-adjusted GCaMP6f fluorescence throughout the current trial from ten simultaneously imaged PL-NAc neurons. Data are presented as mean ± SEM across mice. Red dashed line indicates median onset of reward consumption. *$p < 0.01$, two-tailed, one-sample t test across mice comparing decoding accuracy to chance, n = 6 mice.

the RPE observed in dopamine neurons requires that the dopamine neuron population receive a fast, positive value signal $V(t)$ and a delayed negative value signal $V(t-\Delta)$, as well as a direct reward signal $r(t)$ (Figure 5B). In Figure 5A, the summation of NAc inputs and sign inversion occurs in the ventral pallidum (VP) (Kimura et al., 1996; Oorschot, 1996), so that the fast value signal is due to direct VP to VTA dopamine input. The delayed negative value signal to the dopamine population is due to a slower, disynaptic pathway that converges first upon the VTA γ-aminobutyric acid (GABA) neurons, so that these neurons encode a value signal as observed experimentally (Cohen et al., 2012). The temporal discounting factor γ is implemented through different strengths of the two pathways to the VTA dopamine neurons (Figure 5B). Other mathematically equivalent circuit architectures, including those involving other structures such as the lateral habenula (Li et al., 2019), are given in Figure S9. Learning is achieved through dopamine-dependent modification of the PL-NAc synaptic strengths. We assume that PL-NAc neuronal activity leads to an exponentially decaying synaptic "eligibility trace" (Gerstner et al., 2018; Sutton and Barto, 1998). The correlation of this presynaptically driven eligibility trace with dopamine input then drives learning (Figure 5B). Altogether, this circuit architecture (as well as those shown in Figure S9) realizes a TD learning algorithm for generating value representations in the NAc, providing a substrate for the selection of proper choice based on previous trial outcomes.

The synaptic plasticity model was able to correctly perform the task and recapitulate the mice's behavior. It achieved a comparable rate of reward (47.2% for the model, 47.6% for the mice) and exhibited similar alternation of choice following block reversals (Figures 5D and 5E; compare with Figures 1B and 1C;choice was based upon a probabilistic readout, at the start of the sequence, of the difference between right and left values plus a stay-with-previous choice bias [STAR Methods]) and similarly

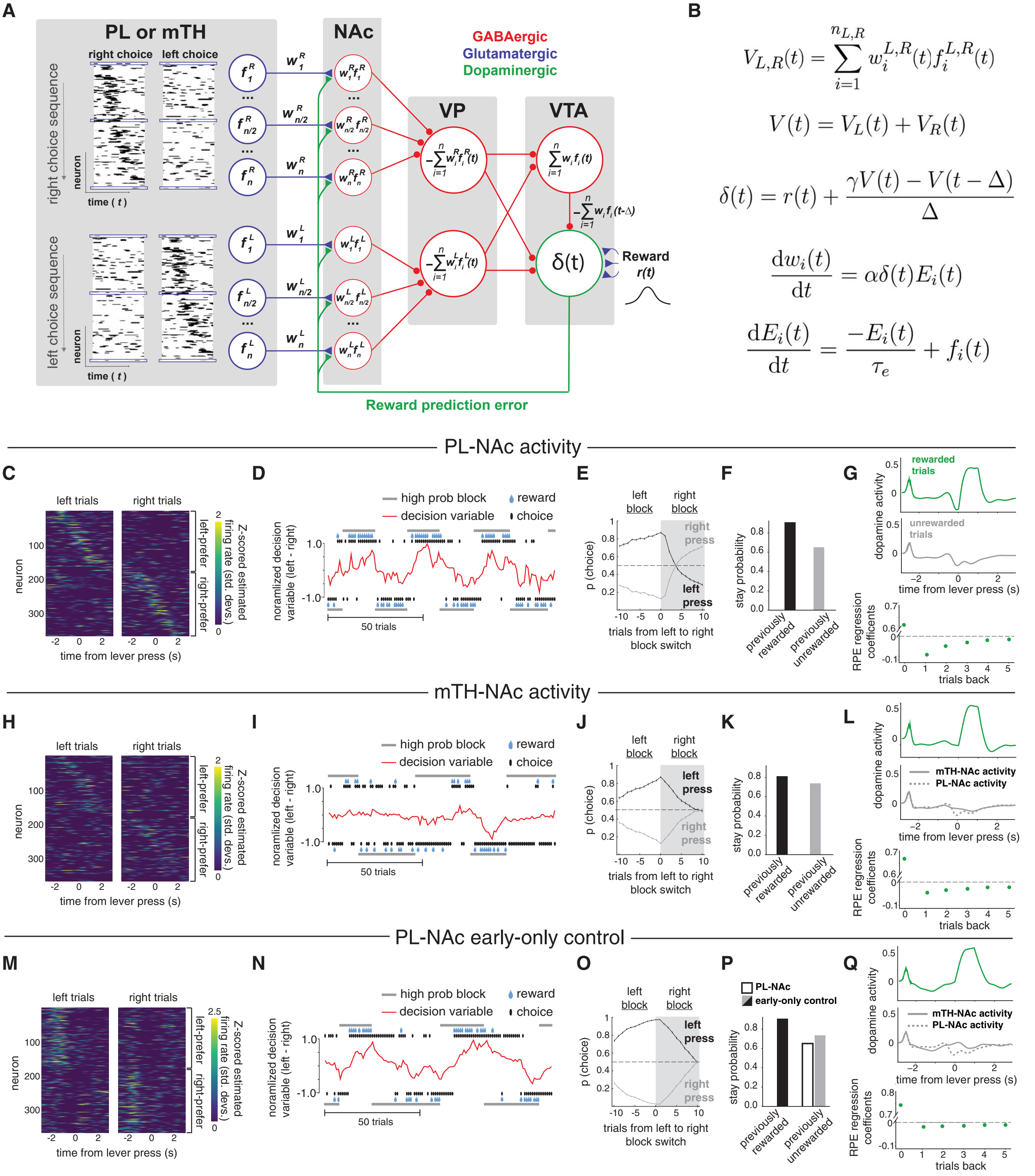

Figure 5. Choice-selective sequences recorded in PL-NAc, combined with known downstream connectivity, can implement a temporal difference (TD) learning model based on synaptic plasticity

(A) Schematic of circuit architecture used in the model. Model implementation used single-trial recorded PL-NAc or mTH-NAc responses as input. See results and STAR Methods for model details and Figure S9 for alternative, mathematically equivalent circuit architectures.

(B) Model equations. V: value; V_L, V_R: weighted sum of the n_L left-choice- or n_R right-choice-preferring NAc neuron activities f_i^L and f_i^R, respectively, with weights w_i^L or w_i^R; α: learning rate; τ_e: decay time constant for the PL-NAc synaptic eligibility trace $E(t)$; Δ: delay of the pathway through the VTA GABA interneuron; γ: discounting of value during time Δ.

(legend continued on next page)

higher stay probability following rewarded relative to unrewarded trials (Figure 5F; compare with Figure 1D).

Model neuron responses resembled those previously observed experimentally. The RPE signal within a trial showed characteristic positive response to rewarded outcomes and negative response to unrewarded outcomes (Figure 5G; compare with Figures S10A and S10B) and had similar dependence upon previous trial outcomes (Figure 5G, multiple linear regression similar to Bayer and Glimcher, 2005; Parker et al., 2016; Figures S10C and S10D). The VTA GABA interneuron had a sustained value signal, due to the converging input of the transient, sequential value signals from NAc/VP (Figure S11), replicating the sustained value signal in VTA GABA interneurons observed in monosynaptic inputs to VTA dopamine neurons (Cohen et al., 2012). Alternatively, the VP neurons shown in Figure 5A could project to a second set of VP neurons that functionally take the place of the VTA GABA interneurons (Figures S9A, S9C, and S9F), leading to sustained positive value encoding VP neurons as observed in VTA-projecting VP neurons (Tian et al., 2016).

We next ran the same model using single-trial activity from choice-selective mTH-NAc neurons instead of PL-NAc (Figure 5H). In line with the less consistent sequential choice-selective activity in mTH-NAc relative to PL-NAc (Figures 4 and S7), the correct value after a block switch was learned much more slowly within the NAc and VTA GABA neurons (Figures S11C and S11D), leading to correspondingly slow changes in choice probability (Figures 5I and 5J). As a result, choice probabilities were often out of sync with the current block, leading to overall reward rate near chance levels (38.7% reward rate, chance rate of 40%). Stay probabilities were inappropriately high following unrewarded trials (Figure 5K), reflecting reduced formation of an RPE and thus less negative modulation of dopamine signal at the time of expected reward (Figure 5L).

The choice-selective sequences in PL-NAc neurons were critical to model performance, as they allowed proper formation of an RPE at the time of reward receipt. This was verified by generating a control model that only included early-firing PL-NAc neurons (neurons active at the onset of the sequence when the model makes its choice) (Figure 5M). This “early-only control” model failed to quickly modulate lever value following block reversals (~10 trials to reverse following a block switch rather than ~3 trials for the full PL-NAc data; Figures 5N–5P). The inferior performance of this control model (model reward rate: 43.9%) reflected two factors. First, the early-only control model was unable to generate a well-timed RPE signal due to the absence of significant PL-NAc input activity at the time of reward. As a result, on unrewarded trials there was almost no negative reward-predictive dip in dopamine activity at the time of reward omission, unlike for the model with the full PL-NAc input activity (Figure 5Q). This lack of learning from unrewarded trials is evident in the stay probability plot (Figure 5P), which shows less modulation by unrewarded trials when controlling (by adjusting the model’s action-selection parameters) for the stay probability following rewarded trials. Second, unlike the sequential model, the RPE in the early-only control model could not propagate backward across successive trials, so single-trial learning (enabled by the eligibility trace) was the only mechanism available to bridge the gap in time between the firing of the early-firing decision neurons and an RPE occurring at the time of reward.

Neural dynamics model

The synaptic plasticity model described above requires fast, dopamine-mediated synaptic plasticity, on the timescale of a trial, to mediate behavior. Whether plasticity operates in the NAc on this timescale is unclear. We thus developed an alternative model (Figure 6A and STAR Methods) in which the across-trial updating of values and corresponding selection of actions is accomplished through the dynamics of a recurrent neural network rather than the dynamics of synaptic plasticity (Botvinick et al., 2019, 2020; Doshi-Velez and Konidaris, 2016; Song et al., 2017; Wang et al., 2018). The initial learning of the neural network’s synaptic weights is based on a reinforcement learning algorithm, which models slow initial task acquisition, but during task performance synaptic weights remain fixed and the dopamine RPE serves only to alter neural dynamics.

Similar to the synaptic plasticity model, single-trial, experimentally recorded PL-NAc activity was input to a (now recurrent) neural network that modeled NAc and other associated brain regions (the “critic network”) to calculate value. RPE was calculated in the dopamine neurons from the value signal using the same circuit architecture as the synaptic plasticity model. However, rather than reweighting PL-NAc synapses on the timescale of trials, the RPE was input to a second recurrent neural network that modeled dorsomedial striatum (DMS) and other associated brain regions (the “actor network;” Atallah et al., 2007; Lau and Glimcher, 2008; O’Doherty et al., 2004; Richard et al., 2016; Tsutsui et al., 2016). This actor network used the RPE input from the previous timestep, the action from the previous timestep, and a “temporal context” sequence that may arise from hippocampus or other cortical or subcortical areas (Akhlaghpour

(C) Heatmap of single-trial PL-NAc estimated firing rates input to the model.
(D) Behavior of the synaptic plasticity model for 120 example trials. The decision variable (red trace) and the choice of the model (black dots) follow the identity of the higher probability lever.
(E) Probability the model chooses left (black) and right (gray) following a left-to-right block reversal.
(F) Stay probability of the synaptic plasticity model following rewarded and unrewarded trials.
(G) Top: simulated VTA dopamine neuron activity averaged across rewarded (green) and unrewarded (gray) trials. Bottom: coefficients from a linear regression that uses outcome of the current and previous five trials to predict dopamine neuron activity following outcome feedback (STAR Methods).
(H–L) Same as (C) to (G), instead showing results from using estimated firing rates from mTH-NAc single-trial activity. The mTH-NAc model input generates worse performance than using PL-NAc input, with less and slower modulation of the decision variables, and weaker modulation of dopamine activity by previous trial outcomes. Dashed line in (L) shows results from PL-NAc model (same data as in G).
(M) Control model including only early-firing neurons active at the onset of the sequence, when the model makes the choice.
(N–Q) Same as (D) to (G), instead showing results from using the early-only control model. Open bar in (P) and dashed line in (Q) show results from PL-NAc model (same data as in F and G).

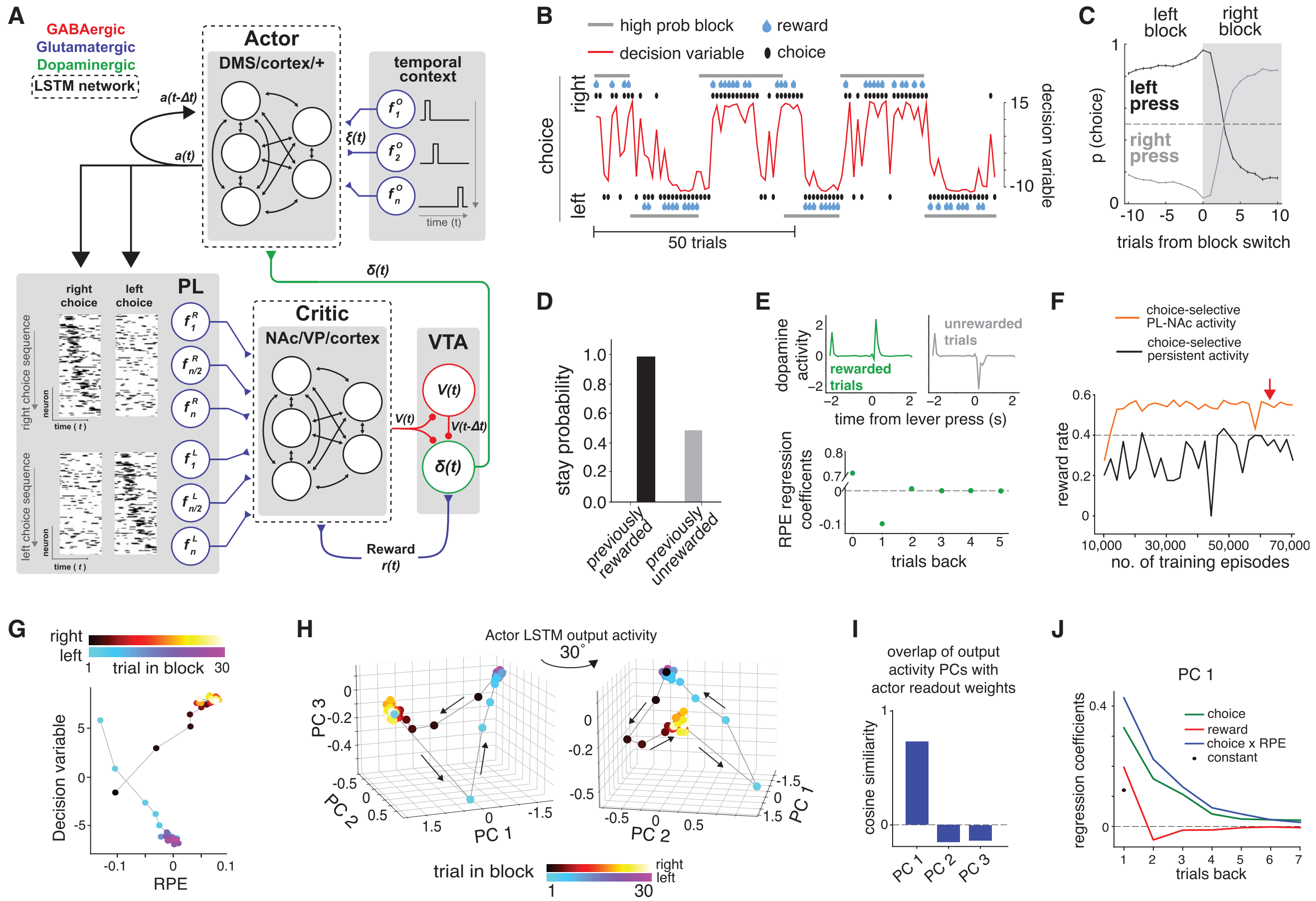

Figure 6. Neural dynamics model, with recorded choice-selective PL-NAc activity input to the critic, performs the task similarly to synaptic plasticity model

(A) Model schematic. See results and STAR Methods for details.

(B–E) Example behavior and dopamine activity from the neural dynamics model. Panel descriptions are the same as those for the synaptic plasticity model (Figures 5D–5G).

(F) Reward rate as a function of the number of training episodes for the model with recorded PL-NAc input to the critic (orange) and for a model with persistent choice-selective input to the critic (black). Red arrow indicates the training duration used to generate all other figure panels. Gray dashed line indicates chance reward rate of 0.4.

(G) Relationship between the decision variable used to select the choice on the next trial and the calculated RPE across right and left blocks. The RPE shown is an average of 0–2 s after lever press, averaged across blocks. The decision variable is also averaged across blocks.

(H) Evolution of the principal components of the output of the actor LSTM units across trials within a right and left block. The displayed activity is from the first time point in each trial (when the choice is made), averaged across blocks. The first three components accounted for 70.9%, 16.6%, and 6.4% of the total variance at this time point, respectively.

(I) Cosine of the angle between the actor network's readout weight vector and the vectors corresponding to the first three principal components (PCs). Network activity in the PC1 direction (but not PC2 or PC3) aligns with the network readout weights.

(J) Coefficients from a linear regression that uses choice on the previous trial (green), average RPE from 0–2 s after the lever press (red), and "choice × RPE" interaction (blue) from the previous seven trials to predict the amplitude of activity in PC1 on the current trial.

et al., 2016; Howard and Eichenbaum, 2013; Leon and Shadlen, 2003) to generate a decision variable corresponding to the probability of selecting one of three choices (left, right, or nothing) at any time. Selection of the left or right choice then triggered the onset of the corresponding PL-NAc activity sequence.

The neural dynamics model appropriately modulated choice following a reversal in the identity of the high-probability lever (Figure 6B–6D) and generated RPE signals in VTA dopamine neurons that resemble previous experimental recordings (Figures 6E and S10). By contrast, when we replaced the choice-selective sequences to the NAc by choice-selective persistent activity, the model failed to train within the same number of training episodes (Figure 6F). This suggests that temporal structure in this input is beneficial for efficient task learning.

To reveal how the model appropriately modulates its choices, we analyzed the evolution of the actor network's activity across trials (Figures 6G–6J). We found that the actor network's activity at the time of decision was low-dimensional, with the first three

CellPress
OPEN ACCESS

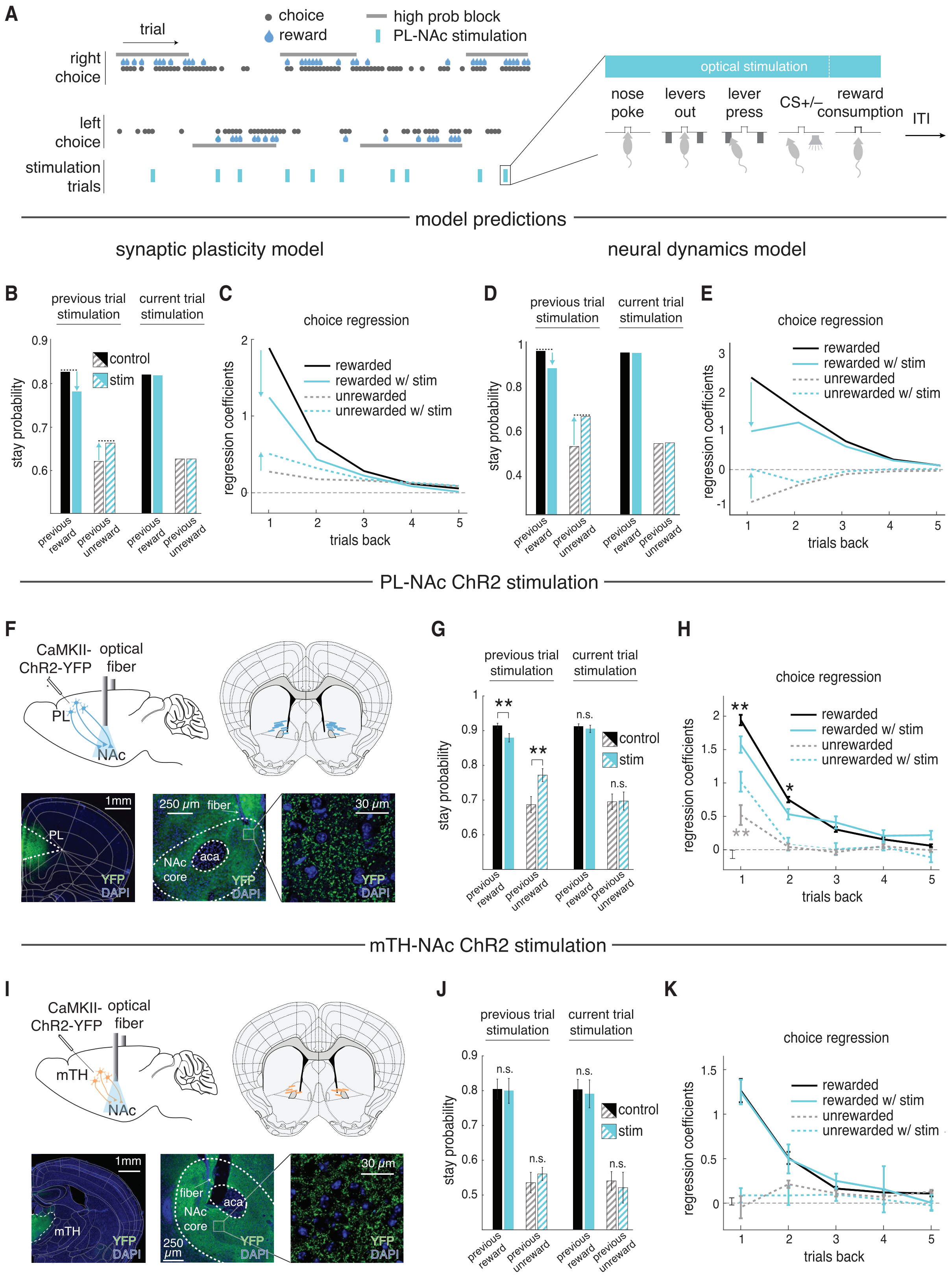

(legend on next page)

principal components explaining ∼94% of the variance. Given the symmetry in the block structure, the average RPE signal as a function of trial number is similar for the left and right blocks. However, the model should make opposite choices for left and right blocks, meaning that the actor network needs to respond oppositely to similar RPE inputs. Consistent with this, the decision variable for a given RPE was approximately opposite for left versus right blocks (Figure 6G). At a block reversal, for example from a left block to a right block, the network activity rapidly transitioned from the approximately steady-state representation of the left block (cluster of blue-purple points in Figure 6H) to the approximately steady-state representation of the right block (cluster of red-yellow points). Furthermore, the model learned to align the first principal component of activity along the direction of the network readout weights that determine the actor's choice $a(t)$ (Figure 6I). Thus, the actor learned to generate an explicit representation of the decision variable in the first principal component of its activity.

To solve the reversal learning task, the network needs to use its past history of choices and rewards to accumulate evidence for whether the current block is a left block or a right block. Rewarded left-side choices, or unrewarded right-side choices, represent evidence that the current block is a left block, while the converse represents evidence for a right block. In the synaptic plasticity model (Figure 5), new evidence (not accounted for by previous expectations) is accumulated in the PL-NAc synaptic weights as the product of the eligibility trace (which, due to the choice selectivity of the PL-NAc activity, represents the current choice) and the RPE. To analyze whether the actor network uses a similar accumulation of evidence to solve the task, we linearly regressed the first principal component of actor activity (PC1, which correlated strongly with the decision variable as described above) against the past history of choices and RPEs, which serve as inputs to the network, as well as the product of these ("choice × RPE"). PC1 most strongly depended upon the "choice × RPE" predictor, with coefficients that decayed on a timescale of approximately three trials, suggesting that the actor used a leaky accumulation of evidence over this timescale to solve the task (Figure 6J, blue trace). In addition, like the mice and the synaptic plasticity model, the neural dynamics model tended to stay with its previous choices, as evident from the positive coefficients for the previous choice regressors in Figure 6J (green trace). Thus, both the synaptic plasticity model and the neural dynamics model follow the same principle of accumulating evidence across trials to perform fast reversal learning in addition to having a tendency to repeat their previous choices.

Stimulation of PL-NAc (but not mTH-NAc) neurons decreases the effect of previous trial outcomes on subsequent choice in both the models and the mice

We next generated experimentally testable predictions from our models by examining the effect of disruption of the PL-NAc inputs on behavioral performance. To do so, we simulated optogenetic-like neural stimulation of this projection by replacing the PL-NAc sequential activity in the model with constant, choice-independent activity across 70% of the population on a subset of trials (Figure 7A). For both models, this generated a decrease in the probability of staying with the previously chosen lever following rewarded trials and an increase following unrewarded trials relative to unstimulated trials (Figures 7B and 7D). In other words, the effect of previous outcome on choice was reduced when PL-NAc activity was disrupted. This effect persists for multiple trials, as revealed by a logistic regression of current-trial choice on the history of previous rewarded and unrewarded choices with and without stimulation (Figures 7C and 7E; note that the negative coefficients for unrewarded trials in the neural dynamics model reflect that, unlike the synaptic plasticity model, this model does not include an explicit stay-with-previous choice bias). This reduced effect of outcome on choice arises because the stimulation disrupts the calculation of value. In the synaptic plasticity model, the stimulation of both left- and right-preferring PL-NAc neurons has two effects: first, it disrupts the RPE calculation by the circuit; second, it leads to dopamine indiscriminately adjusting the synaptic weights (i.e., value) of both the right and left PL-NAc synapses following rewarded or unrewarded outcomes. These weight changes then persist for multiple trials, leading to

Figure 7. Stimulation of PL-NAc neurons disrupts the influence of previous trial outcomes on subsequent choice in both the models and mice

(A) In the mice and models, PL-NAc neurons were stimulated for the whole trial on a random 10% of trials, disrupting the endogenous choice-selective sequential activity (see STAR Methods and Figure S13).

(B) Effect of stimulating the PL-NAc input on the previous (left) or current (right) trial in the synaptic plasticity model.

(C) Logistic choice regression showing dependence of the current choice on previously rewarded and unrewarded choices, with and without stimulation. Higher coefficients indicate a higher probability of staying with the previously chosen lever.

(D and E) Same as (B) and (C) for the neural dynamics model.

(F) Top left: schematic illustrating injection site in the PL (black needle) and optical fiber implant in the NAc core. Top right: location of optical fiber tips of PL-NAc ChR2 cohort (n = 14 mice) Bottom left: coronal section showing ChR2-YFP expression in PL. Bottom middle and right: ChR2-YFP expression in PL terminals in the NAc core.

(G) Similar to the models, PL-NAc ChR2 stimulation on the previous trial significantly reduced the mice's stay probability following a rewarded trial ($p = 0.002$) while increasing stay probability following an unrewarded trial ($p = 0.0005$). Stimulation on the current trial had no significant effect on stay probability following rewarded ($p = 0.62$) or unrewarded ($p = 0.91$) trials. All comparisons were paired two-tailed t tests, n = 14 mice.

(H) PL-NAc ChR2 stimulation decreased the weight of rewarded choices one and two trials back ($p = 0.002$: one trial back; $p = 0.023$: two trials back) and increased the weight of unrewarded choices one trial back ($p = 5.4 \times 10^{-6}$).

(I–K) Same as (F) to (H) for mTH-NAc ChR2 stimulation (n = 8 mice). mTH-NAc stimulation had no significant effect on stay probability following either rewarded ($p = 0.85$) or unrewarded choices ($p = 0.40$) on the previous trial back (J, paired t test, n = 8 mice) or multiple trials back (K, $p > 0.05$ for all trials back, one-sample t tests). Current-trial stimulation also had no effect following either rewarded ($p = 0.59$) or unrewarded ($p = 0.50$) choices.

**$p < 0.005$ and *$p < 0.05$ for one-sample two-tailed t tests.

decreased performance in subsequent trials. In the neural dynamics model, stimulation reduces behavioral performance on subsequent trials by disrupting the RPE signal that is transmitted to the actor, and this effect lasts for multiple trials because the actor network temporally accumulates RPE signals across multiple trials (Figure 6J). In both models, the choice behavior on the current trial is unaffected because choice is determined at the beginning of the trial, before the weights are updated (Figures 7B and 7D).

We tested these model predictions experimentally by performing an analogous optogenetic manipulation in mice (Figure 7F). In close agreement with our models, mice significantly decreased their stay probability following a rewarded trial that was paired with stimulation and significantly increased their stay probability following an unrewarded trial paired with stimulation (Figure 7G). Similar to the models, the effect of stimulation on the mouse's choice persisted for multiple trials. Mice had a significant decrease in their stay probability following PL-NAc stimulation on rewarded choices one and two trials back (Figure 7H). Also similar to the model, stimulation on the current trial had no significant effect on choice following either rewarded or unrewarded trials (Figure 7G).

In contrast to PL-NAc stimulation, but consistent with the relatively weak choice encoding in mTH-NAc compared with PL-NAc (Figures 3A and 3B) and weak trial-by-trial learning in our synaptic plasticity model (Figures 5H–5K), mTH-NAc stimulation (Figure 7I) had no significant effect on the mice's stay probability on the subsequent trial following either rewarded or unrewarded stimulation trials (Figure 7J). Similarly, inclusion of mTH-NAc stimulation in our choice regression model revealed no significant effect of stimulation on rewarded or unrewarded choices (Figure 7K). Additionally, there was no effect on the mice's stay probability for current-trial stimulation (Figure 7J).

For both PL-NAc and mTH-NAc stimulation, we observed an increase in the probability of mice abandoning the trials with stimulation compared with those trials without ($p = 0.0006$ for PL-NAc and $p = 0.032$ for mTH-NAc, paired two-tailed t test comparing percentage of abandoned trials on stimulated versus non-stimulated trials; 12.2% ± 2.5% and 22.1% ± 7.9% abandoned for PL-NAc and mTH-NAc stimulated trials, respectively; 0.9% ± 0.2% and 6.4% ± 3.1% for PL-NAc and mTH-NAc non-stimulated trials, respectively). Relatedly, we also found an increase in the latency to initiate a trial following either PL-NAc or mTH-NAc stimulation (Figures S12A–S12C). Together, these results suggest that this manipulation had some influence on the mouse's motivation to perform the task. However, unlike the stronger effect of PL-NAc versus mTH-NAc stimulation on subsequent choice behavior, this trial-abandonment effect was stronger for mTH-NAc than for PL-NAc.

To control for non-specific effects of optogenetic stimulation, we ran a control cohort of mice that received identical stimulation but did not express the opsin (Figures S12E and S12F). Stimulation had no significant effect on the mice's choice behavior (Figures S12D, S12G, and S12H) or probability of abandoning trials on stimulation versus control trials ($p = 0.38$, paired two-tailed t test comparing percentage of abandoned trials on stimulated with non-stimulated trials; 0.4% ± 0.08% for stimulated trials, 0.4% ± 0.01% for non-stimulated trials).

DISCUSSION

This work provides both experimental and computational insights into how the NAc and associated regions could contribute to reinforcement learning. Experimentally, we found that mTH-NAc neurons were preferentially modulated by a reward-predictive cue while PL-NAc neurons more strongly encoded actions (e.g., nose poke, lever press). In addition, PL-NAc neurons display choice-selective sequential activity which persists for several seconds after the lever-press action, beyond the time the mice receive reward feedback. Computationally, we demonstrate that the choice-selective and sequential nature of PL-NAc activity can contribute to performance of a choice task by implementing a circuit-based version of reinforcement learning based on either synaptic plasticity or neural dynamics. Furthermore, PL-NAc perturbations affect future but not current choice in both the models and mice, consistent with perturbation of the critic, not the actor.

Relationship to previous neural recordings in the NAc and associated regions

To our knowledge, a direct comparison, at cellular resolution, of activity across multiple glutamatergic inputs to the NAc has not previously been conducted. The preferential representations of actions relative to sensory stimuli in PL-NAc is somewhat surprising, given that previous studies have focused on sensory representations in this projection (Otis et al., 2017), and also given that the NAc is heavily implicated in Pavlovian conditioning (Day and Carelli, 2007; Day et al., 2006; Di Ciano et al., 2001; Parkinson et al., 1999; Roitman et al., 2005; Wan and Peoples, 2006).

On the other hand, there is extensive previous evidence of action correlates in prefrontal cortex (Cameron et al., 2019; Genovesio et al., 2006; Luk and Wallis, 2013; Siniscalchi et al., 2019; Sul et al., 2010), and NAc is implicated in operant conditioning in addition to Pavlovian conditioning (Atallah et al., 2007; Cardinal and Cheung, 2005; Collins et al., 2019; Hernandez et al., 2002; Kelley et al., 1997; Kim et al., 2009; Salamone et al., 1991). Our finding of sustained choice encoding in PL-NAc neurons is in agreement with previous work recording from medial prefrontal cortex neurons during a different reinforcement learning task (Maggi and Humphries, 2019; Maggi et al., 2018). Additionally, other papers have reported choice-selective sequences in other regions of cortex as well as in the hippocampus (Harvey et al., 2012; Pastalkova et al., 2008; Terada et al., 2017). In fact, given previous reports of choice-selective (or outcome-selective) sequences in multiple brain regions and species (Kawai et al., 2015; Kim et al., 2017; Long et al., 2010; Ölveczky et al., 2011; Picardo et al., 2016; Sakata et al., 2008), the relative absence of sequences in mTH-NAc neurons may be more surprising than the presence in PL-NAc.

Our observation of prolonged representation of the CS+ in mTH-NAc (Figures 2D and 2F) is in line with previous observations of pronounced and prolonged encoding of task-related stimuli in the primate thalamus during a Pavlovian conditioning task (Matsumoto et al., 2001). Together with our data, this suggests that the thalamus is contributing information about task-relevant stimuli to the striatum, which could potentially serve to

bridge the gap between a CS and unconditioned stimulus (US) in a Pavlovian trace conditioning task (Campus et al., 2019; Do-Monte et al., 2017; Otis et al., 2019; Zhu et al., 2018).

Implementation of reinforcement learning in models based on synaptic plasticity or neural dynamics

We presented two different classes of models that could solve the reversal learning task when provided with the choice-selective sequences observed in PL-NAc neurons as inputs. In our synaptic plasticity model, we show how these sequences may contribute to a neural implementation of TD learning by providing a temporal basis set that bridges the gap in time between actions and outcomes and enables the calculation of RPE in dopamine neurons. Other forms of neural dynamics, such as constant or slowly decaying persistent activity, can also maintain values across a delay period. However, creating a temporally precise RPE from such persistent activity is challenging if the persistent activity does not have sharp temporal features. Likewise, synaptic eligibility traces are another useful mechanism for bridging gaps in time, enabling earlier inputs to be reinforced by an RPE, but they do not provide the active input required to create the RPE itself.

A limitation of the synaptic plasticity model for producing the rapid reversals of behavior at block switches is that it requires a dopamine-dependent synaptic plasticity mechanism that operates on the timescale of trials (Figure 5). Whether dopamine-mediated synaptic plasticity operates on such fast timescales is not clear. Furthermore, model-free TD learning cannot take advantage of additional task-structure information such as the reward probabilities within a block (Collins and Cockburn, 2020; Doll et al., 2012; but see Figure S14 for challenges in identifying this ability within tasks like ours). These observations motivated the neural dynamics model in which, following initial slow-timescale learning of synaptic weights, the plasticity was turned off and trial-by-trial modulation of behavior was mediated by dopamine-dependent neural dynamics instead of synaptic plasticity (Figure 6; see related work by Botvinick et al., 2019, 2020; Doshi-Velez and Konidaris, 2016; Nagabandi et al., 2018; Song et al., 2017; Wang et al., 2018; Sæmundsson et al., 2018; Finn et al., 2017; Duan et al., 2016; Rakelly et al., 2019). Because the recurrent "critic" network dynamics can be trained to construct a temporally rich representation, the neural dynamics model has less need for precise temporal sequences in the PL-NAc inputs. However, we found that strictly eliminating the temporal structure of the PL-NAc input while preserving the choice selectivity made training of the network less efficient (Figure 6F), suggesting that having temporal structure in PL-NAc inputs facilitates the calculation of value.

Previous work in biological TD learning has used sequentially active neurons as the basis for learning in the context of sequential behaviors (Fee and Goldberg, 2011; Jin et al., 2009) and learning the timing of a CS-US relationship (Aggarwal et al., 2012; Carrillo-Reid et al., 2008; Gershman et al., 2014; Ponzi and Wickens, 2010). Likewise, our neural dynamics model was inspired by a previous meta-reinforcement learning model that was used to solve a reversal learning task (Wang et al., 2018). Here we extend these ideas in multiple important ways:

First, we link these theoretical ideas directly to data, by demonstrating that choice-selective sequential activity in the NAc is provided primarily by PL-NAc (as opposed to mTH-NAc) input neurons and that perturbation of the PL-NAc (but not mTH-NAc) projection disrupts action-outcome pairing consistent with model predictions. As such, our models provide a mechanistic explanation of a puzzling experimental finding: that optogenetic manipulation of PL-NAc neurons affects subsequent choices but not the choice on the stimulation trial itself, and that this stimulation creates oppositely directed effects following rewarded versus unrewarded trials.

Second, both of our models replicate numerous experimental findings in the circuitry downstream of PL-NAc. Each calculates an RPE signal in dopamine neurons (Bayer and Glimcher, 2005; Parker et al., 2016), generates conjunctive encoding of actions and outcomes (Kim et al., 2009, 2013), and calculates chosen value signals (Lau and Glimcher, 2008). Additionally, both models generate encoding of value by GABA interneurons (Cohen et al., 2012; Tian et al., 2016), which produces the temporally delayed, sign-inverted signals required for the calculation of a temporally differenced RPE (Figure 5A; see Aggarwal et al., 2012; Carrillo-Reid et al., 2008; Doya, 2002; Hazy et al., 2010; Ito and Doya, 2015; Joel et al., 2002; Pan et al., 2005; Suri and Schultz, 1998, 1999). Consistent with our models, electrical stimulation of VP generates both immediate inhibition of dopamine neurons and delayed excitation (Chen et al., 2019). Conceptually, the proposed temporal differencing by the VTA GABA interneuron is attractive in that it could provide a generalizable mechanism for calculating RPE: it could extend to any pathway that projects to both the dopamine and GABA neurons in the VTA (Beier et al., 2015) and that also receives a dopaminergic input that can modify synaptic weights.

Third, we showed that the fundamental operating principle of both models was similar: each temporally accumulates the correlation of previous choices with reward to determine the current-trial choice probability. In the synaptic plasticity model, this accumulation is done in the PL-NAc synaptic weights (Figure 5B). In the neural dynamics model, the accumulation is done in the low-dimensional neural dynamics of the actor network (Figure 6J). Future experiments that exploit these differences will need to be designed and executed to determine whether the brain more closely resembles the synaptic plasticity or neural dynamics model.

Limitations of the study

A limitation of this study is that we could not artificially recapitulate sequential firing to directly test its role in constructing value representations. Additionally, any artificial stimulation can have off-target and unintended consequences. Thus, further work directly investigating the causal role of PL-NAc sequences in reinforcement learning is needed. Neither of our models account for the influence of glutamatergic inputs to NAc from regions other than prelimbic cortex and the medial thalamus. In addition, our neural dynamics model used long short-term memory (LSTM) units, which should not be interpreted as single neurons but might model computations performed by larger populations. Finally, single-photon imaging limits the ability to resolve single z planes during imaging and,

thus, can make single neuron identification difficult. Future studies confirming our studies with other methods may be helpful.

STAR★METHODS

Detailed methods are provided in the online version of this paper and include the following:

- KEY RESOURCES TABLE
- RESOURCE AVAILABILITY
  - Lead contact
  - Materials availability
  - Data and code availability
- EXPERIMENTAL MODEL AND SUBJECT DETAILS
  - Mice
- METHOD DETAILS
  - Probabilistic reversal learning task
  - Cellular-resolution calcium imaging
  - Optogenetic stimulation of PL-NAc neurons
- QUANTIFICATION AND STATISTICAL ANALYSIS
  - Logistic choice regression
  - Encoding model to generate response kernels for behavioral events
  - Quantification of neural modulation to behavioral events
  - Neural decoders
  - Statistics
  - Synaptic plasticity model
  - Synaptic plasticity model description
  - Synaptic plasticity model implementation
  - Neural dynamics model
  - Neural dynamics model description
  - Neural dynamics model implementation
  - Cross-trial analysis of RPE in dopamine neurons
  - Simulation of model-free versus model-based task performance

SUPPLEMENTAL INFORMATION

Supplemental information can be found online at https://doi.org/10.1016/j.celrep.2022.110756.

ACKNOWLEDGMENTS

This work was supported by grants from NIH R01 DA047869 (I.B.W.), U19 NS104648 (M.S.G., I.B.W.), F32 MH112320 (J.C.), ARO W911NF1710554 (I.B.W.), Brain Research Foundation (I.B.W.), Simons Collaboration on the Global Brain (M.S.G., I.B.W.), and the New York Stem Cell Foundation (I.B.W.). I.B.W. is an NYSCF—Robertson Investigator.

AUTHOR CONTRIBUTIONS

N.F.P., J.C., L.M.H., A.Z., and M.M. performed the experiments under the supervision of I.B.W.; N.F.P., A.B., J.C., and B.E. analyzed the behavioral and neural data; A.B. performed the modeling work under the supervision of M.S.G.; N.F.P., A.B., J.C., M.S.G., and I.B.W. wrote the paper.

DECLARATION OF INTERESTS

The authors declare no competing interests.

Received: July 28, 2019
Revised: February 18, 2022
Accepted: April 7, 2022
Published: May 17, 2022

REFERENCES

Aggarwal, M., Hyland, B.I., and Wickens, J.R. (2012). Neural control of dopamine neurotransmission: implications for reinforcement learning. Eur. J. Neurosci. *35*, 1115–1123.

Akhlaghpour, H., Wiskerke, J., Choi, J.Y., Taliaferro, J.P., Au, J., and Witten, I.B. (2016). Dissociated sequential activity and stimulus encoding in the dorsomedial striatum during spatial working memory. Elife *5*, e19507.

Apicella, P., Ljungberg, T., Scarnati, E., and Schultz, W. (1991). Responses to reward in monkey dorsal and ventral striatum. Exp. Brain Res. *85*, 491–500.

Asaad, W.F., Lauro, P.M., Perge, J.A., and Eskandar, E.N. (2017). Prefrontal neurons encode a solution to the credit-assignment problem. J. Neurosci. *37*, 6995–7007.

Atallah, H.E., Lopez-Paniagua, D., Rudy, J.W., and O'Reilly, R.C. (2007). Separate neural substrates for skill learning and performance in the ventral and dorsal striatum. Nat. Neurosci. *10*, 126–131.

Bayer, H.M., and Glimcher, P.W. (2005). Midbrain dopamine neurons encode a quantitative reward prediction error signal. Neuron *47*, 129–141.

Beier, K.T., Steinberg, E.E., DeLoach, K.E., Xie, S., Miyamichi, K., Schwarz, L., Gao, X.J., Kremer, E.J., Malenka, R.C., and Luo, L. (2015). Circuit architecture of VTA dopamine neurons revealed by systematic input-output mapping. Cell *162*, 622–634.

Botvinick, M., Ritter, S., Wang, J.X., Kurth-Nelson, Z., Blundell, C., and Hassabis, D. (2019). Reinforcement learning, fast and slow. Trends Cogn. Sci. *23*, 408–422.

Botvinick, M., Wang, J.X., Dabney, W., Miller, K.J., and Kurth-Nelson, Z. (2020). Deep reinforcement learning and its neuroscientific implications. Neuron *107*, 603–616.

Brog, J.S., Salyapongse, A., Deutch, A.Y., and Zahm, D.S. (1993). The patterns of afferent innervation of the core and shell in the "accumbens" part of the rat ventral striatum: immunohistochemical detection of retrogradely transported fluoro-gold. J. Comp. Neurol. *338*, 255–278.

Bromberg-Martin, E.S., Matsumoto, M., Hong, S., and Hikosaka, O. (2010). A pallidus-habenula-dopamine pathway signals inferred stimulus values. J. Neurophysiol. *104*, 1068–1076.

Cador, M., Robbins, T.W., and Everitt, B.J. (1989). Involvement of the amygdala in stimulus-reward associations: interaction with the ventral striatum. Neuroscience *30*, 77–86.

Cameron, C.M., Murugan, M., Choi, J.Y., Engel, E.A., and Witten, I.B. (2019). Increased cocaine motivation is associated with degraded spatial and temporal representations in IL-NAc neurons. Neuron *103*, 80–91.e7.

Campus, P., Covelo, I.R., Kim, Y., Parsegian, A., Kuhn, B.N., Lopez, S.A., Neumaier, J.F., Ferguson, S.M., Solberg Woods, L.C., Sarter, M., et al. (2019). The paraventricular thalamus is a critical mediator of top-down control of cue-motivated behavior in rats. Elife *8*, e49041.

Cardinal, R.N., and Cheung, T.H.C. (2005). Nucleus accumbens core lesions retard instrumental learning and performance with delayed reinforcement in the rat. BMC. Neurosci. *6*, 9.

Carelli, R.M., King, V.C., Hampson, R.E., and Deadwyler, S.A. (1993). Firing patterns of nucleus accumbens neurons during cocaine self-administration in rats. Brain. Res. *626*, 14–22.

Carrillo-Reid, L., Tecuapetla, F., Tapia, D., Hernández-Cruz, A., Galarraga, E., Drucker-Colin, R., and Bargas, J. (2008). Encoding network states by striatal cell assemblies. J. Neurophysiol. *99*, 1435–1450.

Chen, R., Puzerey, P.A., Roeser, A.C., Riccelli, T.E., Podury, A., Maher, K., Farhang, A.R., and Goldberg, J.H. (2019). Songbird ventral pallidum sends diverse performance error signals to dopaminergic midbrain. Neuron *103*, 266–276.e4.

Cohen, J.Y., Haesler, S., Vong, L., Lowell, B.B., and Uchida, N. (2012). Neuron-type-specific signals for reward and punishment in the ventral tegmental area. Nature *482*, 85–88.

Collins, A.G.E., and Cockburn, J. (2020). Beyond dichotomies in reinforcement learning. Nat. Rev. Neurosci. *21*, 576–586.

Collins, A.L., Aitken, T.J., Huang, I.-W., Shieh, C., Greenfield, V.Y., Monbouquette, H.G., Ostlund, S.B., and Wassum, K.M. (2019). Nucleus accumbens cholinergic interneurons oppose cue-motivated behavior. Biol. Psychiatry *86*, 388–396.

Cox, J., and Witten, I.B. (2019). Striatal circuits for reward learning and decision-making. Nat. Rev. Neurosci. *20*, 482–494.

Day, J.J., and Carelli, R.M. (2007). The nucleus accumbens and Pavlovian reward learning. Neuroscientist *13*, 148–159.

Day, J.J., Wheeler, R.A., Roitman, M.F., and Carelli, R.M. (2006). Nucleus accumbens neurons encode Pavlovian approach behaviors: evidence from an autoshaping paradigm. Eur. J. Neurosci. *23*, 1341–1351.

Dayan, P., and Niv, Y. (2008). Reinforcement learning: the good, the bad and the ugly. Curr. Opin. Neurobiol. *18*, 185–196.

Di Ciano, P., Cardinal, R.N., Cowell, R.A., Little, S.J., and Everitt, B.J. (2001). Differential involvement of NMDA, AMPA/kainate, and dopamine receptors in the nucleus accumbens core in the acquisition and performance of pavlovian approach behavior. J. Neurosci. *21*, 9471–9477.

Doll, B.B., Simon, D.A., and Daw, N.D. (2012). The ubiquity of model-based reinforcement learning. Curr. Opin. Neurobiol. *22*, 1075–1081.

Do-Monte, F.H., Minier-Toribio, A., Quiñones-Laracuente, K., Medina-Colón, E.M., and Quirk, G.J. (2017). Thalamic regulation of sucrose seeking during unexpected reward omission. Neuron *94*, 388–400.e4.

Doshi-Velez, F., and Konidaris, G. (2016). Hidden parameter markov decision processes: a semiparametric regression approach for discovering latent task parametrizations. IJCAI *2016*, 1432–1440.

Doya, K. (2002). Metalearning and neuromodulation. Neural. Netw. *15*, 495–506.

Duan, Y., Schulman, J., Chen, X., Bartlett, P.L., Sutskeve, I., and Abbeel, P. (2016). RLˆ2: Fast reinforcement learning via slow reinforcement learning. Preprint at arXiv, 1611.02779. https://doi.org/10.48550/arXiv.1611.02779.

Engelhard, B., Finkelstein, J., Cox, J., Fleming, W., Jang, H.J., Ornelas, S., Koay, S.A., Thiberge, S.Y., Daw, N.D., Tank, D.W., et al. (2019). Specialized coding of sensory, motor and cognitive variables in VTA dopamine neurons. Nature *570*, 509–513.

Everitt, B.J., Morris, K.A., O'Brien, A., and Robbins, T.W. (1991). The basolateral amygdala-ventral striatal system and conditioned place preference: further evidence of limbic-striatal interactions underlying reward-related processes. Neuroscience *42*, 1–18.

Fee, M.S., and Goldberg, J.H. (2011). A hypothesis for basal ganglia-dependent reinforcement learning in the songbird. Neuroscience *198*, 152–170.

Finn, C., Abbeel, P., and Levine, S. (2017). RLˆ2: model-agnostic meta-learning for fast adaptation of deep networks. Preprint at arXiv, 1703.03400. https://doi.org/10.48550/arXiv.1703.03400.

Fisher, S.D., Robertson, P.B., Black, M.J., Redgrave, P., Sagar, M.A., Abraham, W.C., and Reynolds, J.N.J. (2017). Reinforcement determines the timing dependence of corticostriatal synaptic plasticity in vivo. Nat. Commun. *8*, 334.

Fürth, D., Vaissière, T., Tzortzi, O., Xuan, Y., Märtin, A., Lazaridis, I., Spigolon, G., Fisone, G., Tomer, R., Deisseroth, K., et al. (2018). An interactive framework for whole-brain maps at cellular resolution. Nat. Neurosci. *21*, 139–149.

Genovesio, A., Brasted, P.J., and Wise, S.P. (2006). Representation of future and previous spatial goals by separate neural populations in prefrontal cortex. J. Neurosci. *26*, 7305–7316.

Gerfen, C.R., and Surmeier, D.J. (2011). Modulation of striatal projection systems by dopamine. Annu. Rev. Neurosci. *34*, 441–466.

Gersch, T.M., Foley, N.C., Eisenberg, I., and Gottlieb, J. (2014). Neural correlates of temporal credit assignment in the parietal lobe. PLoS One *9*, e88725.

Gershman, S.J., Moustafa, A.A., and Ludvig, E.A. (2014). Time representation in reinforcement learning models of the basal ganglia. Front. Comput. Neurosci. *7*, 194.

Gerstner, W., Lehmann, M., Liakoni, V., Corneil, D., and Brea, J. (2018). Eligibility traces and plasticity on behavioral time scales: experimental support of NeoHebbian three-factor learning rules. Front. Neural Circuits *12*, 53.

Groenewegen, H.J., Becker, N.E., and Lohman, A.H. (1980). Subcortical afferents of the nucleus accumbens septi in the cat, studied with retrograde axonal transport of horseradish peroxidase and bisbenzimid. Neuroscience *5*, 1903–1916.

Hahnloser, R.H.R., Kozhevnikov, A.A., and Fee, M.S. (2002). An ultra-sparse code underliesthe generation of neural sequences in a songbird. Nature *419*, 65–70.

Harvey, C.D., Coen, P., and Tank, D.W. (2012). Choice-specific sequences in parietal cortex during a virtual-navigation decision task. Nature *484*, 62–68.

Hazy, T.E., Frank, M.J., and O'Reilly, R.C. (2010). Neural mechanisms of acquired phasic dopamine responses in learning. Neurosci. Biobehav. Rev. *34*, 701–720.

Hernandez, P.J., Sadeghian, K., and Kelley, A.E. (2002). Early consolidation of instrumental learning requires protein synthesis in the nucleus accumbens. Nat. Neurosci. *5*, 1327–1331.

Hinton, G., Srivastava, N., and Swersky, K. (2012). Neural networks for machine learning lecture 6A overview of mini-batch gradient descent. https://www.cs.toronto.edu/∼tijmen/csc321/slides/lecture_slides_lec6.pdf.

Howard, M.W., and Eichenbaum, H. (2013). The hippocampus, time, and memory across scales. J. Exp. Psychol. Gen. *142*, 1211–1230.

Hunnicutt, B.J., Jongbloets, B.C., Birdsong, W.T., Gertz, K.J., Zhong, H., and Mao, T. (2016). A comprehensive excitatory input map of the striatum reveals novel functional organization. Elife *5*, e19103.

Ito, M., and Doya, K. (2015). Parallel representation of value-based and finite state-based strategies in the ventral and dorsal striatum. PLoS Comput. Biol. *11*, e1004540.

Jin, D.Z., Fujii, N., and Graybiel, A.M. (2009). Neural representation of time in cortico-basal ganglia circuits. Proc. Natl. Acad. Sci. U S A *106*, 19156–19161.

Joel, D., Niv, Y., and Ruppin, E. (2002). Actor–critic models of the basal ganglia: new anatomical and computational perspectives. Neural Netw. *15*, 535–547.

Kalivas, P.W., Churchill, L., and Klitenick, M.A. (1993). GABA and enkephalin projection from the nucleus accumbens and ventral pallidum to the ventral tegmental area. Neuroscience *57*, 1047–1060.

Kawai, T., Yamada, H., Sato, N., Takada, M., and Matsumoto, M. (2015). Roles of the lateral habenula and anterior cingulate cortex in negative outcome monitoring and behavioral adjustment in nonhuman primates. Neuron *88*, 792–804.

Kelley, A.E., Smith-Roe, S.L., and Holahan, M.R. (1997). Response-reinforcement learning is dependent on N-methyl-D-aspartate receptor activation in the nucleus accumbens core. Proc. Natl. Acad. Sci. U S A *94*, 12174–12179.

Kim, H., Sul, J.H., Huh, N., Lee, D., and Jung, M.W. (2009). Role of striatum in updating values of chosen actions. J. Neurosci. *29*, 14701–14712.

Kim, H., Lee, D., and Jung, M.W. (2013). Signals for previous goal choice persist in the dorsomedial, but not dorsolateral striatum of rats. J. Neurosci. *33*, 52–63.

Kim, C.K., Ye, L., Jennings, J.H., Pichamoorthy, N., Tang, D.D., Yoo, A.-C.W., Ramakrishnan, C., and Deisseroth, K. (2017). Molecular and circuit-dynamical identification of top-down neural mechanisms for restraint of reward seeking. Cell *170*, 1013–1027.e14.

Kimura, M., Kato, M., Shimazaki, H., Watanabe, K., and Matsumoto, N. (1996). Neural information transferred from the putamen to the globus pallidus during learned movement in the monkey. J. Neurophysiol. *76*, 3771–3786.

Kondo, M., Kobayashi, K., Ohkura, M., Nakai, J., and Matsuzaki, M. (2017). Two-photon calcium imaging of the medial prefrontal cortex and hippocampus without cortical invasion. Elife *6*, e26839.

CellPress
OPEN ACCESS

Kozhevnikov, A.A., and Fee, M.S. (2007). Singing-related activity of identified HVC neurons in the zebra finch. J. Neurophysiol. *97*, 4271–4283.

Krumin, M., Lee, J.J., Harris, K.D., and Carandini, M. (2018). Decision and navigation in mouse parietal cortex. Elife *7*, e42583.

Lau, B., and Glimcher, P.W. (2008). Value representations in the primate striatum during matching behavior. Neuron *58*, 451–463.

Lee, R.S., Mattar, M.G., Parker, N.F., Witten, I.B., and Daw, N.D. (2019). Reward prediction error does not explain movement selectivity in DMS-projecting dopamine neurons. Elife *8*, e42992.

Leon, M.I., and Shadlen, M.N. (2003). Representation of time by neurons in the posterior parietal cortex of the macaque. Neuron *38*, 317–327.

Li, H., Vento, P.J., Parrilla-Carrero, J., Pullmann, D., Chao, Y.S., Eid, M., and Jhou, T.C. (2019). Three rostromedial tegmental afferents drive triply dissociable aspects of punishment learning and aversive valence encoding. Neuron *104*, 987–999.e4.

Long, M.A., Jin, D.Z., and Fee, M.S. (2010). Support for a synaptic chain model of neuronal sequence generation. Nature *468*, 394–399.

Lovett-Barron, M., Chen, R., Bradbury, S., Andalman, A.S., Wagle, M., Guo, S., and Deisseroth, K. (2019). Multiple overlapping hypothalamus-brainstem circuits drive rapid threat avoidance. Preprint at bioRxiv. https://doi.org/10.1101/745075.

Luk, C.-H., and Wallis, J.D. (2013). Choice coding in frontal cortex during stimulus-guided or action-guided decision-making. J. Neurosci. *33*, 1864–1871.

Maggi, S., and Humphries, M.D. (2019). Independent population coding of the present and the past in prefrontal cortex during learning. Preprint at bioRxiv. https://doi.org/10.1101/668962.

Maggi, S., Peyrache, A., and Humphries, M.D. (2018). An ensemble code in medial prefrontal cortex links prior events to outcomes during learning. Nat. Commun. *9*, 2204.

Matsumoto, M., and Hikosaka, O. (2009). Two types of dopamine neuron distinctly convey positive and negative motivational signals. Nature *459*, 837–841.

Matsumoto, N., Minamimoto, T., Graybiel, A.M., and Kimura, M. (2001). Neurons in the thalamic CM-pf complex supply striatal neurons with information about behaviorally significant sensory events. J. Neurophysiol. *85*, 960–976.

Mnih, V., Badia, A.P., Mirza, M., Graves, A., Lillicrap, T., Harley, T., Silver, D., and Kavukcuoglu, K. (2016). Asynchronous methods for deep reinforcement learning. International conference on machine learning, 1928–1937.

Montague, P.R., Dayan, P., and Sejnowski, T.J. (1996). A framework for mesencephalic dopamine systems based on predictive Hebbian learning. J. Neurosci. *16*, 1936–1947.

Musall, S., Kaufman, M.T., Juavinett, A.L., Gluf, S., and Churchland, A.K. (2019). Single-trial neural dynamics are dominated by richly varied movements. Nat. Neurosci. *22*, 1677–1686.

Nagabandi, A., Kahn, G., Fearing, R.S., and Levine, S. (2018). Neural network dynamics for model-based deep reinforcement learning with model-free fine-tuning. In 2018 IEEE International Conference on Robotics and Automation (ICRA).

O'Doherty, J., Dayan, P., Schultz, J., Deichmann, R., Friston, K., and Dolan, R.J. (2004). Dissociable roles of ventral and dorsal striatum in instrumental conditioning. Science *304*, 452–454.

O'Doherty, J.P., Dayan, P., Friston, K., Critchley, H., and Dolan, R.J. (2003). Temporal difference models and reward-related learning in the human brain. Neuron *38*, 329–337.

Ölveczky, B.P., Otchy, T.M., Goldberg, J.H., Aronov, D., and Fee, M.S. (2011). Changes in the neural control of a complex motor sequence during learning. J. Neurophysiol. *106*, 386–397.

Oorschot, D.E. (1996). Total number of neurons in the neostriatal, pallidal, subthalamic, and substantia nigral nuclei of the rat basal ganglia: a stereological study using the cavalieri and optical disector methods. J. Comp. Neurol. *366*, 580–599.

Otis, J.M., Namboodiri, V.M.K., Matan, A.M., Voets, E.S., Mohorn, E.P., Kosyk, O., McHenry, J.A., Robinson, J.E., Resendez, S.L., Rossi, M.A., et al. (2017). Prefrontal cortex output circuits guide reward seeking through divergent cue encoding. Nature *543*, 103–107.

Otis, J.M., Zhu, M., Namboodiri, V.M.K., Cook, C.A., Kosyk, O., Matan, A.M., Ying, R., Hashikawa, Y., Hashikawa, K., Trujillo-Pisanty, I., et al. (2019). Paraventricular thalamus projection neurons integrate cortical and hypothalamic signals for cue-reward processing. Neuron *103*, 277–290.e6.

Pan, W.-X., Schmidt, R., Wickens, J.R., and Hyland, B.I. (2005). Dopamine cells respond to predicted events during classical conditioning: evidence for eligibility traces in the reward-learning network. J. Neurosci. *25*, 6235–6242.

Park, I.M., Meister, M.L.R., Huk, A.C., and Pillow, J.W. (2014). Encoding and decoding in parietal cortex during sensorimotor decision-making. Nat. Neurosci. *17*, 1395–1403.

Parker, N.F., Cameron, C.M., Taliaferro, J.P., Lee, J., Choi, J.Y., Davidson, T.J., Daw, N.D., and Witten, I.B. (2016). Reward and choice encoding in terminals of midbrain dopamine neurons depends on striatal target. Nat. Neurosci. *19*, 845–854.

Parkinson, J.A., Olmstead, M.C., Burns, L.H., Robbins, T.W., and Everitt, B.J. (1999). Dissociation in effects of lesions of the nucleus accumbens core and shell on appetitive pavlovian approach behavior and the potentiation of conditioned reinforcement and locomotor activity by D-amphetamine. J. Neurosci. *19*, 2401–2411.

Pastalkova, E., Itskov, V., Amarasingham, A., and Buzsáki, G. (2008). Internally generated cell assembly sequences in the rat hippocampus. Science *321*, 1322–1327.

Paxinos, G., and Franklin, K.B.J. (2004). The Mouse Brain in Stereotaxic Coordinates (Gulf Professional Publishing).

Phillips, G.D., Le Noury, J., Wolterink, G., Donselaar-Wolterink, I., Robbins, T.W., and Everitt, B.J. (1993). Cholecystokinin-dopamine interactions within the nucleus accumbens in the control over behaviour by conditioned reinforcement. Behav. Brain. Res. *55*, 223–231.

Phillips, G.D., Robbins, T.W., and Everitt, B.J. (1994). Mesoaccumbens dopamine-opiate interactions in the control over behaviour by a conditioned reinforcer. Psychopharmacology *114*, 345–359.

Phillipson, O.T., and Griffiths, A.C. (1985). The topographic order of inputs to nucleus accumbens in the rat. Neuroscience *16*, 275–296.

Picardo, M.A., Merel, J., Katlowitz, K.A., Vallentin, D., Okobi, D.E., Benezra, S.E., Clary, R.C., Pnevmatikakis, E.A., Paninski, L., and Long, M.A. (2016). Population-level representation of a temporal sequence underlying song production in the zebra finch. Neuron *90*, 866–876.

Pinto, L., and Dan, Y. (2015). Cell-type-specific activity in prefrontal cortex during goal-directed behavior. Neuron *87*, 437–450.

Pnevmatikakis, E.A., and Giovannucci, A. (2017). NoRMCorre: an online algorithm for piecewise rigid motion correction of calcium imaging data. J. Neurosci. Methods. *291*, 83–94.

Ponzi, A., and Wickens, J. (2010). Sequentially switching cell assemblies in random inhibitory networks of spiking neurons in the striatum. J. Neurosci. *30*, 5894–5911.

Poulin, J.-F., Caronia, G., Hofer, C., Cui, Q., Helm, B., Ramakrishnan, C., Chan, C.S., Dombeck, D.A., Deisseroth, K., and Awatramani, R. (2018). Mapping projections of molecularly defined dopamine neuron subtypes using intersectional genetic approaches. Nat. Neurosci. *21*, 1260–1271.

Rakelly, K., Zhou, A., Quillen, D., Finn, D., and Levine, D. (2019). Efficient off-policy meta-reinforcement learning via probabilistic context variables. Preprint at arXiv. https://doi.org/10.48550/arXiv.1903.08254.

Reed, S.J., Lafferty, C.K., Mendoza, J.A., Yang, A.K., Davidson, T.J., Grosenick, L., Deisseroth, K., and Britt, J.P. (2018). Coordinated reductions in excitatory input to the nucleus accumbens underlie food consumption. Neuron *99*, 1260–1273.e4.

Reynolds, J.N.J., and Wickens, J.R. (2002). Dopamine-dependent plasticity of corticostriatal synapses. Neural Netw. *15*, 507–521.

Richard, J.M., Ambroggi, F., Janak, P.H., and Fields, H.L. (2016). Ventral pallidum neurons encode incentive value and promote cue-elicited instrumental actions. Neuron *90*, 1165–1173.

Robbins, T.W., Cador, M., Taylor, J.R., and Everitt, B.J. (1989). Limbic-striatal interactions in reward-related processes. Neurosci. Biobehav. Rev. *13*, 155–162.

Roitman, M.F., Wheeler, R.A., and Carelli, R.M. (2005). Nucleus accumbens neurons are innately tuned for rewarding and aversive taste stimuli, encode their predictors, and are linked to motor output. Neuron *45*, 587–597.

Russo, S.J., Dietz, D.M., Dumitriu, D., Morrison, J.H., Malenka, R.C., and Nestler, E.J. (2010). The addicted synapse: mechanisms of synaptic and structural plasticity in nucleus accumbens. Trends Neurosci. *33*, 267–276.

Sabatini, B.L. (2019). The impact of reporter kinetics on the interpretation of data gathered with fluorescent reporters. Preprint at bioRxiv. https://doi.org/10.1101/834895.

Sæmundsson, S., Hofmann, K., and Deisenroth, M.P. (2018). Meta reinforcement learning with latent variable Gaussian processes. Preprint at arXiv, 1803.07551. https://doi.org/10.48550/arXiv.1803.07551.

Sakata, J.T., Hampton, C.M., and Brainard, M.S. (2008). Social modulation of sequence and syllable variability in adult birdsong. J. Neurophysiol. *99*, 1700–1711.

Salamone, J.D., Steinpreis, R.E., McCullough, L.D., Smith, P., Grebel, D., and Mahan, K. (1991). Haloperidol and nucleus accumbens dopamine depletion suppress lever pressing for food but increase free food consumption in a novel food choice procedure. Psychopharmacology *104*, 515–521.

Saunders, B.T., Richard, J.M., Margolis, E.B., and Janak, P.H. (2018). Dopamine neurons create Pavlovian conditioned stimuli with circuit-defined motivational properties. Nat. Neurosci. *21*, 1072–1083.

Schultz, W. (1998). Predictive reward signal of dopamine neurons. J. Neurophysiol. *80*, 1–27.

Schultz, W., Dayan, P., and Montague, P.R. (1997). A neural substrate of prediction and reward. Science *275*, 1593–1599.

Seo, M., Lee, E., and Averbeck, B.B. (2012). Action selection and action value in frontal-striatal circuits. Neuron *74*, 947–960.

Setlow, B., Schoenbaum, G., and Gallagher, M. (2003). Neural encoding in ventral striatum during olfactory discrimination learning. Neuron *38*, 625–636.

Siniscalchi, M.J., Wang, H., and Kwan, A.C. (2019). Enhanced population coding for rewarded choices in the medial frontal cortex of the mouse. Cereb. Cortex. *29*, 4090–4106.

Song, H.F., Yang, G.R., and Wang, X.-J. (2017). Reward-based training of recurrent neural networks for cognitive and value-based tasks. Elife *6*, e21492.

Steinmetz, N.A., Zatka-Haas, P., Carandini, M., and Harris, K.D. (2019). Distributed coding of choice, action and engagement across the mouse brain. Nature *576*, 266–273.

Stuber, G.D., Sparta, D.R., Stamatakis, A.M., van Leeuwen, W.A., Hardjoprajitno, J.E., Cho, S., Tye, K.M., Kempadoo, K.A., Zhang, F., Deisseroth, K., et al. (2011). Excitatory transmission from the amygdala to nucleus accumbens facilitates reward seeking. Nature *475*, 377.

Sul, J.H., Kim, H., Huh, N., Lee, D., and Jung, M.W. (2010). Distinct roles of rodent orbitofrontal and medial prefrontal cortex in decision making. Neuron *66*, 449–460.

Suri, R.E., and Schultz, W. (1998). Learning of sequential movements by neural network model with dopamine-like reinforcement signal. Exp. Brain. Res. *121*, 350–354.

Suri, R.E., and Schultz, W. (1999). A neural network model with dopamine-like reinforcement signal that learns a spatial delayed response task. Neuroscience *91*, 871–890.

Sutton, R.S. (1988). Learning to predict by the methods of temporal differences. Mach. Learn. *3*, 9–44.

Sutton, R.S., and Barto, A.G. (1998). Reinforcement Learning: An Introduction (MIT Press).

Swanson, L.W. (1982). The projections of the ventral tegmental area and adjacent regions: a combined fluorescent retrograde tracer and immunofluorescence study in the rat. Brain. Res. Bull. *9*, 321–353.

Tai, L.-H., Lee, A.M., Benavidez, N., Bonci, A., and Wilbrecht, L. (2012). Transient stimulation of distinct subpopulations of striatal neurons mimics changes in action value. Nat. Neurosci. *15*, 1281–1289.

Taylor, J., and Robbins, T. (1986). 6-Hydroxydopamine lesions of the nucleus accumbens, but not of the caudate nucleus, attenuate enhanced responding with reward-related stimuli produced by intra-accumbens d-amphetamine. Psychopharmacology *90*, 1651–1659.

Terada, S., Sakurai, Y., Nakahara, H., and Fujisawa, S. (2017). Temporal and rate coding for discrete event sequences in the Hippocampus. Neuron *94*, 1248–1262.e4.

Thévenaz, P., Ruttimann, U.E., and Unser, M. (1998). A pyramid approach to subpixel registration based on intensity. IEEE. Trans. Image Process. *7*, 27–41.

Tian, J., Huang, R., Cohen, J.Y., Osakada, F., Kobak, D., Machens, C.K., Callaway, E.M., Uchida, N., and Watabe-Uchida, M. (2016). Distributed and mixed information in monosynaptic inputs to dopamine neurons. Neuron *91*, 1374–1389.

Tsitsiklis, J.N., and Van Roy, B. (1997). An analysis of temporal-difference learning with function approximation. IEEE Trans. Automat. Contr. *42*, 674–690.

Tsutsui, K.-I., Grabenhorst, F., Kobayashi, S., and Schultz, W. (2016). A dynamic code for economic object valuation in prefrontal cortex neurons. Nat. Commun. *7*, 12554.

Wan, X., and Peoples, L.L. (2006). Firing patterns of accumbal neurons during a pavlovian-conditioned approach task. J. Neurophysiol. *96*, 652–660.

Wang, J.X., Kurth-Nelson, Z., Kumaran, D., Tirumala, D., Soyer, H., Leibo, J.Z., Hassabis, D., and Botvinick, M. (2018). Prefrontal cortex as a meta-reinforcement learning system. Nat. Neurosci. *21*, 860–868.

Watabe-Uchida, M., Zhu, L., Ogawa, S.K., Vamanrao, A., and Uchida, N. (2012). Whole-brain mapping of direct inputs to midbrain dopamine neurons. Neuron *74*, 858–873.

Wörgötter, F., and Porr, B. (2005). Temporal sequence learning, prediction, and control: a review of different models and their relation to biological mechanisms. Neural. Comput. *17*, 245–319.

Wright, C.I., and Groenewegen, H.J. (1995). Patterns of convergence and segregation in the medial nucleus accumbens of the rat: relationships of prefrontal cortical, midline thalamic, and basal amygdaloid afferents. J. Comp. Neurol. *361*, 383–403.

Yagishita, S., Hayashi-Takagi, A., Ellis-Davies, G.C.R., Urakubo, H., Ishii, S., and Kasai, H. (2014). A critical time window for dopamine actions on the structural plasticity of dendritic spines. Science *345*, 1616–1620.

Zhou, P., Resendez, S.L., Rodriguez-Romaguera, J., Jimenez, J.C., Neufeld, S.Q., Giovannucci, A., Friedrich, J., Pnevmatikakis, E.A., Stuber, G.D., Hen, R., et al. (2018). Efficient and accurate extraction of in vivo calcium signals from microendoscopic video data. Elife *7*, e28728.

Zhou, S., Masmanidis, S.C., and Buonomano, D.V. (2020). Neural sequences as an optimal dynamical regime for the readout of time. Neuron *108*, 651–658.e5.

Zhu, Y., Wienecke, C.F.R., Nachtrab, G., and Chen, X. (2016). A thalamic input to the nucleus accumbens mediates opiate dependence. Nature *530*, 219–222.

Zhu, Y., Nachtrab, G., Keyes, P.C., Allen, W.E., Luo, L., and Chen, X. (2018). Dynamic salience processing in paraventricular thalamus gates associative learning. Science *362*, 423–429.

CellPress
OPEN ACCESS

STAR★METHODS

KEY RESOURCES TABLE

REAGENT or RESOURCE	SOURCE	IDENTIFIER
Antibodies		
Mouse monoclonal anti-GFP	Life Technologies Corporation	Cat# G10362; RRID: AB_2536526
Donkey anti-rabbit coupled to Alexa 488	Jackson ImmunoResearch	Cat# 711-545-152; RRID: AB_2313584
Bacterial and virus strains		
CAV-Cre virus	IGMM Vector core, France	NA
retroAAV-Ef1a-NLS-Cre_WPRE-hGHpA	PNI Viral Core, Princeton	; RRID: Addgene_5536
AAV2/5-CAG-Flex-GCamp6f-WPRE-SV40	UPenn Vector Core	AV-5-PV2816; RRID: Addgene_100835
AAV2/5-CamKIIa-hChR2-EYFP	UNC Vector Core	https://www.addgene.org/26969; RRID: Addgene_26969
Experimental models: Organisms/strains		
Mouse: wild type C57BL/6J	Jackson Laboratory	JAX: 000,664; RRID: ISMR_JAX_000664
Software and algorithms		
"Synaptic plasticity" temporal difference learning algorithm	Generated by this study	https://github.com/baiydaavi/RL_models_with_choice_selective_sequences
"Neural dynamics" deep reinforcement learning algorithm	Generated by this study	https://github.com/baiydaavi/RL_models_with_choice_selective_sequences
Behavioral event encoding model	Generated by this study	https://github.com/nfparker/event_encoding_model
Other		
Fibers for optogenetics	Thor Labs	BFL37-300
Ferrules for optogenetics	Precision Fiber Products	MM-FER-2006SS-330
0.5mm diameter, ~6.1mm length GRIN lens	Inscopix	GLP-0561
Imaging baseplate	Inscopix	BPL-2
Baseplate cover	Inscopix	BPC-2

RESOURCE AVAILABILITY

Lead contact

Further information and requests for resources and reagents should be directed to and will be fulfilled by the lead contact, Ilana Witten (iwitten@princeton.edu).

Materials availability

This study did not generate unique reagents.

Data and code availability

- Microscopy data reported in this paper will be shared by the lead contact upon request. Behavioral and one-photon imaging data used in this paper will be shared by the lead contact upon request.
- Original code related to the synaptic plasticity and neural dynamics models (Figures 5 and 6) and the event encoding model (Figure 2) has been deposited at GitHub and is publicly available as of the date of publication. The URLs are listed in the key resources table. All other code used in this study is available from the lead contact upon request.
- Any additional information required to reanalyze the data reported in this paper is available from the lead contact upon request.

EXPERIMENTAL MODEL AND SUBJECT DETAILS

Mice

46 male C57BL/6J mice from The Jackson Laboratory (strain 000664) were used for these experiments. Prior to surgery, mice were group-housed with 3–5 mice/cage. All mice were >6 weeks of age prior to surgery and/or behavioral training. To prevent mice from damaging the implant of cagemates, all mice used in imaging experiments were singly housed post-surgery. All mice were kept on a

12-h on/12-h off light schedule. All experiments and surgeries were performed during the light off time. All experimental procedures and animal care was performed in accordance with the guidelines set forth by the National Institutes of Health and were approved by the Princeton University Institutional Animal Care and Use Committee.

METHOD DETAILS

Probabilistic reversal learning task

Beginning three days prior to the first day of training, mice were placed on water restriction and given per diem water to maintain >80% original body weight throughout training. Mice performed the task in a 21 × 18 cm operant behavior box (MED associates, ENV-307W). A shaping protocol of three stages was used to enable training and discourage a bias from forming to the right or left lever. In all stages of training, the start of a trial was indicated by illumination of a central nose poke port. After completing a nose poke, the mouse was presented with both the right and left lever after a temporal delay drawn from a random distribution from 0 to 1s in 100ms intervals. The probability of reward of these two levers varied based on the stage of training (see below for details). After the mouse successfully pressed one of the two levers, both retracted and, after a temporal delay drawn from the same uniform distribution, the mice were presented with one of two auditory cues for 500ms indicating whether the mouse was rewarded (CS+, 5 kHz pure tone) or not rewarded (CS–, white noise). Concurrent with the CS + presentation, the mouse was presented with 6μL of 10% sucrose reward in a dish located equidistantly between the two levers, just interior to the central nose poke. The start time of reward consumption was defined as the moment the mouse first made contact with the central reward port spout following the delivery of the reward. The end of the reward consumption period (i.e., reward exit) was defined as the first moment at which the mouse was disengaged from the reward port for >100ms. In all stages of training, trials were separated by a 2s intertrial interval, which began either at the end of CS on unrewarded trials or at the end of reward consumption on rewarded trials.

In the first stage of training (“100-100 debias”), during a two-hour session, mice could make a central nose poke and be presented with both the right and left levers, each with a 100% probability of reward. However, to ensure that mice did not form a bias during this stage, after five successive presses of either lever the mouse was required to press the opposite lever to receive a reward. In this case, a single successful switch to the opposite lever returned both levers to a rewarded state. Once a mouse received >100 rewards in a single session they were moved to the second stage (“100-0”) where only one of the two levers would result in a reward. The identity of the rewarded lever reversed after 10 rewarded trials plus a random number of trials drawn from the geometric distribution:

$$P(k) = (1-p)^{k-1}p \quad \text{(Equation 1)}$$

where $P(k)$ is the probability of a block reversal k trials into a block and p is the success probability of a reversal for each trial, which in our case was 0.4. After 3 successive days of receiving >100 total rewards, the mice were moved to the final stage of training (“70-10”), during which on any given trial pressing one lever had a 70% probability of leading to reward (high-probability lever) while pressing the opposite lever had only a 10% reward probability (low-probability lever). The identity of the higher probability lever reversed using the same geometric distribution as the 100-0 training stage. On average, there were 23.23 ± 7.93 trials per block and 9.67 ± 3.66 blocks per session (mean +/− std. dev.). In this final stage, the mice were required to press either lever within 10s of their presentation; otherwise, the trial was considered an ‘abandoned trial’ and the levers retracted. All experimental data shown was collected while mice performed this final “70-10” stage.

Cellular-resolution calcium imaging

To selectively image from neurons which project to the NAc, we utilized a combinatorial virus strategy to image cortical and thalamic neurons which send projections to the NAc. 16 mice (7 PL-NAc, 9 mTH-NAc) previously trained on the probabilistic reversal learning task were unilaterally injected with 500nL of a retrogradely transporting virus to express Cre-recombinase (CAV2-cre, IGMM vector core, France, injected at ~2.5 × 10^{12} parts/mL or retroAAV-EF1a-Cre-WPRE-hGHpA, PNI vector core, injected at ~6.0 x 10^{13}) in either the right or left NAc core (1.2 mm A/P, +/− 1.0 mm M/L, −4.7 D/V) along with 600nL of a virus to express GCaMP6f in a Cre-dependent manner (AAV2/5-CAG-Flex -GCaMP6f-WPRE-SV40, UPenn vector core, injected at ~1.27 x 10^{13} parts/mL) in either the mTH (−0.3 & −0.8 A/P, +/− 0.4 M/L, −3.7 D/V) or PL (1.5 & 2.0 A/P, +/− 0.4 M/L, −2.5 D/V) of the same hemisphere. 154 of 278 (55%, n = 5 mice) PL-NAc neurons and 95 out of 256 (37%, n = 5 mice) mTH-NAc neurons were labeled using the CAV2-Cre virus, the remainder were labeled using the retroAAV-Cre virus. In this same surgery, mice were implanted with a 500 μm diameter gradient refractive index (GRIN) lens (GLP-0561, Inscopix) in the same region as the GCaMP6f injection – either the PL (1.7 A/P, +/− 0.4 M/L, −2.35 D/V) or mTH (−0.5 A/P, +/− 0.3 M/L, −3.6 D/V). 2-3 weeks after this initial surgery, mice were implanted with a base plate attached to a miniature, head-mountable, one-photon microscope (nVISTA HD v2, Inscopix) above the top of the implanted lens at a distance which focused the field of view. All coordinates are relative to bregma using *Paxinos and Franklin’s the Mouse Brain in Stereotaxic Coordinates*, *2nd edition* (Paxinos and Franklin, 2004). GRIN lens location was imaged using the Nanozoomer S60 Digital Slide Scanner (Hamamatsu) (location of implants shown in Figure S1). The subsequent image of the coronal section determined to be the center of the lens implant was then aligned to the Allen Brain Atlas (Allen Institute, brain-map.org) using the *Wholebrain* software package (wholebrainsoftware.org; Fürth et al., 2018).

Post-surgery, mice with visible calcium transients were then retrained on the task while habituating to carrying a dummy microscope attached to the implanted baseplate. After the mice acclimated to the dummy microscope, they performed the task while images of the recording field of view were acquired at 10 Hz using the Mosaic acquisition software (Inscopix). To synchronize imaging data with behavioral events, pulses from the microscope and behavioral acquisition software were recorded using either a data acquisition card (USB-201, Measurement computing) or, when LED tracking (see below for details) was performed, an RZ5D BioAmp processor from Tucker-Davis Technologies. Acquired videos were then pre-processed using the Mosaic software and spatially downsampled by a factor of 4. Subsequent down-sampled videos then went through two rounds of motion-correction. First, rigid motion in the video was corrected using the translational motion correction algorithm based on (Thévenaz et al., 1998) included in the Mosaic software (Inscopix, motion correction parameters: translation only, reference image: the mean image, speed/accuracy balance: 0.1, subtract spatial mean [r = 20 pixels], invert, and apply spatial mean [r = 5 pixels]). The video then went through multiple rounds of non-rigid motion correction using the NormCore motion correction algorithm (Pnevmatikakis and Giovannucci, 2017) NormCore parameters: gSig = 7, gSiz = 17, grid size and grid overlap ranged from 12-36 and 8-16 pixels, respectively, based on the individual motion of each video. Videos underwent multiple (no greater than 3) iterations of NormCore until non-rigid motion was no longer visible). Following motion correction, the CNMFe algorithm (Zhou et al., 2018) was used to extract the fluorescence traces (referred to as 'GCaMP6f' throughout the text) as well as an estimated firing rate of each neuron (CNMFe parameters: spatial downsample factor = 1, temporal downsample = 1, Gaussian kernel width = 4, maximum neuron diameter = 20, tau decay = 1, tau rise = 0.1). Only those neurons with an estimated firing rate of four transients/minute or higher were considered 'task-active' and included in this paper – 278/330 (84%; each mouse contributed 49,57,67,12,6,27,60 neurons, respectively) of neurons recorded from PL-NAc passed this threshold while 256/328 (78%; each mouse contributed 17,28,20,46,47,40,13,13,32 neurons, respectively) passed in mTH-NAc. Across all figures, to normalize the neural activity across different neurons and between mice, we Z-scored each GCaMP6f recording trace using the mean and standard deviation calculated using the entire recording session.

Optogenetic stimulation of PL-NAc neurons

22 male C57BL/6J mice were bilaterally injected in either the PL (n = 14 mice, M–L ± 0.4, A–P 2.0 and D–V −2.5 mm) or mTH (n = 8 mice, M–L ± 0.3, A–P −0.7 and D–V −3.6 mm) with 600nL AAV2/5-CaMKIIa-hChR2-EYFP (UPenn vector core, injected 0.6 μL per hemisphere of titer of 9.6×10^{13} pp per ml). Optical fibers (300 μm core diameter, 0.37 NA) delivering 1–3 mW of 447 nm laser light (measured at the fiber tip) were implanted bilaterally above the NAc Core at a 10° angle (M–L ± 1.1, A–P 1.4 and D–V −4.2 mm). An additional cohort of control mice (n = 8) were implanted with optical fibers in the NAc without injection of ChR2 and underwent the same stimulation protocol outlined below (Figures S12E–12H). Mice were anesthetized for implant surgeries with isoflurane (3–4% induction and 1–2% maintenance). Mice were given 5 days of recovery after the surgical procedure before behavioral testing.

During behavioral sessions, 5 ms pulses of 1–3 mW, 447 nm blue light was delivered at 20 Hz on a randomly selected 10% of trials beginning when the mouse entered the central nose poke. Light stimulation on unrewarded trials ended 1s after the end of the CS– presentation. On rewarded trials, light administration ended either 1s after CS + presentation ('cohort 1') or at the end of reward consumption, as measured by the mouse not engaging the reward port for 100ms ('cohort 2'). See Figure S13 for a schematic of stimulation times as well as the behavior of the two cohorts. Mice alternated between sessions with and without stimulation – sessions without stimulation were excluded from analysis. Anatomical targeting was confirmed as successful in all mice through histology after the experiment, and therefore no mice were excluded from this dataset.

To quantify the effect of laser stimulation on latency times shown in Figures S12A–12D, we ran a mixed effects linear model using the *fitglme* package in MATLAB. In this model, the median latency to initiate a trial of a mouse, defined as the time between illumination of the central nose poke (i.e., trial start) and the mouse initiating a trial via nose poke, was predicted using i) opsin identity (PL-NAc CaMKII-ChR2, mTH-NAc CaMKII-ChR2 or no-opsin controls), ii) laser stimulation on the current trial, iii) laser stimulation on the previous trial, iv) the interaction between opsin identity and laser stimulation on the current trial and v) the interaction between opsin and laser stimulation on the previous trial. To account for individual variation between mice, a random effect of mouse ID was included.

QUANTIFICATION AND STATISTICAL ANALYSIS

Logistic choice regression

For the logistic choice regressions shown in Figures 1E and S2A, we modeled the choice of the mouse on trial *i* based on lever choice and reward outcome information from the previous n trials using the following logistic regression model:

$$\log\left(\frac{C(i)}{1 - C(i)}\right) = \beta_0 + \sum_{j=1}^{n} \beta_j^R R(i-j) + \sum_{j=1}^{n} \beta_j^U U(i-j) + error \qquad \text{(Equation 2)}$$

where $C(i)$ is the probability of choosing the right lever on trial i, and $R(i\text{-}j)$ and $U(i\text{-}j)$ are the choice of the mouse j trials back from the ith trial for either rewarded or unrewarded trials, respectively. $R(i\text{-}j)$ was defined as +1 when the jth trial back was both rewarded and a right press, −1 when the jth trial back was rewarded and a left press and 0 when it was unrewarded. Similarly, $U(i\text{-}j)$ was defined as +1 when the jth trial back was both unrewarded and a right press, −1 when the jth trial back was unrewarded and a left press and 0 when it

was rewarded. The calculated regression coefficients, β_j^R and β_j^U, reflect the strength of the relationship between the identity of the chosen lever on a previously rewarded or unrewarded trial, respectively, and the lever chosen on the current trial.

To examine the effect of optogenetic stimulation from multiple trials back on the mouse's choice (Figure 7C, 7E, 7H, 7K; S12H and S13C and S13D), we expanded our behavioral logistic regression model to include the identity of those trials with optical stimulation, as well as the interaction between rewarded and unrewarded choice predictors and stimulation:

$$\log\left(\frac{C(i)}{1-C(i)}\right) = \beta_0 + \sum_{j=1}^{n}\beta_j^R R(i-j) + \sum_{j=1}^{n}\beta_j^U U(i-j) + \ldots$$
$$\sum_{j=1}^{n}\beta_j^{LR} L(i-j)R(i-j) + \sum_{j=1}^{n}\beta_j^{LU} L(i-j)U(i-j) + \sum_{j=1}^{n}\beta_j^{L} L(i-j) + error \quad \text{(Equation 3)}$$

where $L(i)$ represents optical stimulation on the ith trial (1 for optical stimulation, 0 for control trials), β_j^L represents the coefficient corresponding to the effect of stimulation on choice j trials back, and β_j^{LR} and β_j^{LU} represent the coefficients corresponding to the interaction between rewarded choice x optical stimulation and unrewarded choice x stimulation, respectively.

To visualize the relative influence of stimulation on the mice's choices compared with unstimulated trials, in Figures 7C, 7E, 7H, 7K, S12H, S13C, and S13D, the solid blue trace represents the sum of the rewarded choice coefficients (represented by the black trace) and rewarded choice x stimulation coefficients ($\beta_j^R + \beta_j^{LR}$). Similarly, the dashed blue trace represents the sum of the unrewarded choice coefficients (gray trace) and unrewarded choice x stimulation coefficients ($\beta_j^U + \beta_j^{LU}$). For all choice regressions, the coefficients for each mouse were fit using the *glmfit* function in MATLAB and error bars represent mean ± SEM across mice.

Encoding model to generate response kernels for behavioral events

To determine the response of each neuron attributable to each of the events in our task, we used a multiple linear encoding model with lasso regularization to generate a response kernel for each behavioral event (example kernels shown in Figure 2B). In this model, the dependent variable was the GCaMP6f trace of each neuron recorded during a behavioral session and the independent variables were the times of each behavioral event ('nose poke', 'levers out', 'ipsilateral lever press', 'contralateral lever press', 'CS+', 'CS–' and 'reward consumption) convolved with a 25 degrees of freedom spline basis set that spanned −2 to 6s before and after the time of action events ('nose poke', 'ipsilateral press', 'contralateral press' and 'reward consumption') and 0 to 8s from stimulus events ('levers out', 'CS+' and 'CS–'). To generate this kernel, we used the following linear regression with lasso regularization using the *lasso* function in MATLAB:

$$\min_{\beta_0,\beta_{jk}}\left(\sum_{t=1}^{T}\left(F(t) - \sum_{k=1}^{K}\sum_{j=1}^{N_{sp}}\beta_{jk}X_{jk}(t) - \beta_0\right)^2 + \lambda\sum_{k=1}^{K}\sum_{j=1}^{N_{sp}}|\beta_{jk}|\right) \quad \text{(Equation 4)}$$

where $F(t)$ is the Z-scored GCaMP6f fluorescence of a given neuron at time t, T is the total time of recording, K is the total number of behavioral events used in the model, N_{sp} is the degrees of freedom for the spline basis set (25 in all cases, splines generated using the FDAfuns MATLAB package), β_{jk} is the regression coefficient for the jth spline basis function and k^{th} behavioral event, β_0 is the intercept term and λ is the lasso penalty coefficient. The value of lambda was chosen for each neuron that minimized the mean squared error of the model, as determined by 5-fold cross validation. The predictors in our model, X_{jk}, were generated by convolving the behavioral events with a spline basis set, to enable temporally delayed versions of the events to predict neural activity:

$$X_{jk}(t) = \sum_{i=1}^{N=81} S_j(i)e_k(t-i) \quad \text{(Equation 5)}$$

where $S_j(i)$ is the jth spline basis function at time point i with a length of 81 time bins (time window of −2 to 6s for action events or 0 to 8s for stimulus events sampled at 10 Hz) and e_k is a binary vector of length T representing the time of each behavioral event k (1 at each time point where a behavioral event was recorded using the MED associates and TDT software, 0 at all other timepoints).

Using the regression coefficients, β_{jk}, generated from the above model, we then calculated a 'response kernel' for each behavioral event:

$$\text{kernel}_k(t) = \sum_{j=1}^{N_{sp}}\beta_{jk}S_j(t) \quad \text{(Equation 6)}$$

This kernel represents the (linear) response of a neuron to each behavioral event, while accounting for the linear component of the response of this neuron to the other events in the task.

Quantification of neural modulation to behavioral events

To identify neurons that were significantly modulated by each of the behavioral events in our task (fractions shown in Figures 2G and 2H), we used the encoding model described above, but without the lasso regularization:

$$F(t) = \beta_0 + \sum_{k=1}^{K} \sum_{j=1}^{N_{sp}} \beta_{jk} X_{jk}(t) \quad \text{(Equation 7)}$$

As above, $F(t)$ is the Z-scored GCaMP6f fluorescence of a given neuron at time t, K is the total number of behavioral events used in the model, N_{sp} is the degrees of freedom for the spline basis set (25 in all cases), β_{jk} is the regression coefficient for the jth spline basis function and k^{th} behavioral event and β_0 is the intercept term. To determine the relative contribution of each behavioral event when predicting the activity of a neuron, we compared the full version of this model to a reduced model with the X and β terms associated with the behavioral event in question excluded. For each behavioral event, we first generated an F-statistic by comparing the fit of a full model containing all event predictors with that of a reduced model that lacks the predictors associated with the event in question. We then calculated this same statistic on 500 instances of shuffled data, where shuffling was performed by circularly shifting the GCaMP6f fluorescence by a random integer. We then compared the F-statistic from the real data to the shuffled distribution to determine whether the removal of an event as a predictor compromised the model significantly more than expected by chance. If the resulting p-value was less than the significance threshold of $p = 0.01$, after accounting for multiple comparison testing of each of the behavioral events by Bonferroni correction, then the event was considered significantly encoded by that neuron.

To determine whether a neuron was significantly selective to the choice or outcome of a trial ('choice-selective' and 'outcome-selective', fractions of neurons from each population shown in Figures 3A and 3C), we utilized a nested model comparison test similar to that used to determine significant modulation of behavioral events above, where the full model used the following behavioral events as predictors: 'nose poke', 'levers out', 'all lever press', 'ipsilateral lever press', 'all CS', 'CS+' and 'reward consumption'. For choice-selectivity, an F-statistic was computed for a reduced model lacking the 'ipsilateral lever press' predictors and significance was determined by comparing this value with a null distribution generated using shuffled data as described above. For outcome-selectivity, the reduced model used to test for significance lacked the predictors associated with both the 'CS+' and 'reward consumption' events.

By separating the lever press and outcome-related events into predictors that were either blind to the choice or outcome of the trial ('all lever press' and 'all CS', respectively) and those which included choice or outcome information ('ipsilateral lever press' or 'CS+' and 'reward consumption', respectively) we were able to determine whether the model was significantly impacted by the removal of either choice or outcome information. Therefore, neurons with significant encoding of the 'ipsilateral lever press' event (using the same p-value threshold determined by the shuffled distribution of F-statistics) were considered choice-selective, while those with significant encoding of the 'CS+/reward consumption' events were considered outcome-selective.

Neural decoders

Choice decoder

In Figure 3B, we quantified how well simultaneously imaged populations of 1–10 PL-NAc or mTH-NAc neurons could be used to decode choice using a logistic regression:

$$\log\left(\frac{C(i)}{1 - C(i)}\right) = \beta_0 + \sum_{j=1}^{n} \beta_j X_j(i) + \varepsilon \quad \text{(Equation 8)}$$

where $C(i)$ is the probability the mouse made an ipsilateral choice on trial i, β_0 is the offset term, n is the number of neurons (between 1 and 10), β_j is the regression weight for each neuron, $X_j(i)$ is the mean z-scored GCaMP6f fluorescence from -2s to 6s around the lever press on trial i and ε is the error term.

Given that the mice's choices were correlated across neighboring trials, we weighted the logistic regression based on the frequency of each trial type combination. This was to ensure that choice decoding of a given trial was a reflection of the identity of the lever press on the current trial as opposed to that of the previous or future trial. Thus, we classified each trial as one of eight 'press sequence types' based on the following 'previous-current-future' press sequences: ipsi-ipsi-ipsi, ipsi-ipsi-contra, ipsi-contra-contra, ipsi-contra-ipsi, contra-contra-contra, contra-contra-ipsi, contra-ipsi-ipsi, contra-ipsi-contra. We then used this classification to equalize the effects of press-sequence type on our decoder by generating weights corresponding to the inverse of the frequency of the press sequence type of that trial. These weights were then used as an input to the *fitglm* function in MATLAB, which was used to fit a weighted version of the logistic regression model above (Equation 8).

Decoder performance was evaluated with 5-fold cross-validation by calculating the proportion of correctly classified held-out trials. Predicted ipsilateral press probabilities greater than or equal to 0.5 were decoded as an ipsilateral choice and values less than 0.5 were decoded as a contralateral choice. This was repeated with 100 combinations of randomly selected, simultaneously imaged neurons from each mouse. Reported decoding accuracy is the average accuracy across the 100 runs and 5 combinations of train-test data for each mouse. Note that only 6/7 mice in the PL-NAc cohort were used in the decoder analyses as one mouse had fewer than 10 simultaneously imaged neurons.

Outcome decoder

For the outcome decoder in Figure 3D, we used the same weighted logistic regression used for choice decoding, except the dependent variable was the outcome of the trial (+1 for a reward, 0 for no reward) and the predictors were the average GCaMP6f fluorescence during the intertrial interval (ITI) of each trial. The ITI was defined as the time between CS presentation and either 1s before the

next trial's nose poke or 8s after the CS, whichever occurred first. This was used in order to avoid including any neural activity attributable to the next trial's nose poke in our analysis.

To correct for outcome correlations between neighboring trials, we performed a similar weighting of predictors as performed in the choice decoder above using the following eight outcome sequence types: 'reward-reward- reward', 'reward-reward- unreward', 'reward-unreward- unreward', 'reward-unreward- reward', 'unreward-unreward- unreward', 'unreward-unreward- reward', 'unreward-reward- reward', 'unreward-reward- unreward.'

Time course choice decoder

To determine how well activity from PL-NAc and mTH-NAc neurons was able to predict the mouse's choice as a function of time throughout the trial (Figures 4E, S7E, and S8E), we trained separate logistic regressions on 500ms bins throughout the trial, using the GCaMP6f fluorescence of 10 simultaneously imaged neurons.

Because of the variability in task timing imposed by the jitter and variability of the mice's actions, we linearly interpolated the GCaMP6f fluorescence trace of each trial to a uniform length, t_{adjusted}, relative to behavioral events in our task. Specifically, for each trial, T, we divided time into the following four epochs: (i) 2s before nose poke, (ii) time from the nose poke to the lever press, (iii) time from the lever press to the nose poke of the subsequent trial, T+1 and (iv) the 3s following the next trial nosepoke. For epochs *ii* and *iii*, t_{adjusted} was determined by interpolating the GCaMP6f fluorescence trace from each trial to a uniform length defined as the median time between the flanking events across all trials. Thus, t_{adjusted} within each epoch for each trial, T, was defined as:

$$T_{adjusted}(t) \equiv \begin{cases} t, & \\ 2 + \dfrac{t - t_{np}^{T}}{t_{lp}^{T} - t_{np}^{T}} \widetilde{ep_{ii}}, & t_{np}^{T} - 2 \le t < t_{np}^{T} \\ & t_{np}^{T} \le t < t_{lp}^{T} \\ 2 + \widetilde{ep_{ii}} + \dfrac{t - t_{np}^{T}}{t_{np}^{T+1} - t_{lp}^{T}} \widetilde{ep_{iii}}, & t_{lp}^{T} \le t < t_{np}^{T+1} \\ & t_{np}^{T+1} \le t < t_{np}^{T+1} + 3 \\ t, & \end{cases} \quad \text{(Equation 9)}$$

where t_{np}^{T}, and t_{lp}^{T} are the times of the nose poke and lever press on the current trial, t_{np}^{T+1} is the time of the nose poke of the subsequent trial and $\widetilde{ep_{ii}}$ and $\widetilde{ep_{iii}}$ are the median times across trials of epoch *ii* and *iii*.

The resulting time-adjusted GCaMP6f traces were divided into 500ms bins. For each bin, we fit the weighted logistic regression described above to predict choice on the current, previous or future trial from the activity of 10 simultaneously imaged neurons. Predictors were weighted based on press sequence type as described above. Decoding accuracy was assessed as described above using 100 combinations of 10 randomly selected neurons and 5-fold cross-validation. To determine if decoding was significantly above chance, which is 0.5, for each time point we performed a two-tailed, one-sample t test.

Statistics

All t-tests reported in the results and as specified in each figure legend were performed using either the *ttest* or *ttest2* function in MATLAB. In all cases, t-tests were two-tailed. In cases where multiple comparisons were performed, we applied a Bonferroni correction to determine the significance threshold. Two-proportion Z-tests (used to compare fractions of significantly modulated/selective neurons, Figures 2H, 3A and 3C) and Fisher's Z (used to compare correlation coefficients, Figures 4D and S7D) were performed using Vassarstats.net. Asterisks indicating significance thresholds are referenced in respective figure legends.

For all t-tests in this paper, data distributions were assumed to be normal, but this was not formally tested. No statistical methods were used to predetermine sample sizes, but our sample sizes were similar to those generally employed in the field.

Synaptic plasticity model

To computationally model how the brain could solve the reversal learning task using fast dopamine-mediated synaptic plasticity, we generated a biological instantiation of the TD algorithm for reinforcement learning (Sutton and Barto, 1998) by combining the recorded PL-NAc activity with known circuit connectivity in the NAc and associated regions (Hunnicutt et al., 2016; Kalivas et al., 1993; Otis et al., 2017; Watabe-Uchida et al., 2012). The goal of the model is to solve the "temporal credit assignment problem" by learning the value of each choice at the onset of the choice-selective PL-NAc sequence, when we assume the mouse makes its decision and which is well before the time of reward.

Synaptic plasticity model description

The value function

Our implementation of the TD algorithm seeks to learn an estimate, at any given time, of the total discounted sum of expected future rewards, known as the value function $V(t)$. To do this, we assume that the value function over time is decomposed into a weighted sum of temporal basis functions $f_i^R(t)$ and $f_i^L(t)$ (Sutton and Barto, 1998) corresponding to the right-choice and left-choice preferring neurons:

$$V_R(t) = \sum_{i=1}^{n_R} w_i^R(t) f_i^R(t)$$
$$V_L(t) = \sum_{i=1}^{n_L} w_i^L(t) f_i^L(t) \quad \text{(Equation 10)}$$

with the total value being given by the sum over both the left and right neurons as

$$V(t) = V_R(t) + V_L(t) \quad \text{(Equation 11)}$$

Here, V_R(t) and V_L(t) are the components of the value functions encoded by the right- and left-preferring neurons respectively, n_R and n_L are the number of right- and left-preferring choice-selective neurons respectively, and $w_i^{R,L}$ are the weights between the ith PL neuron and the NAc, which multiply the corresponding basis functions. Thus, each term in V_R(t) or V_L(t) above corresponds to the activity of one of the striatal neurons in the model (Figure 5A). Note that, in our model, the total value *V*(t) sums the values associated with the left and right actions and is thus not associated with a particular action. At any given time on a given trial, the choice-selective activity inherent to the recorded PL-NAc neurons results predominantly in activation of the sequence corresponding to the chosen lever compared to the unchosen lever (see Figure 5C), so that a single sequence, corresponding to the chosen action, gets reinforced.

The reward prediction error (RPE)

TD learning updates the value function iteratively by computing errors in the predicted value function and using these to update the weights w_i. The RPE at each moment of time is calculated from the change in the estimated value function over a time step of size *dt* as follows

$$RPE = \delta(t)dt = r(t)dt + e^{\frac{-dt}{\tau}}V(t) - V(t-dt) \quad \text{(Equation 12)}$$

where $\delta(t)$ is the reward prediction error per unit time. Here, the first two terms represent the estimated value at time *t*, which equals the sum of the total reward received at time *t* and the (discounted) expectation of rewards, i.e., value at all times into the future. This is compared to the previous time step's estimated value *V*(*t*-*dt*). The coefficient $e^{\frac{-dt}{\tau}}$ represents the temporal discounting of rewards incurred over the time step *dt*. Here τ denotes the timescale of temporal discounting and was chosen to be 0.7s.

To translate this continuous time representation of RPE signals to our biological circuit model, we assume that the RPE $\delta(t)$ is carried by dopamine neurons (Montague et al., 1996; Schultz et al., 1997). These dopamine neurons receive three inputs corresponding to the three terms on the right side of the above equation: a reward signal originating from outside the VTA, a discounted estimate of the value function *V*(*t*) that, in Figure 5A, represents input from the striatum via the ventral pallidum (Chen et al., 2019; Tian et al., 2016) and an oppositely signed, delayed copy of the value function *V*(*t*-Δ) that converges upon the VTA interneurons (Cohen et al., 2012).

Because the analytical formulation of TD learning in continuous time is defined in terms of the infinitesimal time step *dt*, but a realistic circuit implementation needs to be characterized by a finite delay time for the disynaptic pathway through the VTA interneurons, we rewrite the above equation approximately for small, but finite delay Δ as:

$$\delta(t)dt = r(t)dt + \frac{\gamma V(t) - V(t-\Delta)}{\Delta}dt \quad \text{(Equation 13)}$$

where we have defined $\gamma = e^{-\frac{\Delta}{\tau}}$ as the discount factor corresponding to one interneuron time delay and, in all simulations, we chose a delay time Δ = 0.01s. Note that the discount factor is biologically implemented in different strengths of the weights of the VP inputs to the GABA interneuron and dopaminergic neuron in the VTA.

The proposed circuit architecture of Figure 5A can be rearranged into several other, mathematically equivalent architectures (Figure S9). These architectures are not mutually exclusive, so other more complicated architectures could be generated by superpositions of these architectures.

The eligibility trace

The RPE at each time step $\delta(t)$ was used to update the weights of the recently activated synapses, where the "eligibility" $E_i(t)$ of a synapse for updating depends upon an exponentially weighted average of its recent past activity (Gerstner et al., 2018; Sutton and Barto, 1998):

$$E_i(t) = \int_{-\infty}^{t} e^{\frac{s-t}{\tau_e}} f_i(s)ds \quad \text{(Equation 14)}$$

which can be rewritten as

$$\frac{dE_i(t)}{dt} = -\frac{E_i(t)}{\tau_e} + f_i(t) \quad \text{(Equation 15)}$$

or, in the limit dt<<1,

$$E_i(t) \approx e^{-\frac{dt}{\tau_e}}E_i(t-dt) + f_i(t)dt \quad \text{(Equation 16)}$$

where τ_e defines the time constant of the decay of the eligibility trace, which was chosen to be 0.8s consistent with (Gerstner et al., 2018; Yagishita et al., 2014).

Weight updates

The weight of each PL-NAc synapse, w_i, is updated according to the product of its eligibility $E_i(t)$ and the RPE rate $\delta(t)$ at that time using the following update rule (Gerstner et al., 2018; Sutton and Barto, 1998):

$$\frac{d\widehat{w}_i(t)}{dt} = \alpha\delta(t)E_i(t)$$
$$w_i(t) = \max[0, \widehat{w}_i(t)] \qquad \text{(Equation 17)}$$

where $\alpha = 0.009(\text{spikes/s})^{-1}$ was the learning rate. Note that the units of α derive from the units of weight being *value*(spikes/s)$^{-1}$. The PL-NAc weights used in the model are thresholded to be non-negative so that the weights obey Dale's principle.

Action selection

In the model, the decision to go left or right is determined by "probing" the relative values of the left versus right actions just prior to the start of the choice-selective sequence. To implement this, we assumed that the choice was readout in a noisy, probabilistic manner from the activity of the cluster of neurons that responded at the time choice-selectivity robustly appeared, when we assume the decision is made. This corresponded to the first 17 neurons in each (left or right) PL population prior to the start of the sequential activity. This was accomplished by providing a 50 ms long, noisy probe input to each of these PL neurons and reading out the summed activity of the left and the summed activity of the right striatal populations. The difference between these summed activities was then put through a softmax function (given below) to produce the probabilistic decision.

To describe this decision process quantitatively, we define the probability of making a leftward or rightward choice in terms of underlying decision variables d_{left} and d_{right} corresponding to the summed activity of the first 17 striatal neurons in each population:

$$d_{left} = \mathbb{E}_t\left[\sum_{i=1}^{17} w_i^{left} n_i^{left}(t)\right]$$
$$d_{right} = \mathbb{E}_t\left[\sum_{i=1}^{17} w_i^{right} n_i^{right}(t)\right] \qquad \text{(Equation 18)}$$

where $\mathbb{E}_t[.]$ denotes time-averaging over the 50 ms probe period and $n_i^{left}(t)$ and $n_i^{right}(t)$ denote the non-negative stochastic probe input, which was chosen independently for each neuron and each time step from a normal distribution (truncated at zero to enforce non-negativity) with mean prior to truncation equal to 0.05s^{-1} (5% of peak activity) and a standard deviation of ${}^{0.0025}/_{\sqrt{dt}}\ s^{-1}$. Note that the weights $w_i^{left/right}$ used here correspond to the weights from the end of the previous trial, which we assume are the same as the weights at the beginning of the next trial. The probability of choosing the left or the right lever for a given trial *n* is modeled as a softmax function of these decision variables plus a "stay with the previous choice" term that models the tendency of mice in our study to return to the previously chosen lever irrespective of reward (Figure 1D), given by the softmax distribution

$$Prob(left) = \frac{\exp(\beta_{value}d_{left} + \beta_{stay}l_{left})}{\exp(\beta_{value}d_{left} + \beta_{stay}l_{left}) + \exp(\beta_{value}d_{right} + \beta_{stay}l_{right})}$$
$$Prob(right) = \frac{\exp(\beta_{value}d_{right} + \beta_{stay}l_{right})}{\exp(\beta_{value}d_{left} + \beta_{stay}l_{left}) + \exp(\beta_{value}d_{right} + \beta_{stay}l_{right})} \qquad \text{(Equation 19)}$$

where $l_{left/right}$ is 1 if that action (i.e., left or right) was chosen on the previous trial and 0 otherwise, and $\beta_{value} = 7000$ and $\beta_{stay} = 0.15$ are free parameters that define the width of the softmax distribution and the relative weighting of the value-driven versus stay contributions to the choice.

Synaptic plasticity model implementation

Block structure for the model

Block reversals were determined using the same criteria as in the probabilistic reversal learning task performed by the mice – the identity of the rewarded lever reversed after 10 rewarded trials plus a random number of trials drawn from the geometric distribution given by Equation 1. The model used p = 0.4 as in the reversal learning experiments. Given the variation in performance across the models that use PL-NAc, mTH-NAc or early-only activity as input (see Figure 5), the average block length for each model varied as well (because block reversals depended upon the number of rewarded trials). The average block length for the single-trial PL-NAc model, single-trial mTH-NAc model and early-only control were 23.0 ± 7.6, 28.1 ± 8.8 and 25.1 ± 6.3 trials (mean +/− std. dev.), respectively. The PL-NAc model produced a similar block length as that of behaving mice (23.2 ± 7.9 trials, mean +/− std. dev.). Because a block reversal in our task is dependent on the mice receiving a set number of rewards, the choices just prior to a block

reversal are more likely to align with the identity of the block and result in reward (see Figure 5E, 5J, and 5O). Thus, the increase in choice probability observed on trials close to the block reversal is an artifact of this reversal rule and not reflective of the model learning choice values.

PL-NAc inputs to the neural circuit model

To generate the temporal basis functions $f_i(t)$ (example activity shown in Figure 5C), we used the choice-selective sequential activity recorded from the PL-NAc neurons shown in Figures 4B and 4C. Spiking activity was inferred from calcium fluorescence using the CNMFe algorithm (Zhou et al., 2018) and choice-selectivity was determined using the nested comparison model used to generate Figure 3A (see "Quantification of neural modulation to behavioral events" above for details). Model firing rates were generated by Z-scoring the inferred spiking activity of each choice-selective PL-NAc neuron. The resulting model firing rates were interpolated using the *interp* function from Python's numpy package to match the time step, $dt = 0.01$s, and smoothed using a Gaussian kernel with zero mean and a standard deviation of 0.2s using the *Gaussian_filter1d* function from the ndimage module in Python's SciPy package.

To generate a large population of model input neurons on each trial, we created a population of 368 choice-selective "pseudoneurons" on each trial. This was done as follows: for each simulated trial, we created 4 copies (pseudoneurons) of each of the 92 recorded choice-selective PL-NAc neurons using that neuron's inferred spiking activity from 4 different randomly selected trials. The pool of experimentally recorded trials from which pseudoneuron activities were chosen was balanced to have equal numbers of stay and switch trials. This was done because the choices of the mice were strongly positively correlated from trial to trial (i.e., had more stay than switch trials), which (if left uncorrected) potentially could lead to biases in model performance if activity late in a trial was reflective of choice on the next, rather than the present trial. To avoid choice bias in the model, we combined the activity of left- and right-choice-preferring recorded neurons when creating the pool of pseudoneurons. We then randomly selected 184 left-choice-preferring and 184 right-choice-preferring model neurons from this pool of pseudoneurons. An identical procedure, using the 92 most choice-selective mTH-NAc neurons, was followed to create the model mTH-NAc neurons. The identity of these 92 neurons was determined by ranking each neuron's choice-selectivity using the p value calculated to determine choice-selectivity (see "Quantification of neural modulation to behavioral events" above for details).

To generate the early-only control activity (example activity shown in Figure 5M), similar to the PL-NAc activity, we created a population of 368 pseudoneurons on each trial that were divided into 184 left-choice-preferring and 184 right-choice-preferring pseudoneurons. However, in this case, we only used the early-firing neurons (neurons active at the onset of the sequence) of the PL-NAc population to create the pseudoneurons. Thus, for this control simulation, all neurons contribute to the decision as they are all active at the onset of the sequence when the model makes its choice. More specifically, to create a pool of pseudoneurons, we created multiple copies of each of the first 17 neurons of the left-choice-preferring and right-choice-preferring PL-NAc population, where each copy corresponds to the activity of the neuron on a different randomly selected trial. We then randomly selected 184 left-choice-preferring and 184 right-choice-preferring model neurons from this pool of pseudoneurons. We used a smaller learning rate $\alpha = 0.003\left(spikes/_{s}\right)^{-1}$ in this case in order to prevent the PL-NAc synaptic weights from exhibiting unstable growth. We also adjust $\beta_{value} = 1000$ in order to match the stay probability following rewarded trials to that of the model with recorded PL-NAc input (Figure 5P).

To mimic the PL-NAc activity during the optogenetic stimulation of PL-NAc neurons (Figures 7B and 7C), we set $f_i^{R,L}(t)$ equal to 0.2 for a randomly selected 70% of PL neurons, at all times t, from the time of the simulated nosepoke to 2s after the reward presentation. These 'stimulation trials' occurred on a random 10% of trials. 70% of PL neurons were activated to mimic the incomplete penetrance of ChR2 viral expression.

Reward input to the neural circuit model. The reward input $r(t)$ to the dopamine neurons was modeled by a truncated Gaussian temporal profile centered at the time of the peak reward:

$$r(t) = R(i)\frac{1}{\sqrt{2\pi\sigma_r^2}}e^{-\frac{(t-\mu_r)^2}{2\sigma_r^2}} \quad \text{(Equation 20)}$$

where $R(i)$ is 1 if trial i was rewarded and 0 otherwise, μ_r is the time of peak reward and σ_r defines the width of the Gaussian (0.3s in all cases, width chosen to approximate distribution of dopamine activity in response to reward stimuli observed in previous studies such as Matsumoto and Hikosaka, 2009 and Schultz et al., 1997). For each trial, a value of μ_r was randomly drawn from a uniform distribution spanning 0.2–1.2s from the time of the lever press. This distribution was chosen to reflect the 1s jitter between lever press and reward used in our behavioral task (see Methods above) as well as the observed delay between reward presentation and peak dopamine release in a variety of studies (Cohen et al., 2012; Matsumoto and Hikosaka, 2009; Parker et al., 2016; Saunders et al., 2018). To ensure that no residual reward response occurred before the time of the lever press, $r(t)$ was set to 0 for any time t that was 0.2s before the time of the peak reward, μ_r.

Initial weights

The performance of the model does not depend on the choice of the initial weights as the model learns the correct weights by the end of the first block irrespective of the chosen initial weights. We chose the initial weights to be zero.

Weight and eligibility update implementation

We assumed that the weight and eligibility trace updates start at the time of the simulated nose poke. The nose poke time, relative to the time of the lever press, varies due to a variable delay between the nose poke and the lever presentation as well as variation in time between lever presentation and lever press. To account for this, the weight and eligibility trace updates are initiated at time $t = t_{\text{start}}$, where t_{start} was drawn from a Gaussian distribution with a mean at −2.5s, and a variance of 0.2s, which was approximately both the time of the nose poke and the time at which choice-selective sequences initiated in the experimental recordings. The eligibility trace is reset to zero at the beginning of each trial. We stopped updating the weights at the end of the trial, defined as 3s after the time of lever press. The eligibility traces were updated according to Equation 16. The weights were updated by integrating Equation 17 with a first-order forward Euler routine. In all simulations, we used a simulation time step $dt = 0.01s$.

Neural dynamics model

To computationally model how the brain could solve the reversal learning task without fast dopamine-mediated synaptic plasticity, we used an actor-critic network based on the meta-RL framework introduced by Wang et al. (2018). The model actor and critic networks are recurrent neural networks of Long Short-Term Memory (LSTM) units whose weights are learned slowly during the training phase of the task. The weights are then frozen during the testing phase so that fast reversal learning occurs only through the activation dynamics of the recurrent actor-critic network. Like the synaptic plasticity model, we input recorded PL-NAc activity to a value-generating “critic” network (conceived of as NAc, VP, and associated cortical regions) to generate appropriate reward prediction error signals in dopamine neurons. Unlike the synaptic plasticity model, the reward prediction error signals in this model are sent to an explicit actor network (conceived of as DMS and associated cortical regions), where they act as an input to help generate appropriate action signals based on reward history.

Neural dynamics model description

LSTM

The model comprises two separate fully connected, gated recurrent neural networks of LSTM units, one each for the actor and critic network. An LSTM unit works by keeping track of a "long-term memory" state ("memory state" $\boldsymbol{c}(t)$, also known as cell state) and a "short-term memory" state ("output state" $\boldsymbol{h}(t)$, also known as hidden state) at all times. To regulate the information to be kept or discarded in the memory and output states, LSTMs use three types of gates: the input gate $\boldsymbol{i}(t)$ regulates what information is input to the network, the forget gate $\boldsymbol{\varphi}(t)$ regulates what information to forget from the previous memory state, and the output gate $\boldsymbol{o}(t)$ (not to be confused with the output state $\boldsymbol{h}(t)$ regulates the output of the network. More precisely, the dynamics of an LSTM is defined by the following equations:

$$\begin{aligned}\boldsymbol{\varphi}(t) &= \sigma(\boldsymbol{W}_\varphi \boldsymbol{x}(t) + \boldsymbol{U}_\varphi \boldsymbol{h}(t-\Delta t) + \boldsymbol{b}_\varphi)\\ \boldsymbol{i}(t) &= \sigma(\boldsymbol{W}_i \boldsymbol{x}(t) + \boldsymbol{U}_i \boldsymbol{h}(t-\Delta t) + \boldsymbol{b}_i)\\ \boldsymbol{o}(t) &= \sigma(\boldsymbol{W}_o \boldsymbol{x}(t) + \boldsymbol{U}_o \boldsymbol{h}(t-\Delta t) + \boldsymbol{b}_o)\\ \boldsymbol{c}(t) &= \boldsymbol{\varphi}(t)\odot \boldsymbol{c}(t-\Delta t) + \boldsymbol{i}(t)\odot \tanh(\boldsymbol{W}_c \boldsymbol{x}(t) + \boldsymbol{U}_c \boldsymbol{h}(t-\Delta t) + \boldsymbol{b}_c)\\ \boldsymbol{h}(t) &= \boldsymbol{o}(t)\odot \tanh(\boldsymbol{c}(t))\end{aligned} \qquad \text{(Equation 21)}$$

where $\boldsymbol{x}(t)$ is the vector of external inputs to the LSTM network at time step t, $\boldsymbol{W}_q$ and $\boldsymbol{U}_q$ are the weight matrices of the input and recurrent connections, respectively, where the subscript q denotes the state or gate being updated, $\boldsymbol{b}_q$ are the bias vectors, $\odot$ denotes element-wise multiplication and σ denotes the softmax function.

Critic network

As in the synaptic plasticity model, the goal of the critic is to learn the value (discounted sum of future rewards) of a given choice at any time in a trial. The learned value signal can then be used to generate the RPE signals that are sent to the actor. The critic is modeled as a network of LSTM units that linearly project through trainable weights to a value readout neuron that represents the estimated value $V(t)$ at time step t. The critic takes as input the reward received $r(t)$ and the experimentally recorded PL-NAc choice-selective sequential input $f_i(t)$. The PL-NAc input provides the critic with a representation of the chosen side on the current trial as well as the time during the trial. This allows the critic to output an appropriately timed value signal (and consequently an appropriately timed RPE signal) corresponding to the chosen side. The reward input acts as a feedback signal to the critic that provides information about the correctness of the chosen action.

To map the critic to a biological neural circuit, we hypothesize that NAc, together with VP and associated cortical areas, form the critic recurrent neural network (Figure 6A; Atallah et al., 2007; Lau and Glimcher, 2008; O’Doherty et al., 2004; Richard et al., 2016; Tsutsui et al., 2016). The choice-selective sequential input $f_i(t)$ to the critic is provided by the recorded choice-selective sequential activity in PL-NAc neurons (Figure 6A).

The reward prediction error (RPE)

As in the synaptic plasticity model (Figure 5A), the RPE $\delta(t)$ is computed in the VTA DA neurons based on the value signal from the critic network (Figure 6A).

$$\delta(t) = r(t) + \gamma V(t) - V(t-\Delta t) \qquad \text{(Equation 22)}$$

Unlike the synaptic plasticity model, the RPE signal is conveyed by the VTA dopamine neurons to the actor network. Note that the delay of the negative value signal equals one time step $\Delta t = 0.1s$ in this model, rather than the smaller delay $\Delta = 0.01s$ for the synaptic plasticity model. This is because the neural dynamics model used a larger time step for simulations due to limitations in computational power.

Actor network

In contrast to the synaptic plasticity model, in which actions were directly readout from the activity of the value neurons early in the trial, we consider an explicit actor network that generates actions. The actor is modeled as a network of LSTM units that compute the policy, i.e., the probability of choosing an action $a(t)$ at time step t given the current state of the network. The policy is represented by three policy readout neurons, corresponding to choosing left, right or 'do nothing', whose activities are given by a (trainable) linear readout of the activities of the actor LSTM units. The actor receives three inputs: (i) an efference copy of the action taken at the previous time step $a(t-\Delta t)$, (ii) a 'temporal context' input $\xi(t)$, encoded as a vector of all 0s except for a value of 1 in the entry corresponding to the current time point t, that provides the actor with a representation of the time within the trial, and (iii) the reward prediction error at the current time step $\delta(t)$.

To map the actor to a biological neural circuit, we hypothesize that the DMS and associated cortical areas form the actor recurrent neural network (Figure 6A; Atallah et al., 2007; O'Doherty et al., 2004; Seo et al., 2012; Tai et al., 2012). The temporal sequence input $\xi(t)$ to the actor is assumed to originate in the hippocampus or other cortical areas (Figure 6A; Hahnloser et al., 2002; Howard and Eichenbaum, 2013; Kozhevnikov and Fee, 2007; Zhou et al., 2020).

Training algorithm

To train the recurrent weights of the network, which are then held fixed during task performance, we implement the Advantage Actor-Critic algorithm (Mnih et al., 2016) on a slightly modified version of the reversal learning task (see "Block structure for training" section below). In brief, the weights of the neural network are updated via gradient descent and backpropagation through time. The loss function for the critic network, $\mathcal{L}_{critic}$, defines the error in the estimated value function. The synaptic weight parameters θ_v of the critic network are updated through gradient descent on the critic loss function $\mathcal{L}_{critic}$:

$$\Delta\theta_v = -\alpha\nabla\mathcal{L}_{critic}$$
$$\nabla\mathcal{L}_{critic} = -\beta_v\delta_t(s_t;\theta_v)\frac{\partial V}{\partial\theta_v} \quad \text{(Equation 23)}$$

where α is the learning rate, s_t is the state at time step t, V denotes the value function and β_v is the scaling factor of the critic loss term. $\delta_t(s_t;\theta_v)$ is the k-step return temporal difference error (not to be confused with the RPE input to the actor defined in Equation 22) defined as follows:

$$\delta_t(s_t;\theta_v) = R_t - V(s_t;\theta_v)$$

where R_t is the discounted k-step bootstrapped return at time t

$$R_t = \sum_{i=0}^{k-1}\left(r_{t+i}\prod_{j=0}^{i}\gamma_{t+j}\right) + V(s_{t+k};\theta_v)\prod_{j=0}^{k}\gamma_{t+j}$$

where r_t is the reward received at time step t, γ_t is the discount factor at time step t (defined below), and k is the number of time steps until the end of an episode.

The loss function for the actor network, $\mathcal{L}_{actor}$, is given by a weighted sum of two terms: a policy gradient loss term, which enables the actor network to learn a policy $\pi(a_t|s_t)$ that approximately maximizes the estimated sum of future rewards $V(s_t)$, and an entropy regularization term that maximizes the entropy of the policy π to encourage the actor network to explore by avoiding premature convergence to suboptimal policies. The gradient of the actor loss function $\mathcal{L}_{actor}$ with respect to the synaptic weight parameters of the actor network, θ, is given by.

$$\Delta\theta = -\alpha\nabla\mathcal{L}_{actor}$$
$$\nabla\mathcal{L}_{actor} = -\frac{\partial\log\pi(a_t|s_t;\theta)}{\partial\theta}\delta_t(s_t;\theta_v) - \beta_e\frac{\partial H(s_t;\theta)}{\partial\theta} \quad \text{(Equation 24)}$$

where a_t is the action at time step t, π is the policy, β_e is the scaling factor of the entropy regularization term and $H(s_t;\theta)$ is the entropy of the policy π

$$H(s_t;\theta) = -\sum_{a\in A}\pi(a|s_t;\theta)\log\pi(a|s_t;\theta)$$

where A denotes the space of all possible actions.

Neural dynamics model implementation

LSTM

Both the actor and critic LSTM networks consisted of 128 units each and were implemented using TensorFlow's Keras API. The weight matrices $\boldsymbol{U_q}$ were initialized using Keras's 'glorot_uniform' initializer, the weight matrices $\boldsymbol{W}_q$ were initialized using Keras's 'orthogonal' initializer and the biases $\boldsymbol{b}_q$ were initialized to 0. The output and memory states for both LSTM networks were initialized to zero at the beginning of each training or testing episode.

PL-NAc inputs to the critic

Input to the critic was identical to the smoothed, single-trial input used for the synaptic plasticity model described above, except i) activity was not interpolated because each time step in this model was equivalent to the sampling rate of the collected data (10 Hz), and ii) we chose to input only the activity from 2s before to 2s after the lever press (as compared to 3s after the lever press for the synaptic plasticity model) in order to reduce the computational complexity of the training process. To reduce episode length, and therefore training time, we also excluded those neurons whose peak activity occurred more than 2s after the lever press, reducing the final number of 'pseudoneurons' used as input to 306 (compared with 368 for the synaptic plasticity model).

Optogenetic-like stimulation of the PL-NAc population (Figures 7D and 7E) was performed in a similar manner to the synaptic plasticity model, with activity set to 0.15 for a randomly selected 70% of neurons for the duration of the trial.

Trial structure

Each trial was 4s long starting at 2s before lever press and ending at 2s after lever press. At any given time, the model has three different choices: choose left, choose right or do nothing. Similar to the synaptic plasticity model, the model makes its decision to choose left or right at the start of a trial, which then leads to the start of the corresponding choice-selective sequential activity. However, unlike the synaptic plasticity model, the model can also choose 'do nothing' at the first time step, in which case an activity pattern of all zeros is input to the critic for the rest of the trial. For all other time steps, the correct response for the model is to 'do nothing'. Choosing 'do nothing' on the first time step or choosing something other than 'do nothing' on the subsequent time steps results in a reward $r(t)$ of -1 at that time. If a left or right choice is made on the first time step, then the current trial is rewarded based on the reward probabilities of the current block (Figure 1A) and the reward input $r(t)$ to the critic is modeled by a truncated Gaussian temporal profile centered at the time of the peak reward (Equation 20) with the same parameters as in the synaptic plasticity model.

Block structure for training

We used a slightly modified version of the reversal learning task performed by the mice in which the block reversal probabilities were altered in order to make the block reversals unpredictable. This was done to discourage the model from learning the expected times of block reversals based on the number of rewarded trials in a block and to instead mimic the results of our behavioral regressions (Figure 1E) suggesting that the mice use only the previous ~4 trials to make a choice. To make the block reversals unpredictable, the identity of the high-probability lever reversed after a random number of trials drawn from a geometric distribution (Equation 1) with p = 0.9.

Training

Each training episode was chosen to be 15 trials long and the model was trained for 62,000 episodes. For this model, we used a time step $\Delta t = 0.1$s. The values of the training hyperparameters were as follows: the scaling factor of the critic loss term $\beta_v = 0.05$, the scaling factor of the entropy regularization term $\beta_e = 0.05$, the learning rate $\alpha = 0.01\text{s}^{-1}$($\alpha = 0.001$ per time step), and the timescale of temporal discounting within a trial $\tau = 2.5$s, leading to a discount factor $\gamma = e^{-\frac{\Delta t}{\tau}} = 0.96$ for all times except for the last time step of a trial when the discount factor was 0 to denote the end of a trial. The network's weights and biases were trained using the RMSprop gradient descent optimization algorithm (Hinton et al., 2012) and backpropagation through time, which involved unrolling the LSTM network over an episode (630 time steps).

Block structure for testing

Block reversal probabilities for the testing phase were the same as in the probabilistic reversal learning task performed by the mice. The average block length for the PL-NAc neural dynamics model was 19.3 ± 5.0 trials (mean+/-std. dev.).

Testing

The model's performance (Figures 6B–6J) was evaluated in a testing phase during which all network weights were held fixed so that reversal learning was accomplished solely through the neural dynamics of the LSTM networks. The network weights used in the testing phase were the weights learned at the end of the training phase. A testing episode was chosen to be 1500 trials long and the model was run for 120 episodes.

Actor network analysis

For Figures 6G–6J, we tested the model's performance on a slightly modified version of the reversal learning task in which, after training, block lengths were fixed at 30 trials. This facilitated the calculation and interpretation of the block-averaged activity on a given trial of a block. Dimensionality reduction of the actor network activity (Figure 6H) was performed using the *PCA* function from the decomposition module in Python's scikit-learn package.

Replacing sequential input to the critic with persistent input

In Figure 6F, we analyzed how model performance changed when the temporal structure provided by the choice-selective sequential inputs to the critic were replaced during training by persistent choice-selective input. The persistent choice-selective input was generated by setting the activity of all the left-choice selective neurons to 1 and all the right-choice selective neurons to 0 for all time points on left-choice trials and vice versa on right-choice trials.

Cross-trial analysis of RPE in dopamine neurons

To generate the regression coefficients in Figures 5G, 5L, 5Q, 6E, S10C, and S10D, we performed a linear regression analysis adapted from (Bayer and Glimcher, 2005), which uses the mouse's reward outcome history from the current and previous 5 trials to predict the average dopamine response to reward feedback on a given trial, i:

$$D(i) = \beta_0 + \sum_{j=0}^{5} \beta_j \widehat{R}(i-j) + error \quad \text{(Equation 25)}$$

where $D(i)$ is the average dopamine activity from 0.2 to 1.2s following reward feedback on trial i, $\widehat{R}(i-j)$ is the reward outcome j trials back from trial i (1 if j trials back is rewarded and 0 if unrewarded) and β_j are the calculated regression coefficients that represent the effect of reward outcome j trials back on the strength of the average dopamine activity, $D(i)$. For the regression coefficients generated from recorded dopamine activity (Figures S10C and S10D) we used the Z-scored GCaMP6f fluorescence from VTA-NAc terminal recordings of 11 mice performing the same probabilistic reversal learning task described in this paper (for details see Parker et al., 2016). The regression coefficients for the experimental data as well as the synaptic plasticity and neural dynamics model simulations were fit using the *LinearRegression* function from the linear_model module in Python's scikit-learn package.

Simulation of model-free versus model-based task performance

Overview

In order to identify possible RPE signatures that distinguish ideal observer ("model-based") versus Q-learning ("model-free") behavior in this task (Figure S14), we simulated choices using the two models. Based on the dopaminergic signature of block reversal inference reported in (Bromberg-Martin et al., 2010), we first confirmed that our ideal observer and Q-learning models gave rise to distinct dopamine signatures when performing the task used in (Bromberg-Martin et al., 2010). In that task, reward probabilities were 100% and 0% for the "high probability" and "low probability" choices, respectively, and the reward probabilities reversed with a 5% probability on each trial. Next, we applied the same framework to our task, to determine if we could observe similar distinctions between the models. In this case, the reward probabilities were 70% and 10%, as in the task studied in this paper, and blocks reversed with a 5% probability on each trial, which resulted in block lengths comparable to those observed in our experiments.

Ideal observer model

The ideal observer model was provided with knowledge of the reward probabilities associated with each block and the probability of block reversal on each trial. The 5% block reversal probability on each trial can be written in terms of the block state transition probabilities as

$$T_{ij} = P(s(t) = s_j | s(t-1) = s_i) = \begin{bmatrix} 0.95 & 0.05 \\ 0.05 & 0.95 \end{bmatrix} \quad \text{(Equation 26)}$$

where T_{ij} is defined as the transition probability between block state s_i on trial t and block state s_j on trial t+1. Here, 'block state' refers to whether the current block has a higher probability of left or right choices being rewarded. The reward probabilities for each block were as follows

$$R_{ik} = P(r(t) = 1 | s(t) = s_i, c(t) = c_k) = \begin{cases} \begin{bmatrix} 1.0 & 0.0 \\ 0.0 & 1.0 \end{bmatrix}, & \text{Bromberg} - \text{Martin Task} \\ \begin{bmatrix} 0.7 & 0.1 \\ 0.1 & 0.7 \end{bmatrix}, & \text{Our Task} \end{cases} \quad \text{(Equation 27)}$$

where R_{ik} is defined as the probability of reward for block state s_i and choice c_k.

On each trial, the ideal observer model selects the choice with the highest expectation of reward based on its belief about the current block state given the choice and reward history. The expectation of reward $\rho_l(t+1)$ for choice l on trial $t+1$, given the entire reward history $r(1{:}t)$ and choice history $c(1{:}t)$ up until trial t is given by

$$\begin{aligned} \rho_l(t+1) &= \sum_{i=1}^{2} R_{il} P(s(t+1) = s_i | r(1:t), c(1:t)) \\ &= \sum_{i=1}^{2}\sum_{j=1}^{2} R_{il} P(s(t+1) = s_i | s(t) = s_j) P(s(t) = s_j | r(1:t), c(1:t)) \\ &= \sum_{i=1}^{2}\sum_{j=1}^{2} R_{il} T_{ji} P(s(t) = s_j | r(1:t), c(1:t)) \end{aligned} \quad \text{(Equation 28)}$$

where l can be either 1 (left choice) or 2 (right choice) and $P(s(t) = s_j | r(1:t), c(1:t))$ is the probability of being in block state s_j on trial t given the entire reward and choice history up to and including trial t. Equation 28 tells us that estimating the block state probability $P(s(t) = s_j | r(1:t), c(1:t))$ will provide us with an estimate of the expected reward for a given choice on trial t+1 as R_{il} and T_{ji} are already known. Using Bayes' theorem, we can estimate the block state probability as

$$P(s(t)=s_j|r(1:t),c(1:t))=\frac{P(r(t)|r(1:t-1),c(1:t),s(t)=s_j)P(s(t)=s_j|r(1:t-1),c(1:t))}{\sum_{k=1}^{2}P(r(t)|r(1:t-1),c(1:t),s(t)=s_k)P(s(t)=s_k|r(1:t-1),c(1:t))} \quad \text{(Equation 29)}$$

The first term in the numerator of the right-hand side of Equation 29, $P(r(t)|r(1:t-1),c(1:t),s(t)=s_j)$, is the probability of receiving reward $r(t)$ (1 if rewarded and 0 if unrewarded) on trial t given the current choice $c(t)=c_k$, the block state s_j, and the reward history $r(1{:}t\text{-}1)$ and the choice history $c(1{:}t\text{-}1)$ up to trial t-1. Because the past history of rewards and choices does not affect the reward probability once the block state is known, this can be rewritten as

$$P(r(t)|r(1:t-1),c(1:t),s(t)=s_j)=P(r(t)|c(t)=c_k,s(t)=s_j)=R_{jk}^{r(t)}(1-R_{jk})^{1-r(t)} \quad \text{(Equation 30)}$$

The second term in the numerator of the right-hand side of Equation 29, $P(s(t)=s_j|r(1:t-1),c(1:t))$, is the probability that the current block state is s_j given the reward and choice history. This can be rewritten as

$$\begin{aligned}P(s(t)&=s_j|r(1:t-1),c(1:t))\\&=P(s(t)=s_j|r(1:t-1),c(1:t-1))\\&=\sum_{m=1}^{2}P(s(t)=s_j|s(t-1)=s_m)P(s(t-1)=s_m|r(1:t-1),c(1:t-1))\\&=\sum_{m=1}^{2}T_{mj}P(s(t-1)=s_m|r(1:t-1),c(1:t-1))\end{aligned} \quad \text{(Equation 31)}$$

In the second line above, the dependence on $c(t)$ has been removed because the choice on the current trial, in the absence of reward information on the current trial, does not provide any additional information about the current state beyond that provided by the past reward and choice history. Combining Equations 29–31, the block state probability on the current trial t can be written in terms of the known reward probabilities, known state transition probabilities and the previous block state probability as

$$P(s(t)=s_j|r(1:t),c(1:t))=\frac{\sum_{m=1}^{2}R_{jk}^{r(t)}(1-R_{jk})^{1-r(t)}T_{mj}P(s(t-1)=s_m|r(1:t-1),c(1:t-1))}{\sum_{l=1}^{2}\sum_{m=1}^{2}R_{lk}^{r(t)}(1-R_{lk})^{1-r(t)}T_{ml}P(s(t-1)=s_m|r(1:t-1),c(1:t-1))} \quad \text{(Equation 32)}$$

The above equation allows us to estimate the current trial block state probability $P(s(t)=s_j|r(1:t),c(1:t))$ recursively, since it can be expressed in terms of the previous trial block state probability $P(s(t-1)=s_m|r(1:t-1),c(1:t-1))$ and other known constant terms. This combined with the known reward and block transition probabilities allows the model to select the optimal choice according to Equation 28.

Q-learning model

To simulate trial-by-trial, model-free performance of the tasks, we used a Q-learning model in which the value of the chosen action is updated on each trial as follows:

$$\begin{aligned}Q_{right}(t+1)&=\begin{cases}Q_{right}(t)+\alpha(r(t)-Q_{right}(t)), & \text{if } c(t)=right\\ Q_{right}(t), & \text{if } c(t)=left\end{cases}\\ Q_{left}(t+1)&=\begin{cases}Q_{left}(t), & \text{if } c(t)=right\\ Q_{left}(t)+\alpha(r(t)-Q_{left}(t)), & \text{if } c(t)=left\end{cases}\end{aligned} \quad \text{(Equation 33)}$$

where Q_{right} is the value for the right choice and Q_{left} is the value for the left choice. t is the current trial and α is the learning rate, which was set to 0.612 per trial. $r(t)$ is the outcome of trial t (1 for reward, 0 for no reward). Q-values for each choice were initialized to 0. The outcome $r(t)$ was determined based on the reward probability for choice $c(t)$ given the block. Choice was simulated using a softmax equation such that the probability of choosing right or left is given by,

$$\begin{aligned}p(c(t)=right)&=\frac{\exp(\beta_{value}Q_{right}(t)+\beta_{stay}I_{right}(t))}{\exp(\beta_{value}Q_{right}(t)+\beta_{stay}I_{right}(t))+\exp(\beta_{value}Q_{left}(t)+\beta_{stay}I_{left}(t))}\\ p(c(t)=left)&=\frac{\exp(\beta_{value}Q_{left}(t)+\beta_{stay}I_{left}(t))}{\exp(\beta_{value}Q_{right}(t)+\beta_{stay}I_{right}(t))+\exp(\beta_{value}Q_{left}(t)+\beta_{stay}I_{left}(t))}\end{aligned} \quad \text{(Equation 34)}$$

Where β_{value} is the inverse temperature parameter, which was set to 0.99. β_{stay} is a parameter accounting for how likely mice were to repeat their previous choice, which was set to 0.95. $I_{\text{left/right}}$ is 1 if that action (i.e., left or right) was chosen on the previous trial and 0 otherwise. Parameters for the Q-learning model were fit in (Lee et al., 2019) to the behavior of mice in which dopamine neuron activity was recorded in Parker et al. (2016).

Comparison of RPE at block reversals

RPE for both the ideal-observer model and the Q-learning model (Figure S14) was defined as the difference between the experienced reward $r(t)$ and the expected reward for the chosen action ($\rho_{chosen}(t)$ for the ideal-observer model or $Q_{chosen}(t)$ for the Q-learning model) as follows:

$$\begin{aligned} RPE_{Ideal\ Observer} &= r(t) - \rho_{chosen}(t) \\ RPE_{Q-learning} &= r(t) - Q_{\mathrm{chosen}}(t) \end{aligned} \quad \text{(Equation 35)}$$

To identify RPE signatures of model free versus model based performance of the two tasks, we compared the RPE from the ideal-observer model and the Q-learning model on trials around block reversals. Specifically, we compared the RPE from the two models on the first trial of a block with the RPE on the second trial of a block when the choice on trial 1 was different from the choice on trial 2. This means that any changes in RPE from trial 1 to trial 2 were inferred because the new action-outcome relationship for the choice made on trial 2 had not been explicitly experienced in the new block.

Cell Reports, Volume *39*

Supplemental information

Choice-selective sequences dominate in cortical relative to thalamic inputs to NAc to support reinforcement learning

Nathan F. Parker, Avinash Baidya, Julia Cox, Laura M. Haetzel, Anna Zhukovskaya, Malavika Murugan, Ben Engelhard, Mark S. Goldman, and Ilana B. Witten

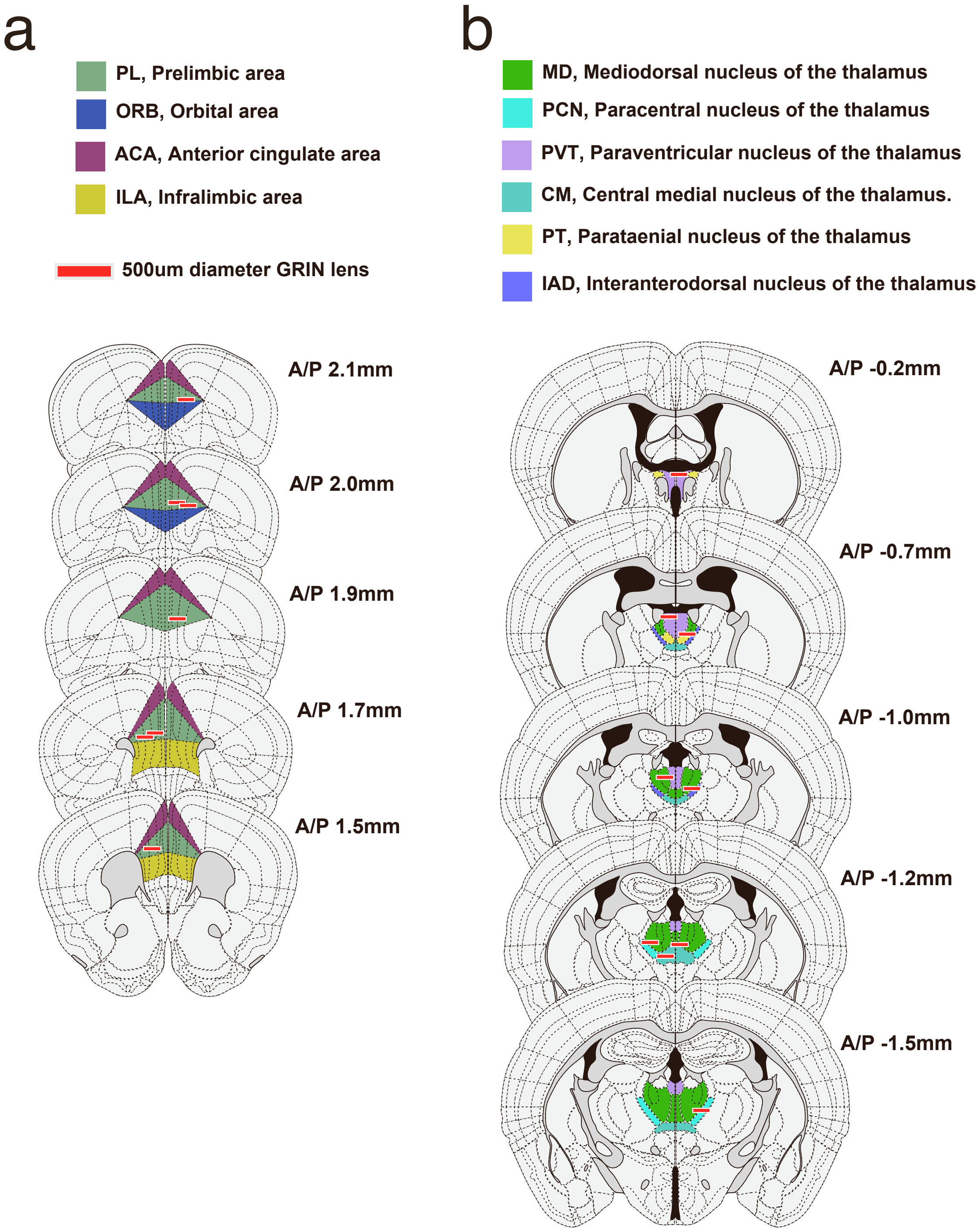

Supplementary Figure 1 - Locations of GRIN lens implants. Related to STAR METHODS. (a) Schematic of coronal sections along the anterior/posterior axis (A/P, numbers relative to bregma) with recording locations of 7 PL-NAc mice. Red lines indicate bottom of lens implant. **(b)** Same as **a** except location of 9 mTH-NAc recordings.

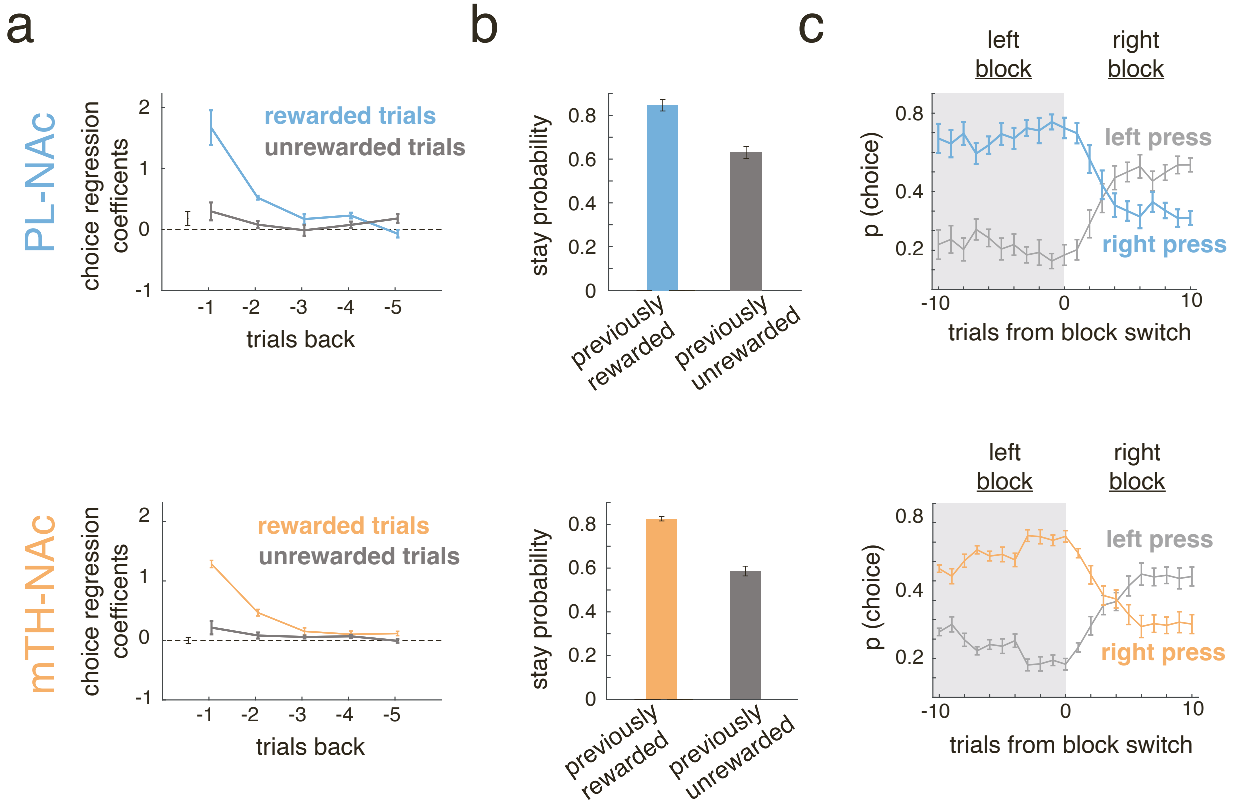

Supplementary Figure 2 - Mice in the PL-NAc and mTH-NAc imaging cohorts have comparable behavior. Related to Figure 1 and STAR METHODS. (a) Top**,** coefficients from logistic regression to predict choice (see **Figure 1**) from PL-NAc imaging cohort (n=7 mice). Bottom, same except coefficients from mTH-NAc imaging cohort (n=9 mice). Both cohorts use choice and outcome information from previous trials to predict the current choice. Regression coefficients between the two cohorts are not significantly different for any trials back for either rewarded or unrewarded trials (P>0.01, unpaired, two-tailed t-test of regression coefficients across mice at each trial back, n=7 and 9 mice for PL-NAc and mTH-NAc, respectively). **(b)** Stay probability following rewarded (blue or orange) and unrewarded (grey) trials for PL-NAc (top) and mTH-NAc (bottom) cohorts. Both cohorts have a significantly higher stay probability following a rewarded trial (PL-NAc: P=0.00008; mTH-NAc: P=0.00003, paired, two-tailed t-test comparing stay probability on rewarded and unrewarded trials across mice, n=7 and 9 mice for PL-NAc and mTH-NAc, respectively). **(c)** Probability of a left or right lever press following a reversal from a left-preferring to right-preferring block of mice from the PL-NAc (top, n=7 mice) and mTH-NAc (bottom, n=9 mice) cohorts. Both cohorts display a qualitatively similar change in choice behavior following a block reversal. In all panels, data are represented as mean +/- SEM across mice.

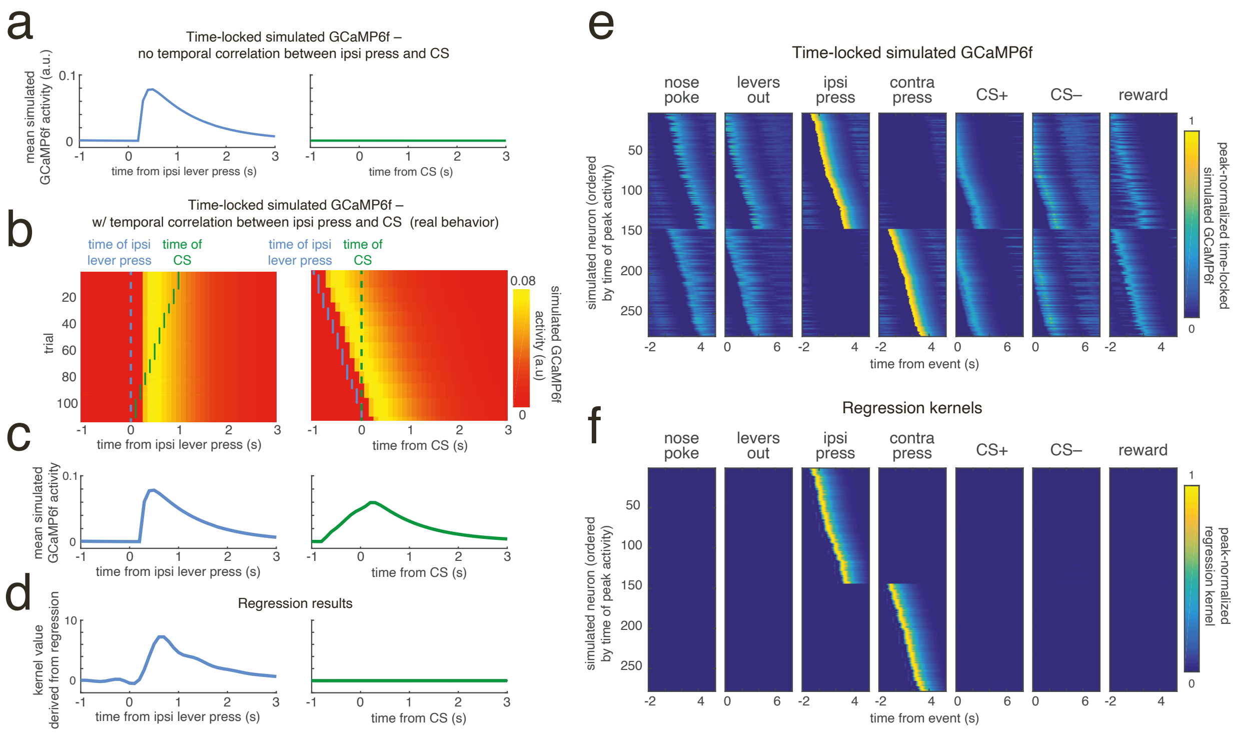

Supplementary Figure 3 - Simulated neural activity to illustrate the ability of the encoding model to successfully relate neural activity to the appropriate behavioral event. Related to Figure 2 and STAR METHODS. **(a)** Simulated neuron that is responsive only to the ipsilateral lever press. **(b)** Trial-by-trial heatmap of a simulated neuron that has increased activity time-locked to the ipsilateral lever press. In the data, there was a correlation between the time of lever press and the time of the CS, which produced a time-locked response to the CS, even though the neuron did not respond to that event. Left, activity heatmap aligned to the time of an ipsilateral lever press (dashed blue line) sorted by the time of the subsequent CS presentation (green dots). Right, activity heatmap is aligned to the time of the CS (dashed green line), ordered by the time of the preceding lever press (blue dots). **(c)** Average activity across trials of the example simulated neuron in **b** aligned to the lever press, left, and CS presentation, right. Unlike the idealized case in **a**, when the timing of task events is maintained from the real behavior, the temporal correlations result in a bump in activity aligned to the CS (right plot). Note that this bump in activity is generated entirely by the correlation in event times, since this simulated neuron only had activity in relation to the lever press (and not the CS presentation). **(d)** Response kernels for lever press, left, and CS, right, derived from the encoding model used to attribute the neural response of individual task events. The model successfully recovers the fact that neural activity in this simulated neuron is related to the lever press and not the CS. **(e)** Heatmap displaying the average activity from a population of 278 simulated neurons that respond to either the ipsilateral or contralateral lever press, but not the other events. Each neuron responds to the lever press, with a randomly assigned response latency from -1 to 3s. While the strongest average time-locked response is to the ipsilateral or contralateral lever presses, there are visible responses to the other task events as a consequence of the correlation between task events resulting from their temporal proximity. **(f)** Same as **e** except heatmap displays the response kernels derived from the encoding model. The model successfully discovers the underlying structure of the data (i.e., that responses are driven by the lever press).

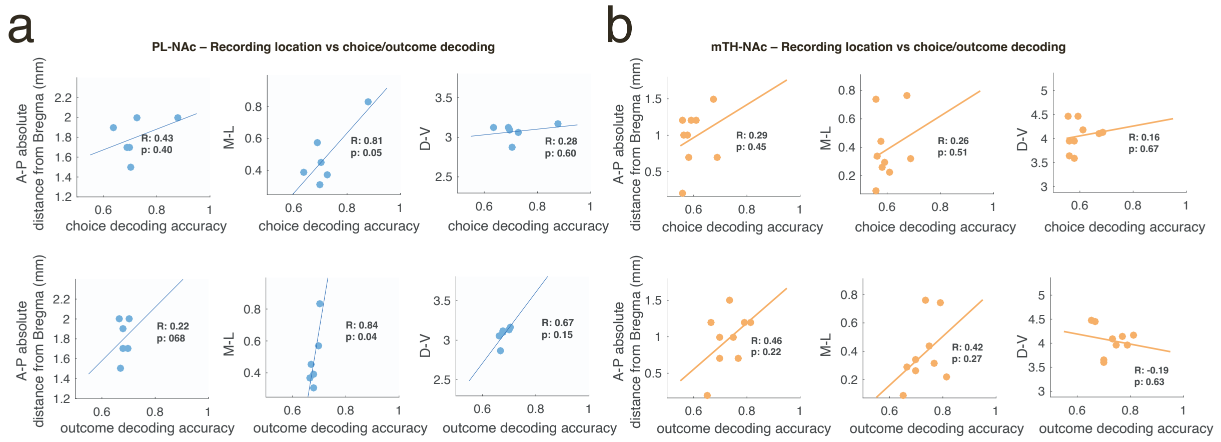

Supplementary Figure 4 - Lack of correlation between recording locations relative to Bregma and choice / outcome decoding. Related to Figure 3. (a) Top, correlations between choice decoding accuracy using recorded PL-NAc activity and, in order, the anterior/posterior (A/P), medial/lateral (M/L) and dorsal/ventral (D/V) recording locations relative to Bregma (see **Supplemental Figure 1** for schematic of recording locations; recording locations were aligned to the Allen atlas using the Wholebrain software suite (http://www.wholebrainsoftware.org/) of Furth et al. (2018); see Methods for details, n=6 mice). Bottom, same as top except correlation between recording location and outcome decoding accuracy using PL-NAc activity. **(b)** Same as **a** except decoding accuracy for choice (top) and outcome (bottom) determined using recorded mTH-NAc activity (n=9 mice). All p-values are calculated from Pearson's correlation coefficient; none are significant at the $p<.05$ level after correction for multiple (6) hypotheses using Bonferroni correction.

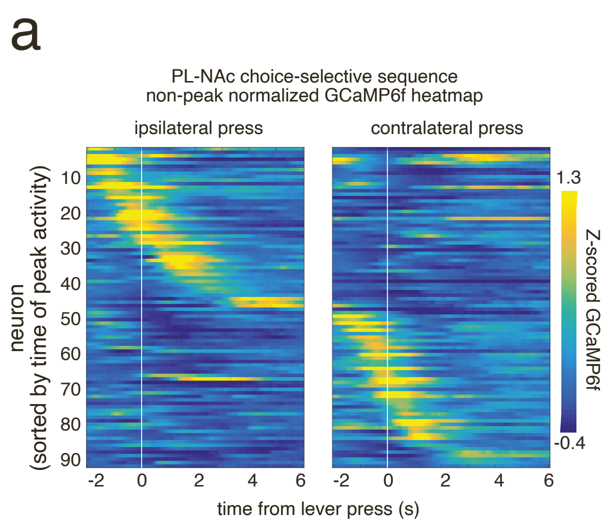

Supplementary Figure 5 - Choice-selective sequences in PL-NAc neurons without peak-normalization. Related to Figure 4. Heatmap demonstrating sequential response of choice-selective PL-NAc neurons to the ipsilateral and contralateral lever press (n=92 neurons from 7 mice). Similar to **Figure 4b-c**, but time-locked, trial-averaged GCaMP6f fluorescence is not normalized by the peak response to the lever press and is taken from all trials.

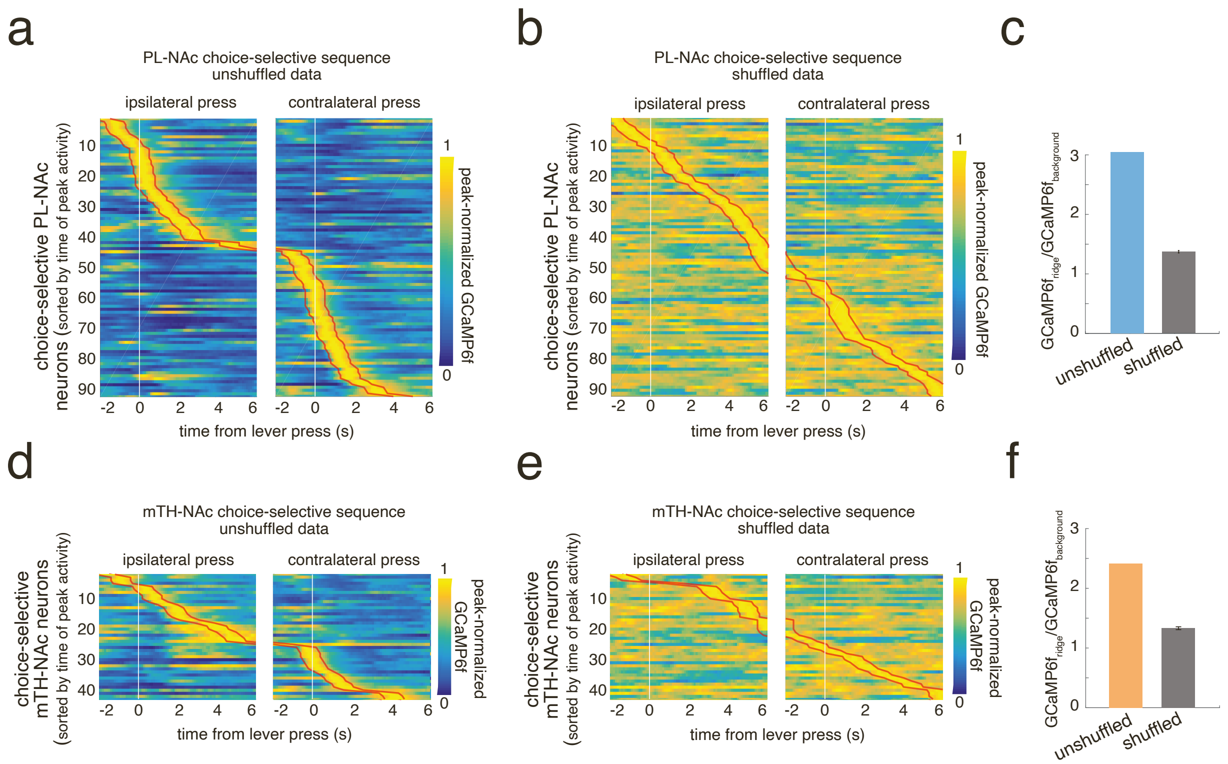

Supplementary Figure 6 – The calculated ridge-to-background ratio of PL-NAc neurons supports the presence of sequences. Related to Figure 4. (a) Sequential activity of PL-NAc choice-selective neurons. Similar to **Figure 4b-c,** the heatmap is ordered by the time of peak activity time-locked to the ipsilateral (left column) and contralateral (right column) lever press of each neuron**,** but instead of cross-validation, activity is averaged across all trials. Red trace represents the borders of the one-second window around the peak defined as the 'ridge'. Activity at all other surrounding timepoints is considered the 'background'. **(b)** Same as **a** for data that is shuffled by temporally shifting the GCaMP6f fluorescence trace across a recording session separately for each neuron by a random number of frames, chosen from a uniform distribution. Ordering by the time of peak activity generates spurious sequential activity across the diagonal in shuffled data. **(c)** Calculated ridge-to-background ratio of PL-NAc neurons using unshuffled (blue) and shuffled (grey) data. A ratio is calculated for each individual neuron and the average of these ratios across all neurons displayed in the heatmap is shown. The ratio calculated from unshuffled data is significantly larger than that from the shuffled data (P<0.0001, comparison between unshuffled data and distribution of 500 shuffled iterations). Error bars for shuffled data indicate one standard deviation. **(d-f)** Same as **a-c** but ridge-to-background is calculated using mTH-NAc neural recordings. Similar to PL-NAc, the ratio calculated from unshuffled data was significantly larger than that from the shuffled data (P<0.0001). However, when comparing across the populations, the ridge-to-background calculated using PL-NAc neurons (3.06+/-0.12, mean+/-sem, n=92 neurons from 7 mice) was significantly larger than that using mTH-NAc (2.42+/-0.12, mean+/-sem, n=42 neurons from 9 mice; P=0.004: unpaired, two-tailed t-test comparing ratio between PL-NAc and mTH-NAc neurons).

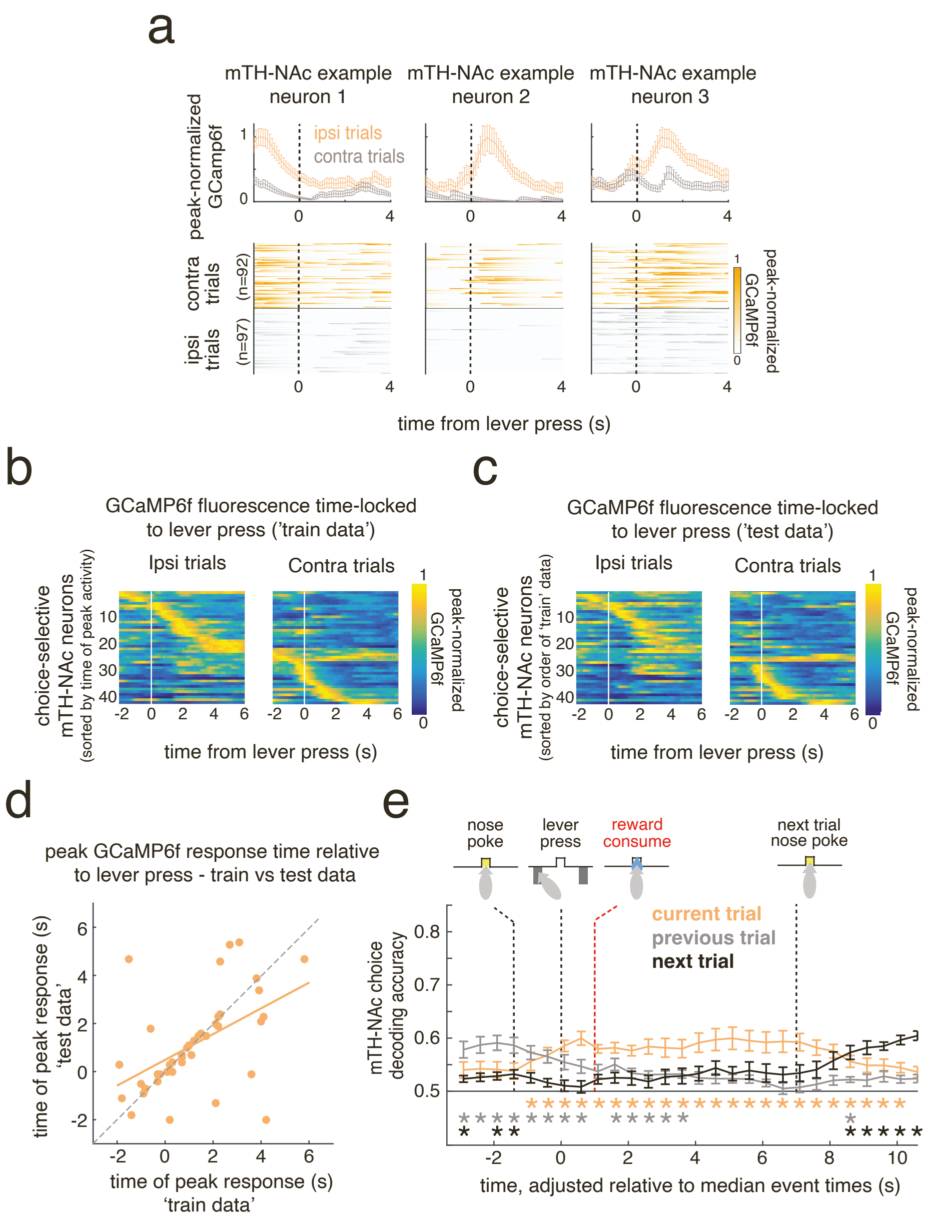

Supplementary Figure 7 - mTH-NAc choice-selective neurons display sequential activity that is less consistent than PL-NAc. Related to Figure 4. (a) Top; average GCaMP6f fluorescence of three simultaneously imaged mTH-NAc choice-selective neurons with different response times relative to the lever press. Error bars are s.e.m across trials. Bottom, heatmaps of GCaMP6f fluorescence response across trials to ipsilateral (orange) and contralateral (grey) lever presses. **(b,c)** Heatmaps of choice-selective mTH-NAc neurons' peak-normalized GCaMP6f responses to lever press (n=42/256 neurons from 9 mice). Each row is the average GCaMP6f fluorescence time-locked to the ipsilateral (left column) and contralateral (right column) lever press for a neuron, normalized by the neuron's peak average fluorescence. In **b** ('train data'), heatmap is generated using a randomly selected half of trials and ordered by the time of each neuron's peak activity. In **c** ('test data'), the peak-normalized, time-locked GCaMP6f fluorescence from the other half of trials was used while maintaining the order from 'train data' in **b**. Compare to PL-NAc data in **Figure 4b-c**. **(d)** Correlation between the time of peak activity using the 'train' (horizontal axis) and 'test' (vertical axis) trials for choice-selective mTH-NAc neurons. While mTH-NAc choice-selective neurons also show significant correlation between 'train' and 'test' trials ($R^2 = 0.51$, $P = 5.5 \times 10^{-4}$, n=42 neurons from 9 mice), this correlation is significantly lower than that of PL-NAc (comparison with data in **Figure 4d**; P=0.005, Z=2.81, Fisher's R-to-Z transformation, comparison of correlation coefficients derived from comparing peak activity

between ‘test’ and ‘training’ data from PL-NAc versus mTH-NAc). **(e)** Average choice decoding accuracy of the mice’s choice on the current (orange), previous (grey) and next trial (black) as a function of GCaMP6f fluorescence throughout the current trial. GCaMP6f fluorescence is taken from 100 random selections per mouse of 10 simultaneously imaged mTH-NAc neurons (each trial’s activity is adjusted in a piecewise linear manner relative to the median time of the nose poke, lever press and next trial nose poke, see Methods for details). Data are represented as mean +/- SEM across mice (n=9 mice). Red dashed line indicates median onset of reward consumption. * indicate significant decoding accuracy above chance, $P<0.01$, two-tailed, one-sample t-test across mice.

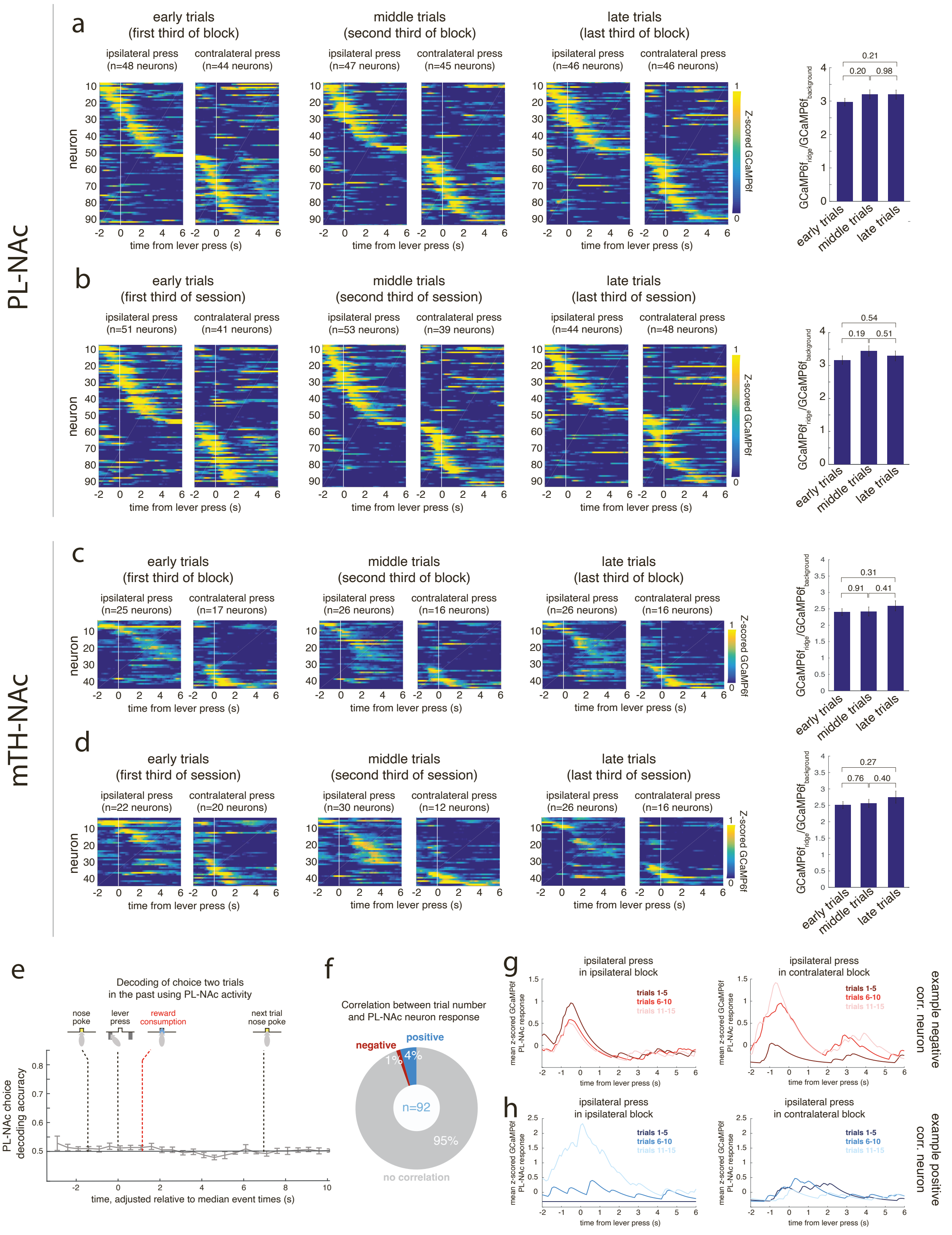

Supplementary Figure 8 – Trial number within a block, and two-trial-back choice, are not strongly encoded by PL-NAc activity. Related to Figure 4. (a) Left, heatmaps of Z-scored GCaMP6f activity from 92 choice-selective PL-NAc neurons averaged across the first, middle and last third of trials of each block. Right, calculated ridge-to-background ratio derived from average activity from each third of trials within a block. Data are represented as mean +/- SEM across neurons (n=92 from 7 mice). No significant changes are observed in the ratios calculated across a block, suggesting that the strength of sequences is not modulated by block trial number (p>0.05, paired,

two-tailed t-test). **(b)** Same as **a** except data is split into the first, middle and final third of the entire recording session. **(c,d)** Same as **a,b** except activity is of mTH-NAc choice-selective neurons. **(e)** Decoding accuracy for the mice's choice two trials back using activity from 10 simultaneously recorded PL-NAc neurons. Unlike choice decoding on the current and previous trial (**Figure 4e;** blue and black traces, respectively), PL-NAc activity is not able to accurately decode choice from two trials back after correcting for cross-trial choice correlations (see Methods for details) at any time point in the trial ($p>0.05$ for all time points: one-sample, two-tailed t-test across mice comparing decoding accuracy with chance rate of 0.5). **(f)** Proportion of PL-NAc choice-selective neurons whose activity is significantly positively (blue, n=4 neurons) or negatively (red, n=1 neuron) correlated with the number of trials into a block ($P<0.01$). Significance was determined by comparing the calculated correlation coefficient of each neuron to a null distribution of 500 correlation coefficients generated using GCaMP6f signal circularly shifted by a random integer, to control for slow drift in the data. R-values were calculated using the maximum GCaMP6f activity from 2s before to 6s after the time of lever press for the first 15 trials in a block. Using the same criteria, no mTH-NAc neurons were significantly correlated, either positively or negatively with trial number in a block ($p>0.05$). **(g)** Left, average activity of the negatively correlated PL-NAc neuron in response to an ipsilateral lever press at various trials in an ipsilateral block, where the ipsilateral lever had a higher probability of reward and, thus, the value of the ipsilateral lever increases as a function of trial number. Right, average activity of the negatively correlated PL-NAc neuron in response to an ipsilateral press in a contralateral block. **(h)** Same as **g** except for an example of a positively correlated PL-NAc neuron.

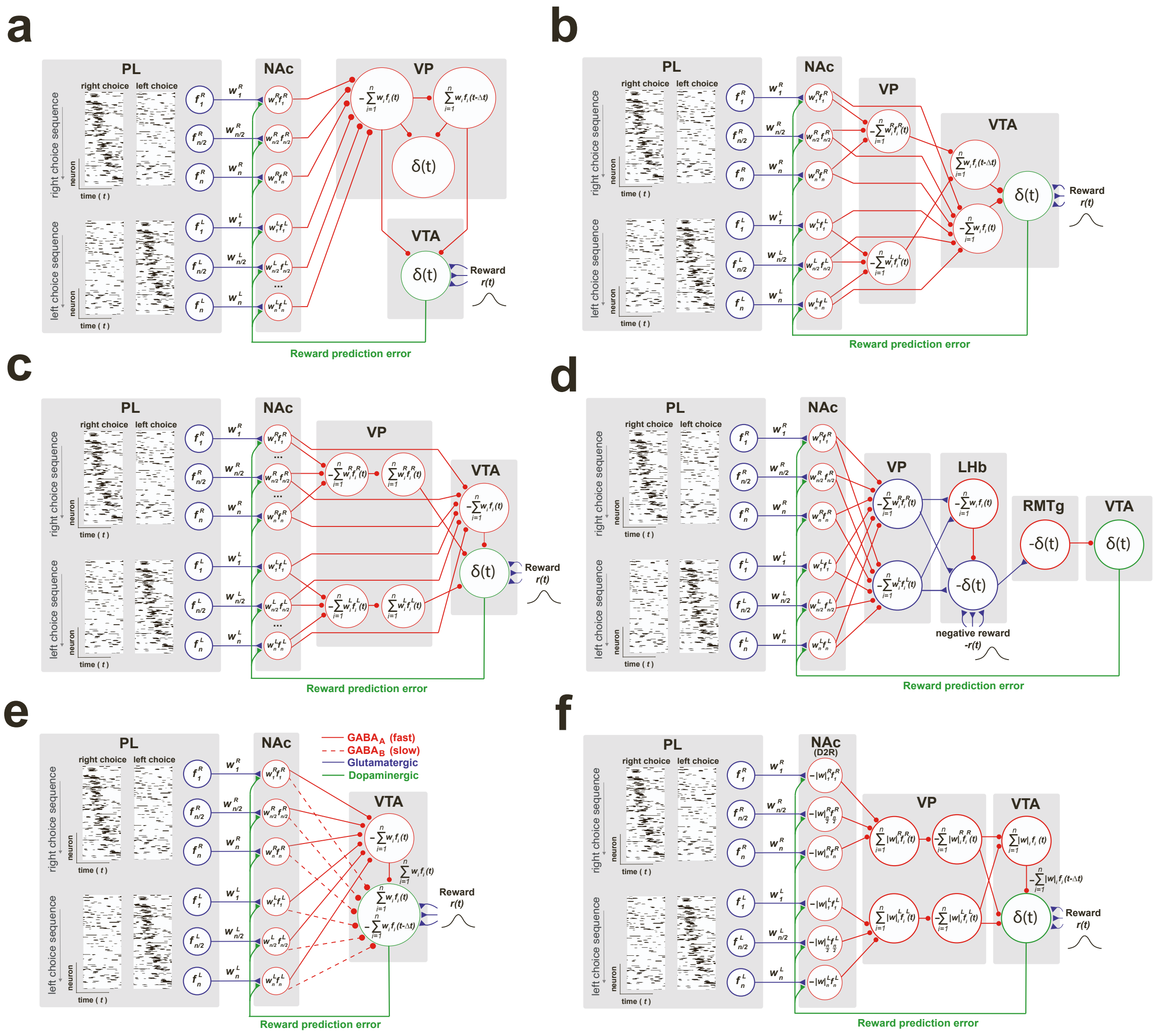

Supplementary Figure 9 - Alternative model architectures used to implement synaptic plasticity model. Related to Figure 5. (a-f) Alternative models constructed using known circuit architecture. All models except **a,d** generate an RPE signal by providing a 'fast excitatory' and 'slow-inhibitory' value pathway to the VTA dopamine neuron population. Note that all model variants rely on choice-selective sequences in PL-NAc to bridge the beginning of the action sequence and outcomes across time. For all model variants, GABAergic, glutamatergic and dopaminergic projections are denoted as red, blue and green, respectively. Brain region abbreviations are: prelimbic cortex, PL; nucleus accumbens, NAc; ventral pallidum, VP; ventral tegmental area, VTA; lateral habenula, LHb; rostromedial tegmental nucleus, RMTg. **(a)** In this model, the delay and inversion of the value signal is accomplished through a second VP neuron. These two VP neurons converge onto a third VP interneuron to generate an RPE signal in VP, as has been observed by (Ottenheimer et al., 2020). **(b)** In this model, the fast excitatory pathway is generated via a direct projection of NAc neurons onto the VTA GABA neuron while the slow inhibitory pathway passes through the VP before synapsing onto a VTA GABA neuron. **(c)** Similar to **b** except that the slow inhibitory pathway contains an additional VP neuron, which accomplishes the sign inversion and delay assigned to a VTA GABA neuron in **b**. Since the models in **b** prescribe a role for the observed NAc-D1R projections to VTA

GABA neurons, they produce negative value signals in VTA GABA neurons, whereas only positive value signals have been observed experimentally in identified GABA interneurons in the VTA (Cohen et al., 2012)**. (d)** In this model, a negative reward prediction error is calculated in the LHb using glutamatergic projections from the VP (Tooley et al., 2018), inversion and delay in the value signal from local inhibitory LHb neurons to produce an inverted RPE, which is then transmitted to the VTA via the RMTg (Webster and Wozny, 2020; Li et al., 2019). **(e)** To account for previous work describing direct projections from NAc D1R neurons to the VTA (Beier et al., 2015; Watabe-Uchida et al., 2012; Yang et al., 2018), this alternative model architecture has NAc neurons projecting directly to the VTA, skipping the VP. In this model, the timing difference needed to compute an RPE signal is generated through the activity of fast ionotropic GABA-A receptors (solid red trace), which have been shown to preferentially express in NAc-VTA GABA interneuron projection postsynaptic densities (Edwards et al., 2017), while activity of metabotropic GABA-B receptors (dashed red trace), which are preferentially expressed in the postsynaptic densities of NAc-VTA DA projections (Edwards et al., 2017), generate the slow-inhibitory pathway. Notably, without this differential expression of GABA receptors in the DAergic and GABAergic populations of the VTA, this model architecture would fail to produce an RPE signal, as it would instead generate a fast-inhibitory and slow-excitatory signal in the VTA DA neuron population. **(f)** Multiple studies have implicated D2-R expressing MSNs as playing a critical role in reversal learning in multiple mammalian species (Boulougouris et al., 2009; DeSteno and Schmauss, 2009; Eisenegger, 2014; Kruzich and Grandy, 2004; Kruzich et al., 2006; Kwak et al., 2014; Piray, 2011). Thus, in this model we account for the possibility that the reversal behavior in our task is mediated specifically by changes to synaptic weights from PL to D2-R-expressing NAc MSNs. This model assumes the opposite dopamine-mediated plasticity rule (LTD rather than LTP) than the previous models.

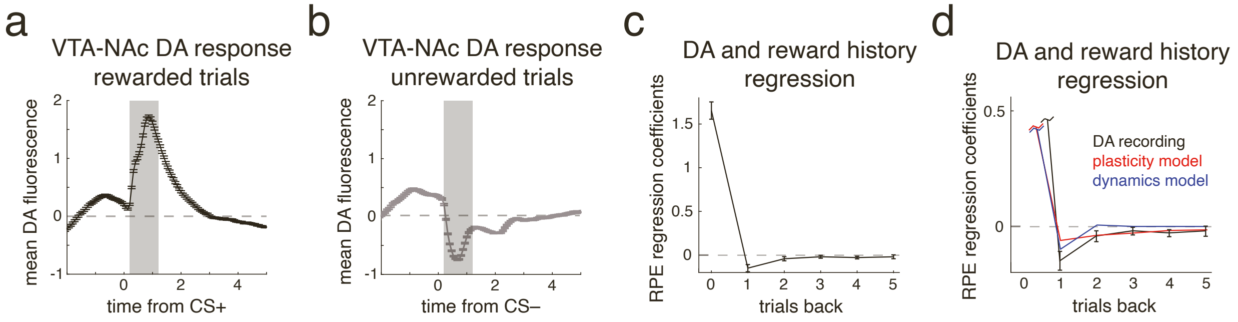

Supplementary Figure 10 - Reward prediction error (RPE) encoding observed in recorded dopamine (DA) activity is similar to that produced by our synaptic plasticity and neural dynamics models. Related to Figures 5 and 6. (a) Mean bulk GCaMP6f fluorescence from VTA-NAc DA terminals in response to a conditioned stimulus signaling reward (CS+, data taken from (Parker et al., 2016, n=11 recording sites). Note that terminal fluorescence recordings are presented here to more accurately reflect the signal that downstream NAc neurons are receiving in our model. **(b)** Same as **a** except DA fluorescence in response to the conditioned stimulus signaling an unrewarded trial (CS–). **(c)** Coefficients from a multiple linear regression in which outcome is predicted using mean DA fluorescence signals from 0.2-1.2s relative to the time of CS presentation across current ("0") and multiple previous trials (see shaded region in **a,b**), similar to **Figure 5g,l,q** and **Figure 6e**. The positive coefficient for the current trial and negative coefficients for previous trials indicate the encoding of an RPE. **(d)** Same as **c**, but also including coefficients from the synaptic plasticity model (red, same coefficients as **Figure 5g**) and the neural dynamics model (black, same coefficients as **Figure 6e**), to allow direct comparison. Error bars in all panels represent s.e.m across 11 recording sites.

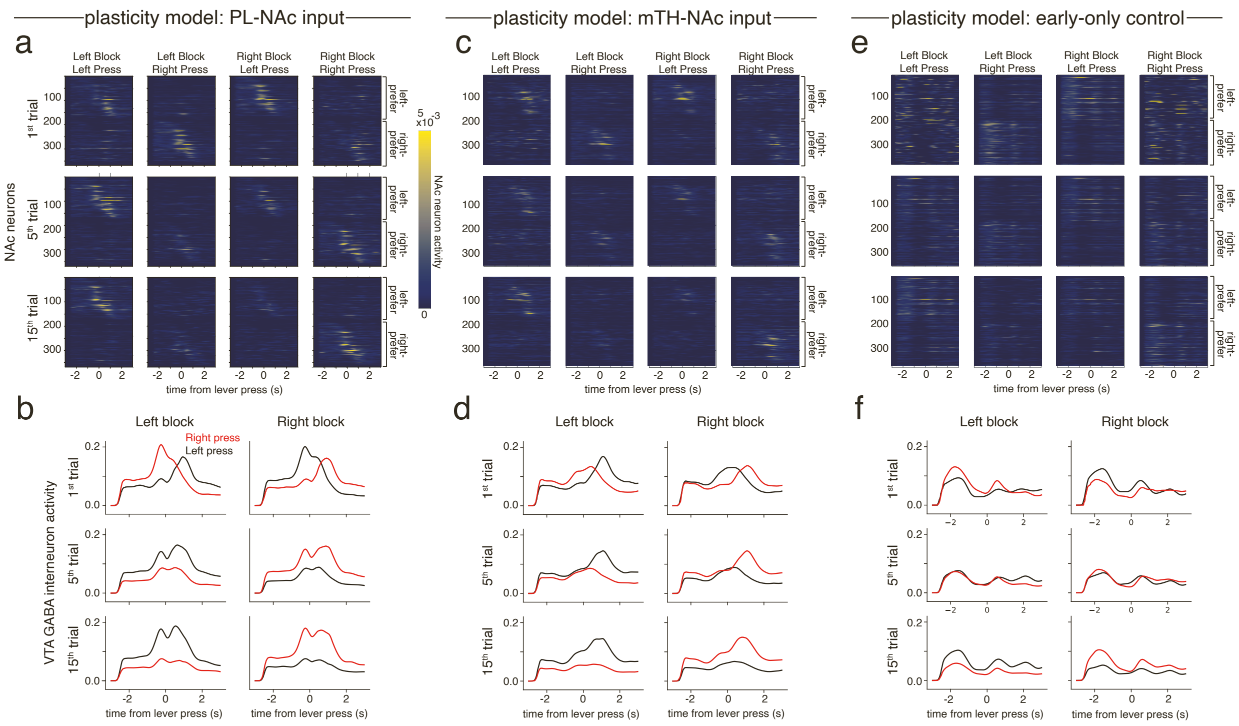

Supplementary Figure 11 - Synaptic plasticity model using sequential PL-NAc but not early-only or mTH-NAc activity correctly modulates activity in NAc projection neurons and VTA GABA interneurons. Related to Figure 5. (a) Heatmaps of average activity relative to the time of the lever press for NAc projection neurons in the PL-NAc model (**Figure 5c**). Top, middle and bottom heatmaps are the average activity across the first, fifth and fifteenth trial of each block, respectively. Each column is the average activity across trials from different block/press combinations. For each subplot, neurons 1-184 are left-preferring and neurons 185-368 are right-preferring. The activity of these left- and right-preferring NAc neurons increases throughout a block of their respective lever preference. In contrast, their activity decreases throughout a block opposite to their lever preference. **(b)** Average activity of VTA GABA interneuron from synaptic plasticity model using PL-NAc activity as input on left (black) or right (red) trials. Activity is relative to the time of the lever press across the first, fifth and fifteenth trials of a left-preferring (left column) or right-preferring (right column) block. Similar to **a**, throughout a left block (left column), the activity on left press trials increases from the first to fifteenth trial while the activity on right press trials decreases. The opposite pattern is seen for left and right press trials throughout a right block (right column). **(c,d)** Same as **a,b** except NAc and VTA GABA interneuron generated using mTH-NAc as input to the synaptic plasticity model. **(e,f)** Same as **a,b** except NAc and VTA GABA interneuron from the early-only control synaptic plasticity model.

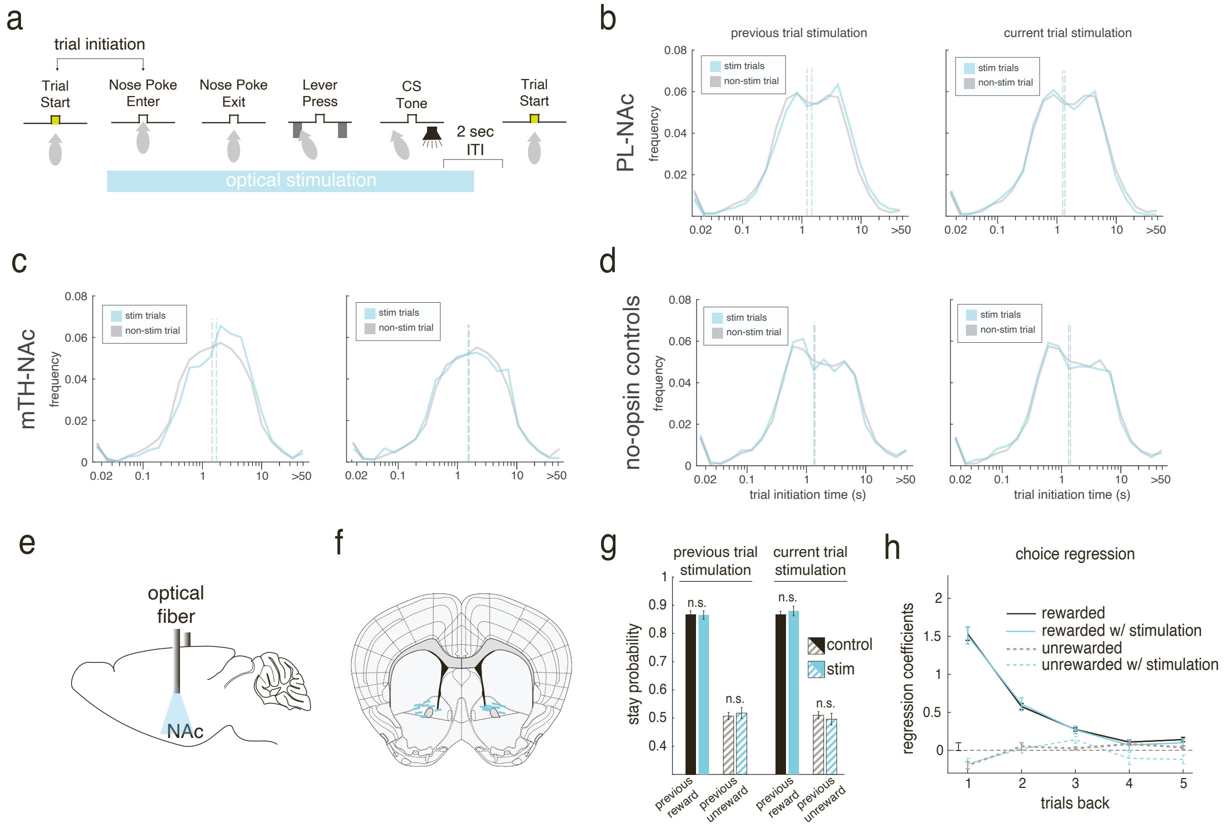

Supplementary Figure 12 – Laser stimulation affects trial initiation times in both PL-NAc and mTH-NAc but does not affect behavior in control mice that do not express opsin. Related to Figure 7. (a) Schematic of trial structure and time of optical stimulation. "trial initiation" time is defined as the latency between the start of the trial and the mouse entering the central nose poke. **(b)** Left, distribution of trial initiation times following stimulation trials (blue) and non-stimulation trials (grey) in PL-NAc ChR2 mice. Blue and grey vertical lines indicate median initiation times for stim and non-stim trials, respectively. Right, same only effect of current trial stimulation on trial initiation times. Previous trial stimulation resulted in significantly longer trial initiations in PL-NAc ChR2 mice than no-opsin control mice (P=3.46x10^-7, p-value from the 'opsin group X previous trial stimulation' interaction term of a mixed effects model used to predict latency times of PL-NAc and control mice, fit using the fitglme function in MATLAB; see Methods for additional model details). In contrast, current trial stimulation had no significant effect on initiation times (P=0.95, same test as above except p-value is that of the interaction term of 'opsin group X current trial stimulation'), an expected result as the start of stimulation was contingent on the mouse performing a nose poke. **(c)** mTH-NAc ChR2 mice had significantly longer trial initiation times following optical stimulation than no-opsin control mice (P=2.34x10^-4, p-value from the 'opsin group X previous trial stimulation' interaction term of a mixed effects model used to predict latency times of mTH-NAc and control mice) but no effect of current trial stimulation was observed (P=0.74, same test as above except the p-value is that from the 'opsin group X current trial stimulation' interaction term). **(d)** Same as **b,c** except latencies from no-opsin control cohort. **(e)** Surgical schematic of no-opsin control cohort. Optical fibers were implanted into the NAc. **(f)** Optical fiber tip locations of no-opsin control cohort (n=8 mice). **(g)** Unlike PL-NAc ChR2 expressing mice (**Figure 7f-h**), neither current nor previous trial stimulation changed the stay probability in control mice following rewarded (P=0.52: previous trial stimulation; P=0.24: current trial stimulation; paired t-test) or unrewarded trials (P=0.52: previous trial stimulation; P=0.47: current trial stimulation; paired t-test). **(h)** Likewise, stimulation from multiple trials back had no effect on choice (P>0.05 for all trials back, t-test across mice's laser x choice interaction term coefficients). Data in **g,h** are represented as mean +/- SEM across mice, n=8.

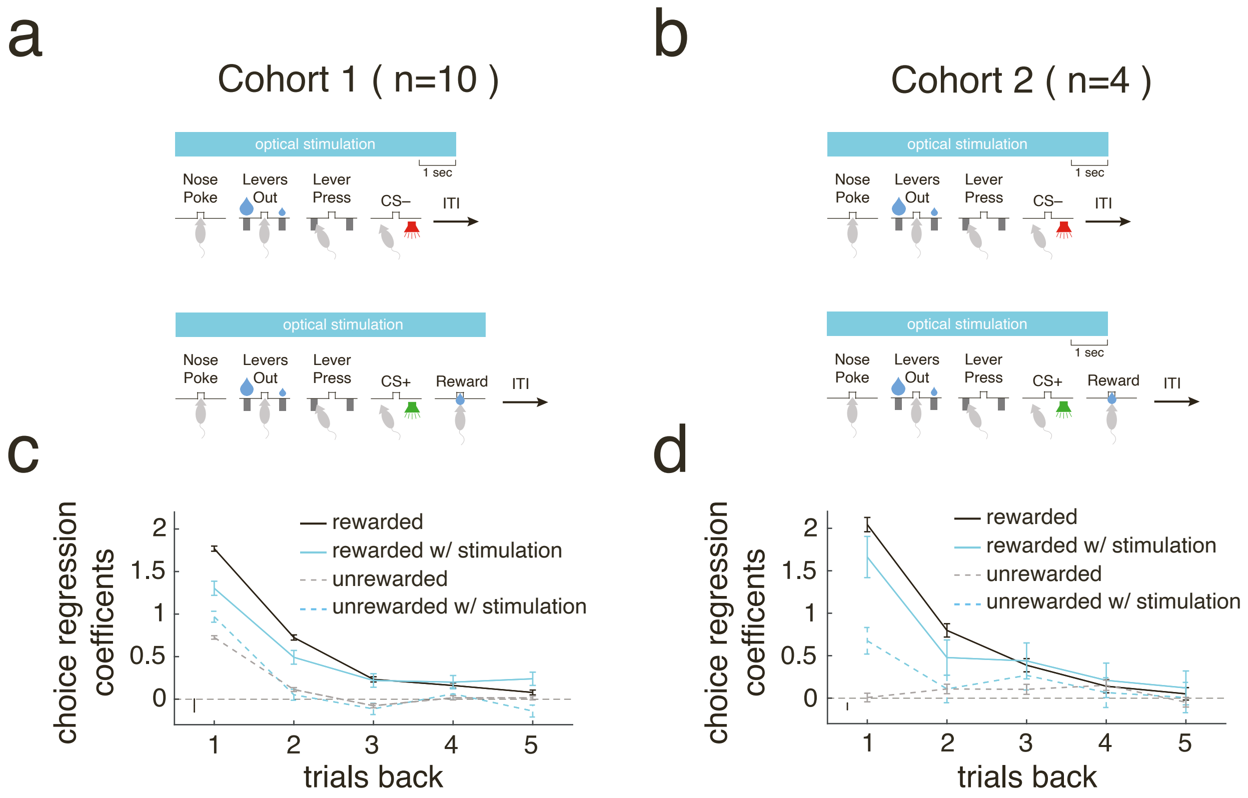

Supplementary Figure 13 - Effect of PL-NAc optogenetic stimulation in two cohorts. Related to Figure 7. (a) Schematic of optical stimulation parameters for cohort 1. On 10% of unrewarded trials, optical stimulation began when the mouse entered the central nosepoke and ended 1s into the intertrial interval (ITI), which began at the end of the 500ms CS– tone. On 10% of rewarded trials, stimulation began with nose poke and ended after the mouse left the reward port. **(b)** Schematic for cohort 2. Unlike cohort 1, optical stimulation ended on the same timescale on both rewarded and unrewarded trials, 1s after the end of CS presentation. **(c)** Logistic regression model similar to that in **Figure 1e** demonstrating the effect of PL-NAc stimulation on lever choice in cohort 1 mice (n=10 mice, see Methods for model details). Rewarded trials with stimulation one and two trials back decreased stay probability compared with rewarded trials without stimulation. Stimulation had an opposite effect on unrewarded trials, for which there was an increase in stay probability following stimulation one trial back compared to trials without stimulation. **(d)** Same as **c** except data from cohort 2 (n=4 mice). Effect of optical stimulation of PL-NAc neurons was qualitatively similar across the two cohorts. Data in **c,d** are represented as mean +/- SEM across mice.

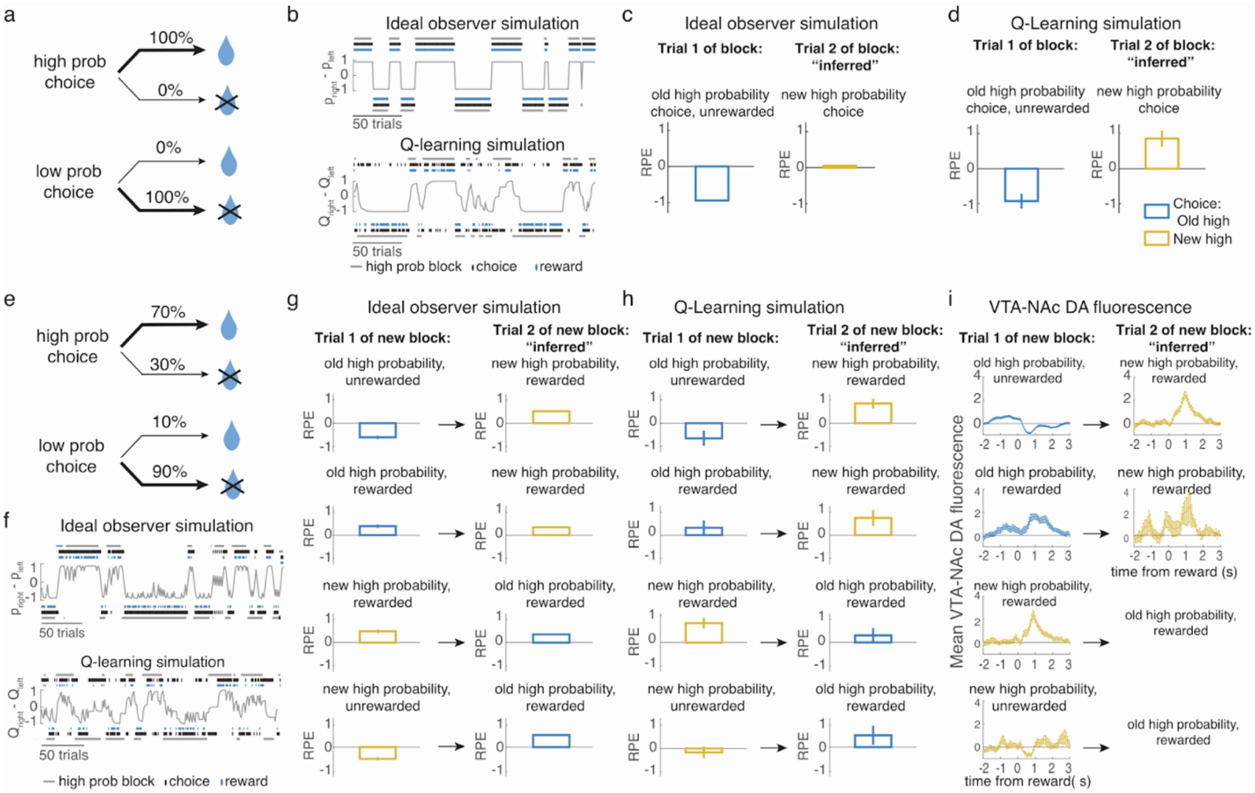

Supplementary Figure 14 - Similar RPE signatures for ideal observer and Q-learning simulation during reversal learning. Related to Figure 5 and STAR METHODS. Given previous evidence that dopamine signals can reflect knowledge of task structure (Bromberg-Martin et al., 2010; Sadacca et al., 2016), we used modeling to gain insight into how clearly RPE in the probabilistic reversal learning task (a bandit task) can indicate the use of model-based inference of block reversals for different reward probability structures. This was done by simulating task performance using an ideal observer model with knowledge of the block structure, and a Q-learning model which did not have information about the block structure (see Methods for more details). **(a-d)** To confirm that our ideal observer simulation captured a previously reported RPE signature of model-based block reversal inference, we first simulated behavior of 100,000 trials of a task similar to that used in (Bromberg-Martin et al., 2010). **(a)** In this task, one option was rewarded 100% of the time while the other was never rewarded, and the identity of the high probability choice randomly reversed with a probability of 0.05 on each trial. **(b)** Example performance of the ideal observer simulation (top) and the Q-learning simulation (bottom). Choice is determined by the difference between expected reward for the available actions, ρ, for the ideal observer and the difference between the action values, Q, for the Q-learning simulation (these values are plotted in grey). **(c)** To evaluate RPE signatures of model-based block reversal inference, we compared the estimated RPE (experienced reward minus expected reward for the chosen action) on trial 1 and trial 2 of the new block. The RPE on trial 2 was low for the high probability choice in the new block even without direct experience of that action-outcome pairing. This means that the ideal observer infers the block reversal, so the new, not yet experienced reward contingency is expected and the RPE is low. **(d)** In contrast, because the Q-learning model only updates the value of the chosen action, on trial 2, when the simulation is rewarded for the previously low-probability choice, the reward remains unexpected and the RPE is high. **e-i)** Simulated performance of 100,000 trials using the reward probabilities from this study. **(e)** The high probability action was rewarded 70% of the time while the low probability action was rewarded 10% of the time and the blocks reversed according to the same rule as in **a**. **(f)** Example performance of the ideal observer (top) and the Q-learning (bottom) simulations in this task. **(g)** To determine whether there is a strong qualitative RPE signature of block reversal inference in this task, we compared RPE on the 4 possible trial-1 types to RPE on the subsequent rewarded switch trials (i.e. choice on trial 2 was different than trial 1, meaning that any changes in RPE must be inferred). We focus on rewarded trials to aid comparison with reward responses recorded in dopamine terminals during this task (Parker et al., 2016). In this case, inference of the block reversal is not obviously reflected in the RPE, since the RPE for a given action on trial 1 and trial 2 are similar (comparing the same color bars for rewarded actions on trials 1

and 2). This is because, even though the ideal observer updates the predicted reward for both the chosen and unchosen actions, when reward delivery is probabilistic, predicted reward remains moderate for both actions and RPE changes only subtly. **(h)** Same as in **g** for the Q-learning simulation. As expected, RPE looks very similar on trial 1 and trial 2 for a given rewarded action because the Q-learning simulation does not update the value of the unchosen action on trial 1. **(i)** Consistent with the results from both simulations, GCaMP6f z-scored dF/F from dopaminergic axons in the NAc recorded in (Parker et al., 2016) is also very similar for a given rewarded action on trial 1 and trial 2. Note that the mice did not make all possible choices in this task, so some trial types are missing. The simulations also rarely made these choices (e.g., switch following a rewarded new high probability choice). Error bars in **c-h** are SEM across block transitions. Data in **i** are represented as mean +/- SEM across 11 recording sites).

Supplemental References

Boulougouris, V., Castañé, A., and Robbins, T.W. (2009). Dopamine D2/D3 receptor agonist quinpirole impairs spatial reversal learning in rats: investigation of D3 receptor involvement in persistent behavior. Psychopharmacology *202*, 611–620.

DeSteno, D.A., and Schmauss, C. (2009). A role for dopamine D2 receptors in reversal learning. Neurosci. *162*, 118–127.

Edwards, N.J., Tejeda, H.A., Pignatelli, M., Zhang, S., McDevitt, R.A., Wu, J., Bass, C.E., Bettler, B., Morales, M., and Bonci, A. (2017). Circuit specificity in the inhibitory architecture of the VTA regulates cocaine-induced behavior. Nat. Neurosci. *20*, 438–448.

Eisenegger, C., Naef, M., Linssen, A., Clark, L., Gandamaneini, P.K., Müller, U., and Robbins, T.W. (2014). Role of dopamine D2 receptors in human reinforcement learning. Neuropsychopharmacology *39,* 2366-2375.

Kruzich, P.J., and Grandy, D.K. (2004). Dopamine D2 receptors mediate two-odor discrimination and reversal learning in C57BL/6 mice. BMC Neurosci. *5*, 12.

Kruzich, P.J., Mitchell, S.H., Younkin, A., and Grandy, D.K. (2006). Dopamine D2 receptors mediate reversal learning in male C57BL/6J mice. Cogn. Affect. Behav. Neurosci. *6*, 86–90.

Kwak, S., Huh, N., Seo, J.-S., Lee, J.-E., Han, P.-L., and Jung, M.W. (2014). Role of dopamine D2 receptors in optimizing choice strategy in a dynamic and uncertain environment. Front. Behav. Neurosci. *8*.

Ottenheimer, D.J., Bari, B.A., Sutlief, E., Fraser, K.M., Kim, T.H., Richard, J.M., Cohen, J.Y., and Janak, P.H. (2020). A quantitative reward prediction error signal in the ventral pallidum. Nat. Neurosci. *23*, 1267–1276.

Piray, P. (2011). The role of dorsal striatal D2-like receptors in reversal learning: a reinforcement learning viewpoint. J. Neurosci. *31*, 14049–14050.

Sadacca, B.F., Jones, J.L., and Schoenbaum, G. (2016). Midbrain dopamine neurons compute inferred and cached value prediction errors in a common framework. Elife *5*.

Tooley, J., Marconi, L., Alipio, J.B., Matikainen-Ankney, B., Georgiou, P., Kravitz, A.V., and Creed, M.C. (2018). Glutamatergic Ventral Pallidal Neurons Modulate Activity of the Habenula–Tegmental Circuitry and Constrain Reward Seeking. Biol. Psychiatry *83*, 1012–1023.

Webster, J.F., and Wozny, C. (2020). Behavior: Local Lateral Habenula Interneurons Mediate Aggression. Curr. Biol. *30*, R954–R956.

Yang, H., de Jong, J.W., Tak, Y., Peck, J., Bateup, H.S., and Lammel, S. (2018). Nucleus accumbens subnuclei regulate motivated behavior via direct inhibition and disinhibition of VTA dopamine subpopulations. Neuron *97*, 434–449.e4.
